# Overexpression of α (1,6) fucosyltransferase in the development of castration-resistant prostate cancer cells

**DOI:** 10.1038/s41391-017-0016-7

**Published:** 2018-01-16

**Authors:** Naseruddin Höti, Shuang Yang, Yingwei Hu, Punit Shah, Michael C. Haffner, Hui Zhang

**Affiliations:** 1 Department of Pathology, Johns Hopkins University School of Medicine, Baltimore, MD 21231 USA; 2 Sidney Kimmel Comprehensive Cancer Center, Johns Hopkins University School of Medicine, Baltimore, MD 21231 USA

## Abstract

Glycosylation is recognized as one of the most common modifications on proteins. Recent studies have shown that aberrant expression of α (1,6) fucosyltransferase (FUT8), which catalyzes the transfer of fucose from GDP-fucose to core-GlcNAc of the N-linked glycoproteins, modulates cellular behavior that could lead to the development of aggressive prostate cancer. While the relationship between the abnormal expression of FUT8 and glycoprotein fucosylation in different prostate cancer cells has been demonstrated, there is no evidence that shows dysregulated fucosylation might be involved in prostate cancer progression from androgen-dependent to castration-resistant prostate cancer. In this study, using a proteomics approach, we analyzed androgen-dependent and androgen-resistant LAPC4 cells and identified FUT8 to be significantly overexpressed in the androgen-resistant LAPC4 cells. These findings were independently confirmed in LAPC4 cells that were treated with non-steroidal anti-androgen (bicalutamide) and in the in vivo castrated tumor xenograft models. Similarly, we also demonstrated that overexpression of FUT8 might be responsible for the decreased PSA expression in prostate cancer specimens. To our knowledge, this is the first study reporting the functional role of fucosylated enzyme in the development of castration-resistant prostate cancer.

## Introduction

Prostate cancer is the most common and the second leading cause of cancer death in men in the United States [1]. Clinically, organ-confined prostate cancer is managed through surgery or localized radiation therapy; however, for some patients who recur systemically following treatment or in advanced high-risk prostate cancer patients or metastatic disease, the mainstay of treatment is androgen-deprivation therapy (ADT) [2]. However, long-term ADT leads to the emergence of resistance mechanisms and ultimately the disease progresses to a castration-resistant phenotype which is fatal [3]. This transformation from a clinically localized hormone-dependent state to the androgen-resistant phenotype may involve changes in androgen receptor (AR) and associated pathways [4, 5].

Fucosylation of glycoproteins has been shown to play pivotal roles in many aspects of biological processes such as lymphocyte homing, immune responses, fertilization, and development [6]. Moreover, aberrant fucosylation, which results from the deficiency or overexpression of fucosyltransferases (FUTs), is associated with a variety of human diseases, including cancers [7, 8]. Unlike other FUTs that are functionally redundant, the α (1,6) fucosyltransferase (FUT8) is the only enzyme responsible for the α 1,6-linked (core) fucosylation that adds fucose to the inner most GlcNAc of an N-linked glycan [9]. A growing body of evidence indicates that core fucosylation is important for regulating protein functions [10, 11]. Transgenic and knockout animal models for core fucosylation have been generated to study the physiological role of FUT8 [12, 13]. Ectopic expression of FUT8 resulted in the steatosis-like phenotype in transgenic mice [14], while on the other hand, knocking out FUT8 in mice was reported to dramatically decrease the postnatal survival and inhibition of chemical-induced hepatocellular carcinoma and tumorigenesis [1, 2]. Core fucosylation is also crucial for the ligand-binding affinity of transforming growth factor (TGF)-β1 receptor, epidermal growth factor (EGF) receptor [15], and integrin α3β1 [16]. Lack of the core fucose on these receptors leads to a marked reduction in ligand-binding ability and downstream signaling. Furthermore, an increase in core fucosylation on E-cadherin has been shown to strengthen cell–cell adhesion [17].

Overexpression of FUT8 has been observed in several malignant tumors, which is linked to the severity of these cancers [18, 19]. In papillary thyroid carcinoma, higher expression of FUT8 is linked to larger tumor volumes and lymph node metastasis [20]. Similarly, in prostate cancer, we have previously observed and reported higher FUT8 expression in aggressive tumors (Gleason 8 and above) compared to its non-aggressive Gleason 6 and lower [21]. In addition, we have also reported that overexpression of FUT8 in prostate cancer cells was correlated with increased fucosylation of glycoproteins in aggressive prostate cancer cells [22]. Here we report that FUT8 overexpression was induced in castration-resistant cells and was responsible for the lower prostate-specific antigen (PSA) production and cell survival in prostate cancer.

## Materials and methods

### Cell lines and reagents

The prostate cancer LAPC4 cell lines that harbor the wild-type AR, that was regularly validated by DNA typing, were obtained from Dr. Johns Isaacs in 2016 (Johns Hopkins School of Medicine), which were cultured in Iscove’s Modified Dulbecco’s Medium (IMDM) containing 10% charcoal-stripped fetal bovine serum (cFBS) (GIBCO, Carlsbad, CA), 100 U of penicillin, and 100 µg/mL of streptomycin in the presence of 5 nM synthetic androgen R1881. PC3 cell lines were previously obtained from American Type Culture Collection (Manassas, VA, USA) and were maintained in T-75 flasks in RPMI-1640. The LNCaP-95 cell line, an androgen-independent prostate cancer cell line, was provided by Dr. Alan K. Meeker (Johns Hopkins University) and has been previously described [23, 24]. LNCaP-95 cells were maintained in Phenol red-free and 10% cFBS media. The LAPC4-AI cell line was developed from the LAPC4 wild-type cells in our lab by continuous culturing in cFBS for a prolonged period of 6 months. All cell lines were routinely tested for mycoplasma contamination using the American Type Tissue Culture Universal Mycoplasma Detection Kit. Primary mouse monoclonal antibodies for AR (AR 441, dilution 1:1000) and polyclonal AR (N-20, dilution 1:1000) were from Santa Cruz Biotech (Santa Cruz, CA), the polyclonal FUT8 antibody was from R&D systems (Minneapolis, MN), and the polyclonal PSA antibody (1:1000) was from DAKO (Carpinteria, CA). Similarly, mAb-β Actin (1:25,000), the anti-mouse and the anti-rabbit IgG HRP-conjugated (1:20,000) were from Sigma Aldrich (St. Louis, MO) while the anti-sheep IgG HRP-conjugated (1:20,000) was from Thermo Fisher Scientific (Grand Island, NY). The majority of all other chemical reagents and compounds were ordered from Sigma Aldrich, unless otherwise specified. Aminolink resin, spin columns (snap cap), and Zeba spin desalting columns were purchased from Life Technologies (Grand Island, NY); Alltech Extract-Clean Carbograph columns were from Fisher Sci. (Waltham, MA). Peptide-N-glycosidase F (PNGase F), denaturing buffer (10×), and reaction buffer (G7; 10×) were from New England Biolabs (Ipswich, MA). Trypsin gold was from Promega (Madison, WI). Orbitrap Velos LC-MS (Thermo) was used for quantitative proteomics analysis.

### iTRAQ labeling of global tryptic peptides from cell lines

Each iTRAQ (isobaric tags for relative and absolute quantitation) 4-plex reagent was dissolved in 70 μL of ethanol. One milligram of each tryptic peptide sample was added into 250 µL of iTRAQ dissolution buffer, then mixed with iTRAQ 4-plex reagent and incubated for 1 h at room temperature. iTRAQ channels 114 and 115 were used to label LAPC4 WT samples; iTRAQ 116 and 117 were used for labeling the LAPC4-AI cells. The 4 sets of tagged peptides were combined and purified by SCX column. Then, 10% of the labeled peptides were dried and re-suspended into 0.4% acetic acid solution prior to fractionation for mass spectrometry analysis.

### Peptide fractionation using basic RPLC

The high-pH reverse-phase liquid chromatography (RPLC) separation was performed on the 1220 Infinity LC system with a Zorbax Extended-C18 analytical column containing 1.8 µM particles (Agilent Technologies Inc., CA); flow rate was 0.2 mL/min. The mobile-phase A consisted of 10 mM ammonium formate (pH 10) and B consisted of 10 mM ammonium formate and 90% acetonitrile (pH 10). A total of 50 µg peptides were fractionated using the following linear gradient: from 0% to 2% B in 10 min, from 2% to 8% B in 5 min, from 8% to 35% B in 85 min, from 35% to 95% B in 5 min, and then held at 95% B for an additional 15 min. Peptides were detected at 215 nm and 96 fractions were collected along with the LC separation in a time-based mode from 16 to 112 min. The separated peptides in 96 wells were concatenated into 24 fractions by combining four wells into one sample, such as 1, 25, 49, and 73 as fraction one; 2, 26, 50, and 74 as fraction two; and so on. The peptides were then dried in a Speed-Vacuum and stored at −80 °C until liquid chromatography-tandem mass spectrometry (LC-MS/MS) analysis as described in our Supplemental Materials and Methods section.

### LC-MS/MS

Peptides were re-suspended in 0.1% formic acid in water and subjected to LC-MS/MS analysis. Peptides from each fraction were separated on a Dionex Ultimate 3000 RSLCnano system (Thermo Scientific) with a 75 µm × 15 cm Acclaim PepMap100 separating column (Thermo Scientific) and with a 2 cm guarding column (Thermo Scientific). The flow rate was 300 nL/min with 0.1% formic acid in water (A) and 0.1% formic acid, 95% acetonitrile (B). The gradient profile was 5–40% B for 90 min. MS analysis was performed using an Orbitrap Velos Pro mass spectrometer (Thermo Scientific). The spray voltage was set at 2.2 kV. Orbitrap spectra (AGC 1 × 10^6^) were collected with resolution of 30,000 followed by 10 data-dependent HCD MS/MS (at a resolution of 7500, collision energy 35%, activation time 0.1 ms). A dynamic exclusion time was set at 25 s with a repeat count of 2.

### Protein expression data analysis

MS/MS Data and Differential Expression Analysis: we searched our tandem mass spectrometry derived raw data against the RefSeq protein database using the SEQUEST search engine in Proteome Discoverer v1.4. We specified carbarmidomethylation of cysteine, lysine (K) iTRAQ modifications, and N-terminal iTRAQ modification as fixed residue modifications. We specified deamidation at asparagine and oxidation of methionine as dynamic modifications. Peptide identification false discovery rate (FDR) was specified as 0.01. Parsimonious protein grouping was specified to allow at least one peptide per protein. High-confidence peptide spectrum matches (PSMs) (i.e., PSMs better than pre-specified FDR cutoff) were used for protein grouping. Peptide and protein quantifications were based on ratios of iTRAQ reporter ions: 114, 115, 116, and 117. Our specified reporter ion quantification LAPC4-wt/LAPC4-wt was 115/114. The LAPC4-AI/LAPC4-WT ratios done in duplicate were 116/114 and 117/114. The variability between the replicates were very minimal; therefore no normalization was performed for quantification. Proteome Discoverer reports coefficient of variations (CVs) of unique PSMs’ reporter ion ratios. As a second-level quality control measure, we set an acceptable limit at less than or equal to 30%. We filtered out PSMs and associated protein with reporter ion ratio CVs greater than 30%.

## Results

### Development of androgen-resistant cell line and mass spectrometry-based proteomic analysis

**Fig. 1:**
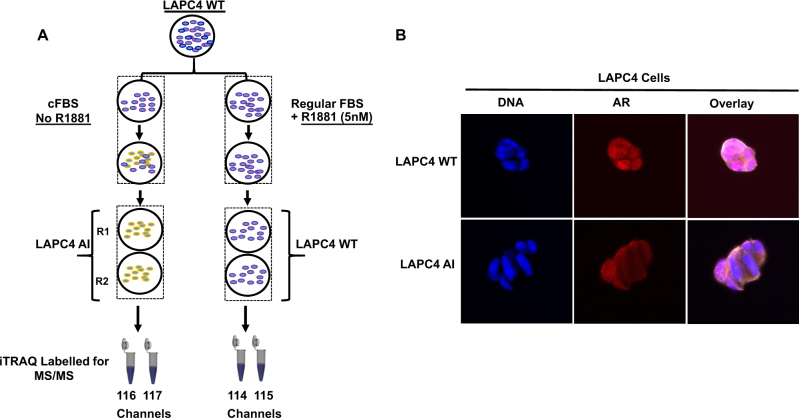
Schematic showing the workflow for generating LAPC4-AI cell line and iTRAQ 4 plex (114, 115, 116, and 117) labeling of the samples for mass spectrometer analysis (**a**). The cytoplasmic and nuclear localization of AR in LAPC4-WT and LAPC4-AI cells (**b**) (online color figure)

The wild-type LAPC4 prostate cancer cell line was continuously cultured in charcoal dextran-stripped FBS-containing medium for more than 6 months to obtain androgen-resistant LAPC4-AI cells as shown by schematics (Fig. 1a). We evaluated the AR status of these cells by imunofluorescence (IF) microscopy and compared the AR localization between the LAPC4-AI grown in cFBS-containing medium and the wild-type LAPC4 cells that were grown in normal FBS-containing media supplemented with 1 nM of synthetic androgens (R1881). As shown in Fig. 1b, cells that were resistant to androgen (LAPC4-AI) had a predominant cytoplasmic AR localization compared to the wild-type LAPC4 cells, where the majority of the AR was found inside the nucleus. To evaluate whether the changes in protein expression might play a role in the development of castration-resistant phenotype, we performed a global proteomic analysis using LC-MS/MS on these cells. Briefly, cell lysates from the wild-type LAPC4 and androgen-resistant LAPC4-AI were subjected to the 4-plex iTRAQ labeling before combining them together (Supplemental Fig. 1). Fifty micrograms of protein samples labeled with iTRAQ were subjected to basic reverse-phase liquid chromatography (bRPLC). A total of 96 fractions were collected and concentrated into 24 fractions. Each bRPLC fraction was analyzed by LC-MS/MS to obtain both qualitative and quantitative information on the proteome of LAPC4 and LAPC4-AI cells. At 1% spectral FDR, a total of 3365 protein groups were identified with a minimum of two peptides per protein (Supplemental Table S1).

### FUT8 overexpression in androgen-resistant LAPC4 cells

**Fig. 2:**
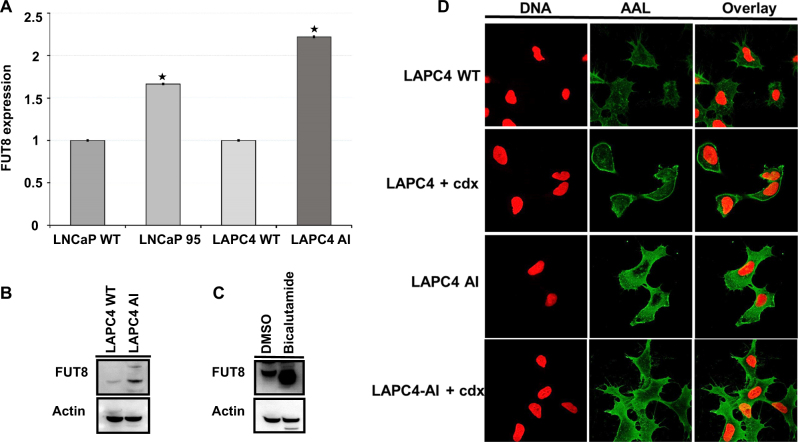
Relative quantitative qRT-PCR analysis for mRNA expression of FUT8 in wild-type (LNCaP and LAPC4) and in androgen-resistant LNCaP-95 and LAPC4-AI prostate cancer cells. Error bars represent mean ± SE and * indicates *P* ≤ 0.05 (**a**). Western blot analysis of FUT8 in LAPC4-WT and LAPC4-AI prostate cancer cells (**b**). Western blot analysis of FUT8 in LAPC4-WT cells treated with or without bicalutamide (10 µM) for 72 h (**c**). Confocal microscopy for the AAL lectin in LAPC4-WT or bicalutamide (cdx) treated or LAPC4-AI and LAPC4-AI treated with 10 µM of bicalutamide (**d**) (online color figure)

To evaluate whether glycosylation was related to androgen resistance, we searched for the detected N-linked glycosylated related enzymes in the globally identified proteins and were able to detect a total of 20 N-linked glycosylated related enzymes (Table 1). To determine which N-Linked glycosylated related enzymes were differentially expressed between the LAPC4-AI androgen-resistant and wild-type prostate cancer cells, we compared the iTRAQ ratios between the wild type (114, 115) to that of LAPC4-AI (116, 117). As shown in Table 1, FUT8, which is responsible for the core fucosylation of many cellular glycoproteins, was significantly overexpressed (more than 3-fold) in LAPC4-AI cells compared to that of the androgen-sensitive wild-type LAPC4 cells. To further investigate whether overexpression of FUT8 in androgen-resistant prostate cancer cells was due to the upregulation of FUT8 mRNA, we analyzed the LAPC4-AI and LAPC4 wild-type cells along with the LNCaP and LNCaP-95 prostate cancer cell lines using qRT-PCR analysis. The relative quantitative mRNA analysis from wild types (LNCaP and LAPC4) and the androgen-resistant prostate cancer cells (LNCaP-95 and LAPC4-AI) revealed overexpression of FUT8 in both androgen-resistant prostate cancer cell lines (Fig. 2a). In order to determine whether FUT8 protein expression was present in androgen-resistant LAPC4-AI cells, we performed western blot analysis using the wild-type LAPC4 and the androgen-resistant LAPC4-AI cells. As shown in Fig. 2b, FUT8 protein was overexpressed in LAPC4-AI cells compared to that of the wild-type cells. We further examined six different prostate cancer cell lines including the LNCaP-AI (androgen independent) and PC3 (androgen resistant) prostate cancer cells and found higher expression of FUT8 in LNCaP-AI and PC3 cells compared to the wild-type LNCaP cells (Supplemental Fig. 2)

**Table 1:** N-glycosylated related enzymes identified in global iTRAQ data

Gene	Protein	Description	Coverage	# Peptides	# PSMs	Gene IDs	LAPC4-WT (115)/LAPC4 -T (114)	LAPC4-AI (116)/LAPC4-WT (114)	LAPC4-AI (117)/LAPC4-WT (114)
DAD1	Dolichyl-diphosphooligosaccharide--protein glycosyltransferase subunit DAD1	Defender against cell death 1, isoform CRA_a	26.55	3	32	1603	1.310	0.986	1.030
B4GALT1	Beta-1,4-galactosyltransferase 1	Unnamed protein product	8.14	2	6	2683	0.871	0.595	0.582
MAN2A1	Alpha-mannosidase 2	PREDICTED: alpha-mannosidase 2 isoform × 1	7.49	7	18	4124	1.231	0.687	0.691
PRKCSH	Glucosidase 2 subunit beta	PRKCSH protein, partial	13.44	7	47	5589	1.092	0.768	0.743
RPN1	Dolichyl-diphosphooligosaccharide--protein glycosyltransferase subunit 1	Unnamed protein product	53.05	27	322	6184	1.226	0.910	0.901
RPN2	Dolichyl-diphosphooligosaccharide--protein glycosyltransferase subunit 2	RPN2 protein, partial	39.23	13	107	6185	1.138	0.916	0.992
MOGS	Mannosyl-oligosaccharide glucosidase	Mannosyl-oligosaccharide glucosidase isoform 2	15.18	10	37	7841	1.047	0.990	0.986
B4GALT3	Beta-1,4-galactosyltransferase 3	Unnamed protein product	12.78	3	5	8703	0.794	1.262	1.131
DPM1	Dolichol-phosphate mannosyltransferase subunit 1	Dolichyl-phosphate mannosyltransferase polypeptide 1, catalytic subunit	24.23	5	23	8813	1.227	1.031	1.161
ALG3	Dol-P-Man:Man(5)GlcNAc(2)-PP-Dol alpha-1,3-mannosyltransferase	ALG3 protein, partial	5.08	2	5	10,195	1.261	0.811	0.808
GANAB	GANABGANABNeutral alpha-glucosidase AB	Glucosidase, alpha; neutral AB, isoform CRA_d	28.3	20	157	23,193	1.035	1.113	1.074
GANAB	GANABGANABNeutral alpha-glucosidase AB	Glucosidase, alpha; neutral AB, isoform CRA_a	25.73	21	164	23,193	1.047	1.102	1.078
ST8SIA5	Alpha-2,8-sialyltransferase 8E	Asparagine-linked glycosylation 6 homolog isoform CRA_d	6.32	3	5	29,929	1.118	0.776	0.793
DPM3	Dolichol-phosphate mannosyltransferase subunit 3	Dolichyl-phosphate mannosyltransferase polypeptide 3, isoform CRA_a	23.91	2	17	54,344	1.121	0.920	0.881
UGGT1	UDP-glucose:glycoprotein glucosyltransferase 1	UDP-glucose ceramide glucosyltransferase-like 1	10.58	17	37	56,886	1.063	1.085	1.053
ALG8	Probable dolichyl pyrophosphate Glc1Man9GlcNAc2 alpha-1,3-glucosyltransferase	Glucosyltransferase, partial	4.32	2	4	79,053	1.056	0.599	0.629
ALG2	Alpha-1,3/1,6-mannosyltransferase ALG2	Unnamed protein product	8.06	2	5	85,365	1.065	2.104	2.111
RFT1	Protein RFT1 homolog	PREDICTED: protein RFT1 homolog isoform × 4	13.02	6	10	91,869	0.994	0.696	0.745
STT3B	Dolichyl-diphosphooligosaccharide--protein glycosyltransferase subunit STT3B	STT3, subunit of the oligosaccharyltransferase complex, homolog B	18.04	15	108	2E+05	1.163	1.031	1.029
FUT8	Alpha-(1,6)-fucosyltransferase	Chain X, crystal structure of human alpha 1,6-fucosyltransferase, Fut8	14.26	8	18	2530	0.949	3.041	3.047

To evaluate if pharmacological inhibition of AR signaling with the non-steroidal competitive AR antagonist bicalutamide exerts similar changes in FUT8 expression, we treated wild-type LAPC4 cells with 1 µM of bicalutamide or mock control (DMSO) in normal growth medium. Cells harvested after 72 h of treatment were subjected to western blot analysis for FUT8 expression. Beta actin was included to ensure equal amount of loading across the lanes. As shown in Fig. 2c, there was a significant increase in the FUT8 expression compared to the mock-treated controls. We next sought to determine whether the increased levels of FUT8 expression in castration-resistant LAPC4-AI cells were functional. We evaluated the fucosylated status of the wild-type and androgen-resistant LAPC4 cells by performing the *Aleuria* *aurantia* lectin (AAL) analysis using confocal microscopy and by AAL blot analysis. AAL has been previously shown to specifically bind to the α (1,6)-linked core fucose of the N-acetylglucosamine of glycopeptides. As shown in Fig. 2d, a higher staining of AAL was observed in bicalutamide-treated (casadox, cdx) or androgen-resistant LAPC4-AI cells compared to the wild type suggesting a functional overexpression of FUT8 in castration-resistant cells (Fig. 2d). Similar results were obtained by AAL blot in LAPC4-AI cells when compared to the LAPC4 wild-type cells (Supplemental Fig. 3A).

### Effect of surgical castration on the FUT8 expression in LAPC4 xenograft animal model

**Fig. 3:**
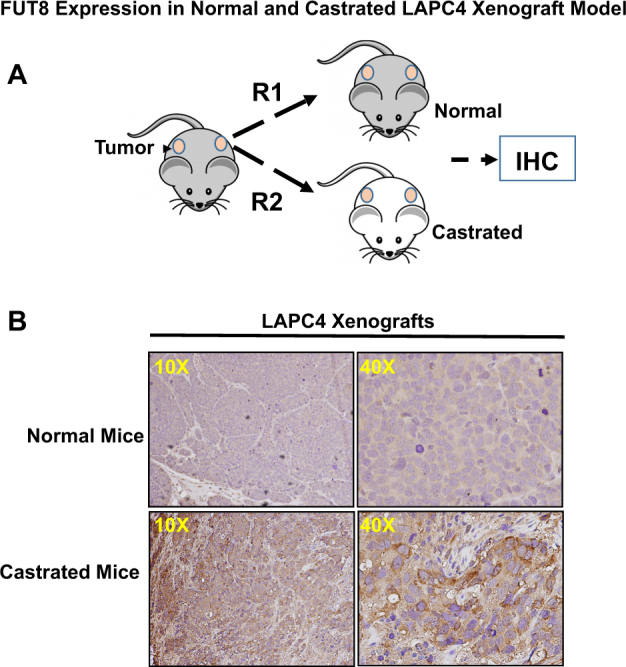
Schematic showing LAPC4 cells implanted as xenografts on the lower back of athymic male nude mice; after the tumor volume reached around 1 cm, mice were either castrated or mock operated (**a**). Tumors were harvested at the end of experiments and subjected to IHC for FUT8 expression (**b**) (online color figure)

Prostate cancer xenograft mimics many features of human prostate tumors, which makes it a valuable model to study the mechanisms of progression to castration-resistant prostate cancer. To further determine whether androgen ablation was indeed responsible for the overexpression of FUT8 in prostate cancer, we moved to a more biological relevant mice model. LAPC4 wild-type cells at a density of 1 × 10^6^ in PBS were mixed in 1:3 ratio with 1× Matrigel (BD biosciences) and implanted into the dorsal flanks of athymic nude mice. Once tumors were established and reached to tumor volume of ~1 cm^3^, the animals were divided into two groups. Castration was performed in one group of animals by bilateral orchiectomy. Mice in control group were shame-operated under anesthesia to localize sex organs but without orchiectomy. At the end of the experiment (5 weeks after castration), all animals were sacrificed and tumors were removed for immunohistochemical studies. As shown in Fig. 3, the animals that were castrated have a significantly higher FUT8 staining in the tumor xenograft compared to that of the normal uncastrated animals. These data independently confirm FUT8 overexpression in the in vivo castration-resistant LAPC4 prostate cancer xenograft model.

### Overexpression of FUT8 reduces the production of PSA in prostate cancer cells

**Fig. 4:**
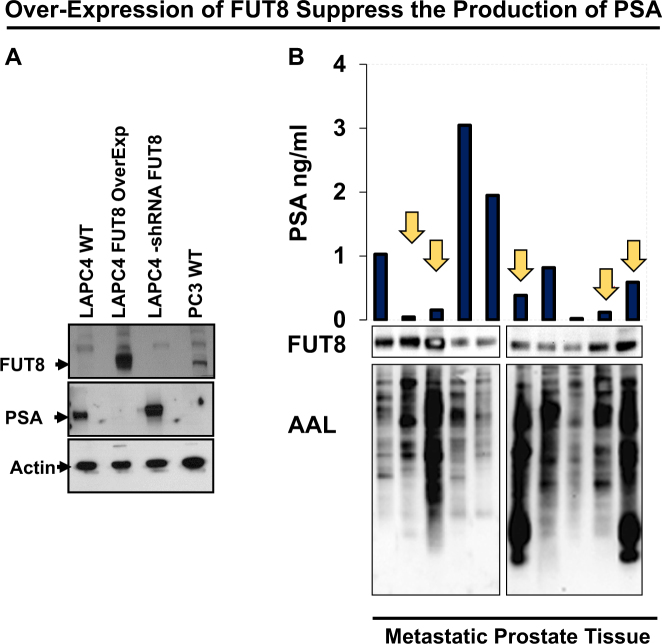
Western blot analysis for PSA and FUT8 in LAPC4 cells that were transfected with plasmid carrying wild-type FUT8 or shRNA against FUT8 or vector control. PC3 cells were included as a positive control for FUT8 and negative control for PSA expression. Beta actin was included to ensure equal amount of loading across the lanes (**a**). Inverse association between FUT8 and PSA expression in 10 metastatic prostate tumor samples (*R* = −0.321). AAL blot was included to confirm the functional increase of FUT8 in all 10 samples (**b**) (online color figure)

FUT8 expression was induced by androgen ablation in LAPC4 cells. We next inquired whether exogenous expression of FUT8 in the LAPC4 cells had any biological activity on the PSA expression in these cells. LAPC4 cells that were stably selected to express FUT8 (pCMV6-FUT8-Myc-DDK) or shRNA against FUT8 (pLKO-shRNA FUT8) or empty vectors (pCMV6 were subjected to western blot analysis for PSA and FUT8 expression). Protein lysates from PC3 cells, an androgen-resistant prostate cancer cell line that had previously shown to express higher levels of FUT8, were included as a positive control for FUT8 expression. As shown in Fig. 4a, LAPC4 cells that were transfected with FUT8 overexpressing plasmid had a significant reduction in the PSA expression compared to the mock-transfected cells. Similarly, knocking down FUT8 in the LAPC4 cells causes an increase in the total PSA suggesting an inhibitory role of FUT8 in the PSA signaling. To further confirm these findings, we evaluated 10 different metastatic prostate cancer tissues for PSA and FUT8 expression using western blot analysis. The AAL blot analysis was also performed using the same lysates to confirm the functional FUT8 overexpression in these specimens. As shown in Fig. 4b, tissue samples that had higher expression of FUT8, except Lane 8, had a significantly lower PSA production and vice versa, which was in accordance with our in vitro western blot analysis (Fig. 4a) suggesting the inhibitory role of FUT8 on the PSA expression in metastatic prostate cancer tissues.

### Overexpression of FUT8 support cell proliferation in castrated condition

**Fig. 5:**
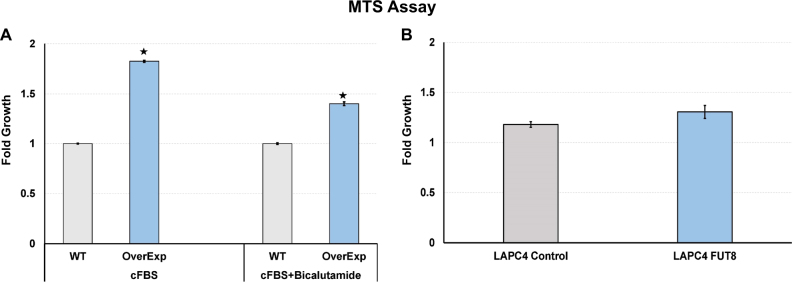
MTS assay was performed to evaluate the proliferation of LAPC4-WT and LAPC4 cells that were stably selected to overexpress FUT8 in cFBS-containing media or in the presence of bicalutamide (10 µM) (**a**). LAPC4 control or LAPC4-FUT8 cells grown in regular growth media containing 10% FBS and 5 nM R1881 (**b**). Error bars indicate ± SEM and * indicates *P* ≤ 0.05 (online color figure)

To further demonstrate whether FUT8 overexpression in prostate cancer cells promotes the proliferation of cells in normal or androgen-ablated conditions, we preformed the MTS assay on the LAPC4 cells that were stably selected to overexpress FUT8 (LAPC4-FUT8) or control vectors (LAPC4 Ctr). Briefly, LAPC4-FUT8 or control cells plated at the density of 2 × 10^4^ cells in quadruplets were grown in the cFBS-containing IMDM media or cFBS-containing media with 10 µM bicalutamide or DMSO for 3 days or in normal FBS-containing media. By the end of 3 days, cells were subjected to the MTS assay to examine cell growth. Data from the MTS assay were normalized to the 0 h reading from the replica plates. Fold growth was calculated by normalizing MTS readings to the wild-type control cells. As shown in Fig. 5a, cells that were selected to overexpress FUT8 (LAPC4-FUT8) had significantly higher readings compared to the wild-type control. Similarly, in the presence of bicalutamide (10 µM), more of LAPC4-FUT8 cells were detected by the MTS assay compared to the wild-type control cells. We also evaluated the proliferation of LAPC4-FUT8 and control cells in normal FBS-containing IMDM media supplemented with 1 nM of R1881 and found no growth differences between LAPC4-FUT8 and control cells (Fig. 5b). In addition to the LAPC4 cell line model, we also confirmed these observations in the LNCaP model that was stably selected to express FUT8 (LNCaP-FUT8) (Supplemental Fig. 3B). Together, these data suggest a positive role of FUT8 in the castration resistance biology of prostate cancer. Further studies are underway in our laboratory to explicitly identify the mechanisms underlying these phenomena.

## Discussion

One of the underlying cause of glycosylation changes in cancer is the dysregulated expression of glycosyltransferases and associated proteins within the cancer cell [25]. Fucosylated glycans have been shown to play essential roles in many aspects of mammalian processes [6], while their dysregulation was responsible for a variety of human diseases including cancers [7, 8]. Unlike other FUTs, FUT8 is the only enzyme responsible for the α 1,6-linked (core) fucosylation of proteins. Our initial studies with FUT8 in prostate cancer specimens demonstrated a strong correlation between FUT8 expression and aggressive prostate cancer. In this study, we discovered a novel role of FUT8 overexpression, which might be responsible for driving castration resistance in prostate cancer. Using a global proteomic approach, we were able to identify FUT8 as one of the significantly overexpressed glycosyltransferases proteins in the LAPC4-AI cells, which were further verified by other androgen-resistant models (Figs. 2, 3).

AR signaling is a major player in all aspects of prostate biology [26]. AR activation is responsible for the development of the prostate and is required in the control of prostatic cell growth, differentiation, function, and survival [5]. During prostate cancer, it is the aberrant AR signaling that results in the uncontrolled proliferation and survival of prostate cancer cells, especially in advanced hormone refractory disease where AR becomes active in the absence of a ligand. Blocking the AR pathway in prostate cancer patients can be achieved by several mechanisms including surgical orchiectomy or chemically with luteinizing hormone-releasing hormone (LHRH) agonists, LHRH antagonists, or anti-androgens [27, 28]. Although these therapies do seem to work, the effects on prostate cancer cells are very transient [29]. To demonstrate whether glycosyltransferase was involved in the development of this phenotype, we treated the LAPC4 cells with bicalutamide, a non-steroidal anti-androgen and identified FUT8 to be overexpressed in these cells suggesting a role of glycosylation in the biology of androgen ablation (Fig. 1c). Interestingly, we had previously shown negligible levels of FUT8 expression in LNCaP cells compared to the more aggressive PC3 prostate cancer cells [21]. However, when LNCaP cells were grown in charcoal-stripped media for several passages (LNCaP-95), there was an increase in the FUT8 expression (Fig. 1a) indicating androgen ablation as a requisite for the FUT8 overexpression in the AR-positive prostate cancer cells. Similarly, we found that the increase in FUT8 levels in these cells was a functional increase as the ALL microscopy (Fig. 2d) and lectin blot (Supplemental Fig. 3A) showed higher levels of staining in the LAPC4-AI or LAPC4-AI cells treated with bicalutamide.

The glycosylation on the cell surface proteins including EGF receptor (EGFR) [30] has been shown to play a role in cancer biology and cellular proliferation [31, 32]. Transforming growth factor-α which is known to specifically bind the EGFR and activates the intracellular signaling pathways was shown to play role in the prostate cell growth [33]. Therefore, there was a plausible hypothesis that overexpression of FUT8 may contribute to the expression profiles of EGFR or Transforming growth factor-α. However, based on the LC-MS/MS iTRAQ data, we found downregulated expression of EGFR and TGF-α proteins, thus nullifying the role of EGFR and TGF-α in androgen-resistant LAPC4-AI development. To further investigate the phenomena of FUT8–androgen nexus, we recapitulated the androgen ablation condition using surgical orchiectomy LAPC4 mouse xenograft model (Fig. 3). Our findings revealed that FUT8 overexpression was in fact driven by androgen ablation in prostate cancer cells. To better understand whether overexpression of FUT8 might be responsible for castration-resistant phenotype in prostate cancer cells, we ectopically overexpressed FUT8 in LAPC4 cells and found that overexpression of FUT8 in LAPC4 cells suppressed the production of PSA, while knocking down the endogenous FUT8 with shRNA in LAPC4 cells significantly enhanced PSA production (Fig. 3a). These results were in accordance with the patient samples where overexpression of FUT8 had lower PSA values and vice versa (Fig. 3b). Further studies are underway in the laboratory to understand whether FUT8 overexpression acts as a driver in the development of castration-resistant phenotypes, which may be used as a therapeutic target to overcome prostate cancer resistance to anti-androgen-based therapies.

## Conclusion

We presented evidence that dysregulated fucosylation might play a role in the development of castration-resistant prostate cancer, thus extending the notion to include aberrant glycosylation and glycosylated related enzymes to the growing list of mechanistic pathways involved in prostate cancer.

## Electronic supplementary material

Figures 1–3

Materials and Methods

Supplemental Table 1

Supplemental Table 1
