# Supplementary material for: Overexpression of α (1,6) fucosyltransferase in the development of castration-resistant prostate cancer cells
Source: Prostate Cancer Prostatic Dis. 2018 Jan 16;21(1):137–46. doi: 10.1038/s41391-017-0016-7 (PMC5895601; doi:10.1038/s41391-017-0016-7)
Supplement: Supplementary file 1 — Figures 1–3 [file 41391_2017_16_MOESM1_ESM.pptx]

## Slide 1
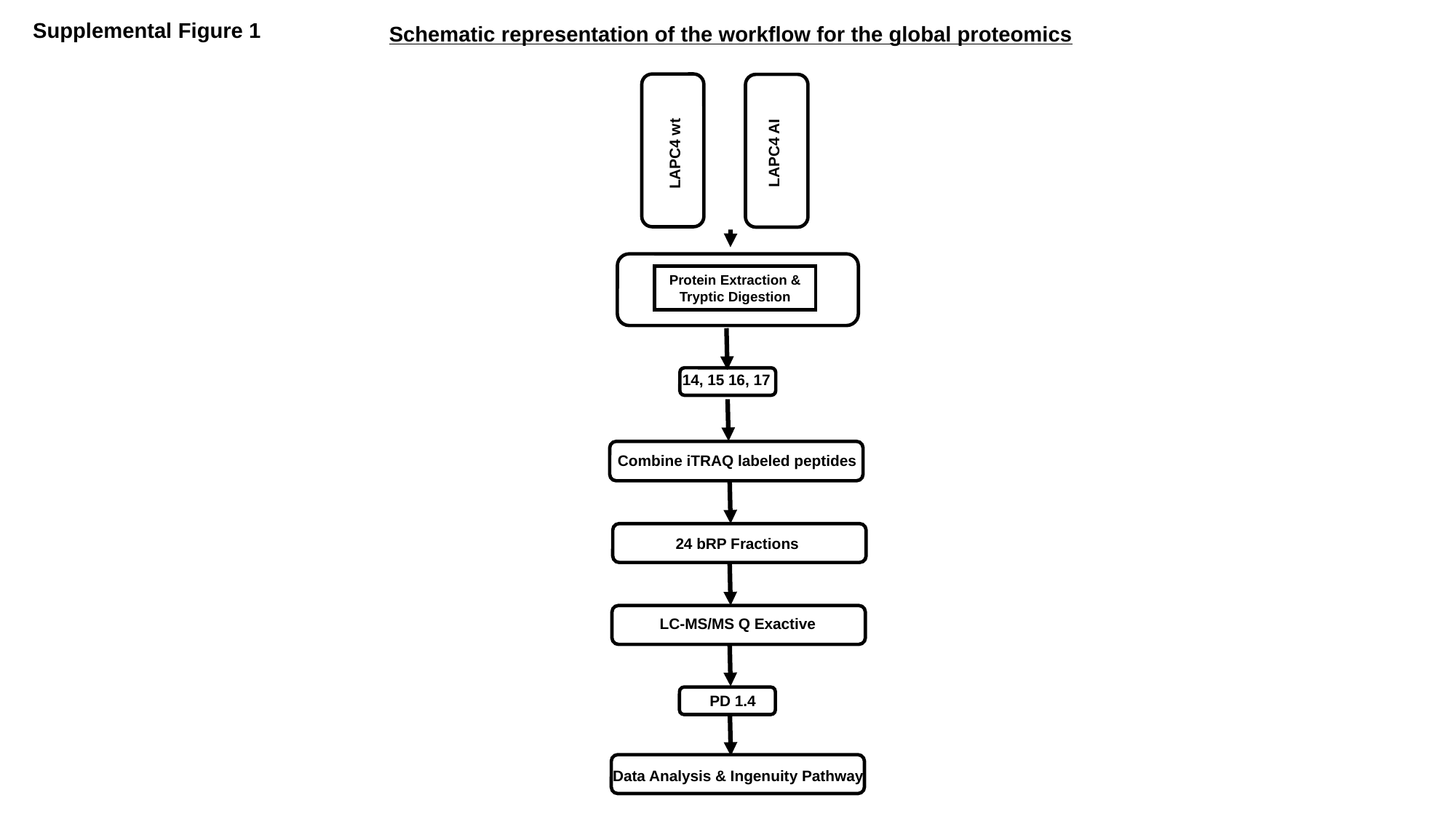

Supplemental Figure 1
Schematic representation of the workflow for the global proteomics
LAPC4 AI
LAPC4 wt
Protein Extraction & Tryptic Digestion
14, 15 16, 17
Combine iTRAQ labeled peptides
24 bRP Fractions
LC-MS/MS Q Exactive
PD 1.4
Data Analysis & Ingenuity Pathway

## Slide 2
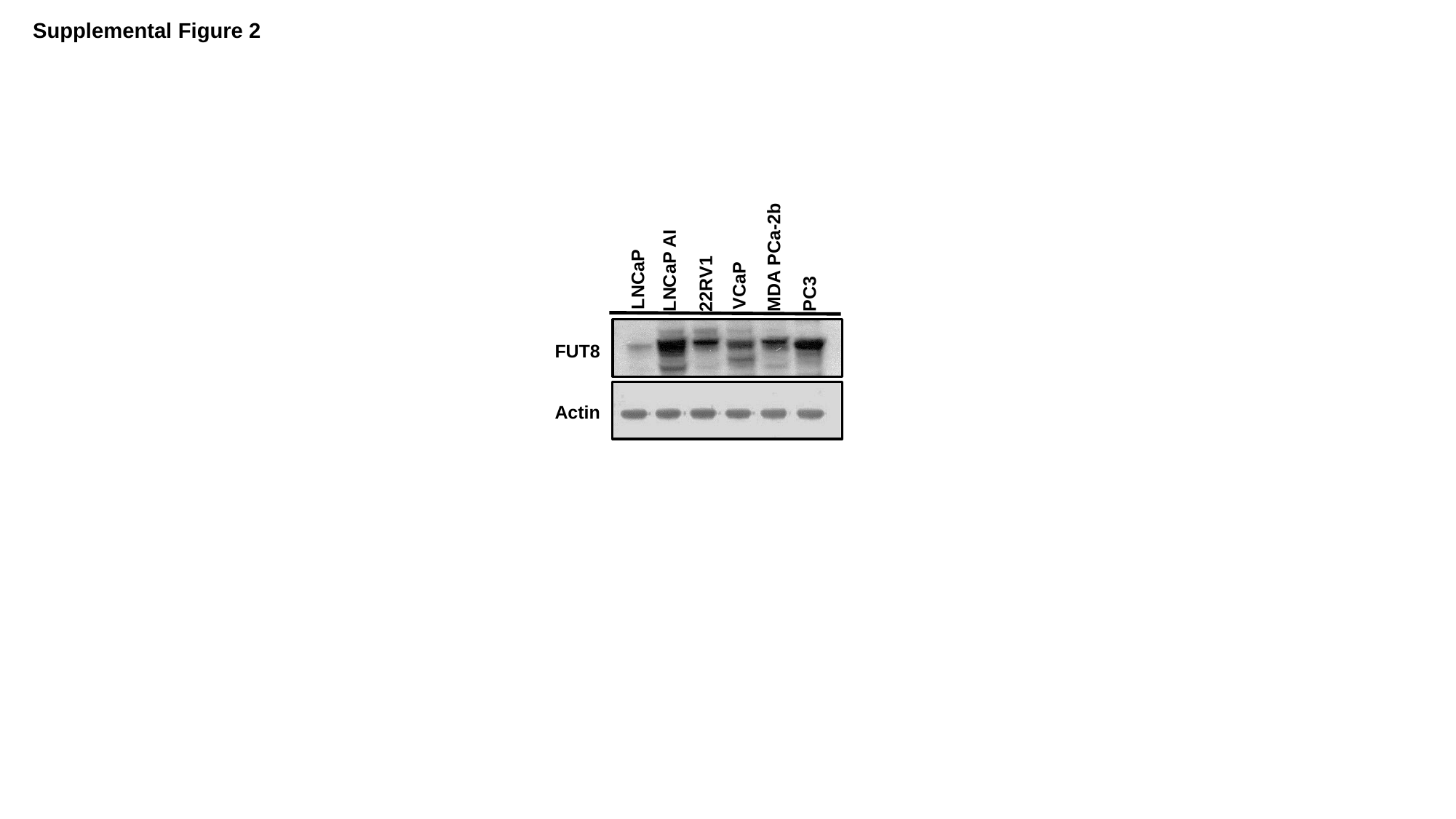

Supplemental Figure 2
LNCaP
VCaP
LNCaP AI
22RV1
MDA PCa-2b
PC3
FUT8
Actin

## Slide 3
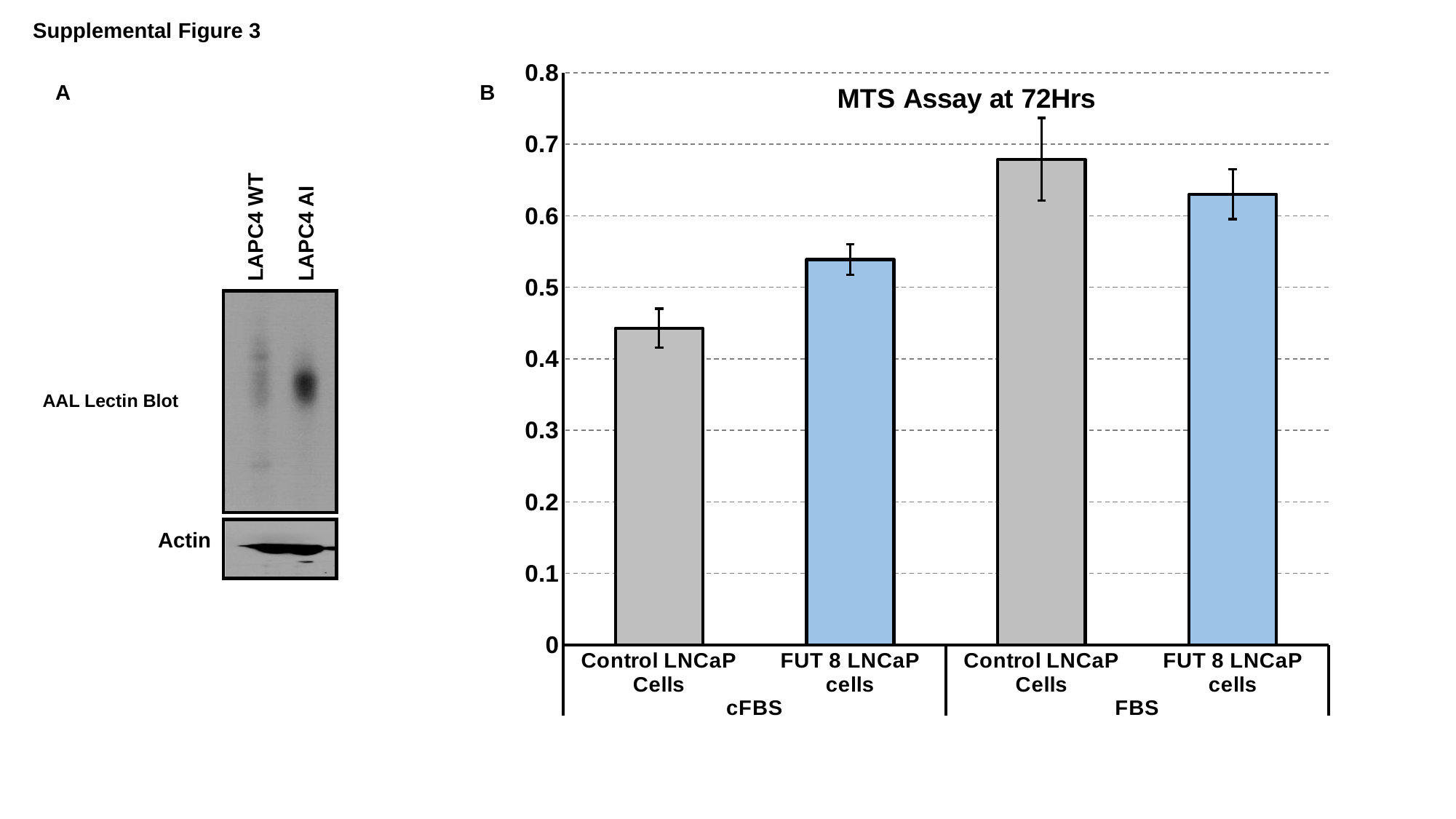

Supplemental Figure 3
### Chart
| Category | AVG-bg |
|---|---|
| Control LNCaP Cells | 0.44293750000000004 |
| FUT 8 LNCaP cells | 0.5389562499999999 |
| Control LNCaP Cells | 0.6789624999999999 |
| FUT 8 LNCaP cells | 0.630275 |B
A
LAPC4 WT
LAPC4 AI
AAL Lectin Blot
Actin
