# Supplementary material for: Overexpression of α (1,6) fucosyltransferase in the development of castration-resistant prostate cancer cells
Source: Prostate Cancer Prostatic Dis. 2018 Jan 16;21(1):137–46. doi: 10.1038/s41391-017-0016-7 (PMC5895601; doi:10.1038/s41391-017-0016-7)
Supplement: Supplementary file 2 — Materials and Methods [file 41391_2017_16_MOESM2_ESM.docx]

**Supplemental Materials and Methods:**

**Q-RT PCR analysis:** mRNA (1 μg) from wild type LAPC4 or LNCaP and the androgen resistant LNCaP-95 and the LAPC4-AI cells were each reverse transcribed using QuantiTect Reverse Transcription Kit (Qiagen). Syber green–based real-time qRT-PCR was performed using SYBR GreenER qPCR SuperMix (Invitrogen) according to the manufacturer’s instructions. Standard curves were generated by serial dilution of each sample, and the relative amount of FUT8 gene mRNA in the cells lines were normalized to Actin mRNA using the following specific set of primers

FUT8-Forward: GACAGAACTGGTTCAGCGGAGA

FUT8 Reverse: GCAGTAGACCACATGATGGAGC

Actin-Forward: GTACCACTGGCATCGTGATGGACT

Actin- Reverse: CCGCTCATTGCCAATGGTGAT

**Western blot analysis:** Western blotting was done to confirm some of the protein identified in our Mass-Spec analysis. Briefly, cells were washed with 1 × PBS and re-suspended with five volumes of cold lysis buffer (50 mM Tris-HCl, pH 7.5, 250 mM NaCl, 5 mM EDTA, 50 mM NaF, 0.5% NP-40) supplemented with protease inhibitor cocktail (Roche, Indianapolis, IN). The cell lysate was incubated on ice for 30 min and then centrifuged for 10 min at 4 °C. Equal amounts of proteins were separated by SDS-PAGE, and the resolved proteins were then transferred to a nitrocellulose membrane. After blocking with 5% nonfat milk in TBST overnight at 4 °C, the blot was incubated with primary antibody at 1 h at room temperature. The membrane was then probed with HRP-conjugated secondary antibody for 1 h and developed (ECL-Plus system, Amersham Pharmacia, Piscataway, NJ) using the manufacturer's protocol.

**Proteomic Analysis of Prostate Cancer Cells:** The detailed procedures are described in our previous studies ^25,26^. Proteins were extracted from cell pellets by sonication in RIPA buffer (1x PBS, 1% NP-40, 0.5% sodium deoxycholate, 0.1% SDS, 2mM EDTA, and 50 mM NaF). The supernatant was taken for protein immobilization after buffer exchanged using pH 9.0 buffer (sodium citrate and sodium carbonate) using desalting column (Zeba; Life Technologies). Protein concentration was measured by a BCA assay.

**Fluorescence microscopy:** The immunofluorescence method was performed using the LAPC4 wildtype or the Androgen resistant LAPC4-AI prostate cancer cells were grown on poly-d-lysine coated glass coverslips were fixed with 2% paraformaldehyde-PBS for 10 min. After fixation, cells were washed three times with PBS, followed by permeabilization in 0.1% Triton X-100 for 5 min or without permebalization for AAL lectin staining. The fixed were blocked in 1% bovine serum albumin in PBS for 15 min, followed by a 1-h incubation with the primary antibodies against AR or with the biotinylated AAL lectin. After washing three times with TPBS, cells were then probed with secondary antibodies ( rhodamine-conjugated anti-rabbit IgG or Streptavidin anti-rabbit IgG), and the DNA was stained with DAPI for 10 min. Images were taken on Zeiss Axioobserver 40X fluorescence microscope and with LSM 710 confocal microscopy (Carl Zeiss, Jena, Germany). Figures were constructed using Adobe Photoshop (Adobe Systems, CA).

**Xenograft animal models:** LAPC4 wildtype cells at a density of 1x 10^6^ in PBS were mixed in 1:1 ratio with 1x Matrigel (BD biosciences) and implanted into the dorsal flanks of Athymic nude male mice. Once tumors were established and reached to tumor volume of ~1cm^3^, animals were divided into two groups by total tumor volume with five animals per group. Since no data were available to evaluate the effect of castration on the LAPC4 xenografts as well as the dichotomous outcome of the FUT8 staining between the groups, we choose to have five animals per group to get enough power (76.6%) that can differentiate 1.5 standard deviation assuming equal variability among each group. Castration was performed in one group of animals by dissecting away the testis through the 8mm to 10 mm midline skin incision along the scortal sac. Mice in control group were shame-operated under anesthetized to localize sex organs but without orchiectomy.. Animal studies were carried out in compliance with the U.S. Public Health Service Policy as approved by the Institutional Animal Care and Use Committee. Animals were monitored every other day for any signs of cachexia and distress. No animals were excluded from the analysis. Investigators involved in animal experiment were not blinded to the treatment.

At the end of the experiment (5 weeks after castration) all animals were sacrificed and tumor were removed. Two tumors from each group were used for immunohistochemisty using the FUT8 antibody (Minneapolis, MN).

**Statistical analysis:** All experiments were done in triplicate or quadruplicate and plotted with s.e.m. All statistical analysis was performed using Excel running on IBM-PC compatible computer on the Windows 7-operating system. Statistical comparisons for the in vitro data were analyzed by two sided Student’s t-test, similar variance between the groups of data were calculated by squaring the S.D of the sample sets. Using Excel, F-statistics were than used to estimate equal variance between samples. Statistical significance was defined as P<0.05.
