# Supplementary material for: Overexpression of α (1,6) fucosyltransferase in the development of castration-resistant prostate cancer cells
Source: Prostate Cancer Prostatic Dis. 2018 Jan 16;21(1):137–46. doi: 10.1038/s41391-017-0016-7 (PMC5895601; doi:10.1038/s41391-017-0016-7)
Supplement: Supplementary file 3 — Supplemental Table 1 [file 41391_2017_16_MOESM3_ESM.pdf]

Accession	Description	# Peptides	# PSMs	Biological Process	115/114	116/114	117/114
6648060	cold-inducible RNA-binding protein isoform 2 [Homo sapiens]	2	8	cell organization and biogenesis	1.013	16.020	11.638
5303936	PREDICTED: actin-binding LIM protein 1 isoform X15 [Homo sapie	3	9	; regulation of biological process	0.892	15.387	12.645
1196134	hCG17415, isoform CRA_b, partial [Homo sapiens]	2	4		0.889	14.952	13.029
1195703	PDZ and LIM domain 1 (elfin), isoform CRA_c [Homo sapiens]	2	9	; regulation of biological process	0.907	14.758	27.020
4104443	LIM and SH3 domain protein 1 isoform b [Homo sapiens]	2	8	; regulation of biological process	0.934	10.701	9.226
2913274	oxysterol-binding protein-related protein 10 isoform 2 [Homo sapie	3	6	metabolic process; transport	0.991	10.660	9.344
3180786	ZNF326 protein, partial [Homo sapiens]	5	12	; regulation of biological process	0.928	8.596	6.759
1802737	unknown [Homo sapiens]	2	3	al process; response to stimulus	0.798	8.432	6.791
1162832	CALD1 protein, partial [Homo sapiens]	2	4	cellular component movement	0.904	8.316	6.394
1195894	complement component 3, isoform CRA_a [Homo sapiens]	2	4	esponse to stimulus; coagulation	0.973	8.227	8.099
1404241	unnamed protein product [Homo sapiens]	2	4	cell organization and biogenesis	1.034	8.040	6.150
1195949	RNA binding motif protein 14, isoform CRA_a [Homo sapiens]	16	86	cell organization and biogenesis	0.967	8.024	7.159
1196183	ataxin 2, isoform CRA_b [Homo sapiens]	4	7	ization and biogenesis; transport	0.856	7.980	5.128
1702836	Similar to gelsolin (amyloidosis, Finnish type), partial [Homo sapie	2	4	e to stimulus; metabolic process	0.882	7.466	6.361
224053	macroglobulin alpha2	4	9		0.855	7.295	7.380
1582561	unnamed protein product [Homo sapiens]	7	15	gical process; metabolic process	0.923	7.284	6.612
7679088	PREDICTED: regulation of nuclear pre-mRNA domain-containing p	4	8		0.813	7.088	6.539
28590	unnamed protein product [Homo sapiens]	5	27	; regulation of biological process	0.995	6.664	6.852
7680162	PREDICTED: protein LSM14 homolog B isoform X11 [Homo sapien	2	5	biological process; development	0.792	6.662	5.836
1196125	N-myc downstream regulated gene 1, isoform CRA_a [Homo sapie	4	26	cell organization and biogenesis	0.890	6.655	5.029
5747213	NFkB interacting protein 1 [Homo sapiens]	4	11	ss; metabolic process; cell death	0.920	6.583	5.280
8645108	UDP-glucuronosyltransferase 1 family polypeptide A8s, partial [Ho	2	5	; regulation of biological process	0.857	6.582	5.680
8280276	RBM1, partial [Homo sapiens]	7	49	metabolic process	1.029	6.442	6.509
1196009	potassium channel tetramerisation domain containing 12 [Homo s	5	7	cell organization and biogenesis	1.053	6.368	6.202
1568012	Similar to breast cancer anti-estrogen resistance 1, partial [Homo s	2	3	mponent movement; cell division	1.051	6.291	5.531
2175539	unnamed protein product [Homo sapiens]	9	65	biogenesis; response to stimulus	0.987	6.227	6.416
1196260	albumin, isoform CRA_k [Homo sapiens]	5	42	; regulation of biological process	0.869	6.186	7.135
1196051	splicing factor, arginine/serine-rich 14, isoform CRA_b [Homo sapi	3	7	metabolic process	0.844	6.156	5.600
7020475	unnamed protein product, partial [Homo sapiens]	2	4	metabolic process	0.917	5.832	4.671
1453865	phostensin [Homo sapiens]	2	3		0.831	5.781	4.966
3475826	charged multivesicular body protein 2b isoform 2 [Homo sapiens]	2	3	cal process; cellular homeostasis	0.814	5.552	4.692
1578298	Chain A, Crystal Structure Of Human Annexin I At 2.5 Angstroms R	4	18		0.941	5.551	6.560
4519940	nuclear receptor co-repressor, partial [Homo sapiens]	3	5		0.910	5.160	3.835

4884368	hypothetical protein, partial [Homo sapiens]	2	5		1.100	5.060	4.691
1092552	centrosomal protein of 170 kDa isoform gamma [Homo sapiens]	4	7		0.983	4.949	4.706
8273432	Chain A, Crystal Structure Of Cerebral Cavernous Malformation 2 C	2	3		0.764	4.844	4.220
2210413	unnamed protein product [Homo sapiens]	7	18	biogenesis; response to stimulus	0.878	4.831	4.336
7680047	PREDICTED: cytokine receptor-like factor 1 isoform X3 [Homo sap	2	8	regulation of biological process	1.438	4.789	5.690
1195733	ubiquilin 4, isoform CRA_a [Homo sapiens]	4	28	; regulation of biological process	0.984	4.683	3.888
8655681	hypothetical protein, partial [Homo sapiens]	2	3	cell organization and biogenesis	0.942	4.676	4.246
7679722	PREDICTED: pregnancy zone protein isoform X4 [Homo sapiens]	2	4	regulation of biological process	0.894	4.668	4.587
1195734	KIAA0907, isoform CRA_b [Homo sapiens]	2	4		0.826	4.657	4.120
1479027	RNA-binding protein MEX3A [Homo sapiens]	2	2		0.799	4.647	4.661
1196312	myosin IB, isoform CRA_d [Homo sapiens]	2	5	s; cellular component movement	0.920	4.613	4.553
7491970	Chain B, Structure Of Paxx	2	6		1.052	4.575	4.166
6330051	KIAA1150 protein, partial [Homo sapiens]	6	17	cell organization and biogenesis	0.837	4.556	3.628
1943870	unnamed protein product [Homo sapiens]	2	3	metabolic process	0.905	4.467	3.699
3165722	PREX1 protein, partial [Homo sapiens]	2	5	n; cellular component movement	0.887	4.449	4.321
1943823	unnamed protein product [Homo sapiens]	2	3	cell death	0.869	4.424	3.486
1196088	euchromatic histone-lysine N-methyltransferase 1, isoform CRA_b	3	7	cell organization and biogenesis	0.833	4.321	3.752
5667557	unnamed protein product, partial [Homo sapiens]	3	10		1.041	4.224	3.865
1943745	unnamed protein product [Homo sapiens]	6	25	al process; response to stimulus	0.785	4.216	3.324
3387189	C9orf75 protein, partial [Homo sapiens]	2	3		0.763	4.213	3.552
7679743	PREDICTED: CCR4-NOT transcription complex subunit 2 isoform X	2	4	bolic process; cell differentiation	0.912	4.194	3.526
3906086	baculoviral IAP repeat-containing protein 2 isoform 2 [Homo sapie	2	3	nd biogenesis; defense response	0.914	4.176	3.946
3866428	membrane-associated tyrosine- and threonine-specific cdc2-inhibi	2	3	cell organization and biogenesis	1.066	4.167	3.594
4550347	unnamed protein product [Homo sapiens]	2	3		0.761	4.160	3.540
1195903	exocyst complex component 8 [Homo sapiens]	4	11	sis; transport; metabolic process	0.811	4.150	3.638
1195830	ubiquilin 1, isoform CRA_d [Homo sapiens]	5	30	to stimulus; cell communication	0.932	4.072	3.364
1043701	unnamed protein product [Homo sapiens]	2	3		1.071	4.014	3.567
6836882	MHC class I antigen, partial [Homo sapiens]	3	5	e response; response to stimulus	1.124	3.943	3.687
5788261	PREDICTED: sphingosine-1-phosphate phosphatase 1 isoform X1	2	3	al process; response to stimulus	0.846	3.929	3.644
1086388	U4/U6.U5 tri-snRNP-associated protein 1 [Homo sapiens]	9	24	al process; response to stimulus	0.957	3.924	3.599
6220248	CIP29 protein [Homo sapiens]	3	11	n of biological process; transport	0.822	3.919	3.792
1195969	bin3, bicoid-interacting 3, homolog (Drosophila), isoform CRA_b [H	4	10	gical process; metabolic process	1.011	3.872	3.802
4004698	unnamed protein product [Homo sapiens]	3	12		1.079	3.843	3.514
1196048	A kinase (PRKA) anchor protein 8 [Homo sapiens]	2	4	al process; response to stimulus	0.853	3.836	3.425
1937884	unnamed protein product [Homo sapiens]	3	7	cell organization and biogenesis	0.874	3.833	3.338
2210396	unnamed protein product [Homo sapiens]	2	3	gical process; cell differentiation	0.870	3.773	3.472
1943787	unnamed protein product [Homo sapiens]	2	10	ent movement; defense response	0.947	3.759	4.019
1943825	unnamed protein product [Homo sapiens]	2	3	metabolic process	0.787	3.650	3.694
1943763	unnamed protein product [Homo sapiens]	3	8		1.140	3.641	3.611
1943753	unnamed protein product, partial [Homo sapiens]	2	2	of biological process; cell death	1.119	3.593	3.476

1196083	argininosuccinate synthetase, isoform CRA_c [Homo sapiens]	5	10	; regulation of biological process	0.990	3.588	2.910
5262627	hypothetical protein, partial [Homo sapiens]	9	20	to stimulus; cell communication	0.936	3.568	3.363
1196318	plakophilin 4, isoform CRA_g [Homo sapiens]	11	25	; regulation of biological process	0.942	3.566	3.402
1943739	unnamed protein product [Homo sapiens]	6	20	metabolic process	0.923	3.558	3.299
1195877	myeloid/lymphoid or mixed-lineage leukemia (trithorax homolog, D	3	4	zation and biogenesis; cell death	0.823	3.542	3.068
5803165	protein transport protein Sec61 subunit beta [Homo sapiens]	3	15	al process; response to stimulus	1.134	3.541	2.677
1196184	TRAF-type zinc finger domain containing 1, isoform CRA_a [Homo	3	7	; regulation of biological process	0.949	3.540	3.465
1704637	SON DNA binding protein isoform A [Homo sapiens]	7	35	; regulation of biological process	1.038	3.492	2.760
2019848	182kDa tankyrase1-binding protein [Homo sapiens]	7	17	cell organization and biogenesis	0.898	3.451	3.218
1943865	unnamed protein product, partial [Homo sapiens]	4	10	nd biogenesis; cell differentiation	0.807	3.444	3.005
1195950	RAD9 homolog A (S. pombe), isoform CRA_a [Homo sapiens]	2	3	; regulation of biological process	0.966	3.443	3.311
1195949	copper chaperone for superoxide dismutase, isoform CRA_b [Hom	2	5	; regulation of biological process	1.018	3.419	3.285
5788121	PREDICTED: cell division cycle 5-like protein isoform X1 [Homo sa	3	10	; regulation of biological process	1.117	3.409	3.092
3478355	SRA1 protein, partial [Homo sapiens]	2	5	l proliferation; cell differentiation	0.916	3.402	3.264
7679990	PREDICTED: lisH domain and HEAT repeat-containing protein KIA	3	6		0.959	3.359	3.726
1196013	hCG2028654 [Homo sapiens]	2	4	metabolic process	0.854	3.332	3.139
1943809	unnamed protein product [Homo sapiens]	5	35	of biological process; cell growth	0.984	3.264	3.440
1542655	ADD1 protein [Homo sapiens]	3	9	; response to stimulus; transport	1.186	3.249	3.290
1196297	chromosome 21 open reading frame 70, isoform CRA_f, partial [Ho	2	4		0.985	3.246	3.032
2210394	unnamed protein product [Homo sapiens]	9	23	al process; response to stimulus	0.886	3.241	3.291
1185726	serine/arginine repetitive matrix protein 2 [Homo sapiens]	20	82	metabolic process	0.901	3.238	2.639
1195698	TruB pseudouridine (psi) synthase homolog 1 (E. coli) [Homo sapi	2	4	metabolic process	1.018	3.225	2.670
1195908	microtubule-associated protein 2, isoform CRA_d [Homo sapiens]	2	6	biogenesis; response to stimulus	0.847	3.223	3.204
2098747	PHRF1 protein, partial [Homo sapiens]	3	8	metabolic process	0.877	3.186	2.921
1195790	PC4 and SFRS1 interacting protein 1, isoform CRA_c [Homo sapie	5	12	al process; response to stimulus	0.854	3.183	2.973
1168125	putative RNA-binding protein Luc7-like 2 isoform 1 [Homo sapiens	5	29	cell organization and biogenesis	0.864	3.181	2.415
7679743	PREDICTED: protein phosphatase 1 regulatory subunit 12A isofor	5	12	al process; response to stimulus	0.869	3.143	2.942
1195942	glycine-N-acyltransferase-like 2, isoform CRA_a [Homo sapiens]	2	3	metabolic process	0.838	3.142	3.049
1196113	BAT2 domain containing 1, isoform CRA_a [Homo sapiens]	3	6	cell differentiation	0.846	3.121	2.865
5647487	myocyte enhancer factor 2D/deleted in azoospermia associated pr	4	11	; regulation of biological process	0.945	3.120	2.573
1229199	Chain X, Crystal Structure Of Human Alpha 1,6-fucosyltransferase,	8	18		0.949	3.041	3.047
8655623	hypothetical protein, partial [Homo sapiens]	2	3	; regulation of biological process	0.956	3.019	2.734
2895209	ataxin 3 variant ao, partial [Homo sapiens]	2	3	ical process; cell communication	0.867	3.008	2.423
7679810	PREDICTED: YLP motif-containing protein 1 isoform X2 [Homo sap	6	18	gical process; metabolic process	0.836	2.992	2.779
6673152	hemoglobin beta chain, partial [Homo sapiens]	2	6	cell organization and biogenesis	0.848	2.984	2.854
7679410	PREDICTED: nuclear pore complex protein Nup153 isoform X3 [Ho	4	7	al process; response to stimulus	0.808	2.980	2.931
1195977	dUTP pyrophosphatase, isoform CRA_c [Homo sapiens]	4	11	metabolic process	0.907	2.979	2.656
4767872	TST [Homo sapiens]	2	5	ess; cell differentiation; transport	1.058	2.957	2.983
7679437	PREDICTED: ralBP1-associated Eps domain-containing protein 1 i	2	3		0.884	2.948	2.281
1196241	ral guanine nucleotide dissociation stimulator-like 2, isoform CRA	2	6	al process; response to stimulus	0.819	2.940	2.502

1890553	unnamed protein product [Homo sapiens]	3	7	metabolic process	0.988	2.916	2.629
1890531	unnamed protein product [Homo sapiens]	4	34	sis; metabolic process; transport	1.077	2.906	3.290
5253170	MUC5AC, partial [Homo sapiens]	4	6	al process; response to stimulus	0.940	2.881	3.286
3315082	Ser/Arg-related nuclear matrix protein [Homo sapiens]	2	16	metabolic process; transport	0.898	2.874	3.530
1195778	interferon regulatory factor 2 binding protein 1 [Homo sapiens]	5	13	; regulation of biological process	0.897	2.868	2.008
7679081	PREDICTED: lysine-specific demethylase 5B isoform X2 [Homo sa	2	3	; regulation of biological process	0.977	2.859	2.372
1195882	hCG1990378, isoform CRA_a [Homo sapiens]	11	31	metabolic process	1.058	2.852	2.719
3124340	histone acetyltransferase KAT7 isoform 4 [Homo sapiens]	2	4	; regulation of biological process	0.863	2.849	2.227
1890551	unnamed protein product [Homo sapiens]	3	33	regulation of biological process	0.970	2.832	2.684
1195900	Williams-Beuren syndrome chromosome region 1, isoform CRA_a	4	21	biological process; reproduction	1.044	2.831	2.839
3964451	PUM2 protein, partial [Homo sapiens]	2	4	cell organization and biogenesis	0.777	2.830	2.450
1195931	emerin (Emery-Dreifuss muscular dystrophy), isoform CRA_a [Hom	6	46	erentiation; response to stimulus	0.844	2.822	2.531
4135008	unknown [Homo sapiens]	2	4	; regulation of biological process	0.901	2.800	2.442
1876097	truncated plakophilin-2 [Homo sapiens]	6	10	ical process; cell communication	0.872	2.764	2.503
1890550	unnamed protein product [Homo sapiens]	17	153	transport	1.071	2.749	2.992
1891682	KIAA1966 protein, partial [Homo sapiens]	2	2	cell organization and biogenesis	1.106	2.723	2.713
1245044	HLA-DQB1 protein, partial [Homo sapiens]	2	4	; regulation of biological process	0.748	2.713	2.705
1116002	ITGB4 protein [Homo sapiens]	9	19	n; cellular component movement	0.857	2.713	2.452
3453487	unnamed protein product, partial [Homo sapiens]	2	3	; regulation of biological process	0.923	2.712	2.068
6330129	KIAA1157 protein, partial [Homo sapiens]	3	14	metabolic process	0.918	2.707	2.375
3414762	AMP deaminase 2 isoform 2 [Homo sapiens]	5	9	metabolic process	0.905	2.704	2.476
1582607	unnamed protein product [Homo sapiens]	5	12	e response; response to stimulus	1.034	2.701	1.896
1943854	unnamed protein product [Homo sapiens]	4	16	sponse to stimulus; development	0.884	2.700	2.198
3032279	matrin-3 isoform a [Homo sapiens]	15	135	regulation of biological process	0.952	2.694	2.751
5304063	PREDICTED: transducin-like enhancer protein 3 isoform X14 [Hom	11	46	al process; response to stimulus	0.987	2.689	2.468
7679447	PREDICTED: zinc finger protein 316 isoform X1 [Homo sapiens]	3	6	; regulation of biological process	0.986	2.681	2.287
7680048	PREDICTED: scaffold attachment factor B2 isoform X1 [Homo sapi	10	17	; regulation of biological process	0.949	2.671	2.868
1195872	conserved nuclear protein NHN1, isoform CRA_d, partial [Homo sa	2	6		1.049	2.670	2.368
1943807	unnamed protein product [Homo sapiens]	3	10	cell organization and biogenesis	0.842	2.659	2.449
7332153	TSC22 domain family protein 4 isoform a [Homo sapiens]	2	4	sponse to stimulus; reproduction	0.861	2.620	2.393
2014967	EF-hand domain-containing protein D2 [Homo sapiens]	2	4		0.920	2.619	2.736
7680459	PREDICTED: TATA-binding protein-associated factor 2N isoform X	6	32	regulation of biological process	0.927	2.612	2.694
1943837	unnamed protein product [Homo sapiens]	4	8	; regulation of biological process	0.940	2.598	2.373
1195728	nitric oxide synthase interacting protein, isoform CRA_a [Homo sa	2	4	; regulation of biological process	0.856	2.595	2.515
5696711	Chain B, Structure Of Human Annexin A2 In The Presence Of Calci	11	63		0.991	2.578	2.781
1181378	Chain B, Structure Of Activated G-alpha-i1 Bound To A Nucleotide	5	15	al process; response to stimulus	0.984	2.572	2.239
1196298	DNA segment on chromosome 21 (unique) 2056 expressed sequen	4	10	metabolic process	0.746	2.569	2.410
5254588	hypothetical protein, partial [Homo sapiens]	4	13	of biological process; cell death	0.867	2.567	2.595
4097777	unnamed protein product, partial [Homo sapiens]	2	3		0.995	2.565	2.632
1937851	unnamed protein product [Homo sapiens]	2	3		1.202	2.564	2.680

1195795	DEAH (Asp-Glu-Ala-His) box polypeptide 38, isoform CRA_f [Homo	3	5	metabolic process; transport	0.820	2.561	2.122
1943829	unnamed protein product [Homo sapiens]	7	41	; regulation of biological process	0.900	2.552	2.468
1196187	similar to splicing factor, arginine/serine-rich 4, isoform CRA_e [Ho	3	9		0.953	2.535	1.993
1195862	CTAGE family, member 5, isoform CRA_h [Homo sapiens]	2	3	cell organization and biogenesis	0.854	2.530	2.278
4767841	dJ1104E15.4 [Homo sapiens]	2	7		0.845	2.518	2.204
7679655	PREDICTED: ribosomal RNA-processing protein 8 isoform X1 [Hor	3	4	nunication; response to stimulus	0.984	2.510	2.062
7679843	PREDICTED: myelin expression factor 2 isoform X5 [Homo sapiens	7	21	bolic process; cell differentiation	0.789	2.503	2.304
8874929	Chain c, Structure Of The Hcv Ires Bound To The Human Ribosom	3	45		1.669	2.490	2.491
1155287	SLFN5 protein [Homo sapiens]	5	10	cell differentiation	1.011	2.489	2.587
1960498	Chain D, Crystal Structure Of Human 3-Oxoacid Coa Transferase 1	6	22	metabolic process	1.364	2.479	2.495
3436485	hypothetical protein, partial [Homo sapiens]	2	3	nd biogenesis; defense response	0.889	2.473	2.204
1195804	target of myb1 (chicken), isoform CRA_d [Homo sapiens]	3	7	transport	0.868	2.466	2.505
1943787	unnamed protein product [Homo sapiens]	5	16		0.898	2.465	2.103
1195699	adducin 3 (gamma), isoform CRA_b [Homo sapiens]	5	13	response to stimulus; transport	1.089	2.463	2.297
1943873	unnamed protein product [Homo sapiens]	2	6	; regulation of biological process	0.945	2.458	2.677
5788058	PREDICTED: CLIP-associating protein 2 isoform X31 [Homo sapier	9	16	mponent movement; cell division	0.917	2.451	2.191
5788058	PREDICTED: CLIP-associating protein 2 isoform X22 [Homo sapier	9	16	mponent movement; cell division	0.858	2.451	2.191
7679404	PREDICTED: alanine--tRNA ligase, mitochondrial isoform X1 [Hom	11	28	cell organization and biogenesis	0.920	2.437	2.211
4097770	unnamed protein product, partial [Homo sapiens]	4	26		1.070	2.436	2.565
1943869	unnamed protein product [Homo sapiens]	2	6	; regulation of biological process	0.844	2.432	2.263
1196030	iduronidase, alpha-L-, isoform CRA_a [Homo sapiens]	2	3	d biogenesis; metabolic process	0.783	2.429	2.312
5304275	PREDICTED: heterogeneous nuclear ribonucleoprotein M isoform	25	196	metabolic process	0.925	2.413	1.967
1196099	chromobox homolog 8 (Pc class homolog, Drosophila) [Homo sap	2	7	biogenesis; response to stimulus	1.110	2.403	2.497
8158909	Golgi integral membrane protein 4 isoform 2 [Homo sapiens]	3	9	transport	0.874	2.382	2.097
7679123	PREDICTED: TAF5-like RNA polymerase II p300/CBP-associated fa	2	3	gical process; metabolic process	0.956	2.378	2.052
3387332	KHDRBS1 protein [Homo sapiens]	4	36	nse to stimulus; cell proliferation	0.952	2.377	2.286
4793914	YBX1 protein, partial [Homo sapiens]	5	53	gical process; metabolic process	0.907	2.362	1.869
3327036	KIAA0611 protein, partial [Homo sapiens]	3	5	cell organization and biogenesis	0.933	2.345	2.379
3135309	leucin rich neuronal protein [Homo sapiens]	4	7		1.010	2.343	2.077
1943861	unnamed protein product [Homo sapiens]	4	6	cell organization and biogenesis	0.826	2.341	2.452
1582559	unnamed protein product [Homo sapiens]	2	6	; regulation of biological process	0.984	2.326	2.467
1943891	unnamed protein product [Homo sapiens]	7	53	al process; response to stimulus	1.006	2.322	2.084
3851220	CLIP1 protein, partial [Homo sapiens]	2	4	; response to stimulus; transport	0.865	2.319	2.099
7679831	PREDICTED: ATP-dependent Clp protease ATP-binding subunit clp	2	10	; regulation of biological process	0.968	2.318	2.424
1943843	unnamed protein product [Homo sapiens]	13	41	al process; response to stimulus	0.925	2.306	2.287
1553026	PCNP protein [Homo sapiens]	3	6	metabolic process	0.880	2.293	1.707
3453348	unnamed protein product [Homo sapiens]	3	4	meostasis; response to stimulus	0.978	2.292	2.694
5303715	PREDICTED: leucine-rich repeat flightless-interacting protein 1 iso	4	9	e response; response to stimulus	0.991	2.288	2.043
1195722	exosome component 6 [Homo sapiens]	2	7	; regulation of biological process	0.874	2.287	2.074
1890655	unnamed protein product [Homo sapiens]	2	3	; regulation of biological process	1.053	2.282	2.323

1195836	hook homolog 3 (Drosophila), isoform CRA_a [Homo sapiens]	4	9	; regulation of biological process	0.998	2.267	2.089
1073264	unknown [Homo sapiens]	2	3		0.874	2.263	2.318
1655415	unnamed protein product [Homo sapiens]	2	3	regulation of biological process	0.903	2.257	2.016
1403579	unnamed protein product [Homo sapiens]	3	11		1.052	2.255	1.776
1195743	nudE nuclear distribution gene E homolog 1 (A. nidulans), isoform	5	9	liferation; transport; cell division	0.867	2.243	2.036
7680029	PREDICTED: transcriptional repressor p66-alpha isoform X2 [Hom	3	8	gical process; metabolic process	0.908	2.243	1.839
1196245	kinesin light chain 4, isoform CRA_c [Homo sapiens]	2	4	metabolic process	0.813	2.239	1.938
7679267	PREDICTED: lipoma-preferred partner isoform X5 [Homo sapiens]	3	11		1.042	2.237	2.259
1404256	unnamed protein product [Homo sapiens]	2	4	esponse to stimulus; cell growth	1.130	2.219	1.992
5303847	PREDICTED: heterogeneous nuclear ribonucleoproteins A2/B1 iso	17	478	ss; metabolic process; transport	0.973	2.217	2.100
1195889	hCG2043060, partial [Homo sapiens]	2	3	metabolic process	0.956	2.210	2.026
1195723	ataxin 2-like, isoform CRA_b [Homo sapiens]	5	12	cell organization and biogenesis	0.919	2.193	2.242
1937869	unnamed protein product [Homo sapiens]	2	4		1.071	2.191	2.360
4004321	unnamed protein product, partial [Homo sapiens]	6	30		1.170	2.184	1.983
1196227	plakophilin 3, isoform CRA_b [Homo sapiens]	19	108	cell organization and biogenesis	1.004	2.174	1.980
6208753	arginine/serine-rich splicing factor 6 variant, partial [Homo sapiens	9	53	ization and biogenesis; transport	0.910	2.174	1.930
1196238	HLA-B associated transcript 2, isoform CRA_d [Homo sapiens]	4	13		0.917	2.170	2.287
7679603	PREDICTED: partitioning defective 3 homolog isoform X18 [Homo	7	16	sponse to stimulus; cell division	0.877	2.167	1.973
3801417	WD repeat domain 18 [Homo sapiens]	6	19	development	1.080	2.160	2.059
1196053	drebrin 1, isoform CRA_c, partial [Homo sapiens]	5	35	logical process; cell proliferation	0.958	2.157	2.409
6728836	Chain B, Structure Of The Human Sjogren Larsson Syndrome Enzy	7	26		1.086	2.134	2.115
1196168	pleckstrin homology domain containing, family A member 5, isofor	4	6		0.971	2.134	2.151
3759461	alpha-methyl-acyl-CoA racemase IIAs [Homo sapiens]	5	26	metabolic process	0.907	2.130	2.005
1195950	phosphatidylinositol transfer protein, membrane-associated 1, iso	6	13	al process; response to stimulus	0.974	2.119	1.993
1195962	translocase of outer mitochondrial membrane 34, isoform CRA_a [	3	12	transport	1.064	2.117	2.162
1937860	unnamed protein product [Homo sapiens]	2	5	ic process; response to stimulus	1.065	2.104	2.111
1043487	unnamed protein product [Homo sapiens]	2	4	; regulation of biological process	0.905	2.099	1.924
1195855	ribosomal protein L29, isoform CRA_a [Homo sapiens]	2	5	ization and biogenesis; transport	0.839	2.097	2.061
1592943	MAP4 protein [Homo sapiens]	6	19	mponent movement; cell division	1.046	2.096	1.590
1195691	pleckstrin homology domain interacting protein, isoform CRA_a [H	10	20	biogenesis; response to stimulus	1.014	2.095	1.998
7679259	PREDICTED: segment polarity protein dishevelled homolog DVL-3	3	12	velopment; response to stimulus	0.854	2.093	1.865
1890535	unnamed protein product [Homo sapiens]	2	5	cell organization and biogenesis	0.820	2.091	1.767
4995299	2-methylacyl-CoA racemase [Homo sapiens]	2	11	metabolic process	1.136	2.084	1.751
2210445	unnamed protein product [Homo sapiens]	4	8	transport	0.872	2.082	1.851
1196111	microsomal glutathione S-transferase 3, isoform CRA_b [Homo sa	4	38	al process; response to stimulus	1.027	2.078	1.848
7336061	transducin-like enhancer protein 1 isoform 3 [Homo sapiens]	8	27	sponse to stimulus; development	1.003	2.076	2.009
1195697	BCL2-associated athanogene 3, isoform CRA_a [Homo sapiens]	3	4	al process; response to stimulus	0.828	2.069	2.176
2540282	serine/threonine-protein kinase MARK2 isoform f [Homo sapiens]	6	12	al process; response to stimulus	0.800	2.065	1.807
1195809	SNAP25-interacting protein [Homo sapiens]	3	5	cell organization and biogenesis	0.900	2.058	2.195
2015115	Chain B, Crystal Structure Of The 53bp1 Brct Region Complexed T	2	3		1.030	2.055	1.900

1043426	unnamed protein product [Homo sapiens]	4	7	metabolic process	1.013	2.049	1.968
1943871	unnamed protein product [Homo sapiens]	3	7	; regulation of biological process	1.002	2.044	1.975
5304275	PREDICTED: heterogeneous nuclear ribonucleoprotein M isoform	26	199	metabolic process	0.925	2.036	1.799
1196065	methionine sulfoxide reductase B2, isoform CRA_b [Homo sapiens	2	4	d biogenesis; metabolic process	0.976	2.026	1.729
2746207	MSTP059 [Homo sapiens]	2	3	gical process; metabolic process	0.908	2.021	1.731
1937884	unnamed protein product [Homo sapiens]	2	2	; regulation of biological process	1.065	2.005	2.222
1196267	latrophilin 2, isoform CRA_e [Homo sapiens]	5	9	al process; response to stimulus	0.789	2.002	2.032
1568006	Stathmin 1/oncoprotein 18 [Homo sapiens]	4	23	al process; response to stimulus	1.080	2.002	1.987
7679255	PREDICTED: insulin-like growth factor 2 mRNA-binding protein 2 i	2	2	n of biological process; transport	1.071	1.999	2.054
1195898	hCG22695, isoform CRA_d [Homo sapiens]	2	11	transport	0.926	1.989	1.832
1195929	ribosomal protein S5, isoform CRA_b [Homo sapiens]	7	44	n of biological process; transport	1.058	1.985	1.560
1168126	protein FAM98C [Homo sapiens]	6	19		0.983	1.983	1.778
7311879	Chain A, Crystal Structure Of The Non-phosphorylated Human Alp	6	13		0.941	1.982	1.398
1582569	unnamed protein product [Homo sapiens]	2	4	al process; response to stimulus	0.854	1.980	1.632
1890692	unnamed protein product [Homo sapiens]	2	3	nd biogenesis; cell differentiation	1.176	1.979	1.729
3850200	AGC-like kinases, partial [Homo sapiens]	2	3		0.847	1.977	1.812
7679815	PREDICTED: zinc finger CCCH domain-containing protein 14 isofo	3	5	regulation of biological process	0.898	1.974	1.991
2143946	unnamed protein product [Homo sapiens]	2	10		0.878	1.974	2.012
1195705	multiple inositol polyphosphate histidine phosphatase, 1, isoform	3	7	metabolic process	1.024	1.974	1.700
1195752	integrin, alpha 2 (CD49B, alpha 2 subunit of VLA-2 receptor), isofo	6	8	nd biogenesis; cell differentiation	0.962	1.966	1.898
1196023	nudix (nucleoside diphosphate linked moiety X)-type motif 14, isof	2	5	metabolic process	1.143	1.963	1.517
1195943	vacuolar protein sorting 37C (yeast), isoform CRA_c, partial [Homo	2	6	d biogenesis; metabolic process	0.909	1.957	1.611
7018420	hypothetical protein [Homo sapiens]	2	2		0.836	1.953	1.743
5566953	RNA polymerase-associated protein LEO1 isoform 2 [Homo sapien	2	8	al process; response to stimulus	0.729	1.950	2.045
1196014	splicing factor, arginine/serine-rich 5, isoform CRA_e, partial [Hom	3	27	; regulation of biological process	0.860	1.948	1.739
7561430	Chain C, Crystal Structure Of Autophagic Snare Complex	2	5		0.776	1.947	1.787
1195945	similar to RIKEN cDNA 1810059G22, isoform CRA_a [Homo sapien	2	2		0.921	1.947	1.739
7020416	unnamed protein product [Homo sapiens]	4	15	; regulation of biological process	0.949	1.937	1.781
5788381	PREDICTED: peripheral plasma membrane protein CASK isoform X	4	6	cell organization and biogenesis	0.982	1.937	1.866
3333773	FWP010 [Homo sapiens]	3	15	; regulation of biological process	0.967	1.934	2.106
1943754	unnamed protein product [Homo sapiens]	6	28	metabolic process	1.184	1.927	1.881
1004727	KIAA1598 protein, partial [Homo sapiens]	2	4	s; cellular component movement	0.837	1.926	1.678
1195933	RNA terminal phosphate cyclase domain 1, isoform CRA_b [Homo	2	5	metabolic process	1.343	1.920	1.339
1943861	unnamed protein product [Homo sapiens]	14	156	; regulation of biological process	1.000	1.913	2.034
4097718	unnamed protein product, partial [Homo sapiens]	2	3		0.954	1.911	1.446
5788027	PREDICTED: DNA (cytosine-5)-methyltransferase 3A isoform X6 [H	2	4	us; cell differentiation; cell death	0.806	1.907	1.862
7679687	PREDICTED: cleavage and polyadenylation specificity factor subu	8	42	cell organization and biogenesis	0.865	1.905	1.704
6208746	SAM domain- and HD domain-containing protein 1 variant, partial [	3	12	nd biogenesis; defense response	1.165	1.897	1.999
1327917	TRIOBP protein, partial [Homo sapiens]	2	2	f biological process; cell division	0.897	1.897	1.821
1196093	WNK lysine deficient protein kinase 1, isoform CRA_d [Homo sapi	3	13	al process; response to stimulus	0.813	1.892	1.872

1196229	tumor suppressing subtransferable candidate 4, isoform CRA_b [H	3	9		0.820	1.885	2.002
1943841	unnamed protein product [Homo sapiens]	2	2		0.930	1.885	1.697
4004049	unnamed protein product, partial [Homo sapiens]	5	49		1.150	1.881	1.979
4004183	unnamed protein product, partial [Homo sapiens]	3	22		0.960	1.877	1.821
1196186	splicing factor, arginine/serine-rich 9 [Homo sapiens]	6	44	; regulation of biological process	1.013	1.876	1.915
2397816	Chain A, The Crystal Structure Of The Reduced Form Of Human Ss	4	8	metabolic process	1.036	1.870	2.129
1195817	sideroflexin 1, isoform CRA_a [Homo sapiens]	11	91	transport; cell differentiation	1.085	1.869	1.759
1195920	hCG1748748 [Homo sapiens]	2	3		0.926	1.865	1.564
1890674	unnamed protein product [Homo sapiens]	12	133	cell organization and biogenesis	1.166	1.861	1.948
1196123	hCG2008737 [Homo sapiens]	4	37		0.985	1.851	1.707
1195791	hCG2011540, isoform CRA_b [Homo sapiens]	2	8	; regulation of biological process	0.817	1.850	1.832
8923920	core histone macro-H2A.2 [Homo sapiens]	7	23	cell organization and biogenesis	0.903	1.848	2.080
1196284	brain abundant, membrane attached signal protein 1, isoform CRA	2	5	nd biogenesis; cell differentiation	0.866	1.841	1.440
5587573	Golgi-associated plant pathogenesis-related protein 1 isoform b [H	2	3	regulation of biological process	0.983	1.840	1.513
7679093	PREDICTED: myocyte-specific enhancer factor 2D isoform X5 [Hor	4	9	; regulation of biological process	0.996	1.835	1.529
2210444	unnamed protein product [Homo sapiens]	2	8	metabolic process	0.951	1.835	1.765
2210452	unnamed protein product [Homo sapiens]	2	3	esponse to stimulus; coagulation	1.248	1.835	1.662
1943913	unnamed protein product [Homo sapiens]	5	22	metabolic process	0.959	1.834	1.669
9044183	PTGFRN protein, partial [Homo sapiens]	4	16	cell organization and biogenesis	1.034	1.833	1.792
1943750	unnamed protein product [Homo sapiens]	3	4	; regulation of biological process	0.989	1.831	1.710
1196022	eukaryotic translation initiation factor 5, isoform CRA_b [Homo sa	2	2	; regulation of biological process	1.154	1.831	1.810
1491651	dystrobrevin alpha isoform 8 [Homo sapiens]	5	7	to stimulus; cell communication	0.885	1.819	1.724
5303674	PREDICTED: rab11 family-interacting protein 5 isoform X3 [Homo s	4	8	al process; response to stimulus	0.848	1.818	2.055
7679386	PREDICTED: RUN and FYVE domain-containing protein 1 isoform	4	8	; regulation of biological process	0.828	1.818	1.759
1195919	nucleolar protein 9 [Homo sapiens]	2	2	metabolic process	1.183	1.816	1.628
2038559	placental protein KG-20 [Homo sapiens]	3	5	cell organization and biogenesis	1.171	1.814	1.892
1195833	acetyl-Coenzyme A acyltransferase 2 (mitochondrial 3-oxoacyl-Coe	3	5	; regulation of biological process	0.989	1.813	1.758
1196059	zinc finger protein 598, isoform CRA_e [Homo sapiens]	3	7		0.725	1.809	1.703
1195744	ATP-binding cassette, sub-family F (GCN20), member 2, isoform C	7	32	transport; metabolic process	0.824	1.808	1.746
1196043	zinc finger CCCH-type, antiviral 1, isoform CRA_b, partial [Homo s	5	10	gical process; defense response	0.826	1.798	1.656
4925657	Kinesin light chain 3 [Homo sapiens]	3	8	ent; transport; metabolic process	0.970	1.787	1.703
1196035	engulfment and cell motility 3, isoform CRA_b [Homo sapiens]	3	8	h; cellular component movement	0.974	1.784	1.812
1350932	ribosomal protein L27a [Homo sapiens]	4	23	ization and biogenesis; transport	1.123	1.783	1.659
2889658	Chain B, Human Sumo E1~sumo1-amp Tetrahedral Intermediate M	2	12	metabolic process	1.035	1.781	1.546
2210406	unnamed protein product [Homo sapiens]	2	6	gical process; metabolic process	0.896	1.779	1.771
9954649	XRCC1 DNA repair protein, partial [Homo sapiens]	3	8	ic process; response to stimulus	0.927	1.776	1.635
1943828	unnamed protein product [Homo sapiens]	11	54	; regulation of biological process	0.905	1.773	1.688
6730223	Chain D, Crystal Structure Of Human Profilin Ii	4	23	cell organization and biogenesis	0.978	1.766	1.781
2883834	BCLAF1 protein, partial [Homo sapiens]	2	5	al process; response to stimulus	0.907	1.762	1.456
7679304	PREDICTED: replication factor C subunit 1 isoform X2 [Homo sapi	2	5	al process; cellular homeostasis	1.097	1.755	1.464

6476248	lys-63-specific deubiquitinase BRCC36 isoform 2 [Homo sapiens]	2	3	cell organization and biogenesis	0.983	1.755	1.274
1195947	splicing factor 1, isoform CRA_d [Homo sapiens]	6	28	; regulation of biological process	0.986	1.754	1.659
1195956	peroxisomal proliferator-activated receptor A interacting complex	4	6	; regulation of biological process	0.903	1.753	1.629
1196095	solute carrier family 39 (metal ion transporter), member 11, isoform	3	4	transport; cellular homeostasis	1.009	1.741	1.583
1043537	unnamed protein product, partial [Homo sapiens]	4	6	of biological process; cell death	0.880	1.739	1.565
1662951	uncharacterized protein C17orf85 [Homo sapiens]	2	3	e response; response to stimulus	0.807	1.738	1.728
1196277	cell division cycle associated 8, isoform CRA_a [Homo sapiens]	2	6	sponse to stimulus; cell division	0.928	1.737	1.582
4503977	starch-binding domain-containing protein 1 [Homo sapiens]	6	15	metabolic process	0.930	1.733	1.571
1312477	protein HGH1 homolog [Homo sapiens]	2	3		0.911	1.731	1.717
1195952	START domain containing 10, isoform CRA_c [Homo sapiens]	3	5	; regulation of biological process	1.366	1.724	1.841
7679733	PREDICTED: cysteine and glycine-rich protein 2 isoform X1 [Homo	2	14	development; cell differentiation	1.004	1.722	1.595
1195980	vacuolar protein sorting 13C (yeast), isoform CRA_h [Homo sapien	4	6	transport	0.869	1.716	1.695
1196012	spectrin repeat containing, nuclear envelope 2, isoform CRA_g [Ho	8	14	; regulation of biological process	0.930	1.716	1.591
2154073	unnamed protein product, partial [Homo sapiens]	3	15		0.946	1.714	1.512
1206599	Histone cluster 1, H1t [Homo sapiens]	8	85	n; cellular component movement	1.039	1.713	1.628
4097680	unnamed protein product, partial [Homo sapiens]	3	5		0.847	1.710	1.569
5419846	hypothetical protein, partial [Homo sapiens]	3	5	al process; response to stimulus	0.866	1.709	1.672
1196152	ciliary rootlet coiled-coil, rootletin, isoform CRA_a [Homo sapiens]	5	12	cell organization and biogenesis	0.962	1.709	1.530
1186009	SCRIB protein, partial [Homo sapiens]	5	9	zation and biogenesis; cell death	0.851	1.709	1.676
306549	homology to rat ribosomal protein L23, partial [Homo sapiens]	4	16	cess; transport; cell proliferation	1.145	1.707	1.664
2210440	unnamed protein product [Homo sapiens]	7	14	ss; metabolic process; transport	0.845	1.707	1.773
3440302	testis-expressed sequence 264 protein isoform 2 [Homo sapiens]	2	10		1.102	1.703	1.802
1943876	unnamed protein product [Homo sapiens]	4	14	cell organization and biogenesis	1.114	1.702	1.733
1890533	unnamed protein product [Homo sapiens]	5	11	d biogenesis; metabolic process	1.052	1.700	1.692
8923390	MICOS complex subunit MIC19 [Homo sapiens]	3	27	cell organization and biogenesis	1.075	1.699	1.617
1655065	unnamed protein product [Homo sapiens]	5	16	; regulation of biological process	1.094	1.698	1.591
1943749	unnamed protein product [Homo sapiens]	12	68	n of biological process; transport	0.864	1.696	1.518
6282248	unknown, partial [Homo sapiens]	3	7	cell organization and biogenesis	0.950	1.695	1.493
1196265	3-hydroxybutyrate dehydrogenase, type 2, isoform CRA_c [Homo s	2	3	bolic process; cell differentiation	0.774	1.693	1.535
7679448	PREDICTED: peptidyl-prolyl cis-trans isomerase FKBP9 isoform X1	2	2	metabolic process	1.077	1.692	1.789
1460295	FKBP8 protein [Homo sapiens]	5	9	al process; response to stimulus	1.133	1.689	1.506
1890655	unnamed protein product [Homo sapiens]	4	10	; regulation of biological process	0.963	1.688	1.388
7679770	PREDICTED: fibrosin-1-like protein isoform X19 [Homo sapiens]	2	3		0.829	1.686	1.331
7680541	PREDICTED: N(G),N(G)-dimethylarginine dimethylaminohydrolase	7	48	al process; response to stimulus	0.887	1.683	1.680
3263199	centrosomal protein of 170 kDa protein B isoform 2 [Homo sapiens	2	2		1.323	1.682	1.776
1196229	nucleosome assembly protein 1-like 4, isoform CRA_a [Homo sapi	3	18	cell organization and biogenesis	1.312	1.677	1.410
1195947	multiple endocrine neoplasia I, isoform CRA_c [Homo sapiens]	2	4	biogenesis; cellular homeostasis	1.173	1.674	1.643
1196024	similar to RIKEN cDNA C030006K11 gene, isoform CRA_b [Homo s	5	27		0.855	1.673	1.350
2210438	unnamed protein product [Homo sapiens]	7	25	metabolic process	0.972	1.673	1.642
1196294	phosphoglycerate dehydrogenase like 1, isoform CRA_c [Homo sa	3	8		0.862	1.673	1.452

3056775	Chain A, Ralb-Rlip76 (Ralbp1) Complex	2	3	al process; response to stimulus	0.791	1.669	1.493
1937871	unnamed protein product [Homo sapiens]	2	5	nunication; response to stimulus	0.761	1.664	1.602
5788093	PREDICTED: 60S ribosomal protein L34 isoform X1 [Homo sapiens	6	53	ization and biogenesis; transport	0.937	1.662	1.263
1043372	unnamed protein product [Homo sapiens]	2	3	ic process; response to stimulus	0.904	1.659	1.404
3212676	sialate O-acetylesterase isoform 2 [Homo sapiens]	2	5	gical process; metabolic process	0.767	1.656	1.672
1943847	unnamed protein product [Homo sapiens]	4	10	al process; response to stimulus	1.014	1.656	1.649
7679090	PREDICTED: hepatoma-derived growth factor isoform X2 [Homo sa	2	18	nse to stimulus; cell proliferation	0.887	1.655	1.572
7679123	PREDICTED: protein ELYS isoform X4 [Homo sapiens]	7	15	cell organization and biogenesis	0.905	1.651	1.889
2174861	FLJ00383 protein, partial [Homo sapiens]	6	18	; response to stimulus; transport	0.986	1.645	1.802
1943888	unnamed protein product [Homo sapiens]	3	4	nse to stimulus; cell proliferation	0.895	1.639	1.680
5303608	PREDICTED: protein 4.1 isoform X10 [Homo sapiens]	3	4	cell organization and biogenesis	0.671	1.638	1.604
5106572	transcriptional activator SRCAP [Homo sapiens]	4	6	cell organization and biogenesis	0.971	1.631	1.401
7680398	PREDICTED: filamin-A isoform X2 [Homo sapiens]	71	481	; coagulation; metabolic process	0.947	1.631	1.658
179389	BCR-ABL protein, partial [Homo sapiens]	2	2	cell organization and biogenesis	0.833	1.626	1.510
1195825	heterogeneous nuclear ribonucleoprotein A0 [Homo sapiens]	4	21	; regulation of biological process	0.898	1.626	1.818
7679144	PREDICTED: trifunctional enzyme subunit beta, mitochondrial isof	9	51	metabolic process	1.024	1.624	1.622
1943868	unnamed protein product, partial [Homo sapiens]	5	22	transport; metabolic process	0.832	1.623	1.658
1943869	unnamed protein product [Homo sapiens]	2	4	; metabolic process; cell division	0.881	1.619	2.078
1196095	G protein-coupled receptor, family C, group 5, member C, isoform	2	4	al process; response to stimulus	0.775	1.618	1.407
3171081	endophilin-A2 isoform 3 [Homo sapiens]	3	16	al process; response to stimulus	0.904	1.614	1.521
3475438	dihydropyrimidinase-related protein 2 isoform 3 [Homo sapiens]	3	7	s; cellular component movement	0.871	1.612	1.510
4929567	CGI-49 protein [Homo sapiens]	3	13	metabolic process	1.045	1.611	1.438
1943832	unnamed protein product [Homo sapiens]	2	2	; regulation of biological process	1.002	1.608	1.592
1890530	unnamed protein product [Homo sapiens]	14	50	ic process; response to stimulus	1.089	1.604	1.718
1943826	unnamed protein product [Homo sapiens]	3	17	gical process; metabolic process	0.895	1.603	1.514
5094939	hypothetical protein, partial [Homo sapiens]	2	4	metabolic process	0.874	1.602	1.463
3453245	unnamed protein product [Homo sapiens]	3	12		1.054	1.601	1.335
6005717	ATP synthase subunit e, mitochondrial [Homo sapiens]	3	16	metabolic process; transport	1.209	1.598	1.486
1195805	pyridoxal (pyridoxine, vitamin B6) phosphatase, isoform CRA_a [H	4	16	biogenesis; response to stimulus	0.918	1.597	1.493
1195996	zinc finger protein 9 (a cellular retroviral nucleic acid binding prote	3	13	; regulation of biological process	0.939	1.596	1.752
3478330	ZC3H7B protein, partial [Homo sapiens]	2	3		1.106	1.594	1.437
8375506	Chain C, Structure Of Human Decr Ternary Complex	9	40	metabolic process	0.975	1.592	1.563
1196297	collagen, type VI, alpha 1, isoform CRA_b, partial [Homo sapiens]	10	43	ic process; response to stimulus	0.865	1.589	1.435
5901666	hypothetical protein, partial [Homo sapiens]	4	20	cell organization and biogenesis	0.975	1.588	1.751
1890534	unnamed protein product [Homo sapiens]	12	76	metabolic process	1.065	1.588	1.507
1196098	splicing factor, arginine/serine-rich 2, isoform CRA_d [Homo sapie	2	5	ss; metabolic process; transport	0.830	1.586	1.400
9965297	protocadherin-beta8, partial [Homo sapiens]	3	6		1.285	1.585	1.677
1890694	unnamed protein product [Homo sapiens]	9	47	metabolic process	1.080	1.582	1.488
1890532	unnamed protein product [Homo sapiens]	2	5	n of biological process; transport	0.926	1.580	1.426
4814645	HIP-55 [Homo sapiens]	6	22	; regulation of biological process	0.858	1.579	1.391

1196274	hCG27868, isoform CRA_c [Homo sapiens]	2	5	; regulation of biological process	0.809	1.579	1.641
4004095	unnamed protein product, partial [Homo sapiens]	2	8		0.912	1.579	1.571
1196244	hCG2000808, partial [Homo sapiens]	2	2		0.797	1.579	1.456
7679487	PREDICTED: E3 ubiquitin-protein ligase Hakai isoform X1 [Homo s	2	3	gical process; metabolic process	0.869	1.576	1.457
1195871	ribosomal protein L13, isoform CRA_a [Homo sapiens]	7	85	ization and biogenesis; transport	1.052	1.575	1.522
1937836	unnamed protein product [Homo sapiens]	2	6	; regulation of biological process	0.984	1.575	1.405
8392160	putative sodium-coupled neutral amino acid transporter 10 isoforn	4	6	transport	0.847	1.575	1.333
2265303	protein-methionine sulfoxide oxidase MICAL1 isoform 2 [Homo sa	2	8	e to stimulus; metabolic process	0.897	1.574	1.473
1943803	unnamed protein product [Homo sapiens]	9	34	al process; response to stimulus	1.039	1.574	1.665
4497171	death inducer-obliterator-3 [Homo sapiens]	10	29	al process; response to stimulus	0.874	1.573	1.394
1195721	spen homolog, transcriptional regulator (Drosophila) [Homo sapie	4	6	ic process; response to stimulus	0.809	1.572	1.437
5147618	hypothetical protein [Homo sapiens]	7	12	regulation of biological process	0.938	1.571	1.547
1943795	unnamed protein product [Homo sapiens]	8	28	; regulation of biological process	0.932	1.567	1.484
4004687	unnamed protein product [Homo sapiens]	2	8		1.072	1.566	1.440
1651900	syntaxin-16D [Homo sapiens]	2	3	cell organization and biogenesis	0.804	1.566	1.555
1196031	hypothetical protein FLJ20718, isoform CRA_c, partial [Homo sapi	3	9		1.017	1.565	1.344
2210429	unnamed protein product [Homo sapiens]	4	12	e to stimulus; metabolic process	0.956	1.565	1.453
7679851	PREDICTED: tumor suppressor p53-binding protein 1 isoform X2 [	6	15	; regulation of biological process	0.796	1.562	1.553
7679626	PREDICTED: ankyrin-3 isoform X26 [Homo sapiens]	12	27	component movement; transport	0.865	1.561	1.441
1890546	unnamed protein product [Homo sapiens]	7	23	metabolic process	0.895	1.553	1.459
7679758	PREDICTED: transmembrane protein 263 isoform X1 [Homo sapier	3	8		1.026	1.552	1.402
7680165	PREDICTED: RNA-binding protein Raly isoform X1 [Homo sapiens]	12	73	metabolic process	0.986	1.550	1.450
1195958	protein phosphatase 1, regulatory subunit 3D [Homo sapiens]	2	4	; regulation of biological process	0.961	1.549	1.682
3399117	NAPRT1 protein [Homo sapiens]	6	30	ic process; response to stimulus	1.033	1.548	1.393
1012968	PAXNEB protein, partial [Homo sapiens]	2	6	gical process; metabolic process	1.093	1.548	1.698
1890538	unnamed protein product [Homo sapiens]	6	23	d biogenesis; metabolic process	1.090	1.546	1.306
1195719	hCG30303 [Homo sapiens]	2	4		1.039	1.544	1.616
3165710	DDB1- and CUL4-associated factor 11 isoform 2 [Homo sapiens]	3	10	metabolic process	0.765	1.543	1.401
5304151	PREDICTED: transcription activator BRG1 isoform X4 [Homo sapie	9	22	d biogenesis; metabolic process	0.993	1.541	1.400
1195863	sorting nexin 6, isoform CRA_a [Homo sapiens]	2	4	cell organization and biogenesis	1.076	1.540	1.497
1196178	ATPase, Ca++ transporting, plasma membrane 1, isoform CRA_b [	11	36	; coagulation; metabolic process	1.006	1.539	1.500
2612783	cell growth regulator with EF hand domain protein 1 isoform a pre	3	4	; regulation of biological process	0.747	1.538	1.463
1937850	unnamed protein product [Homo sapiens]	2	3	bolic process; cell differentiation	1.069	1.537	1.647
1196013	gephyrin, isoform CRA_c [Homo sapiens]	2	11	cell organization and biogenesis	1.099	1.535	1.261
1582600	unnamed protein product [Homo sapiens]	2	9	metabolic process; transport	0.888	1.535	1.511
4712447	Serpin peptidase inhibitor, clade H (heat shock protein 47), membe	13	57	d biogenesis; metabolic process	1.004	1.534	1.632
1196088	ATPase, Class VI, type 11C, isoform CRA_b, partial [Homo sapiens	5	8	; regulation of biological process	1.016	1.533	1.444
1043821	unnamed protein product [Homo sapiens]	3	5	; regulation of biological process	0.781	1.533	1.597
1196262	heterogeneous nuclear ribonucleoprotein D (AU-rich element RNA	6	37	; regulation of biological process	0.872	1.532	1.507
3121764	uncharacterized protein KIAA1522 isoform 2 [Homo sapiens]	4	9	metabolic process	0.879	1.532	1.251

1507936	Processing of precursor 1, ribonuclease P/MRP subunit (S. cerevis	5	11	metabolic process	1.025	1.530	1.498
1519350	MRIP-1 [Homo sapiens]	6	17	al process; response to stimulus	0.923	1.527	1.505
1195884	tumor protein p53 inducible protein 11, isoform CRA_c [Homo sap	2	11	; regulation of biological process	1.013	1.524	1.378
1195959	VAMP (vesicle-associated membrane protein)-associated protein B	3	8	zation and biogenesis; cell death	0.960	1.524	1.434
7679622	PREDICTED: cytosolic purine 5'-nucleotidase isoform X8 [Homo sa	10	24	metabolic process	1.096	1.522	1.488
1943737	unnamed protein product [Homo sapiens]	3	6	transport	0.951	1.520	1.632
1195918	polymerase (RNA) III (DNA directed) polypeptide C (62kD), isoform	3	5	e response; response to stimulus	0.781	1.520	1.114
2210411	unnamed protein product [Homo sapiens]	3	10	nd biogenesis; defense response	1.043	1.520	1.360
6718844	RNA polymerase II subunit A C-terminal domain phosphatase isofo	7	14	f biological process; cell division	0.860	1.519	1.450
5454791	mediator of RNA polymerase II transcription subunit 30 isoform 2 [	4	6	ic process; response to stimulus	1.010	1.519	1.423
8819299	Chain B, Human Ppgalnact-2 Complexed With Manganese And Udp	5	14		1.028	1.519	1.424
5304157	PREDICTED: immunity-related GTPase family Q protein isoform X1	5	28		0.997	1.518	1.504
1195923	ATP binding domain 3 [Homo sapiens]	2	3	metabolic process	1.102	1.517	1.344
1195815	polypyrimidine tract binding protein 1, isoform CRA_g [Homo sapi	11	124	gical process; metabolic process	1.093	1.517	1.447
1195894	hCG2004001, isoform CRA_c [Homo sapiens]	11	19	; regulation of biological process	0.857	1.516	1.375
4767860	PES1 [Homo sapiens]	8	23	; regulation of biological process	0.988	1.516	1.340
1195918	peroxisomal biogenesis factor 11B, isoform CRA_a [Homo sapiens	6	16	al process; response to stimulus	1.017	1.515	1.369
1943888	unnamed protein product [Homo sapiens]	6	57	biogenesis; response to stimulus	0.983	1.514	1.473
1195793	mitochondrial ribosomal protein L50 [Homo sapiens]	2	12	d biogenesis; metabolic process	0.782	1.514	1.617
7679536	PREDICTED: epiplakin isoform X1 [Homo sapiens]	15	51		0.988	1.513	1.579
1196279	MARCKS-like 1, isoform CRA_b [Homo sapiens]	3	7	regulation of biological process	0.750	1.512	1.542
1195965	discs, large (Drosophila) homolog-associated protein 4, isoform C	2	4	cell communication	0.929	1.511	1.376
5687865	Chain C, Garp-snare Interaction	2	6		0.879	1.511	1.442
1943898	unnamed protein product [Homo sapiens]	3	18	o stimulus; cellular homeostasis	1.007	1.508	1.347
1280356	Transgelin 2 [Homo sapiens]	11	244	cell differentiation	0.949	1.507	1.305
1195857	adaptor protein containing pH domain, PTB domain and leucine zi	2	5	nse to stimulus; cell proliferation	0.932	1.507	1.420
1196001	ribosomal protein L24, isoform CRA_c [Homo sapiens]	4	23	s; cellular component movement	0.969	1.507	1.417
5303913	PREDICTED: endophilin-B2 isoform X6 [Homo sapiens]	4	16		1.112	1.507	1.431
2511438	calcium/calmodulin-dependent protein kinase II, partial [Homo sap	2	5	cell organization and biogenesis	0.992	1.505	1.452
1196278	splicing factor proline/glutamine-rich (polypyrimidine tract binding	13	120	cell organization and biogenesis	1.030	1.504	1.508
1196052	GEM interacting protein, isoform CRA_c, partial [Homo sapiens]	4	7	al process; response to stimulus	0.821	1.504	1.541
7679806	PREDICTED: ubiquinone biosynthesis monooxygenase COQ6 isofo	3	11	metabolic process	1.053	1.502	1.253
1200197	My016 protein [Homo sapiens]	2	7		0.990	1.492	1.523
6453465	hypothetical protein, partial [Homo sapiens]	2	3	e to stimulus; metabolic process	1.020	1.490	1.439
1195868	O-sialoglycoprotein endopeptidase, isoform CRA_a [Homo sapiens	2	14	metabolic process	0.789	1.490	1.432
1195857	pyruvate dehydrogenase (lipoamide) beta, isoform CRA_b [Homo s	2	4	; regulation of biological process	1.233	1.486	1.159
1195947	hCG2044837 [Homo sapiens]	2	3	al process; response to stimulus	0.845	1.484	1.349
7679683	PREDICTED: 39S ribosomal protein L16, mitochondrial isoform X1	3	6	cell organization and biogenesis	0.992	1.483	1.280
1527721	G9A [Homo sapiens]	2	3	cell organization and biogenesis	0.853	1.481	1.349
7679039	PREDICTED: hydroxymethylglutaryl-CoA synthase, mitochondrial i	3	8	ic process; response to stimulus	0.861	1.479	1.717

4707696	unnamed protein product [Homo sapiens]	5	15	; regulation of biological process	0.902	1.479	1.324
1943896	unnamed protein product [Homo sapiens]	5	13	al process; response to stimulus	0.818	1.479	1.528
7679677	PREDICTED: lysine-specific demethylase 2A isoform X5 [Homo sa	6	17	; regulation of biological process	1.012	1.479	1.529
7679613	PREDICTED: protein O-GlcNAcase isoform X2 [Homo sapiens]	2	6	response to stimulus; cell death	0.795	1.479	1.670
5445834	E3 ubiquitin-protein ligase UBR5 isoform 2 [Homo sapiens]	11	15	; regulation of biological process	0.999	1.474	1.889
7680123	PREDICTED: probable ATP-dependent RNA helicase DHX34 isofor	3	4	; regulation of biological process	0.882	1.471	1.217
5254556	hypothetical protein [Homo sapiens]	2	4		1.274	1.470	1.474
1196284	similar to mouse 2310016A09Rik gene, isoform CRA_a [Homo sapi	4	16	metabolic process	1.096	1.470	1.385
7679686	PREDICTED: syntaxin-5 isoform X3 [Homo sapiens]	3	5	cell organization and biogenesis	0.913	1.470	1.687
2098824	SLC27A3 protein [Homo sapiens]	9	26	metabolic process	0.976	1.468	1.361
2280064	Chromosome 7 open reading frame 27 [Homo sapiens]	2	4	h; cellular component movement	0.812	1.467	1.355
5274588	unnamed protein product [Homo sapiens]	23	54		0.957	1.467	1.294
1196079	RAB GTPase activating protein 1, isoform CRA_d [Homo sapiens]	5	12	regulation of biological process	0.929	1.466	1.308
9296541	KIAA0766 protein [Homo sapiens]	4	10		0.912	1.465	1.206
1195863	protein kinase D1, isoform CRA_a [Homo sapiens]	5	11	esis; cell proliferation; transport	1.062	1.463	1.513
1582557	unnamed protein product [Homo sapiens]	2	5	cell organization and biogenesis	1.015	1.462	1.219
1617312	UDP-GalNAc:polypeptide N-acetylgalactosaminyltransferase (GalN	4	8	metabolic process	1.113	1.461	1.416
1943850	unnamed protein product [Homo sapiens]	4	10		0.913	1.461	1.177
1943833	unnamed protein product, partial [Homo sapiens]	2	3	cell organization and biogenesis	0.797	1.459	1.464
1196218	hCG23783, isoform CRA_b [Homo sapiens]	20	298	ss; metabolic process; transport	1.219	1.458	1.375
1890655	unnamed protein product [Homo sapiens]	3	10	al process; response to stimulus	0.970	1.457	1.237
1195777	vasodilator-stimulated phosphoprotein, isoform CRA_b [Homo sap	2	7	al process; response to stimulus	0.799	1.455	1.405
1890535	unnamed protein product [Homo sapiens]	7	81	cell organization and biogenesis	0.917	1.455	1.565
5788211	PREDICTED: src substrate cortactin isoform X1 [Homo sapiens]	7	28	s; cellular component movement	0.822	1.454	1.503
7679263	PREDICTED: UBX domain-containing protein 7 isoform X1 [Homo s	5	20		0.864	1.453	1.393
1136285	UDP-GalNAc:polypeptide N-acetylgalactosaminyltransferase [Hom	3	5	metabolic process	0.763	1.453	1.285
1195760	lethal giant larvae homolog 1 (Drosophila), isoform CRA_c [Homo	3	10	gical process; cell differentiation	1.015	1.452	1.448
1195982	hCG2004593 [Homo sapiens]	3	13	metabolic process	1.116	1.449	1.672
1591639	Chain A, Solution Structure Of The Ring Domain Of The Synaptota	2	2		0.886	1.445	1.272
5304147	PREDICTED: protein Hook homolog 2 isoform X2 [Homo sapiens]	5	9	cell organization and biogenesis	0.892	1.442	1.360
1196083	nucleoporin 214kDa, isoform CRA_a [Homo sapiens]	6	15	al process; response to stimulus	0.943	1.437	1.307
1732073	TBP-associated factor, partial [Homo sapiens]	2	4	; regulation of biological process	0.809	1.436	1.318
1520130	TBC1 domain family, member 10A [Homo sapiens]	2	3	gical process; metabolic process	0.740	1.435	1.588
1870688	Bruton's tyrosine kinase-associated protein-135 [Homo sapiens]	8	40	ic process; response to stimulus	0.911	1.433	1.480
1890543	unnamed protein product [Homo sapiens]	3	3	; regulation of biological process	1.295	1.430	1.496
1834483	Chain P, Complex Between Pp2a-Specific Methylesterase Pme-1 A	5	8		1.066	1.430	1.421
7020768	unnamed protein product, partial [Homo sapiens]	7	32	; regulation of biological process	0.971	1.429	1.476
6252603	neurochondrin isoform 2 [Homo sapiens]	3	5	; regulation of biological process	1.064	1.428	1.420
7679126	PREDICTED: probable methyltransferase TARBP1 isoform X4 [Hor	3	7	gical process; metabolic process	0.899	1.428	1.434
7679036	PREDICTED: cytochrome P450 4X1 isoform X2 [Homo sapiens]	2	2	metabolic process	0.917	1.427	1.388

4916855	G3BP [Homo sapiens]	7	27	e to stimulus; metabolic process	1.243	1.427	1.213
9327947	Chain B, Crystal Structure Of Human Notch1 Ankyrin Repeats To 1	3	9		1.146	1.425	1.323
2465940	COG3 protein [Homo sapiens]	2	4	; regulation of biological process	1.115	1.423	1.382
1508044	DECR2 protein [Homo sapiens]	4	14	metabolic process	0.921	1.422	1.203
1680323	grancalcin [Homo sapiens]	2	8		0.798	1.421	1.386
1195812	hCG16955, isoform CRA_b [Homo sapiens]	2	6	; regulation of biological process	0.877	1.421	1.613
8209573	Chain A, Pp2ca	5	20		1.082	1.420	1.148
1582571	unnamed protein product [Homo sapiens]	13	98	ization and biogenesis; transport	1.044	1.420	1.244
1943886	unnamed protein product [Homo sapiens]	2	5	metabolic process	1.024	1.420	1.441
1196271	transmembrane protein 48, isoform CRA_a [Homo sapiens]	6	10	ization and biogenesis; transport	1.201	1.419	1.238
7679307	PREDICTED: exocyst complex component 1 isoform X7 [Homo sap	3	5	al process; response to stimulus	0.921	1.418	1.209
1196050	hypothetical protein BC013949, isoform CRA_a [Homo sapiens]	4	15	cell division	1.039	1.418	1.360
3359894	1-phosphatidylinositol 4,5-bisphosphate phosphodiesterase gamm	8	23	t; coagulation; defense response	1.059	1.418	1.258
1196096	mitochondrial ribosomal protein S7, isoform CRA_a [Homo sapien	2	7	d biogenesis; metabolic process	0.723	1.417	1.456
4604176	Chain A, Crystal Structure And Mechanism Of Activation Of Tbk1	3	7		0.994	1.417	1.201
1195878	hCG1732248, isoform CRA_b [Homo sapiens]	2	3		0.943	1.415	1.314
5303730	PREDICTED: ubiquitin-conjugating enzyme E2 E2 isoform X2 [Hom	3	18	; regulation of biological process	0.870	1.414	1.365
4098262	unnamed protein product, partial [Homo sapiens]	2	12		1.131	1.414	1.424
6208922	Etoposide-induced protein 2.4 variant, partial [Homo sapiens]	4	10	; regulation of biological process	0.829	1.413	1.422
4625572	Diacylglycerol lipase, beta [Homo sapiens]	3	6	ss; cell proliferation; coagulation	0.913	1.413	1.161
1890545	unnamed protein product [Homo sapiens]	2	14	d biogenesis; metabolic process	1.041	1.413	1.145
6208905	Inositol 1,4,5-trisphosphate receptor type 3 variant, partial [Homo s	5	9	cell organization and biogenesis	1.010	1.412	1.316
1195807	mitogen-activated protein kinase kinase kinase 7 interacting prote	2	3	bolic process; defense response	1.088	1.412	1.214
1890534	unnamed protein product [Homo sapiens]	9	175	of biological process; transport	1.110	1.411	1.447
1943817	unnamed protein product [Homo sapiens]	3	25	; response to stimulus; transport	1.595	1.409	1.673
6853311	KIF1A variant protein, partial [Homo sapiens]	4	6	ent; transport; metabolic process	0.839	1.408	1.333
1900164	Chain B, Snapshots Of The Rna Processing Factor Scaf8 Bound To	2	3		0.993	1.408	1.502
7679682	PREDICTED: nuclear mitotic apparatus protein 1 isoform X2 [Hom	53	309	; cell division; cell differentiation	1.004	1.407	1.326
1943859	unnamed protein product [Homo sapiens]	2	3	; transport; response to stimulus	0.833	1.407	1.484
1195806	GTP binding protein 1, isoform CRA_a [Homo sapiens]	2	4	; regulation of biological process	1.154	1.406	1.499
1196280	splicing factor, arginine/serine-rich 4, isoform CRA_c [Homo sapie	5	47	; regulation of biological process	0.842	1.405	1.337
1577399	nucleoside diphosphate-linked moiety X motif 19, mitochondrial pr	2	5	metabolic process	1.218	1.405	1.250
3165222	39S ribosomal protein L21, mitochondrial isoform d [Homo sapien	3	6	cell organization and biogenesis	1.041	1.405	1.353
1093378	aminopeptidase B, partial [Homo sapiens]	19	109	metabolic process	1.046	1.402	1.480
2276118	unnamed protein product [Homo sapiens]	3	12	nd biogenesis; defense response	0.894	1.402	1.263
1501081	JKTBP1delta6 [Homo sapiens]	4	16	; regulation of biological process	0.910	1.400	1.644
2752656	hypothetical protein, partial [Homo sapiens]	2	7	; regulation of biological process	0.902	1.400	1.441
7679629	PREDICTED: exosome complex component CSL4 isoform X1 [Hom	2	4	; regulation of biological process	1.259	1.400	1.430
2230294	mothers against decapentaplegic homolog 3 isoform 2 [Homo sap	3	7	nd biogenesis; cell differentiation	0.887	1.399	1.323
7680437	PREDICTED: protein LCHN isoform X1 [Homo sapiens]	2	6		0.977	1.399	1.232

1195825	SMAD, mothers against DPP homolog 5 (Drosophila), isoform CRA	3	4	se to stimulus; cell differentiation	0.983	1.398	1.228
1943767	unnamed protein product [Homo sapiens]	9	53	ic process; response to stimulus	0.983	1.397	1.454
4004668	unnamed protein product [Homo sapiens]	2	8		1.131	1.396	1.332
2819539	histone H2A type 2-B [Homo sapiens]	4	205	; regulation of biological process	0.938	1.395	1.417
5472355	eukaryotic translation initiation factor 3 subunit J isoform 3 [Homo	2	4	; regulation of biological process	1.006	1.394	1.213
563888	beta-centractin, partial [Homo sapiens]	4	9		1.058	1.394	1.356
2032825	Chain A, Crystal Structure Of Snx9px-Bar (230-595), C2221	3	8	cell communication	1.244	1.394	1.385
180985	CpG island protein [Homo sapiens]	2	4	metabolic process	0.948	1.394	1.378
5304105	PREDICTED: DNA topoisomerase 3-alpha isoform X1 [Homo sapie	4	7	cell organization and biogenesis	1.183	1.393	1.160
1195798	topoisomerase (DNA) III beta, isoform CRA_b [Homo sapiens]	2	5	metabolic process	1.183	1.393	1.160
3478504	RPS6 protein, partial [Homo sapiens]	9	66	se to stimulus; cell differentiation	1.159	1.392	1.359
1195918	leucine rich repeat containing 47, isoform CRA_a [Homo sapiens]	10	30	metabolic process	1.108	1.392	1.265
5911963	hypothetical protein, partial [Homo sapiens]	5	7	metabolic process	0.881	1.392	1.252
1706529	RecName: Full=ATP synthase mitochondrial F1 complex assembly	2	7	cell organization and biogenesis	0.892	1.391	1.313
1492425	Chain C, Crystal Structure Of The Human Sec23a24A HETERODIMI	9	148	transport	1.194	1.390	1.399
1943860	unnamed protein product [Homo sapiens]	3	6	; regulation of biological process	0.924	1.390	1.718
4614964	embryonal Fyn-associated substrate isoform 3 [Homo sapiens]	5	11	al process; response to stimulus	0.946	1.389	1.327
1195689	arginyl-tRNA synthetase-like, isoform CRA_e [Homo sapiens]	4	10	cell organization and biogenesis	0.773	1.389	1.265
1195700	nucleolar and coiled-body phosphoprotein 1, isoform CRA_b [Hom	2	7	cell organization and biogenesis	1.010	1.389	1.362
1943897	unnamed protein product [Homo sapiens]	4	20	cell organization and biogenesis	1.128	1.388	1.392
7680045	PREDICTED: far upstream element-binding protein 2 isoform X2 [H	16	107	n of biological process; transport	0.942	1.388	1.409
1943753	unnamed protein product [Homo sapiens]	3	4	metabolic process	1.050	1.387	1.220
1195714	unc-119 homolog (C. elegans), isoform CRA_b [Homo sapiens]	2	5	al process; response to stimulus	0.900	1.385	1.226
7679586	PREDICTED: far upstream element-binding protein 3 isoform X4 [H	12	56	; regulation of biological process	0.934	1.385	1.388
5788393	PREDICTED: secretory carrier-associated membrane protein 3 isof	4	36	transport; response to stimulus	1.048	1.384	1.236
1943807	unnamed protein product [Homo sapiens]	3	3	nd biogenesis; defense response	1.073	1.381	1.190
1229375	protein unc-119 homolog B [Homo sapiens]	2	8	cell organization and biogenesis	0.871	1.380	1.218
1195853	chromosome 3 open reading frame 60, isoform CRA_c [Homo sapi	3	8	cell organization and biogenesis	1.070	1.379	1.119
2646815	xaa-Pro aminopeptidase 1 isoform 2 [Homo sapiens]	7	17	metabolic process	0.967	1.379	1.430
5304148	PREDICTED: neurogenic locus notch homolog protein 3 isoform X	8	26	ic process; response to stimulus	1.084	1.379	1.416
1196072	aspartate beta-hydroxylase, isoform CRA_e, partial [Homo sapiens	6	18	ss; metabolic process; transport	0.828	1.379	1.485
7679342	PREDICTED: exocyst complex component 3 isoform X2 [Homo sap	6	15	d biogenesis; metabolic process	0.906	1.378	1.355
6973506	Chain b, Structure Of The Large Ribosomal Subunit From Human I	4	17		0.936	1.377	1.159
1196277	thyroid hormone receptor associated protein 3, isoform CRA_a [Ho	4	19	ic process; response to stimulus	0.958	1.374	1.417
1582587	unnamed protein product [Homo sapiens]	3	10	ization and biogenesis; transport	1.310	1.374	1.279
7679100	PREDICTED: retinoblastoma-binding protein 5 isoform X4 [Homo s	3	5	al process; response to stimulus	0.979	1.373	1.176
1327906	EPHB4 protein, partial [Homo sapiens]	5	9	al process; response to stimulus	0.889	1.373	1.407
1196072	carbonic anhydrase VIII, isoform CRA_a [Homo sapiens]	3	4	al process; response to stimulus	1.056	1.372	1.304
6010217	NOV/plexin-A1 protein, partial [Homo sapiens]	5	10	nt; cellular component movement	0.908	1.371	1.377
7581362	protein phosphatase 1B isoform 5 [Homo sapiens]	4	20	al process; response to stimulus	1.082	1.371	1.109

2033630	putative ATP-dependent RNA helicase DHX33 isoform 1 [Homo sap	3	5	; regulation of biological process	0.824	1.371	1.264
2226672	LIR-D1 precursor [Homo sapiens]	3	3	nent movement; cell proliferation	1.064	1.370	1.205
7679432	PREDICTED: AT-rich interactive domain-containing protein 1B isof	2	3	; regulation of biological process	0.955	1.370	1.299
7679554	PREDICTED: glyoxylate reductase/hydroxypyruvate reductase isof	2	25	cell organization and biogenesis	1.047	1.370	1.197
1639143	TPA: microtubule-associated protein [Homo sapiens]	3	5	sis; cell death; cell differentiation	0.928	1.368	1.215
5342860	Chain G, Crystal Structure Of Human Nucleosome Core Particle La	4	206		0.933	1.367	1.394
2842264	CROP protein, partial [Homo sapiens]	3	6	d biogenesis; metabolic process	1.058	1.367	1.291
183182	guanine nucleotide-binding regulatory protein alpha-inhibitory sub	6	20	ss; cell proliferation; cell division	1.069	1.366	1.404
1196045	dedicator of cytokinesis 6, isoform CRA_c, partial [Homo sapiens]	6	11	esponse to stimulus; coagulation	0.982	1.364	1.338
3930217	Nck-2 [Homo sapiens]	2	2	s; cellular component movement	0.848	1.364	1.321
1195988	mitochondrial ribosomal protein L47, isoform CRA_c [Homo sapie	2	5	cell organization and biogenesis	1.092	1.364	1.254
1937870	unnamed protein product [Homo sapiens]	3	7	metabolic process	1.021	1.364	1.232
2708305	U4/U6 small nuclear ribonucleoprotein hPrp4 [Homo sapiens]	5	10	metabolic process	1.168	1.363	1.235
1458436	SET, partial [Homo sapiens]	5	21	; regulation of biological process	1.885	1.363	1.668
7799760	mercaptopyruvate sulfurtransferase variant, partial [Homo sapiens	4	17	ic process; response to stimulus	1.132	1.361	1.356
1195713	similar to RIKEN cDNA 0610011N22 [Homo sapiens]	3	6		0.947	1.359	1.367
642532	ras-related small GTP binding protein Rab5 [Homo sapiens]	7	86	al process; response to stimulus	1.098	1.359	1.248
3312841	endophilin-B1 isoform 4 [Homo sapiens]	3	10	cell organization and biogenesis	0.926	1.359	1.382
1265483	Phosphoglucomutase 3 [Homo sapiens]	9	44	metabolic process	1.095	1.358	1.286
4842547	Chain D, Structure Of The Signal Recognition Particle Interacting W	5	11	ization and biogenesis; transport	0.965	1.357	1.359
7105222	FAM65A protein, partial [Homo sapiens]	3	6	al process; response to stimulus	0.952	1.356	1.249
1196207	splicing factor, arginine/serine-rich 7, 35kDa, isoform CRA_b [Hom	5	53	; regulation of biological process	0.948	1.355	1.203
7679810	PREDICTED: N6-adenosine-methyltransferase 70 kDa subunit isofo	2	8	; regulation of biological process	0.918	1.354	1.438
1196013	vesicle transport through interaction with t-SNAREs homolog 1B (	3	7	; regulation of biological process	0.805	1.354	1.238
1943845	unnamed protein product [Homo sapiens]	3	4		0.992	1.353	1.169
1943742	unnamed protein product [Homo sapiens]	8	70	; regulation of biological process	1.023	1.352	1.368
1196044	mitochondrial ribosomal protein L4, isoform CRA_b [Homo sapien	2	3	cell organization and biogenesis	0.848	1.352	1.327
5303816	PREDICTED: cap-specific mRNA (nucleoside-2'-O-)-methyltransfer	4	6	metabolic process	0.832	1.351	1.204
1196097	signal recognition particle 68kDa, isoform CRA_e [Homo sapiens]	12	30	; transport; response to stimulus	1.170	1.349	1.284
4097703	unnamed protein product, partial [Homo sapiens]	3	6		1.129	1.348	1.448
2033631	bcl-2-like protein 11 isoform 2 [Homo sapiens]	2	5	biogenesis; response to stimulus	1.028	1.348	1.241
3801461	C11orf2 protein, partial [Homo sapiens]	12	28	transport; metabolic process	0.927	1.347	1.181
1196275	EBNA1 binding protein 2, isoform CRA_a [Homo sapiens]	2	4		1.173	1.347	1.396
1937841	unnamed protein product [Homo sapiens]	2	3	e response; response to stimulus	1.035	1.347	1.182
6208721	calpastatin isoform a variant, partial [Homo sapiens]	6	9	gical process; cell differentiation	1.020	1.345	1.085
1195971	solute carrier family 25, member 13 (citrin), isoform CRA_b [Homo	16	61	; transport; response to stimulus	1.045	1.345	1.355
1404373	Sorting and assembly machinery component 50 homolog (S. cerev	8	20	d biogenesis; metabolic process	1.040	1.345	1.192
3421153	E6-AP ubiquitin-protein ligase, partial [Homo sapiens]	2	8	; regulation of biological process	0.932	1.344	1.241
1195878	H2A histone family, member X [Homo sapiens]	3	197	; regulation of biological process	0.938	1.342	1.384
5342864	Chain B, Crystal Structure Of Human Prpf4b Kinase Domain In Cor	2	6		0.890	1.342	1.288

1196025	fuse-binding protein-interacting repressor, isoform CRA_g [Homo	11	42	of biological process; cell death	1.105	1.341	1.382
5457199	Chain B, Crystal Structure Of The Trf2-binding Motif Of Slx4 In Con	2	7		0.991	1.341	1.299
1196252	carbonic reductase 4, isoform CRA_b [Homo sapiens]	2	3	cell organization and biogenesis	1.065	1.340	1.210
6573673	Chain A, Crystal Structure Of The Pten Tumor Suppressor	3	7	metabolic process	1.000	1.338	1.452
3925838	SRA stem-loop-interacting RNA-binding protein, mitochondrial iso	4	15	cell organization and biogenesis	0.909	1.338	1.294
1043674	unnamed protein product [Homo sapiens]	3	7	al process; response to stimulus	1.042	1.337	1.247
1788470	Chain A, Solution Structure Of The Sh2 Domain From Human Crk-I	2	8		0.899	1.336	1.129
3387497	SLC4A2 protein [Homo sapiens]	3	5	transport; cellular homeostasis	0.851	1.335	1.363
1119551	centrosomal protein of 131 kDa isoform b [Homo sapiens]	3	4	component movement; transport	0.947	1.335	1.478
6638568	septin-8 isoform f [Homo sapiens]	5	12		0.976	1.334	1.201
4405760	alternative protein SMCR7L [Homo sapiens]	2	6		1.069	1.333	1.338
2905599	Chain A, Crystal Structure Of Human Macrod1	3	19		1.097	1.332	1.206
1193315	PDZ domain protein 3' variant 4 [Homo sapiens]	2	4	al process; response to stimulus	0.790	1.332	1.269
416178	desmoglein 2 [Homo sapiens]	4	10	; regulation of biological process	1.026	1.331	1.218
1196219	inositol(myo)-1(or 4)-monophosphatase 2, isoform CRA_c, partial [	3	8	al process; response to stimulus	1.184	1.331	1.208
5463320	KIAA0216 splice variant 2, partial [Homo sapiens]	6	7	ent movement; cell differentiation	1.030	1.330	1.309
1195944	integrator complex subunit 5 [Homo sapiens]	3	6	metabolic process	0.885	1.330	1.432
1943774	unnamed protein product [Homo sapiens]	3	42	; regulation of biological process	0.898	1.329	1.316
7680182	PREDICTED: DNA topoisomerase 1 isoform X2 [Homo sapiens]	8	22	ell division; response to stimulus	0.924	1.328	1.300
1195918	Rho guanine exchange factor (GEF) 16, isoform CRA_b [Homo sap	8	19	s; cellular component movement	0.871	1.328	1.262
4004171	unnamed protein product, partial [Homo sapiens]	6	50		1.087	1.328	1.220
7678878	PREDICTED: rho guanine nucleotide exchange factor 5-like isoforr	6	13		0.878	1.327	1.341
1043713	unnamed protein product [Homo sapiens]	2	2	; regulation of biological process	1.069	1.327	1.478
7280557	Chain B, Metal Ion And Ligand Binding Of Integrin	4	9		0.829	1.326	1.176
1196077	fascin homolog 1, actin-bundling protein (Strongylocentrotus purp	16	82	s; cellular component movement	1.042	1.326	1.286
3249294	medulloblastoma antigen MU-MB-50.205, partial [Homo sapiens]	6	27	cell organization and biogenesis	0.902	1.325	1.216
5443462	ankyrin repeat and zinc finger domain-containing protein 1 isoforn	4	5	ic process; response to stimulus	0.933	1.325	1.289
1195754	hCG2040192 [Homo sapiens]	3	6		0.882	1.324	1.043
1487451	UNC84A protein [Homo sapiens]	4	7	cell organization and biogenesis	0.934	1.324	1.246
1162841	GTF3C4 protein, partial [Homo sapiens]	4	7	; regulation of biological process	0.973	1.323	1.195
5304218	PREDICTED: ribosomal biogenesis protein LAS1L isoform X6 [Hor	5	16	metabolic process	1.059	1.321	1.346
1943734	unnamed protein product [Homo sapiens]	2	3	biogenesis; response to stimulus	0.850	1.321	1.275
7680110	PREDICTED: BTB/POZ domain-containing protein KCTD15 isoform	2	2	cell organization and biogenesis	0.877	1.320	1.281
3109006	unnamed protein product [Homo sapiens]	2	6		1.035	1.320	1.249
1943781	unnamed protein product [Homo sapiens]	4	14	gical process; cell differentiation	0.969	1.320	1.322
1195692	sorting nexin 2, isoform CRA_a [Homo sapiens]	6	21	cell organization and biogenesis	0.855	1.319	1.161
7679050	PREDICTED: peflin isoform X4 [Homo sapiens]	4	16	ic process; response to stimulus	0.826	1.319	1.234
1196302	splicing factor, arginine/serine-rich 15, isoform CRA_a [Homo sapi	2	3		0.946	1.319	1.247
1196032	nudix (nucleoside diphosphate linked moiety X)-type motif 21, isof	4	16	cell organization and biogenesis	1.088	1.318	1.348
1169190	prolyl oligopeptidase [Homo sapiens]	7	15	metabolic process	1.069	1.318	1.327

5303804	PREDICTED: la-related protein 1 isoform X1 [Homo sapiens]	6	13	al process; response to stimulus	0.930	1.317	1.290
1196296	pericentrin (kendrin), isoform CRA_c [Homo sapiens]	2	5	logical process; cell proliferation	0.959	1.317	1.259
1195808	X-ray repair complementing defective repair in Chinese hamster ce	14	116	gical process; defense response	1.373	1.317	1.428
5303983	PREDICTED: cullin-5 isoform X1 [Homo sapiens]	5	22	; cellular homeostasis; transport	1.188	1.316	1.182
1196077	haloacid dehalogenase-like hydrolase domain containing 3, isofor	2	8	metabolic process	1.005	1.316	1.404
3387257	MTDH protein, partial [Homo sapiens]	2	4	cell organization and biogenesis	1.334	1.316	1.334
1793964	APEX nuclease (multifunctional DNA repair enzyme) 1 [Homo sapi	6	34	al process; cellular homeostasis	1.319	1.315	1.344
1943761	unnamed protein product [Homo sapiens]	3	4	cell organization and biogenesis	0.990	1.314	1.105
1582565	unnamed protein product [Homo sapiens]	6	13	metabolic process	0.950	1.314	1.181
1195821	Treacher Collins-Franceschetti syndrome 1, isoform CRA_f, partial	6	24	transport; metabolic process	0.832	1.313	1.350
1309760	Similar to ribosomal protein L23, partial [Homo sapiens]	4	30	ization and biogenesis; transport	0.956	1.313	1.232
1196115	ring finger protein 2, isoform CRA_a [Homo sapiens]	3	5	cell organization and biogenesis	1.086	1.313	1.276
1890331	L-3-phosphoserine phosphatase [Homo sapiens]	5	18	metabolic process	0.845	1.313	1.319
7561425	Chain x, Human Constitutive 20s Proteasome In Complex With Car	3	41		1.114	1.313	1.095
3441790	glycogen debranching enzyme, partial [Homo sapiens]	5	13	ic process; response to stimulus	0.968	1.312	1.618
2210435	unnamed protein product [Homo sapiens]	6	12	; regulation of biological process	0.880	1.312	1.157
1582557	unnamed protein product [Homo sapiens]	7	21	us; metabolic process; transport	0.906	1.312	1.298
7679105	PREDICTED: sorting nexin-27 isoform X5 [Homo sapiens]	5	15	al process; response to stimulus	0.911	1.310	1.273
5303908	PREDICTED: choline transporter-like protein 1 isoform X5 [Homo s	4	8	metabolic process; transport	0.910	1.310	1.179
6094122	Chain A, The Structure Of 1na In Complex With Src T338g	9	31		1.132	1.310	1.369
1196006	myosin ID, isoform CRA_e, partial [Homo sapiens]	13	36	n of biological process; transport	1.032	1.309	1.434
2210452	unnamed protein product [Homo sapiens]	4	16	cell organization and biogenesis	0.925	1.308	1.265
3387958	GTF3C5 protein, partial [Homo sapiens]	7	9	bolic process; cell differentiation	1.004	1.307	1.208
1195862	GTPase activating Rap/RanGAP domain-like 1, isoform CRA_a [Ho	3	7	regulation of biological process	0.846	1.307	1.322
4091074	Chain D, Complex Structure 6 Of DaxxH3.3(SUB7)H4	6	285		1.047	1.307	1.257
5303674	PREDICTED: echinoderm microtubule-associated protein-like 4 iso	8	22	cell organization and biogenesis	1.080	1.306	1.332
1002923	coronin-like protein [Homo sapiens]	4	14	se to stimulus; defense response	1.036	1.305	1.191
5655362	Chain B, Glycogen Synthase Kinase-3 Beta In Complex With 3-Indo	7	20	metabolic process	0.947	1.305	1.199
3118933	copine-1 isoform c [Homo sapiens]	9	28	esponse to stimulus; cell growth	1.076	1.305	1.305
1937859	unnamed protein product [Homo sapiens]	19	71	cell organization and biogenesis	0.964	1.303	1.241
2645433	CHD3 [Homo sapiens]	9	21	regulation of biological process	1.176	1.303	1.332
7679456	PREDICTED: lanC-like protein 2 isoform X1 [Homo sapiens]	2	8	; regulation of biological process	1.180	1.303	1.357
1196277	splicing factor 3a, subunit 3, 60kDa, isoform CRA_b [Homo sapien	3	17	cell organization and biogenesis	0.857	1.303	1.138
1487533	RCC2 protein, partial [Homo sapiens]	18	98	esponse to stimulus; cell division	1.161	1.302	1.288
7680218	PREDICTED: proteasome assembly chaperone 1 isoform X1 [Home	3	9	cell organization and biogenesis	1.059	1.300	1.161
7679062	PREDICTED: F-actin-capping protein subunit alpha-1 isoform X1 [H	5	47	; regulation of biological process	1.091	1.299	1.314
5303954	PREDICTED: solute carrier family 22 member 18 isoform X9 [Homo	2	3	transport	1.276	1.297	1.252
1195747	pyrophosphatase (inorganic) 1, isoform CRA_a [Homo sapiens]	3	15	metabolic process	1.067	1.297	1.444
1660142	enabled-like protein variant hMenaDv6 [Homo sapiens]	7	38	al process; response to stimulus	0.926	1.297	1.264
5788039	PREDICTED: heterogeneous nuclear ribonucleoprotein A3 isoform	9	46	metabolic process; transport	1.272	1.296	1.741

1196135	HECT, UBA and WWE domain containing 1, isoform CRA_a [Homo	49	176	nd biogenesis; cell differentiation	0.955	1.295	1.240
5303968	PREDICTED: double-strand break repair protein MRE11A isoform X	2	10	l proliferation; defense response	1.095	1.295	1.309
1521495	Chain A, Human Nsun5 Protein	3	6		1.062	1.294	1.283
4825707	CTNND1 protein, partial [Homo sapiens]	16	54	cell organization and biogenesis	0.989	1.294	1.339
1269794	KIAA1699 protein, partial [Homo sapiens]	10	36	sis; transport; metabolic process	1.024	1.293	1.358
1195886	lin-7 homolog C (C. elegans), isoform CRA_b [Homo sapiens]	2	5	cell organization and biogenesis	1.047	1.293	1.026
1196237	chromosome 6 open reading frame 134, isoform CRA_c, partial [Ho	2	4	gical process; metabolic process	0.912	1.293	1.299
1195890	transmembrane protein 41B, isoform CRA_d, partial [Homo sapien	3	8		0.798	1.292	1.244
1193901	Chain A, Crystal Structure Of The Heterodimeric Complex Of Huma	6	25	al process; response to stimulus	1.008	1.290	1.287
2972660	Chain B, Crystal Structure Of Human Junctional Adhesion Molecul	5	26		0.851	1.290	1.146
7680366	PREDICTED: mediator of RNA polymerase II transcription subunit	3	5	ic process; response to stimulus	1.091	1.290	1.174
3315082	MSIN3A [Homo sapiens]	11	28	o stimulus; cellular homeostasis	0.948	1.288	1.268
7680005	PREDICTED: ATP-dependent RNA helicase DDX39A isoform X1 [Ho	14	122	; regulation of biological process	1.048	1.288	1.178
2311101	RNA-binding protein 10 isoform 2 [Homo sapiens]	4	18	metabolic process	0.891	1.287	1.266
7679038	PREDICTED: uncharacterized protein C1orf167 isoform X8 [Homo	2	4		0.838	1.287	1.333
5566953	nucleoside diphosphate kinase, mitochondrial isoform b [Homo sa	3	13	; regulation of biological process	1.079	1.287	1.253
5303793	PREDICTED: ATP-dependent RNA helicase DHX29 isoform X3 [Hom	5	10	metabolic process	0.998	1.286	1.213
3187445	hypothetical protein [Homo sapiens]	2	8	metabolic process	0.945	1.286	1.249
448295	TLS protein	6	19		1.073	1.286	1.036
4004709	unnamed protein product [Homo sapiens]	2	7		0.877	1.286	1.232
5207839	USP19 protein, partial [Homo sapiens]	4	7	al process; response to stimulus	0.892	1.286	1.159
704416	elongation factor Tu [Homo sapiens]	11	152	cell organization and biogenesis	1.045	1.286	1.334
1195776	ethylmalonic encephalopathy 1, isoform CRA_b [Homo sapiens]	4	24	metabolic process	1.042	1.285	1.233
2119391	Chain H, Crystal Structure Of Zn2+-Bound Form Of Des3-23alg-2	4	19		1.137	1.285	1.383
1196108	transmembrane protein 93 [Homo sapiens]	2	3	d biogenesis; metabolic process	0.988	1.285	1.401
5788008	PREDICTED: melanoma inhibitory activity protein 3 isoform X2 [Ho	6	17	biogenesis; response to stimulus	0.958	1.285	1.205
3883269	Chain A, Discovery Of Small Molecules That Bind To K-Ras And In	4	10		0.892	1.284	1.287
1892178	maspardin isoform b [Homo sapiens]	2	6	al process; response to stimulus	0.826	1.284	1.106
1937857	unnamed protein product [Homo sapiens]	2	2	; regulation of biological process	0.876	1.284	1.155
7679729	PREDICTED: bromodomain adjacent to zinc finger domain protein	3	4	cell organization and biogenesis	1.081	1.284	1.294
1616920	a member of the short-chain alcohol dehydrogenase family, partial	3	10	metabolic process	0.834	1.283	1.166
3522970	Trio [Homo sapiens]	4	12	s; cellular component movement	0.855	1.282	1.300
1195836	voltage-dependent anion channel 3, isoform CRA_b [Homo sapien	11	64	; regulation of biological process	1.102	1.282	1.191
2518371	Chain B, Leishmania Tarentolae Mitochondrial Large Ribosomal Su	2	6	metabolic process	0.922	1.281	1.236
1195986	EPH receptor B3, isoform CRA_a [Homo sapiens]	4	10	cell organization and biogenesis	1.062	1.280	1.323
3141720	ASPSCR1 protein [Homo sapiens]	6	22	cell organization and biogenesis	0.970	1.280	1.164
4760749	plectin isoform 1c [Homo sapiens]	138	721	cell organization and biogenesis	1.016	1.280	1.256
5842771	receptor tyrosine-protein kinase erbB-2 isoform d precursor [Home	5	9	nse response; cell differentiation	0.959	1.279	1.180
1582594	unnamed protein product [Homo sapiens]	5	22	metabolic process	0.904	1.278	1.252
1012060	Chain B, L-3-Hydroxyacyl-Coa Dehydrogenase Complexed With Ac	9	46	metabolic process	1.271	1.276	1.330

1196316	dynein, cytoplasmic 1, intermediate chain 2, isoform CRA_a [Homo	5	26	nt movement; metabolic process	1.184	1.276	1.248
1591490	DIS3 [Homo sapiens]	11	30	; regulation of biological process	1.114	1.276	1.268
1196297	protein O-fucosyltransferase 2, isoform CRA_c [Homo sapiens]	2	5	; regulation of biological process	0.978	1.276	1.372
5304250	PREDICTED: phosphatidate phosphatase LPIN2 isoform X1 [Homo	2	2	; regulation of biological process	0.762	1.275	1.142
229080	DNA repair enzyme	2	4	ic process; response to stimulus	1.025	1.275	1.151
1943879	unnamed protein product [Homo sapiens]	4	15	; regulation of biological process	1.018	1.274	1.510
6298884	unknown, partial [Homo sapiens]	34	159	ent movement; defense response	1.048	1.273	1.281
1196060	chromosome 16 open reading frame 34, isoform CRA_d [Homo sa	2	9		0.821	1.272	1.270
3867833	Chain B, Crystal Structure Of A Ribonucleotide Reductase M2 B (r	2	5		0.881	1.272	1.182
1943748	unnamed protein product [Homo sapiens]	4	21	metabolic process	1.211	1.272	1.376
1196075	inositol(myo)-1(or 4)-monophosphatase 1, isoform CRA_d [Homo s	4	12	al process; response to stimulus	0.934	1.271	1.210
1195763	WD repeat domain 70, isoform CRA_a [Homo sapiens]	3	7		0.926	1.271	1.233
1943797	unnamed protein product [Homo sapiens]	6	17	metabolic process	1.161	1.270	1.251
1196109	hCG32973, isoform CRA_b [Homo sapiens]	2	6	proliferation; metabolic process	0.935	1.270	1.086
1890550	unnamed protein product [Homo sapiens]	4	12	cell organization and biogenesis	1.028	1.270	1.301
1195967	protein O-fucosyltransferase 1, isoform CRA_b [Homo sapiens]	2	5	al process; response to stimulus	1.075	1.269	1.358
1527721	putative ATP-dependent RNA helicase [Homo sapiens]	14	139	; regulation of biological process	1.039	1.268	1.164
3328642	pyruvate kinase PKM isoform b [Homo sapiens]	26	445	us; metabolic process; cell death	1.105	1.268	1.272
1195741	mitogen-activated protein kinase 9, isoform CRA_d [Homo sapiens	2	4	ess; transport; defense response	1.176	1.268	1.229
1195772	heterogeneous nuclear ribonucleoprotein L, isoform CRA_a [Homo	14	58	metabolic process	0.917	1.267	1.295
1890549	unnamed protein product [Homo sapiens]	3	5	metabolic process	0.950	1.267	1.088
2276124	unnamed protein product [Homo sapiens]	7	15	us; metabolic process; cell death	1.039	1.267	1.251
1943822	unnamed protein product [Homo sapiens]	7	45	oliferation; response to stimulus	0.968	1.267	1.217
1582602	unnamed protein product [Homo sapiens]	6	21	metabolic process	0.917	1.267	1.302
1195750	ribosomal protein S24, isoform CRA_i [Homo sapiens]	2	9	ization and biogenesis; transport	1.470	1.267	1.168
1196099	hCG1642326, isoform CRA_a [Homo sapiens]	2	3	metabolic process	1.018	1.265	1.197
7021912	unnamed protein product [Homo sapiens]	2	12	ss; metabolic process; transport	0.891	1.265	1.158
4097601	unnamed protein product, partial [Homo sapiens]	3	4		1.216	1.265	1.322
2743694	lamin isoform A [Homo sapiens]	33	337	us; transport; metabolic process	1.084	1.265	1.250
5262603	hypothetical protein, partial [Homo sapiens]	4	5	d biogenesis; metabolic process	1.041	1.264	1.353
7679588	PREDICTED: protein transport protein Sec16A isoform X12 [Homo	11	53	d biogenesis; metabolic process	0.956	1.264	1.155
1890538	unnamed protein product [Homo sapiens]	10	55	cell organization and biogenesis	1.095	1.263	1.226
1890654	unnamed protein product [Homo sapiens]	5	27	e to stimulus; metabolic process	0.989	1.263	1.239
6263020	unknown, partial [Homo sapiens]	10	37	metabolic process	0.980	1.263	1.248
1582563	unnamed protein product [Homo sapiens]	3	5	metabolic process	0.794	1.262	1.131
4240263	KIAA0887 protein, partial [Homo sapiens]	6	29	; regulation of biological process	1.133	1.262	1.195
2146605	Chain A, Crystal Structure Of The Mitochondrial Serine Protease H	6	14	metabolic process	1.135	1.262	1.194
7679228	PREDICTED: probable leucine--tRNA ligase, mitochondrial isoform	3	11	; regulation of biological process	1.069	1.262	1.095
1196268	fucose-1-phosphate guanylyltransferase [Homo sapiens]	2	3	metabolic process	1.024	1.261	1.141
2790032	unnamed protein product [Homo sapiens]	3	16		0.830	1.261	1.199

2143868	unnamed protein product [Homo sapiens]	16	62		1.128	1.260	1.263
3351888	stromal RNA regulating factor [Homo sapiens]	5	15	; regulation of biological process	0.890	1.260	1.344
1195746	chromosome 10 open reading frame 22, isoform CRA_b [Homo sa	2	2	metabolic process	0.792	1.260	1.121
1196269	hCG2031840, isoform CRA_d [Homo sapiens]	4	10	metabolic process	1.148	1.260	1.072
3145553	putative MAPK activating protein [Homo sapiens]	4	12	cell organization and biogenesis	0.834	1.259	1.307
2612788	Chain D, Human Dipeptidyl Peptidase Dpp7	10	38	metabolic process	1.016	1.259	1.209
4876294	huntingtin-interacting protein 1-related protein isoform 1 [Homo sa	10	30	biogenesis; transport; cell death	0.911	1.259	1.135
1196060	KIAA0683 gene product, isoform CRA_a [Homo sapiens]	4	9	regulation of biological process	0.936	1.258	1.279
1597950	Chain A, The Structure Of Receptor-Associated Protein(Rap)	2	7		0.788	1.258	1.133
1926376	Similar to cytoskeleton-associated protein 4, partial [Homo sapiens	16	65	metabolic process	1.095	1.258	1.382
7679128	PREDICTED: CSC1-like protein 1 isoform X2 [Homo sapiens]	3	7	transport	0.990	1.258	1.196
1890693	unnamed protein product [Homo sapiens]	3	11	cell organization and biogenesis	0.875	1.258	1.306
3006478	unnamed protein product [Homo sapiens]	4	9		1.147	1.257	1.157
6973514	Chain B, Methyltransferase Domain Of C9orf114	2	6		0.976	1.257	1.151
6399111	unknown, partial [Homo sapiens]	5	18	tion and biogenesis; cell division	0.942	1.257	1.259
1196124	family with sequence similarity 84, member B [Homo sapiens]	2	5		0.836	1.256	1.144
7680313	PREDICTED: plexin-B2 isoform X1 [Homo sapiens]	10	19	s; development; cell proliferation	0.922	1.256	1.193
4085360	Chain D, Pkm2 In Complex With An Activator	29	463		1.123	1.256	1.255
7679088	PREDICTED: SLIT-ROBO Rho GTPase-activating protein 2 isoform	3	8	nent movement; cell proliferation	0.880	1.255	1.171
1890693	unnamed protein product [Homo sapiens]	15	63	meostasis; response to stimulus	1.076	1.254	1.143
189308	nucleobindin [Homo sapiens]	9	46	; regulation of biological process	1.287	1.254	1.313
2386241	neuroplastin isoform d precursor [Homo sapiens]	2	4	homeostasis; metabolic process	1.077	1.253	1.428
1196128	Rtf1, Paf1/RNA polymerase II complex component, homolog (S. ce	3	7	cell organization and biogenesis	0.882	1.253	1.168
1890533	unnamed protein product [Homo sapiens]	2	13	cell organization and biogenesis	0.806	1.252	1.126
1582566	unnamed protein product [Homo sapiens]	13	142	cell organization and biogenesis	1.074	1.252	1.266
1196248	hCG42063, isoform CRA_a, partial [Homo sapiens]	7	28		1.083	1.251	1.322
1890532	unnamed protein product [Homo sapiens]	4	15	ization and biogenesis; transport	0.914	1.250	1.298
1582590	unnamed protein product [Homo sapiens]	8	37		1.010	1.250	1.089
7679782	PREDICTED: succinyl-CoA ligase [ADP-forming] subunit beta, mito	3	5	metabolic process	1.156	1.249	1.342
7679111	PREDICTED: acyl-CoA:lysophosphatidylglycerol acyltransferase 1	2	2	metabolic process	0.965	1.249	1.296
5559437	general transcription factor 3C polypeptide 1 isoform 2 [Homo sap	14	24	metabolic process	0.953	1.247	1.182
5577861	TBC1 domain family member 4 isoform 3 [Homo sapiens]	5	21	cell organization and biogenesis	0.823	1.247	1.155
1578354	Chain B, Crystal Structure Of Nfkb, Irf7, Irf3 Bound To The Interfero	3	4	regulation of biological process	1.289	1.247	1.158
1937873	unnamed protein product [Homo sapiens]	3	7	; regulation of biological process	0.870	1.245	1.173
1195804	RNA binding motif protein 9, isoform CRA_c [Homo sapiens]	2	5	cell organization and biogenesis	1.616	1.245	1.245
5304093	PREDICTED: mitochondrial Rho GTPase 2 isoform X3 [Homo sapie	8	13	cell organization and biogenesis	0.881	1.245	1.231
8209575	Chain B, Crystal Structure Of Human Fmnl2 Gbd-fh3 Domains Bou	2	5		0.922	1.245	1.168
7679034	PREDICTED: ubiquitin carboxyl-terminal hydrolase 24 isoform X2 [	8	12	; regulation of biological process	0.886	1.245	1.108
1943777	unnamed protein product [Homo sapiens]	2	3	cell organization and biogenesis	0.839	1.244	1.178
1460346	Ribosomal protein L28 [Homo sapiens]	7	54	; regulation of biological process	0.903	1.244	1.145

5304235	PREDICTED: casein kinase II subunit alpha' isoform X1 [Homo sap	2	5	movement; response to stimulus	1.017	1.244	1.180
7679572	PREDICTED: heterogeneous nuclear ribonucleoprotein K isoform	16	304	al process; response to stimulus	1.090	1.244	1.259
106731	inositol-1,4,5-trisphosphate 5-phosphatase (EC 3.1.3.56) - human (	2	2	al process; response to stimulus	0.937	1.243	1.128
2210412	unnamed protein product [Homo sapiens]	2	6		0.976	1.242	1.045
1196123	oxidation resistance 1, isoform CRA_c [Homo sapiens]	2	3	; regulation of biological process	0.848	1.241	1.457
1195947	transmembrane 7 superfamily member 2, isoform CRA_a [Homo sa	5	25	metabolic process	0.992	1.240	1.122
5788239	PREDICTED: 5'-AMP-activated protein kinase subunit gamma-1 iso	4	8	to stimulus; cell communication	1.070	1.239	1.229
3478553	REEP3 protein, partial [Homo sapiens]	2	4	tion and biogenesis; cell division	0.783	1.239	1.131
6208827	metastasis associated protein variant, partial [Homo sapiens]	9	34	; regulation of biological process	0.920	1.237	1.239
1943843	unnamed protein product [Homo sapiens]	8	21	; transport; response to stimulus	1.113	1.236	1.112
1582586	unnamed protein product [Homo sapiens]	4	9		0.870	1.236	1.217
7679510	PREDICTED: cell cycle and apoptosis regulator protein 2 isoform X	13	57	cell organization and biogenesis	1.063	1.235	1.144
1196061	STIP1 homology and U-box containing protein 1, isoform CRA_c [H	7	37	; regulation of biological process	1.149	1.235	1.241
7679387	PREDICTED: clathrin interactor 1 isoform X3 [Homo sapiens]	2	2	cell organization and biogenesis	0.888	1.235	1.086
1195944	inner centromere protein antigens 135/155kDa, isoform CRA_a [Ho	2	2	al process; response to stimulus	0.863	1.233	1.128
1195972	cytochrome P450, family 51, subfamily A, polypeptide 1, isoform C	5	17	metabolic process	0.918	1.233	1.086
7679567	PREDICTED: constitutive coactivator of PPAR-gamma-like protein	17	48		0.986	1.233	1.236
685174	MAP kinase kinase 3 [Homo sapiens]	5	13	bolic process; defense response	1.008	1.233	1.282
1196025	plectin 1, intermediate filament binding protein 500kDa, isoform CR	92	474	cell organization and biogenesis	1.019	1.233	1.212
1890652	unnamed protein product [Homo sapiens]	2	9	cell organization and biogenesis	1.041	1.233	1.306
7679588	PREDICTED: polypyrimidine tract-binding protein 3 isoform X3 [Ho	6	29	; regulation of biological process	1.265	1.232	1.260
4680671	CGI-16 protein [Homo sapiens]	3	9	metabolic process	1.110	1.232	1.159
5430399	peroxiredoxin 6, partial [Homo sapiens]	4	49	e to stimulus; metabolic process	1.049	1.231	1.317
1196256	spermatogenesis associated 5, isoform CRA_a [Homo sapiens]	2	10	development; cell differentiation	0.994	1.230	1.327
4758648	kinesin-1 heavy chain [Homo sapiens]	16	62	; regulation of biological process	1.007	1.230	1.274
1196105	SUMO1/sentrin/SMT3 specific peptidase 3, isoform CRA_c [Homo	3	4	metabolic process	0.959	1.230	1.179
1186009	NUP43 protein, partial [Homo sapiens]	4	24	stimulus; transport; cell division	0.911	1.229	1.294
3806923	probable ATP-dependent RNA helicase DDX56 isoform 2 [Homo sa	2	4	metabolic process	0.884	1.228	1.148
7679122	PREDICTED: adenylosuccinate synthetase isozyme 2 isoform X2 [I	3	7	ic process; response to stimulus	0.861	1.227	1.135
1937864	unnamed protein product [Homo sapiens]	2	3	cell organization and biogenesis	0.789	1.227	1.230
1195961	nuclear receptor coactivator 5, isoform CRA_c [Homo sapiens]	8	29	; regulation of biological process	1.090	1.227	1.223
1196006	ras homolog gene family, member T1, isoform CRA_f [Homo sapie	2	2	cell organization and biogenesis	1.008	1.227	1.194
238427	Porin 31HM [human, skeletal muscle membranes, Peptide, 282 aa]	10	46	; regulation of biological process	1.064	1.227	1.251
1196082	spectrin, alpha, non-erythrocytic 1 (alpha-fodrin), isoform CRA_c [I	50	227	ent movement; defense response	1.005	1.226	1.217
1582612	unnamed protein product [Homo sapiens]	2	5	; regulation of biological process	0.825	1.226	1.065
1943818	unnamed protein product [Homo sapiens]	4	12		0.970	1.226	1.193
1195931	host cell factor C1 (VP16-accessory protein), isoform CRA_c [Hom	19	56	ic process; response to stimulus	0.981	1.226	1.178
1943771	unnamed protein product [Homo sapiens]	2	5	metabolic process; transport	0.807	1.226	1.101
1937861	unnamed protein product [Homo sapiens]	3	4		1.110	1.226	1.186
7311861	TSC1-PDGFEB fusion protein, partial [Homo sapiens]	2	4	al process; response to stimulus	0.932	1.225	0.989

3787925	Chain D, Contribution Of The First K-homology Domain Of Poly(c)-	3	5		1.154	1.224	1.210
1195722	hCG2039634, isoform CRA_e [Homo sapiens]	10	25	; regulation of biological process	1.114	1.224	1.233
1174072	G alpha-q [Homo sapiens]	3	9	; coagulation; metabolic process	0.910	1.224	1.234
5304204	PREDICTED: protein NipSnap homolog 1 isoform X1 [Homo sapien	3	22		1.112	1.224	1.086
5303741	PREDICTED: U2 snRNP-associated SURP motif-containing protein	4	10	metabolic process	0.866	1.224	1.155
5304182	PREDICTED: nuclear receptor coactivator 5 isoform X1 [Homo sap	8	32	; regulation of biological process	1.030	1.224	1.228
1043828	unnamed protein product [Homo sapiens]	2	4	transport	0.933	1.223	1.215
1354361	ATP synthase, H+ transporting, mitochondrial F0 complex, subunit	9	44	metabolic process; transport	1.097	1.223	1.142
3909807	Chain A, Solution Nmr Structure Of The Globular Domain Of Huma	3	8		0.896	1.223	1.446
2737095	PRPF38B protein, partial [Homo sapiens]	2	3	metabolic process	0.788	1.222	1.268
1582548	unnamed protein product [Homo sapiens]	6	24	metabolic process	1.147	1.222	1.087
1196171	poly(rC) binding protein 2, isoform CRA_f [Homo sapiens]	7	86	response; response to stimulus	1.086	1.221	1.179
2052183	KIAA0178 [Homo sapiens]	8	14	f biological process; cell division	0.985	1.219	1.190
1943844	unnamed protein product [Homo sapiens]	3	8	e to stimulus; metabolic process	1.021	1.219	1.082
7679265	PREDICTED: acyl-CoA dehydrogenase family member 9, mitochon	9	26	cell organization and biogenesis	1.069	1.218	1.199
5303775	PREDICTED: N-acylethanolamine-hydrolyzing acid amidase isofor	4	8	metabolic process	1.050	1.217	1.210
5304142	PREDICTED: asparagine--tRNA ligase, cytoplasmic isoform X1 [Ho	13	56	metabolic process	1.211	1.217	1.221
1943763	unnamed protein product [Homo sapiens]	3	12	cell organization and biogenesis	0.966	1.216	1.244
1943865	unnamed protein product [Homo sapiens]	9	30	tion and biogenesis; cell division	1.137	1.215	1.202
1578382	Chain A, Tyrosine Phosphorylated Stat-1DNA COMPLEX	2	4	al process; response to stimulus	0.919	1.215	1.132
5083388	unnamed protein product [Homo sapiens]	3	8		0.962	1.214	1.204
1943865	unnamed protein product [Homo sapiens]	2	6	; regulation of biological process	0.999	1.214	0.997
1196266	HDCMA18P protein, isoform CRA_c [Homo sapiens]	2	5	tabolic process; cell proliferation	0.816	1.212	1.108
1195943	transmembrane protein 109 [Homo sapiens]	2	30	al process; response to stimulus	1.004	1.212	1.137
3453014	unnamed protein product [Homo sapiens]	3	4	al process; response to stimulus	1.015	1.211	1.253
1034854	charged multivesicular body protein 1a isoform 2 [Homo sapiens]	3	13	sponse to stimulus; cell division	1.106	1.211	1.115
1196149	tripartite motif-containing 25, isoform CRA_b [Homo sapiens]	8	21	gical process; defense response	0.961	1.211	1.162
5797624	unnamed protein product [Homo sapiens]	2	2		0.971	1.211	1.116
5788191	PREDICTED: NAD-dependent protein deacetylase sirtuin-1 isoform	2	3	mmunication; cell differentiation	0.734	1.211	1.336
1076413	PELOTA [Homo sapiens]	2	3	; cell division; metabolic process	0.922	1.211	1.075
1195820	mitochondrial ribosomal protein L22 [Homo sapiens]	2	6	cell organization and biogenesis	0.852	1.210	1.205
7679606	PREDICTED: cullin-2 isoform X1 [Homo sapiens]	14	31	al process; response to stimulus	1.093	1.210	1.040
1195921	mitofusin 2, isoform CRA_b [Homo sapiens]	3	8	f biological process; coagulation	0.843	1.210	1.124
2173226	hypothetical protein, partial [Homo sapiens]	2	2	cell organization and biogenesis	0.889	1.209	1.178
1167347	interferon regulatory factor 2-binding protein 2 isoform B [Homo sa	4	23	; regulation of biological process	0.987	1.209	1.149
5788159	PREDICTED: 39S ribosomal protein L13, mitochondrial isoform X1	4	6	cell organization and biogenesis	0.996	1.209	1.230
6270228	unknown [Homo sapiens]	11	30	nd biogenesis; defense response	1.123	1.208	1.225
7680567	PREDICTED: E3 ubiquitin-protein ligase HERC2 isoform X5 [Homo	4	7	; regulation of biological process	1.033	1.208	1.174
1943849	unnamed protein product [Homo sapiens]	3	6	ic process; response to stimulus	0.819	1.207	1.257
1195704	kinesin family member 11 [Homo sapiens]	6	13	f biological process; cell division	1.009	1.207	1.074

1582591	unnamed protein product [Homo sapiens]	4	18	regulation of biological process	1.034	1.207	1.244
1195846	TBC1 domain family, member 5, isoform CRA_a [Homo sapiens]	4	8	; transport; response to stimulus	0.799	1.206	1.233
1196081	cyclin-dependent kinase 9 (CDC2-related kinase), isoform CRA_a [	5	19	logical process; cell proliferation	0.866	1.206	1.139
1195727	ribosomal protein L18, isoform CRA_b [Homo sapiens]	6	72	ization and biogenesis; transport	1.143	1.206	1.114
7679870	PREDICTED: DNA repair endonuclease XPF isoform X4 [Homo sap	2	8	; regulation of biological process	0.955	1.205	1.256
1943891	unnamed protein product [Homo sapiens]	3	9	ic process; response to stimulus	1.104	1.205	1.175
7680316	PREDICTED: serine/threonine-protein phosphatase 6 regulatory su	3	8		0.833	1.204	1.137
1195903	chromosome 1 open reading frame 57, isoform CRA_c [Homo sapi	2	8	ic process; response to stimulus	0.929	1.204	1.138
5489236	2-hydroxyacyl-CoA lyase 1 isoform d [Homo sapiens]	5	20	cell organization and biogenesis	0.940	1.204	1.121
1196172	cyclin-dependent kinase 2, isoform CRA_a [Homo sapiens]	3	9	enesis; coagulation; cell division	1.295	1.204	1.155
1196294	propionyl Coenzyme A carboxylase, alpha polypeptide, isoform CR	6	10	metabolic process	1.047	1.203	1.201
1195976	cancer susceptibility candidate 4, isoform CRA_c [Homo sapiens]	2	6		0.764	1.202	1.201
5443462	DNA-directed RNA polymerase I subunit RPA2 isoform 4 [Homo sa	3	5	; regulation of biological process	1.040	1.202	0.974
4003110	unnamed protein product [Homo sapiens]	2	8		1.262	1.201	1.040
5304144	PREDICTED: nucleus accumbens-associated protein 1 isoform X1	2	8	cell organization and biogenesis	0.999	1.201	1.103
2240282	non-POU domain-containing octamer-binding protein isoform 2 [H	14	107	; regulation of biological process	1.043	1.200	1.162
8819222	Chain B, Structure Of A Monoclinic Crystal Form Of Human Cytoso	11	38	metabolic process	1.073	1.200	1.223
1061128	anpg, partial [Homo sapiens]	7	22	ic process; response to stimulus	0.845	1.199	1.260
2996893	Chain A, The Catalytic Domain Of Usp8 In Complex With A Usp8 S	2	4	metabolic process	1.030	1.199	1.100
223597	actin beta related pseudogene	2	3		1.042	1.199	1.185
1196209	hCG16536, isoform CRA_b [Homo sapiens]	2	4		1.241	1.199	1.200
1196047	regulatory factor X, 1 (influences HLA class II expression) [Homo s	4	6	al process; response to stimulus	1.024	1.199	1.252
1196190	hCG1820685 [Homo sapiens]	2	7		1.120	1.198	1.104
1196080	chromosome 9 open reading frame 126, isoform CRA_b [Homo sa	2	4	al process; response to stimulus	0.873	1.198	1.203
4767861	PK1.3 [Homo sapiens]	3	8	; regulation of biological process	1.132	1.198	1.183
5430390	aging-associated gene 7 protein [Homo sapiens]	5	27	esponse to stimulus; cell division	0.923	1.197	1.062
7679163	PREDICTED: metaxin-2 isoform X1 [Homo sapiens]	2	3	transport; metabolic process	1.030	1.197	1.160
8240795	Chain F, Crystal Structure Of 14-3-3 Gamma In Complex With A Ph	12	200		1.088	1.197	1.163
1195789	smu-1 suppressor of mec-8 and unc-52 homolog (C. elegans), isof	9	28		1.084	1.197	1.143
1943885	unnamed protein product [Homo sapiens]	3	23	n; cellular component movement	0.898	1.196	1.061
1195921	huntingtin interacting protein 1, isoform CRA_a [Homo sapiens]	5	13	erentiation; response to stimulus	0.902	1.196	0.992
1403582	unnamed protein product [Homo sapiens]	6	23		1.108	1.196	1.242
1195957	opioid growth factor receptor, isoform CRA_b [Homo sapiens]	4	7	al process; response to stimulus	1.143	1.195	1.288
1534185	Ribosomal protein L8 [Homo sapiens]	4	46	ization and biogenesis; transport	0.874	1.195	1.196
2498733	Chain B, Structure Of The Human Nuclear Cap-Binding-Complex (C	3	7	metabolic process	1.067	1.195	1.100
1582618	unnamed protein product [Homo sapiens]	4	14	; regulation of biological process	1.024	1.194	1.175
1582548	unnamed protein product [Homo sapiens]	10	43	al process; response to stimulus	1.189	1.194	1.219
2557599	WD repeat-containing protein 81 isoform 1 [Homo sapiens]	3	4	gical process; cell differentiation	0.821	1.194	1.123
5410272	maleylacetoacetate isomerase [Homo sapiens]	2	6	metabolic process	1.006	1.194	1.115
1196028	huntingtin (Huntington disease), isoform CRA_c [Homo sapiens]	6	10	component movement; cell death	1.022	1.193	1.124

1195930	tripartite motif-containing 28, isoform CRA_c [Homo sapiens]	17	140	se to stimulus; defense response	0.968	1.193	1.127
1195934	ATP-binding cassette, sub-family D (ALD), member 3, isoform CRA	13	63	biogenesis; response to stimulus	1.099	1.192	1.153
1196194	elongation factor Tu GTP binding domain containing 1, isoform CR	2	3	cell organization and biogenesis	0.901	1.192	1.131
7680044	PREDICTED: lamin-B2 isoform X1 [Homo sapiens]	29	203		1.055	1.192	1.294
5989713	macoilin isoform 2 [Homo sapiens]	2	5		0.834	1.191	1.132
1943742	unnamed protein product [Homo sapiens]	4	8	metabolic process	1.097	1.191	1.100
7679151	PREDICTED: nucleolysin TIA-1 isoform p40 isoform X1 [Homo sap	8	37	al process; response to stimulus	0.963	1.191	1.045
5788265	PREDICTED: secretory carrier-associated membrane protein 2 isof	4	26	transport	0.946	1.191	1.265
1890537	unnamed protein product [Homo sapiens]	12	90	metabolic process	0.973	1.191	1.110
1943883	unnamed protein product [Homo sapiens]	5	13	metabolic process	0.876	1.190	1.153
7679108	PREDICTED: U4/U6 small nuclear ribonucleoprotein Prp3 isoform	5	9	d biogenesis; metabolic process	1.111	1.189	1.146
4814604	VDAC2 [Homo sapiens]	10	69	; regulation of biological process	1.079	1.189	1.194
2819318	unnamed protein product (plasmid) [Homo sapiens]	2	8	; regulation of biological process	1.090	1.189	1.112
1196221	THO complex 1, isoform CRA_c [Homo sapiens]	4	8	cell death; response to stimulus	0.869	1.188	1.136
1195938	hypothetical protein FLJ20699, isoform CRA_b [Homo sapiens]	4	14		1.032	1.188	1.113
7679908	PREDICTED: ubiquitin carboxyl-terminal hydrolase 10 isoform X3 [	6	15	; regulation of biological process	1.143	1.188	1.193
1196137	neuron derived neurotrophic factor [Homo sapiens]	2	4	regulation of biological process	1.181	1.188	1.230
1639653	thiosulfate sulfurtransferase/rhodanese-like domain-containing pr	3	12		0.850	1.188	1.080
1943814	unnamed protein product [Homo sapiens]	2	3	; regulation of biological process	0.977	1.188	1.109
1196168	cytidine monophosphate N-acetylneuraminic acid synthetase, isof	5	20	metabolic process	1.177	1.187	1.393
1943844	unnamed protein product [Homo sapiens]	8	41	metabolic process; transport	1.245	1.187	1.108
1582580	unnamed protein product [Homo sapiens]	11	58	metabolic process; transport	1.042	1.187	1.134
4030720	Chain F, Espg-Rab1-Arf6 Complex	3	7		1.047	1.186	1.106
1943902	unnamed protein product [Homo sapiens]	2	3		0.862	1.186	1.111
1196118	ADP-ribosylation factor-like 8A, isoform CRA_b [Homo sapiens]	4	16	sponse to stimulus; cell division	1.078	1.185	1.084
5304006	PREDICTED: ras-related protein Rab-5B isoform X1 [Homo sapiens	9	51	al process; response to stimulus	1.114	1.185	1.068
4097910	unnamed protein product, partial [Homo sapiens]	3	6		0.844	1.185	1.118
2737074	Similar to kinesin family member 21A, partial [Homo sapiens]	3	6	; regulation of biological process	0.938	1.184	1.085
1196219	AFG3 ATPase family gene 3-like 2 (yeast), isoform CRA_a, partial [	8	40	n of biological process; transport	1.077	1.183	1.256
2586140	branched-chain-amino-acid aminotransferase, mitochondrial isofo	8	31	metabolic process	1.061	1.183	1.203
3169831	NADH-ubiquinone oxidoreductase 75 kDa subunit, mitochondrial i	11	40	cell organization and biogenesis	0.988	1.182	1.165
6841066	calcium-binding transporter, partial [Homo sapiens]	4	10	al process; response to stimulus	1.176	1.182	1.175
1582605	unnamed protein product [Homo sapiens]	17	73	tion and biogenesis; cell division	1.041	1.182	1.110
7679254	PREDICTED: cohesin subunit SA-1 isoform X1 [Homo sapiens]	6	11	f biological process; cell division	0.988	1.182	1.118
4196362	AP-3 complex subunit beta-1 isoform 2 [Homo sapiens]	16	50	; regulation of biological process	1.050	1.181	1.166
1195721	Vac14 homolog (S. cerevisiae), isoform CRA_c [Homo sapiens]	4	13	al process; response to stimulus	1.201	1.181	1.074
7679203	PREDICTED: calcium-binding mitochondrial carrier protein Aralar1	11	51	; transport; response to stimulus	1.060	1.181	1.195
6263018	unknown, partial [Homo sapiens]	16	64	metabolic process	1.019	1.181	1.105
7679200	PREDICTED: integrin-linked kinase-associated serine/threonine ph	4	11	metabolic process	1.081	1.180	1.095
6650816	PRO1975 [Homo sapiens]	6	12	metabolic process	1.025	1.180	1.092

7679315	PREDICTED: ELMO domain-containing protein 2 isoform X1 [Homo	4	23	e response; response to stimulus	1.054	1.180	1.096
5788093	PREDICTED: rap1 GTPase-GDP dissociation stimulator 1 isoform X	3	8	to stimulus; cellular homeostasis	1.012	1.180	1.208
1943854	unnamed protein product [Homo sapiens]	2	5	metabolic process	0.744	1.179	1.058
1181428	NOP56 protein, partial [Homo sapiens]	12	69	metabolic process	1.127	1.179	1.153
5788397	PREDICTED: elongation factor 1-delta isoform X6 [Homo sapiens]	8	124	al process; response to stimulus	0.969	1.179	1.077
3174551	Chain B, Crystal Structure Of Human Nudt5 Complexed With 8-oxo	3	6		1.062	1.179	0.996
1196137	lysophospholipase-like 1, isoform CRA_d [Homo sapiens]	4	12	; regulation of biological process	0.938	1.179	1.237
1205399	RNA binding motif protein 12 [Homo sapiens]	7	37		0.954	1.177	1.095
1184805	TPA_exp: RNA binding motif protein 15 isoform L2 [Homo sapiens	12	25	; regulation of biological process	0.915	1.177	1.128
5303850	PREDICTED: mitotic spindle assembly checkpoint protein MAD1 is	5	11	esponse to stimulus; cell division	0.927	1.176	1.114
1582559	unnamed protein product [Homo sapiens]	9	23	; regulation of biological process	0.983	1.176	1.192
1180269	8-oxo-dGDP phosphatase NUDT18 [Homo sapiens]	3	8	metabolic process	0.953	1.176	1.123
1195856	transmembrane protein 113, isoform CRA_a [Homo sapiens]	5	12	d biogenesis; metabolic process	0.951	1.175	1.208
7679065	PREDICTED: eukaryotic translation initiation factor 4 gamma 3 iso	6	19	al process; response to stimulus	1.103	1.175	1.286
1195807	solute carrier family 25 (mitochondrial carrier; peroxisomal membr	2	7	metabolic process; transport	0.896	1.175	1.053
7679241	PREDICTED: DNA topoisomerase 2-beta isoform X2 [Homo sapien	23	87	; regulation of biological process	1.043	1.174	1.207
1578314	Chain A, Structural And Electrophysiological Analysis Of Annexin	15	172		1.248	1.173	1.377
1582568	unnamed protein product [Homo sapiens]	3	7	e response; response to stimulus	0.809	1.173	1.058
5819755	putative transferase CAF17, mitochondrial isoform 1 precursor [Ho	3	9	cell organization and biogenesis	0.834	1.173	1.119
2537232	Chain A, Solution Structure Of Human Sap18	3	4		1.008	1.172	1.350
6208870	ATP-binding cassette, sub-family F, member 1 variant, partial [Hon	5	19	n of biological process; transport	1.075	1.172	1.128
1196036	syntrophin, beta 2 (dystrophin-associated protein A1, 59kDa, basic	10	40		0.955	1.172	1.089
4002611	Chain L, Crystal Structure Of The Tip48 (Tip49b) Hexamer	12	117		1.090	1.170	1.080
1195697	bromodomain and WD repeat domain containing 2, isoform CRA_c	6	15	metabolic process	1.039	1.169	1.008
5788290	PREDICTED: pyruvate dehydrogenase phosphatase regulatory sub	10	19	; regulation of biological process	1.116	1.169	1.320
1611721	Chain B, Phosphorylation Independent Interactions Between 14-3-3	14	204		1.164	1.168	1.165
1195777	cleft lip and palate associated transmembrane protein 1, isoform C	4	14	; regulation of biological process	0.839	1.167	1.038
1196243	mitochondrial carrier homolog 1 (C. elegans), isoform CRA_e [Hom	3	8	cell organization and biogenesis	1.057	1.167	0.897
4003102	unnamed protein product [Homo sapiens]	3	13		0.960	1.167	1.050
6298894	unknown, partial [Homo sapiens]	3	4	metabolic process	0.885	1.167	0.971
2210434	unnamed protein product [Homo sapiens]	2	2	metabolic process	0.907	1.167	1.140
1196156	hypothetical protein MGC10993, isoform CRA_a [Homo sapiens]	2	3		1.041	1.167	1.036
7679142	PREDICTED: proteasome activator complex subunit 4 isoform X5 [	4	10	nd biogenesis; defense response	0.988	1.167	1.014
5304253	PREDICTED: receptor-type tyrosine-protein phosphatase S isoform	4	7	d biogenesis; metabolic process	0.986	1.166	1.065
3240217	serine/threonine-protein phosphatase 5 isoform 2 [Homo sapiens]	3	8	ent movement; defense response	1.064	1.166	1.135
1195895	ClpP caseinolytic peptidase, ATP-dependent, proteolytic subunit h	2	14	cell organization and biogenesis	0.911	1.165	1.097
6355745	ras-related protein Rab-5A isoform 2 [Homo sapiens]	8	41	d biogenesis; metabolic process	1.098	1.165	1.068
7680007	PREDICTED: ribonucleoprotein PTB-binding 1 isoform X1 [Homo s	4	11	metabolic process	1.039	1.165	1.192
7679796	PREDICTED: WD repeat-containing protein 89 isoform X1 [Homo s	2	4		0.918	1.164	1.090
1195949	spectrin, beta, non-erythrocytic 2, isoform CRA_b [Homo sapiens]	19	46	sis; transport; defense response	0.993	1.164	1.186

1195824	NADH dehydrogenase (ubiquinone) 1 alpha subcomplex, 2, 8kDa [	2	13	metabolic process; transport	1.127	1.164	1.094
1195942	oxysterol binding protein [Homo sapiens]	12	40	; regulation of biological process	1.142	1.164	1.043
1196108	proline, glutamic acid and leucine rich protein 1, isoform CRA_c [H	7	19	al process; response to stimulus	0.923	1.164	1.189
1277963	Aldehyde dehydrogenase 7 family, member A1 [Homo sapiens]	10	65	metabolic process	0.988	1.164	1.068
5148296	Chain A, Crystal Structure Of The Otu Domain Of Otulin At 1.3 Ang	2	11		1.194	1.163	1.089
1937835	unnamed protein product [Homo sapiens]	2	7	; regulation of biological process	1.001	1.163	1.136
1459896	unnamed protein product [Homo sapiens]	5	10		0.996	1.162	1.095
7679356	PREDICTED: inositol hexakisphosphate and diphosphoinositol-pe	3	8	metabolic process	0.782	1.162	1.038
3394629	lysophosphatidylcholine acyltransferase 1 [Homo sapiens]	9	42	; regulation of biological process	0.976	1.161	1.099
1196050	glycosyltransferase 25 domain containing 1, isoform CRA_c, partia	10	44	cell organization and biogenesis	1.097	1.161	1.182
1113092	CKAP5 protein [Homo sapiens]	3	7	stimulus; transport; cell division	0.905	1.161	1.017
2220800	TBC1 domain family, member 17 [Homo sapiens]	4	16	; regulation of biological process	1.115	1.161	1.064
7679683	PREDICTED: 1-phosphatidylinositol 4,5-bisphosphate phosphodie	18	78	mmunication; metabolic process	0.983	1.160	1.112
4097579	unnamed protein product, partial [Homo sapiens]	4	9		1.085	1.159	1.030
1196149	mbt domain containing 1, isoform CRA_c [Homo sapiens]	2	3	cell organization and biogenesis	0.980	1.159	1.088
2175624	unnamed protein product [Homo sapiens]	3	7	mmunication; cell differentiation	1.169	1.158	1.247
1337772	GGT7 protein, partial [Homo sapiens]	2	5	metabolic process	1.174	1.158	1.164
7561425	Chain v, Human Constitutive 20s Proteasome In Complex With Car	7	47		1.029	1.157	1.037
3940256	26S proteasome non-ATPase regulatory subunit 5 isoform 2 [Hom	7	49	cell organization and biogenesis	1.148	1.157	1.116
4760577	IDN3-B [Homo sapiens]	2	6	regulation of biological process	1.097	1.157	1.056
6208845	Guanine nucleotide-binding protein G, alpha subunit variant, partia	4	18	esponse to stimulus; coagulation	1.097	1.157	1.100
5303831	PREDICTED: unconventional myosin-VI isoform X6 [Homo sapiens	19	79	gical process; cell differentiation	1.020	1.157	1.135
7679979	PREDICTED: serine/threonine-protein phosphatase 4 regulatory su	2	4	al process; response to stimulus	0.798	1.157	1.134
1196115	laminin, gamma 1 (formerly LAMB2), isoform CRA_a [Homo sapien	3	5	; regulation of biological process	0.799	1.157	1.012
7680186	PREDICTED: RNA-binding protein 39 isoform X1 [Homo sapiens]	7	26	; regulation of biological process	1.082	1.156	1.116
5788039	PREDICTED: condensin complex subunit 2 isoform X2 [Homo sapi	2	4	; metabolic process; cell division	0.823	1.156	1.117
6263023	unknown [Homo sapiens]	9	21	cell organization and biogenesis	0.953	1.156	1.143
762885	Plakoglobin [Homo sapiens]	18	160	al process; response to stimulus	1.043	1.155	1.098
7679948	PREDICTED: alpha-N-acetylglucosaminidase isoform X2 [Homo sa	2	3	biogenesis; response to stimulus	0.803	1.155	1.094
2870379	Similar to expressed sequence AI450757, partial [Homo sapiens]	3	17	metabolic process	1.233	1.155	1.133
3081535	RecName: Full=Putative GTP-binding protein 6; AltName: Full=Pse	4	9	transport	1.091	1.154	1.280
1195991	DEAH (Asp-Glu-Ala-His) box polypeptide 36, isoform CRA_c [Hom	5	7	gical process; defense response	0.981	1.154	1.157
6208825	neurofibromin variant, partial [Homo sapiens]	2	3	sis; defense response; transport	1.143	1.154	0.986
6253106	COMMD2 protein [Homo sapiens]	2	5	; regulation of biological process	0.914	1.154	1.180
1196255	progesterone receptor membrane component 2, isoform CRA_b [H	5	36	al process; response to stimulus	1.086	1.153	1.261
1943862	unnamed protein product [Homo sapiens]	22	189	ell growth; response to stimulus	1.141	1.152	1.100
5788329	PREDICTED: transmembrane emp24 domain-containing protein 1 i	2	11	s; cell communication; transport	0.936	1.152	1.009
1196277	mitochondrial ribosomal protein S15, isoform CRA_c [Homo sapie	4	12	cell organization and biogenesis	1.052	1.152	1.131
1195909	X-ray repair complementing defective repair in Chinese hamster ce	22	90	nse response; cell differentiation	1.061	1.152	1.096
3387473	GSPT1 protein [Homo sapiens]	10	30	cell organization and biogenesis	1.025	1.152	1.124

3746838	38kDa splicing factor [Homo sapiens]	6	14		1.076	1.151	1.035
1196031	Nedd4 binding protein 1 [Homo sapiens]	2	2	al process; response to stimulus	0.866	1.151	1.190
5303930	PREDICTED: sideroflexin-4 isoform X1 [Homo sapiens]	5	8	transport	1.077	1.150	1.183
1195833	malic enzyme 2, NAD(+)-dependent, mitochondrial, isoform CRA_b	12	43	; regulation of biological process	1.079	1.150	1.145
1044199	unknown [Homo sapiens]	2	5	gical process; metabolic process	1.166	1.150	1.133
3118150	unnamed protein product [Homo sapiens]	4	29		1.036	1.150	1.089
1196051	homer homolog 3 (Drosophila), isoform CRA_c [Homo sapiens]	3	6	al process; response to stimulus	1.043	1.149	1.059
5303663	PREDICTED: REST corepressor 3 isoform X4 [Homo sapiens]	2	3	; regulation of biological process	0.991	1.148	1.046
7680249	PREDICTED: myosin-9 isoform X1 [Homo sapiens]	62	534	lus; transport; cell differentiation	1.079	1.147	1.146
1891685	KIAA1974 protein, partial [Homo sapiens]	3	8	metabolic process	0.924	1.146	1.057
2425564	Chain A, Structure Of Human Ist1(Ntd) (Residues 1-189)(P43212)	2	8		1.250	1.146	1.219
1943789	unnamed protein product [Homo sapiens]	5	18	component movement; transport	0.973	1.146	1.257
1943862	unnamed protein product [Homo sapiens]	7	29	metabolic process	0.921	1.145	1.063
5254603	hypothetical protein [Homo sapiens]	5	8	metabolic process	1.135	1.145	1.230
3011714	ATP-dependent RNA helicase DDX3X isoform 3 [Homo sapiens]	16	117	nd biogenesis; defense response	1.061	1.144	1.058
7679423	PREDICTED: midasin isoform X1 [Homo sapiens]	3	6	d biogenesis; metabolic process	0.997	1.144	1.095
1890533	unnamed protein product [Homo sapiens]	4	9	metabolic process	1.001	1.144	1.033
1196161	basic transcription factor 3, isoform CRA_b [Homo sapiens]	3	5	n of biological process; transport	0.835	1.144	1.329
1943864	unnamed protein product [Homo sapiens]	5	20	esponse to stimulus; cell division	1.158	1.144	1.089
3387905	glucose transporter glycoprotein, partial [Homo sapiens]	3	12	; regulation of biological process	1.292	1.144	1.078
1449560	CTP synthase [Homo sapiens]	9	25	oliferation; response to stimulus	1.107	1.143	1.141
1724120	tip associating protein [Homo sapiens]	7	22	transport; metabolic process	0.927	1.143	1.010
1195773	suppressor of Ty 5 homolog (S. cerevisiae), isoform CRA_a [Homo	20	67	velopment; response to stimulus	0.954	1.143	1.209
1195943	PRP19/PSO4 pre-mRNA processing factor 19 homolog (S. cerevisi	10	48	; regulation of biological process	1.190	1.143	1.054
7679529	PREDICTED: cleavage and polyadenylation specificity factor subu	16	46	metabolic process; transport	0.945	1.142	1.087
1890544	unnamed protein product [Homo sapiens]	11	77	sis; metabolic process; transport	1.047	1.142	1.137
1937883	unnamed protein product [Homo sapiens]	4	6	gical process; metabolic process	0.979	1.142	1.032
1196009	chromosome 13 open reading frame 10, isoform CRA_b [Homo sa	3	8	; regulation of biological process	0.818	1.141	1.052
6208855	Transformation/transcription domain-associated protein variant, p	14	34	; regulation of biological process	1.000	1.141	1.126
2645479	MARS2 protein [Homo sapiens]	3	5	metabolic process	0.850	1.141	1.112
5094998	hypothetical protein [Homo sapiens]	24	101	cell organization and biogenesis	1.028	1.141	1.113
460771	hnRNP-E1 [Homo sapiens]	11	118	metabolic process	1.085	1.141	1.101
1196091	leprecan-like 2, isoform CRA_a [Homo sapiens]	4	8	cell organization and biogenesis	0.799	1.140	1.171
1943781	unnamed protein product, partial [Homo sapiens]	2	2	; transport; cellular homeostasis	0.889	1.140	1.126
4885381	histone H1.5 [Homo sapiens]	6	56	cell organization and biogenesis	0.895	1.139	1.245
1582566	unnamed protein product [Homo sapiens]	9	72	to stimulus; cell communication	0.947	1.139	1.121
1890533	unnamed protein product [Homo sapiens]	14	83	; regulation of biological process	0.981	1.138	1.184
1196074	mitochondrial ribosomal protein S28, isoform CRA_c [Homo sapie	2	8	d biogenesis; metabolic process	0.974	1.138	1.082
1985191	CLL-associated antigen KW-12, partial [Homo sapiens]	5	21	sis; metabolic process; transport	1.244	1.137	1.205
1043551	unnamed protein product [Homo sapiens]	4	16	metabolic process	1.008	1.136	1.195

5578039	Chain A, Nmr Structures Of Human Apoptotic Protein Tbid In Lppg	4	12		0.923	1.136	1.236
1196293	RAN binding protein 5, isoform CRA_c [Homo sapiens]	7	16	al process; response to stimulus	1.092	1.135	1.059
1943882	unnamed protein product [Homo sapiens]	3	8	cell organization and biogenesis	0.850	1.135	1.235
7332150	transmembrane emp24 domain-containing protein 4 isoform 5 [Ho	6	29	al process; response to stimulus	1.081	1.134	1.180
1196113	aspartyl-tRNA synthetase 2 (mitochondrial), isoform CRA_b [Homo	17	40	metabolic process	1.215	1.133	1.125
1195915	NADH dehydrogenase (ubiquinone) 1 alpha subcomplex, 10, 42kDa	2	9	ic process; response to stimulus	1.059	1.133	0.940
36100	unnamed protein product [Homo sapiens]	9	50		1.075	1.132	1.265
4758504	3-hydroxyacyl-CoA dehydrogenase type-2 isoform 1 [Homo sapien	10	109	cell organization and biogenesis	1.011	1.132	1.077
3478364	SNW1 protein, partial [Homo sapiens]	5	17	se to stimulus; cell differentiation	0.953	1.132	1.200
1937853	unnamed protein product [Homo sapiens]	18	113	gical process; metabolic process	1.165	1.132	1.135
1196219	VAMP (vesicle-associated membrane protein)-associated protein A	3	8	response to stimulus; cell death	0.811	1.131	1.169
30524	dihydrolipoamide S-acetyltransferase, partial [Homo sapiens]	2	3	; regulation of biological process	0.783	1.131	1.140
1196118	zinc finger CCCH-type containing 11A, isoform CRA_a [Homo sapi	2	2	transport	0.873	1.131	1.176
1196173	SWI/SNF related, matrix associated, actin dependent regulator of c	8	24	d biogenesis; metabolic process	0.898	1.131	1.078
1616601	disintegrin-metalloprotease MADM, partial [Homo sapiens]	4	12	cell organization and biogenesis	1.024	1.130	1.083
1890531	unnamed protein product [Homo sapiens]	8	51	gical process; cell differentiation	0.975	1.130	1.184
1937851	unnamed protein product [Homo sapiens]	10	36	metabolic process	1.097	1.130	1.216
7156450	dedicator of cytokinesis 7, partial [Homo sapiens]	11	26	al process; response to stimulus	0.914	1.130	1.101
7679261	PREDICTED: arf-GAP with coiled-coil, ANK repeat and PH domain-	2	3	al process; response to stimulus	0.972	1.130	1.207
1890666	unnamed protein product [Homo sapiens]	15	127	ic process; response to stimulus	1.142	1.130	1.164
5303896	PREDICTED: leucine-rich repeat-containing protein 14 isoform X3	3	4		1.054	1.129	1.202
1582601	unnamed protein product [Homo sapiens]	4	30	metabolic process; transport	1.048	1.129	0.998
1337556	HP95 [Homo sapiens]	15	40	n of biological process; transport	1.008	1.129	1.148
2210433	unnamed protein product [Homo sapiens]	9	519	cell organization and biogenesis	1.040	1.128	1.067
1196246	exportin 5, isoform CRA_b [Homo sapiens]	4	8	; regulation of biological process	0.894	1.127	1.043
1196071	mitochondrial ribosomal protein L15 [Homo sapiens]	5	23	cell organization and biogenesis	0.942	1.127	1.202
1404346	DNA fragmentation factor, 45kDa, alpha polypeptide [Homo sapien	3	9	; regulation of biological process	1.270	1.127	1.117
1195998	karyopherin alpha 1 (importin alpha 5), isoform CRA_a [Homo sapi	3	9	biogenesis; response to stimulus	1.136	1.125	0.909
1195855	RNA binding motif protein 15B [Homo sapiens]	5	12	ss; metabolic process; transport	0.890	1.125	1.100
7679192	PREDICTED: 28S ribosomal protein S9, mitochondrial isoform X1 [	3	5	biogenesis; response to stimulus	0.895	1.125	1.045
1354407	GPD1L protein, partial [Homo sapiens]	7	28	gical process; metabolic process	1.080	1.125	1.190
7679810	PREDICTED: uncharacterized aarF domain-containing protein kina	2	3	metabolic process	0.795	1.125	1.061
6110490	heat shock protein 90Ad [Homo sapiens]	3	86	ic process; response to stimulus	1.261	1.125	1.065
1196253	PET112-like (yeast), isoform CRA_b [Homo sapiens]	2	4	cell organization and biogenesis	0.814	1.125	1.131
7680545	PREDICTED: vacuolar protein sorting-associated protein 52 homo	8	15	cell differentiation; transport	0.953	1.123	0.986
223582	histone H4	6	242		1.047	1.123	1.151
7679343	PREDICTED: NAD(P) transhydrogenase, mitochondrial isoform X1	8	16	; regulation of biological process	0.893	1.122	0.958
2953220	Chain C, Crystal Structure Of Human Hdac2 Complexed With An N	9	51		1.054	1.122	1.082
7680196	PREDICTED: ES1 protein homolog, mitochondrial isoform X5 [Hom	3	15		1.191	1.122	1.186
5304167	PREDICTED: DNA polymerase delta catalytic subunit isoform X2 [H	13	31	to stimulus; cellular homeostasis	1.011	1.122	1.049

7679363	PREDICTED: probable ATP-dependent RNA helicase YTHDC2 isofo	4	11	; regulation of biological process	1.081	1.121	1.038
4176370	similar to calcium-independent phospholipase A2; similar to AC00	3	6	lic process; cell death; transport	0.910	1.121	0.989
1355917	C447E6.1 (nucleotide binding protein 1 (E.coli MinD like)), partial [I	2	9	d biogenesis; metabolic process	0.901	1.121	1.248
8375494	Chain B, The Crystal Structure Of The Ras Related Protein Rras2 (I	5	17	al process; response to stimulus	0.965	1.120	1.187
1196116	translocated promoter region (to activated MET oncogene), isoforr	24	140	sponse to stimulus; cell division	1.049	1.120	1.091
1582579	unnamed protein product [Homo sapiens]	23	100	biogenesis; response to stimulus	0.974	1.120	1.038
1427820	Chain D, Ran-Rcc1-So4 Complex	5	8		1.048	1.120	1.161
1097346	Myosin, heavy chain 10, non-muscle [Homo sapiens]	36	183	tabolic process; cell proliferation	1.028	1.120	1.124
5788323	PREDICTED: oxysterol-binding protein-related protein 1 isoform X	3	7	transport; metabolic process	0.723	1.120	0.966
1196125	family with sequence similarity 49, member B, isoform CRA_b [Hor	4	15		0.937	1.119	0.992
6730236	Chain L, Crystal Structure Of The D3b Subcomplex Of The Human	6	16	regulation of biological process	1.048	1.119	0.996
1937855	unnamed protein product [Homo sapiens]	3	6	metabolic process	0.911	1.117	1.087
2537230	Chain A, Coupling Of Folding And Binding In The Ptb Domain Of T	3	4		1.080	1.117	0.881
1609485	integrator complex subunit 1 [Homo sapiens]	12	27	; regulation of biological process	1.037	1.117	0.999
1195880	FAD-dependent oxidoreductase domain containing 1, isoform CRA	4	11	d biogenesis; metabolic process	1.096	1.116	1.109
5788396	PREDICTED: elongation factor 1-delta isoform X5 [Homo sapiens]	9	157	al process; response to stimulus	0.993	1.116	1.031
3941724	eukaryotic protein synthesis initiation factor [Homo sapiens]	20	103	; regulation of biological process	0.955	1.115	1.094
7679482	PREDICTED: cAMP-dependent protein kinase type II-beta regulator	2	17	; coagulation; defense response	0.895	1.115	0.910
1195859	hCG14948, isoform CRA_b [Homo sapiens]	2	5		1.051	1.115	1.042
7679173	PREDICTED: aspartyl aminopeptidase isoform X2 [Homo sapiens]	6	11	metabolic process	0.856	1.115	0.998
1943900	unnamed protein product [Homo sapiens]	3	9	; regulation of biological process	0.827	1.115	1.083
7679122	PREDICTED: RNA-binding protein 34 isoform X1 [Homo sapiens]	5	15		0.956	1.114	1.021
1943908	unnamed protein product [Homo sapiens]	16	69	e to stimulus; cell differentiation	0.959	1.114	1.066
1196044	eukaryotic translation initiation factor 3, subunit 4 delta, 44kDa [Ho	8	48	; regulation of biological process	1.052	1.114	1.064
6208919	TNF receptor-associated protein 1 variant, partial [Homo sapiens]	20	195	; regulation of biological process	1.086	1.114	1.107
2175254	unnamed protein product [Homo sapiens]	2	3	transport	0.994	1.114	1.111
1196092	FK506 binding protein 4, 59kDa, isoform CRA_a [Homo sapiens]	19	109	al process; response to stimulus	1.152	1.114	1.167
1196102	fructosamine 3 kinase, isoform CRA_a [Homo sapiens]	5	14	bolic process; cell differentiation	0.945	1.114	1.118
1508050	Coiled-coil domain containing 51 [Homo sapiens]	6	11	metabolic process	0.859	1.114	1.135
1195944	glucosidase, alpha; neutral AB, isoform CRA_d [Homo sapiens]	20	157	metabolic process	1.035	1.113	1.074
1632657	truncated BRE alpha a3+ isoform [Homo sapiens]	2	6	cell organization and biogenesis	0.855	1.113	0.963
1943771	unnamed protein product [Homo sapiens]	8	61	cell organization and biogenesis	1.026	1.112	1.089
5788205	PREDICTED: nuclear pore complex protein Nup98-Nup96 isoform X	11	28	al process; response to stimulus	0.964	1.112	1.168
1890655	unnamed protein product [Homo sapiens]	15	62	bolic process; cell differentiation	0.940	1.112	1.109
1943895	unnamed protein product [Homo sapiens]	4	7	; regulation of biological process	0.918	1.112	1.174
5788179	PREDICTED: E3 ubiquitin-protein ligase LRSAM1 isoform X1 [Hom	2	3	ization and biogenesis; transport	0.920	1.112	0.949
3407802	Chain A, Structural Analysis Of Checkpoint Kinase 2 (Chk2) In Con	2	5	metabolic process	0.958	1.111	1.185
1890536	unnamed protein product [Homo sapiens]	8	45	; cell division; cell differentiation	1.103	1.111	1.137
1893429	DEAD box polypeptide 27 [Homo sapiens]	8	20	metabolic process	0.836	1.111	1.058
1181378	Chain D, Crystal Structure Of A Beta-CateninBCL9TCF4 COMPLEX	20	128		1.087	1.110	0.968

3819747	LMAN2L protein, partial [Homo sapiens]	4	10	metabolic process; transport	1.179	1.110	1.080
1219341	KIF5C protein, partial [Homo sapiens]	10	36	ent; metabolic process; transport	0.998	1.109	1.071
1196109	PRP8 pre-mRNA processing factor 8 homolog (yeast), isoform CRA	63	248	d biogenesis; metabolic process	1.005	1.109	1.102
5094929	hypothetical protein, partial [Homo sapiens]	2	6	d biogenesis; metabolic process	1.000	1.109	1.094
1196270	tetratricopeptide repeat domain 4, isoform CRA_d [Homo sapiens]	2	4		0.829	1.109	1.043
3335093	cytoplasmic dynein 1 heavy chain 1 [Homo sapiens]	88	346	component movement; cell death	1.081	1.109	1.096
1196100	solute carrier family 25 (mitochondrial carrier; dicarboxylate transp	11	70	metabolic process; transport	1.049	1.108	1.050
2910847	pyruvate dehydrogenase E1 component subunit alpha, somatic for	10	59	; regulation of biological process	1.057	1.108	1.023
1196101	THO complex 4, partial [Homo sapiens]	3	20	; transport; response to stimulus	0.950	1.108	1.101
1937859	unnamed protein product [Homo sapiens]	11	56	transport	1.085	1.108	1.087
5803679	hypothetical protein, partial [Homo sapiens]	8	26	; regulation of biological process	0.996	1.108	1.180
4688898	hypothetical protein [Homo sapiens]	3	6	on and biogenesis; development	0.893	1.108	1.113
7679809	PREDICTED: tyrosyl-DNA phosphodiesterase 1 isoform X2 [Homo	4	8	ic process; response to stimulus	1.016	1.107	0.969
1124906	Chain A, Structure Of U2af65 Variant With Polyuridine Tract	3	7		0.863	1.107	1.218
1195774	exosome component 5 [Homo sapiens]	3	9	e response; response to stimulus	0.946	1.107	1.135
7679240	PREDICTED: SWI/SNF complex subunit SMARCC1 isoform X2 [Ho	8	35	al process; response to stimulus	0.998	1.106	1.073
1195706	hCG1994130, isoform CRA_b [Homo sapiens]	8	53	metabolic process	0.918	1.106	1.036
1890652	unnamed protein product [Homo sapiens]	3	43	ization and biogenesis; transport	1.031	1.106	1.125
7680175	PREDICTED: ADP-ribosylation factor GTPase-activating protein 1 i	3	6	cell organization and biogenesis	0.959	1.105	1.109
1943865	unnamed protein product [Homo sapiens]	12	74	cell death; response to stimulus	1.092	1.105	0.914
1582581	unnamed protein product [Homo sapiens]	16	127	metabolic process	1.081	1.104	1.082
4049922	transcription factor WSTF [Homo sapiens]	4	10	; regulation of biological process	0.915	1.104	1.074
2052107	KIAA0539 protein, partial [Homo sapiens]	3	4		1.006	1.104	1.147
1043498	unnamed protein product [Homo sapiens]	3	6	transport	1.008	1.104	1.172
1206603	Mitochondrial ribosomal protein L45 [Homo sapiens]	4	7	d biogenesis; metabolic process	1.059	1.104	1.081
1890542	unnamed protein product [Homo sapiens]	12	53	differentiation; defense response	1.079	1.103	1.146
1943743	unnamed protein product [Homo sapiens]	5	27	al process; response to stimulus	1.154	1.103	1.044
1147813	desmoplakin I [Homo sapiens]	45	206	erentiation; response to stimulus	0.976	1.102	1.085
1943866	unnamed protein product [Homo sapiens]	12	37	; regulation of biological process	0.981	1.102	1.023
1891672	KIAA1942 protein, partial [Homo sapiens]	2	4		1.104	1.102	1.161
1196080	GTPase activating protein and VPS9 domains 1, isoform CRA_c [H	9	20	al process; response to stimulus	0.871	1.102	1.018
5820544	fragile X mental retardation 1 [Homo sapiens]	7	28	n of biological process; transport	0.964	1.102	1.113
1195944	glucosidase, alpha; neutral AB, isoform CRA_a [Homo sapiens]	21	164	metabolic process	1.047	1.102	1.078
7680021	PREDICTED: dnaJ homolog subfamily B member 1 isoform X1 [Ho	3	24	; regulation of biological process	1.072	1.102	1.135
7679780	PREDICTED: zinc finger MYM-type protein 2 isoform X6 [Homo sap	2	3	; regulation of biological process	0.974	1.101	1.164
1943840	unnamed protein product [Homo sapiens]	9	46	; regulation of biological process	1.119	1.101	1.108
1354365	Karyopherin alpha 2 (RAG cohort 1, importin alpha 1) [Homo sapie	6	24	; response to stimulus; transport	1.008	1.101	0.947
1195754	exocyst complex component 2, isoform CRA_a [Homo sapiens]	4	12	; regulation of biological process	0.898	1.100	1.147
1378557	p27BBP protein [Homo sapiens]	4	42	cell organization and biogenesis	1.118	1.100	1.043
7679044	PREDICTED: nardilysin isoform X5 [Homo sapiens]	4	10	; regulation of biological process	0.941	1.100	1.148

1879546	LRRC16A protein [Homo sapiens]	4	4	gical process; metabolic process	0.858	1.099	1.151
6208937	CSDA protein variant, partial [Homo sapiens]	2	17	ic process; response to stimulus	0.879	1.099	1.179
1196276	CAP, adenylate cyclase-associated protein 1 (yeast), isoform CRA_	13	65	esponse to stimulus; coagulation	1.051	1.098	1.043
1196309	hCG2039972, isoform CRA_a [Homo sapiens]	3	6	cell organization and biogenesis	0.689	1.098	1.102
1943753	unnamed protein product [Homo sapiens]	4	9	response to stimulus; cell death	0.817	1.098	1.077
2210422	unnamed protein product [Homo sapiens]	2	5	differentiation; defense response	0.939	1.098	1.335
5254589	hypothetical protein, partial [Homo sapiens]	15	122		0.939	1.098	1.051
4712332	Dystrophin [Homo sapiens]	2	4	ic process; cellular homeostasis	1.148	1.098	1.118
3345733	interferon regulatory factor 2-binding protein-like [Homo sapiens]	4	17	regulation of biological process	1.214	1.098	1.071
3817380	ARNT protein [Homo sapiens]	2	4	bolic process; cell differentiation	0.883	1.098	1.019
1113093	Nuclear transport factor 2-like export factor 2 [Homo sapiens]	4	13	transport	0.951	1.097	0.978
6912586	6-phosphogluconolactonase [Homo sapiens]	8	50	metabolic process	0.995	1.097	1.036
1890654	unnamed protein product [Homo sapiens]	6	15	; regulation of biological process	1.068	1.096	1.142
1196106	Y box binding protein 2, isoform CRA_a [Homo sapiens]	3	21	gical process; metabolic process	0.863	1.096	1.170
5087725	annexin A11 isoform 2 [Homo sapiens]	9	48	ell division; response to stimulus	1.034	1.096	1.128
2601666	zinc phosphodiesterase ELAC protein 2 isoform 3 [Homo sapiens]	6	16	cell organization and biogenesis	0.971	1.096	1.072
1195909	ribosomal protein L37a, isoform CRA_c [Homo sapiens]	4	13	ization and biogenesis; transport	1.031	1.096	1.057
7680045	PREDICTED: ran-binding protein 3 isoform X1 [Homo sapiens]	3	5	transport	1.006	1.096	1.128
1196160	telomeric repeat binding factor 2, interacting protein, isoform CRA	3	5	al process; response to stimulus	1.094	1.096	1.088
7679568	PREDICTED: transmembrane protein 245 isoform X6 [Homo sapien	4	19		0.929	1.096	1.077
1196121	RNA binding motif protein 12B, partial [Homo sapiens]	6	17		0.999	1.095	1.135
1196313	NCK-associated protein 1, isoform CRA_d [Homo sapiens]	15	33	nd biogenesis; defense response	1.049	1.095	1.112
1582585	unnamed protein product [Homo sapiens]	2	4	metabolic process	0.690	1.095	1.050
6282242	unknown [Homo sapiens]	5	18	cell organization and biogenesis	1.081	1.095	1.115
5303677	PREDICTED: HEAT repeat-containing protein 5B isoform X4 [Homo	3	3		1.098	1.095	1.172
1794318	Chain S, Crystal Structure Of Human M-Calpain Form Ii	2	10		1.261	1.095	1.172
5655008	WD repeat and HMG-box DNA-binding protein 1 isoform 2 [Homo s	4	8	cell organization and biogenesis	0.957	1.095	1.113
1196309	mitochondrial ribosomal protein S26 [Homo sapiens]	3	12	ic process; response to stimulus	1.001	1.095	1.138
1592875	Ribosomal protein L15 [Homo sapiens]	4	60	ization and biogenesis; transport	1.045	1.095	1.068
1195733	RAB25, member RAS oncogene family [Homo sapiens]	9	29	al process; response to stimulus	0.902	1.094	1.106
9199294	GNB1 protein, partial [Homo sapiens]	9	78	tion; cell proliferation; cell death	0.947	1.093	1.057
1973049	Chain B, Dj-1 Activation By Catechol Quinone Modification	8	37		1.000	1.093	1.143
7679410	PREDICTED: DNA-directed RNA polymerases I and III subunit RPA	3	9	e response; response to stimulus	0.945	1.093	1.029
1196261	nucleoporin 54kDa, isoform CRA_b [Homo sapiens]	4	9	al process; response to stimulus	1.074	1.092	1.084
1196066	heat shock 70kDa protein 14, isoform CRA_a [Homo sapiens]	6	16	d biogenesis; metabolic process	0.889	1.092	1.129
1196148	clathrin, heavy polypeptide (Hc), isoform CRA_d [Homo sapiens]	48	436	; regulation of biological process	1.133	1.092	1.054
1196045	interleukin enhancer binding factor 3, 90kDa, isoform CRA_d [Hom	14	102	e response; response to stimulus	1.066	1.092	0.998
1680250	interferon-stimulated gene factor 3 alpha 113 kda protein; ISGF3 al	2	4		0.822	1.091	1.209
4593585	Chain A, The Wild-type Src Homology 2 (sh2)-domain Containing P	6	30		1.017	1.091	1.027
1195826	DEAD (Asp-Glu-Ala-Asp) box polypeptide 46, isoform CRA_d [Hom	21	73	; regulation of biological process	1.073	1.091	1.063

1196160	hCG27698, isoform CRA_c [Homo sapiens]	3	6	al process; response to stimulus	0.955	1.091	1.049
1196069	BMS1-like, ribosome assembly protein (yeast), isoform CRA_c [Ho	3	6	cell organization and biogenesis	0.908	1.091	1.179
5567127	NADH dehydrogenase subunit 5 (mitochondrion) [Homo sapiens]	2	4	transport; metabolic process	1.099	1.090	0.896
1890691	unnamed protein product [Homo sapiens]	23	207	; regulation of biological process	1.116	1.090	1.025
7680020	PREDICTED: E3 ubiquitin-protein ligase UHRF1 isoform X1 [Homo	3	8	cell organization and biogenesis	0.925	1.090	1.040
1196149	sperm associated antigen 9, isoform CRA_b [Homo sapiens]	5	10	cell organization and biogenesis	1.100	1.090	0.985
7679020	PREDICTED: kinesin-like protein KIF2C isoform X1 [Homo sapiens	4	19	ss; cell proliferation; cell division	1.000	1.090	1.073
1890655	unnamed protein product [Homo sapiens]	16	130	s; cell communication; transport	1.039	1.089	1.108
1195772	ribosomal protein S16, isoform CRA_b [Homo sapiens]	11	53	ization and biogenesis; transport	1.031	1.089	1.149
1043384	unnamed protein product [Homo sapiens]	6	21	on; transport; metabolic process	0.978	1.089	1.049
6094121	Chain B, Crystal Stucture Of The Catalytic Domain Of Selenomethi	2	2		1.075	1.089	0.986
1943782	unnamed protein product [Homo sapiens]	4	18	of biological process; cell death	1.163	1.088	1.031
1943755	unnamed protein product [Homo sapiens]	7	16	metabolic process	1.096	1.088	0.971
3718335	C3ORF4 [Homo sapiens]	2	5		1.011	1.088	1.141
1196279	histone deacetylase 1, isoform CRA_a [Homo sapiens]	8	42	; coagulation; cell differentiation	1.032	1.087	1.037
1196109	methyltransferase 10 domain containing, isoform CRA_a [Homo sa	2	3	metabolic process	0.986	1.086	1.047
1196151	CDK5 regulatory subunit associated protein 3, isoform CRA_c [Ho	2	4	d biogenesis; metabolic process	1.125	1.085	1.127
2699681	UDP-glucose ceramide glucosyltransferase-like 1 [Homo sapiens]	17	37	metabolic process	1.063	1.085	1.053
5788076	PREDICTED: guanine nucleotide-binding protein subunit beta-4 is	6	55	response to stimulus; cell death	0.897	1.085	1.048
1195803	MORC family CW-type zinc finger 2, isoform CRA_b, partial [Homo	6	11	metabolic process	0.859	1.084	1.128
7679221	PREDICTED: ubiquitin-like modifier-activating enzyme ATG7 isofo	2	5	nd biogenesis; defense response	0.872	1.084	1.126
1943744	unnamed protein product [Homo sapiens]	4	17		0.968	1.084	1.662
1808810	General transcription factor IIIC, polypeptide 2, beta 110kDa [Homo	3	6	metabolic process	0.882	1.084	1.073
1890549	unnamed protein product [Homo sapiens]	9	113	cell organization and biogenesis	0.991	1.084	1.048
7679885	PREDICTED: leucine carboxyl methyltransferase 1 isoform X3 [Hon	2	6	; regulation of biological process	1.140	1.084	1.093
36036	GTPase [Homo sapiens]	2	6	cell organization and biogenesis	1.044	1.083	1.113
7680084	PREDICTED: ER membrane protein complex subunit 10 isoform X4	3	11		1.030	1.083	1.153
1196080	ribosomal protein L12, isoform CRA_a [Homo sapiens]	6	49	sis; metabolic process; transport	1.094	1.083	0.987
1890531	unnamed protein product [Homo sapiens]	7	47	ization and biogenesis; transport	1.117	1.083	1.146
3740743	Chain A, Crystal Structure Of 14-3-3 Epsilon With Mlf1 Peptide	15	279		1.161	1.083	1.098
5303618	PREDICTED: carnitine O-palmitoyltransferase 2, mitochondrial iso	7	20	metabolic process; transport	0.896	1.083	1.020
1591645	Chain B, The Structure Of The Cyclin T-Binding Domain Of Hexim1	3	3		1.171	1.083	0.939
3474475	Chain G, Caspase-6 In Complex With Z-Vad-Fmk Inhibitor	3	5	metabolic process	0.805	1.083	1.019
5303765	PREDICTED: glucosamine-6-phosphate isomerase 2 isoform X2 [H	4	9	metabolic process	1.369	1.083	1.039
9272623	Chain C, Truncated Ran Wild Type In Complex With Gdp-bef And R	9	116		1.114	1.082	1.013
1943836	unnamed protein product [Homo sapiens]	4	22	cell organization and biogenesis	0.961	1.082	0.959
5788372	PREDICTED: DNA replication licensing factor MCM5 isoform X1 [H	14	73	metabolic process	1.024	1.082	1.056
5020253	repressor of estrogen receptor activity [Homo sapiens]	21	149	cell organization and biogenesis	1.104	1.081	1.105
7679617	PREDICTED: vesicle transport through interaction with t-SNAREs	2	2	cell organization and biogenesis	1.048	1.081	1.080
1044048	FLJ00084 protein, partial [Homo sapiens]	4	7	al process; response to stimulus	1.094	1.081	1.091

1343646	CPSF6 protein [Homo sapiens]	3	16	cell organization and biogenesis	0.851	1.080	1.009
4877759	MAGE tumor antigen D1 [Homo sapiens]	4	6	cell death; response to stimulus	1.203	1.080	1.110
7680246	PREDICTED: tRNA (uracil-5-)-methyltransferase homolog A isoform	2	6	metabolic process	0.688	1.080	0.954
1196037	synaptophysin-like 1, isoform CRA_b [Homo sapiens]	2	10	transport; cell communication	1.041	1.080	0.985
1143067	HSR1 protein [Homo sapiens]	7	24	n of biological process; transport	0.874	1.080	1.095
7810174	Chain A, 14-3-3 Protein Beta (human) In Complex With Exoenzyme	10	153		0.960	1.080	1.011
4758668	serine palmitoyltransferase 2 [Homo sapiens]	9	21	metabolic process	1.004	1.079	1.094
1195749	vinculin, isoform CRA_c [Homo sapiens]	42	210	of biological process; cell growth	0.949	1.078	1.134
2737057	MTA2 protein, partial [Homo sapiens]	19	72	cell organization and biogenesis	0.964	1.078	1.081
1195748	procollagen-proline, 2-oxoglutarate 4-dioxygenase (proline 4-hydro	3	19	cell organization and biogenesis	0.997	1.078	1.139
7662078	tetratricopeptide repeat protein 37 [Homo sapiens]	7	13		0.879	1.078	0.996
1575536	regulator of nonsense transcript stability [Homo sapiens]	23	78	; regulation of biological process	1.012	1.078	1.049
1215118	NF-kappaB repressing factor, partial [Homo sapiens]	9	22	; regulation of biological process	1.027	1.077	1.133
1341045	Chain A, Structure Of Human Asf1a In Complex With Histone H3	2	2	cell organization and biogenesis	0.781	1.077	1.049
1582596	unnamed protein product [Homo sapiens]	2	5	al process; response to stimulus	0.837	1.076	1.073
3859996	SNARE protein Ykt6, partial [Homo sapiens]	8	28	d biogenesis; metabolic process	1.173	1.076	1.095
4825729	COPG2 protein, partial [Homo sapiens]	11	40	transport	1.063	1.076	0.994
7705805	28S ribosomal protein S2, mitochondrial [Homo sapiens]	5	11	cell organization and biogenesis	1.027	1.075	1.002
5304294	PREDICTED: flotillin-1 isoform X2 [Homo sapiens]	5	12	; transport; response to stimulus	0.956	1.075	1.127
7679680	PREDICTED: phosphatidylinositol 3,4,5-trisphosphate 5-phosphata	11	30	al process; response to stimulus	0.961	1.075	1.040
1337771	TJP3 protein, partial [Homo sapiens]	2	4	regulation of biological process	0.815	1.075	1.054
5552899	3'(2'),5'-bisphosphate nucleotidase 1 isoform 3 [Homo sapiens]	8	38	metabolic process	1.213	1.074	1.040
1196108	phospholipase D2, isoform CRA_b [Homo sapiens]	5	9	e; cellular component movement	0.941	1.074	0.958
4945696	YKT6, partial [Homo sapiens]	8	29	d biogenesis; metabolic process	1.171	1.074	1.085
2195206	SART3 protein [Homo sapiens]	11	31	logical process; cell proliferation	1.148	1.074	1.172
5303945	PREDICTED: ATPase family AAA domain-containing protein 1 isofo	2	6	cell organization and biogenesis	1.098	1.074	1.107
1195764	poly(A) binding protein interacting protein 1, isoform CRA_a [Hom	3	8	; regulation of biological process	0.950	1.074	1.126
7679802	PREDICTED: E3 ubiquitin-protein ligase HECTD1 isoform X4 [Hom	6	13	; regulation of biological process	0.951	1.074	1.105
339516	transferrin receptor [Homo sapiens]	28	115	nse response; cell differentiation	1.026	1.073	1.063
5440633	ubiquitin carboxyl-terminal hydrolase 47 isoform a [Homo sapiens]	6	21	; regulation of biological process	0.930	1.073	1.135
1196279	eukaryotic translation initiation factor 3, subunit 2 beta, 36kDa, iso	7	51	; regulation of biological process	1.062	1.073	0.973
3339442	Chain A, Crystal Structure Of The Tpr Domain Of Kinesin Light Cha	2	9		0.978	1.072	1.015
7679922	PREDICTED: derlin-2 isoform X3 [Homo sapiens]	2	6	n of biological process; transport	1.026	1.072	0.992
1196275	leucine proline-enriched proteoglycan (leprecan) 1, isoform CRA_c	6	16	cell organization and biogenesis	1.154	1.072	0.967
1527724	small nuclear ribonuclear protein D homolog [Homo sapiens]	3	14	metabolic process	1.188	1.072	1.218
1195888	male sterility domain containing 2, isoform CRA_b [Homo sapiens]	10	37	metabolic process	1.111	1.072	1.048
1943764	unnamed protein product [Homo sapiens]	4	10	al process; response to stimulus	1.032	1.071	0.995
1513140	dJ85M6.1 (cleavage stimulation factor, 3' pre-RNA, subunit 3, 77kD	7	21	metabolic process	0.980	1.071	1.108
7679808	PREDICTED: exonuclease 3'-5' domain-containing protein 2 isofor	2	4	metabolic process	0.722	1.071	1.033
1890679	unnamed protein product [Homo sapiens]	2	8	metabolic process; transport	1.110	1.071	1.083

2880091	unnamed protein product [Homo sapiens]	8	19		0.850	1.071	1.051
3723148	USP39 protein, partial [Homo sapiens]	8	31	; metabolic process; cell division	1.004	1.070	1.049
1044138	TPM4-ALK fusion oncoprotein type 2, partial [Homo sapiens]	9	68	n; cellular component movement	1.222	1.069	1.179
1196137	glutamyl-prolyl-tRNA synthetase, isoform CRA_a [Homo sapiens]	26	106	al process; response to stimulus	1.027	1.069	1.103
5303766	PREDICTED: LIM and calponin homology domains-containing prot	3	7	cell organization and biogenesis	0.869	1.069	1.043
1195728	transient receptor potential cation channel, subfamily M, member 4	11	35	ss; metabolic process; transport	0.988	1.069	1.017
1890653	unnamed protein product [Homo sapiens]	29	235	; regulation of biological process	1.178	1.068	1.025
1063647	GTP-binding protein RAB22A [Homo sapiens]	2	4	al process; response to stimulus	0.926	1.068	0.964
7679923	PREDICTED: rabankyrin-5 isoform X4 [Homo sapiens]	6	10	; regulation of biological process	1.116	1.068	1.038
4097700	unnamed protein product, partial [Homo sapiens]	2	5		1.392	1.067	1.065
5123932	fragile X mental retardation 1 [Homo sapiens]	7	27	n of biological process; transport	0.956	1.066	1.110
2210456	unnamed protein product [Homo sapiens]	3	6	n; coagulation; defense response	1.182	1.066	1.019
6242117	actin-like protein, partial [Homo sapiens]	3	120	nd biogenesis; defense response	1.922	1.066	0.927
1196321	hypoxanthine phosphoribosyltransferase 1 (Lesch-Nyhan syndrom	3	25	cell organization and biogenesis	1.063	1.065	0.986
1584281	Chain A, Crystal Structure Of Human Lsd1 At 2.3 A Resolution	9	26	metabolic process	1.012	1.065	1.021
1582555	unnamed protein product [Homo sapiens]	5	13	se to stimulus; defense response	1.077	1.065	1.115
4004214	unnamed protein product, partial [Homo sapiens]	3	8		1.126	1.065	0.983
1196270	mitochondrial ribosomal protein L37, isoform CRA_a, partial [Hom	5	15	cell organization and biogenesis	1.019	1.064	1.044
1890533	unnamed protein product [Homo sapiens]	3	38	ess; transport; cell differentiation	1.388	1.064	1.151
2964392	RecName: Full=Nuclear pore complex protein Nup205; AltName: F	21	50	al process; response to stimulus	1.009	1.064	1.080
2982289	autophagy-related protein 16-1 isoform 3 [Homo sapiens]	2	3	ic process; response to stimulus	0.873	1.063	0.887
7679347	PREDICTED: zinc finger protein 622 isoform X1 [Homo sapiens]	2	9	al process; response to stimulus	0.900	1.063	0.899
3058368	Rho guanine nucleotide exchange factor (GEF) 1 [Homo sapiens]	10	36	nse to stimulus; cell proliferation	0.979	1.063	1.012
1196214	kinase D-interacting substance of 220 kDa, isoform CRA_c [Homo	5	9	cell organization and biogenesis	0.958	1.063	1.129
5788039	PREDICTED: CLIP-associating protein 1 isoform X16 [Homo sapie	7	17	mponent movement; cell division	0.860	1.063	1.064
4147424	unknown, partial [Homo sapiens]	7	19	cell differentiation	1.037	1.062	0.991
7679688	PREDICTED: RNA-binding protein 4B isoform X1 [Homo sapiens]	8	35	al process; response to stimulus	0.980	1.062	1.043
7679277	PREDICTED: fragile X mental retardation syndrome-related protein	10	35	gical process; cell differentiation	0.973	1.061	1.110
2737057	PTPLAD1 protein, partial [Homo sapiens]	6	31	e to stimulus; metabolic process	1.170	1.061	1.054
1552242	hRlf beta subunit (p102 protein) [Homo sapiens]	24	103	metabolic process	1.024	1.061	1.046
8819253	Chain B, Structure Of Human Mitochondrial Malonyltransferase	4	9		0.867	1.061	1.083
7679750	PREDICTED: 60S ribosomal protein L6 isoform X2 [Homo sapiens]	11	94	n of biological process; transport	0.973	1.061	1.076
1196091	nucleolar protein 1, 120kDa [Homo sapiens]	6	21	; regulation of biological process	1.033	1.060	1.072
1196283	sulfatase modifying factor 2, isoform CRA_j [Homo sapiens]	4	18	metabolic process	0.881	1.060	0.866
1937877	unnamed protein product [Homo sapiens]	7	26	; regulation of biological process	0.884	1.060	1.032
1195901	poly (ADP-ribose) polymerase family, member 1, isoform CRA_a [H	22	115	se to stimulus; cell differentiation	1.103	1.060	1.143
7679532	PREDICTED: exosome complex component RRP41 isoform X2 [Ho	3	6	e response; response to stimulus	1.078	1.060	1.028
7105129	BOP1 protein, partial [Homo sapiens]	7	21	; regulation of biological process	0.956	1.060	0.936
2079931	Tho2 [Homo sapiens]	7	18	metabolic process; transport	1.129	1.060	1.138
1196070	protein kinase, DNA-activated, catalytic polypeptide, isoform CRA_	88	439	on; cell death; defense response	1.055	1.060	1.012

7625739	ubiquitin carboxyl-terminal hydrolase 48 isoform b [Homo sapiens]	2	4	; regulation of biological process	0.976	1.059	0.988
7328088	hypothetical protein, partial [Homo sapiens]	3	12	cell organization and biogenesis	0.894	1.059	1.073
2210428	unnamed protein product, partial [Homo sapiens]	4	7	cell organization and biogenesis	0.922	1.058	1.058
5094937	hypothetical protein, partial [Homo sapiens]	2	5	regulation of biological process	0.973	1.058	0.971
1195917	activating signal cointegrator 1 complex subunit 3-like 1, isoform C	41	195	bolic process; cell differentiation	1.111	1.058	1.038
7679834	PREDICTED: codanin-1 isoform X5 [Homo sapiens]	3	5	; regulation of biological process	1.174	1.057	1.231
7679791	PREDICTED: gamma-tubulin complex component 3 isoform X2 [Ho	4	7	; regulation of biological process	0.943	1.057	1.011
7513681	tyrosine-protein phosphatase non-receptor type 23 isoform 2 [Hom	11	26	cell organization and biogenesis	0.955	1.057	1.047
7679848	PREDICTED: vacuolar protein sorting-associated protein 18 homo	3	6	; regulation of biological process	0.940	1.057	1.092
6366664	Chain B, Steroid Receptor Rna Activator (sra) Modification By The	6	22		0.979	1.057	1.051
4530561	Chain A, Molecular Tweezers Modulate 14-3-3 Protein-protein Inter	6	82		0.938	1.057	1.004
2896140	transcriptional coactivator ALY, partial [Homo sapiens]	3	10	; transport; response to stimulus	0.988	1.056	1.236
2837337	Chain B, New Crystal Form Of Human Cd81 Large Extracellular Lo	3	13		1.068	1.056	1.079
1943806	unnamed protein product [Homo sapiens]	4	16	; regulation of biological process	1.070	1.056	0.851
6638511	deoxyribose-phosphate aldolase isoform 2 [Homo sapiens]	2	2	metabolic process	0.927	1.056	1.074
1196034	amyloid beta precursor protein binding protein 1, isoform CRA_b [	5	11	us; metabolic process; cell death	1.096	1.056	0.963
4405760	alternative protein SLC35A4 [Homo sapiens]	2	4	transport	1.184	1.056	1.151
8957402	mitochondrial ATP synthase, H+ transporting F1 complex beta sub	16	424	biogenesis; cellular homeostasis	1.172	1.055	0.995
7680077	PREDICTED: HAUS augmin-like complex subunit 5 isoform X2 [Ho	2	3	tion and biogenesis; cell division	0.944	1.055	0.941
7680244	PREDICTED: SUN domain-containing protein 2 isoform X1 [Homo s	8	31	s; cellular component movement	1.070	1.055	0.989
1195962	phosphatidylinositol glycan, class T, isoform CRA_d [Homo sapien	7	24	ss; cell differentiation; cell death	1.014	1.055	0.944
1195777	optic atrophy 3 (autosomal recessive, with chorea and spastic para	3	7	cell organization and biogenesis	1.002	1.055	1.055
1196044	hCG2033702, isoform CRA_b [Homo sapiens]	5	8		1.028	1.054	0.972
1196288	ribosomal protein L21, isoform CRA_d, partial [Homo sapiens]	7	28	ization and biogenesis; transport	0.870	1.054	0.971
1502992	RNH1 protein, partial [Homo sapiens]	11	84	; regulation of biological process	1.085	1.054	1.027
2098705	Chain B, Crystal Structure Of The Mammalian Copii-coat Protein S	10	32	transport	0.913	1.054	1.015
6942701	Chain A, Leopard Syndrome-associated Shp2/q510e Mutant	6	19		1.010	1.053	1.040
5304149	PREDICTED: coiled-coil and C2 domain-containing protein 1A isof	7	18	ic process; response to stimulus	1.014	1.053	0.938
2430803	E3 UFM1-protein ligase 1 [Homo sapiens]	5	14	e to stimulus; metabolic process	0.961	1.053	1.020
7561425	Chain r, Human Constitutive 20s Proteasome In Complex With Car	5	14	metabolic process	0.939	1.053	0.939
1195817	tubulin, gamma complex associated protein 2, isoform CRA_a, par	3	8	; regulation of biological process	0.916	1.052	1.018
2173932	hypothetical protein, partial [Homo sapiens]	2	3		0.778	1.052	1.065
1597950	Chain B, Crystal Structure Of Human Mitochondrial Tyrosyl-trna Sy	5	11	metabolic process	0.952	1.052	1.038
1196245	chromosome 6 open reading frame 108, isoform CRA_b [Homo sap	4	13	gical process; cell differentiation	0.993	1.052	1.253
3553331	Chain B, Crystal Structure Of Human Mitochondrial Transcription F	2	5		1.292	1.052	1.108
2769254	NIPSNAP2 protein [Homo sapiens]	4	16	; regulation of biological process	0.828	1.051	0.987
1894588	transferrin receptor protein 1 isoform 1 [Homo sapiens]	28	114	nse response; cell differentiation	1.021	1.051	1.038
1943823	unnamed protein product [Homo sapiens]	3	14	d biogenesis; metabolic process	1.110	1.051	1.065
5288810	serine hydroxymethyltransferase, cytosolic isoform 3 [Homo sapie	4	10	cell organization and biogenesis	0.947	1.051	1.031
1196025	hCG1993037, isoform CRA_f [Homo sapiens]	3	5		1.061	1.051	0.961

3248192	kinesin heavy chain member 2 [Homo sapiens]	6	16	; cell differentiation; cell division	1.021	1.051	1.051
1521542	Valyl-tRNA synthetase [Homo sapiens]	30	155	; regulation of biological process	1.038	1.050	1.029
3258631	GDP-D-mannose-4,6-dehydratase [Homo sapiens]	6	19	e to stimulus; metabolic process	0.971	1.050	1.042
1943772	unnamed protein product [Homo sapiens]	2	4	metabolic process	1.050	1.050	1.076
7679957	PREDICTED: DNA topoisomerase 2-alpha isoform X2 [Homo sapie	20	68	; regulation of biological process	1.003	1.050	1.084
2804273	alpha actinin 4 [Homo sapiens]	27	237	cell organization and biogenesis	1.079	1.050	1.108
7679746	PREDICTED: 5'-nucleotidase domain-containing protein 3 isoform	2	2	metabolic process	1.285	1.050	1.034
1196061	CTF18, chromosome transmission fidelity factor 18 homolog (S. ce	4	7	metabolic process	0.823	1.049	1.132
1195855	poly(rC) binding protein 4, isoform CRA_f [Homo sapiens]	2	3		1.085	1.049	1.210
6523835	hypothalamus protein HT002 [Homo sapiens]	2	5	; regulation of biological process	0.931	1.049	1.050
8456994	PURA protein, partial [Homo sapiens]	2	2	gical process; cell differentiation	1.092	1.049	1.194
2172728	polyribonucleotide 5'-hydroxyl-kinase Clp1 isoform 2 [Homo sapie	3	5	cell organization and biogenesis	1.054	1.049	1.039
7679956	PREDICTED: N-sulphoglucosamine sulphohydrolase isoform X4 [H	2	10	metabolic process	0.934	1.049	0.990
1196245	PTK7 protein tyrosine kinase 7, isoform CRA_b [Homo sapiens]	8	15	nd biogenesis; cell differentiation	0.983	1.049	1.000
1196069	GTP binding protein 4, isoform CRA_b [Homo sapiens]	10	23	; response to stimulus; transport	0.998	1.048	0.980
1438062	PWP2 [Homo sapiens]	10	25	d biogenesis; metabolic process	1.085	1.048	0.993
2646814	inner nuclear membrane protein Man1 isoform 2 [Homo sapiens]	3	6	gical process; cell differentiation	1.084	1.048	1.110
4767866	SCA10 [Homo sapiens]	5	13	cell organization and biogenesis	1.067	1.048	1.081
6648059	protein YIF1A isoform 2 [Homo sapiens]	2	3	e to stimulus; metabolic process	1.159	1.048	0.886
1195696	BUB3 budding uninhibited by benzimidazoles 3 homolog (yeast), is	7	39	; metabolic process; cell division	1.059	1.047	1.043
1196100	raptor, isoform CRA_b [Homo sapiens]	3	7	cell growth; cell communication	1.077	1.047	1.043
1196010	pleckstrin homology domain containing, family C (with FERM dom	4	8	cell organization and biogenesis	1.228	1.046	1.101
177783	Ro ribonucleoprotein [Homo sapiens]	10	34	cell organization and biogenesis	1.104	1.046	1.090
1943861	unnamed protein product [Homo sapiens]	2	3	regulation of biological process	1.016	1.046	0.981
5882928	Chain A, Crystal Structure Of Human 5t4 (wnt-activated Inhibitory F	6	24		0.937	1.046	1.074
1196230	transmembrane protein 11, isoform CRA_a [Homo sapiens]	4	11	cell organization and biogenesis	0.984	1.046	1.104
1943833	unnamed protein product [Homo sapiens]	3	27	; coagulation; defense response	1.326	1.046	1.362
1196115	DEAH (Asp-Glu-Ala-His) box polypeptide 9, isoform CRA_b [Homo	37	274	se to stimulus; defense response	1.061	1.046	1.035
1196123	eukaryotic translation initiation factor 3, subunit 3 gamma, 40kDa,	7	13	; regulation of biological process	1.152	1.046	1.131
2537226	Chain A, Crystal Structure Of Sr Protein Kinase 1 Complexed To Its	3	9	metabolic process	1.046	1.045	0.985
1483424	RAB11B protein [Homo sapiens]	12	81	al process; response to stimulus	0.977	1.045	1.085
1943769	unnamed protein product [Homo sapiens]	7	46	; regulation of biological process	1.146	1.045	1.100
5303692	PREDICTED: pre-mRNA 3' end processing protein WDR33 isoform	2	5	ic process; response to stimulus	0.884	1.045	1.102
1943881	unnamed protein product [Homo sapiens]	2	2	; regulation of biological process	0.898	1.045	1.004
1196096	hCG1789827 [Homo sapiens]	2	18	metabolic process	1.137	1.045	0.895
337518	ribosomal protein, partial [Homo sapiens]	6	39	ization and biogenesis; transport	0.905	1.045	1.042
5303669	PREDICTED: isoamyl acetate-hydrolyzing esterase 1 homolog isofo	4	12	metabolic process	0.876	1.045	1.044
5303626	PREDICTED: putative helicase MOV-10 isoform X2 [Homo sapiens]	10	26	se to stimulus; defense response	0.960	1.045	1.008
1196123	RAD21 homolog (S. pombe), isoform CRA_b [Homo sapiens]	4	11	tion and biogenesis; cell division	0.947	1.044	1.052
1377519	SH3 domain-binding glutamic acid-rich-like protein 3 [Homo sapie	3	27	homeostasis; metabolic process	1.188	1.044	1.072

1943744	unnamed protein product [Homo sapiens]	3	10	ic process; response to stimulus	1.088	1.044	0.891
1195911	DnaJ (Hsp40) homolog, subfamily B, member 2, isoform CRA_b [H	5	23	; regulation of biological process	0.902	1.044	0.938
1943769	unnamed protein product [Homo sapiens]	8	24	cell organization and biogenesis	1.045	1.043	1.085
4032340	eukaryotic translation initiation factor 3 [Homo sapiens]	27	121	; regulation of biological process	1.018	1.043	1.008
1893395	Chain B, Crystal Structure Of 2-Enoyl Thioester Reductase Of Hum	2	4	metabolic process	0.956	1.042	1.080
1312351	mitochondrial ribosomal protein-L5 heart-specific splice-variant [H	2	6	d biogenesis; metabolic process	0.805	1.042	0.950
1196154	lysophospholipase II, isoform CRA_d [Homo sapiens]	2	10	metabolic process	0.805	1.042	0.887
5131374	Chain B, Human Cohesin Inhibitor Wapl	2	2		0.815	1.042	0.993
1195741	heterogeneous nuclear ribonucleoprotein H1 (H), isoform CRA_a [	10	238	; regulation of biological process	1.092	1.042	1.084
1147945	Chain F, The Crystal Structure Of Human Enoyl-Coenzyme A (Coa)	5	32	metabolic process	0.884	1.042	1.032
1582565	unnamed protein product [Homo sapiens]	11	52	metabolic process	1.085	1.041	0.990
1195778	glucocorticoid receptor DNA binding factor 1, isoform CRA_d [Hor	3	6	component movement; transport	1.080	1.041	1.215
1196275	phosphopantothenoylcysteine synthetase [Homo sapiens]	9	27	metabolic process	0.974	1.041	1.038
1195979	hCG1786469 [Homo sapiens]	4	123	ic process; response to stimulus	1.402	1.041	1.215
1195839	integrator complex subunit 9, isoform CRA_a [Homo sapiens]	2	3	metabolic process	1.123	1.040	1.160
1943758	unnamed protein product [Homo sapiens]	11	64	s; cellular component movement	1.092	1.040	1.001
1196172	within bgcn homolog (Drosophila), isoform CRA_b, partial [Homo s	3	6	cell organization and biogenesis	0.980	1.040	1.054
5279040	PUS7 protein, partial [Homo sapiens]	2	5	metabolic process	1.019	1.040	0.960
5303968	PREDICTED: NADH dehydrogenase [ubiquinone] iron-sulfur protei	3	15	cell organization and biogenesis	0.884	1.039	1.071
5303648	PREDICTED: nitrilase homolog 1 isoform X1 [Homo sapiens]	4	8	metabolic process	1.090	1.039	1.045
2946607	abhydrolase domain-containing protein 16A isoform b [Homo sapi	6	11	metabolic process	1.115	1.039	1.110
5326860	phospholipase A2 activating protein [Homo sapiens]	9	15	; regulation of biological process	1.027	1.039	0.986
1196047	hypothetical protein MGC3207, isoform CRA_b [Homo sapiens]	9	42	metabolic process	1.051	1.038	1.106
1134073	unnamed protein product [Homo sapiens]	6	19		1.072	1.038	1.055
5788001	PREDICTED: cyclin-dependent kinase 11B isoform X7 [Homo sapie	3	6	and biogenesis; cell proliferation	1.092	1.038	1.098
6298890	unknown, partial [Homo sapiens]	39	193	; transport; response to stimulus	1.109	1.038	1.052
7679395	PREDICTED: ankyrin repeat and SAM domain-containing protein 1	2	3	al process; response to stimulus	0.918	1.038	1.127
1193893	Chain A, Ct10-Regulated Kinase Isoform Ii	5	13		1.077	1.038	1.099
7376040	thymopoietin isoform beta [Homo sapiens]	9	69	regulation of biological process	0.863	1.038	1.058
1820465	Calcium homeostasis endoplasmic reticulum protein [Homo sapie	2	12	n of biological process; transport	0.977	1.038	1.111
3811487	RPL13A protein, partial [Homo sapiens]	6	55	al process; response to stimulus	1.035	1.038	0.954
5304007	PREDICTED: thymopoietin isoform X2 [Homo sapiens]	8	65	regulation of biological process	0.863	1.038	1.012
7679869	PREDICTED: CREB-binding protein isoform X5 [Homo sapiens]	6	13	l proliferation; defense response	1.217	1.037	1.196
1196104	syntaxin 8, isoform CRA_b, partial [Homo sapiens]	3	5	; regulation of biological process	1.045	1.037	0.941
298373	phenylalanine hydroxylase-stimulating protein, pterin-4 alpha-carb	3	7	metabolic process	0.759	1.037	0.937
1195717	hypothetical protein FLJ13611, isoform CRA_d [Homo sapiens]	2	5		1.045	1.037	1.160
7679136	PREDICTED: T-complex protein 1 subunit eta isoform X1 [Homo sa	16	147	metabolic process; transport	1.118	1.036	1.046
1582597	unnamed protein product [Homo sapiens]	10	104	e to stimulus; metabolic process	1.045	1.036	1.065
4837654	RPS4X protein, partial [Homo sapiens]	11	91	; regulation of biological process	1.021	1.036	0.966
1710279	dihyrolipoamide acetyl transferase, partial [Homo sapiens]	2	10	; regulation of biological process	1.030	1.036	0.968

7679466	PREDICTED: protein FAM3C isoform X1 [Homo sapiens]	4	18	cell organization and biogenesis	0.908	1.036	1.008
3819707	SLC25A25 protein, partial [Homo sapiens]	4	7	us; metabolic process; transport	1.047	1.035	1.015
6094124	Chain D, Crystal Structure Of Human Lnkh2b-h2a.z-anp32e	11	311		1.176	1.035	1.023
1943848	unnamed protein product [Homo sapiens]	6	32	metabolic process	1.275	1.034	1.022
1101029	pyruvate carboxylase precursor [Homo sapiens]	17	49	; regulation of biological process	1.012	1.034	0.967
3820171	probable ATP-dependent RNA helicase DDX17 isoform 1 [Homo sa	22	169	; regulation of biological process	1.130	1.034	0.993
3104443	LEM domain-containing protein 2 isoform 1 [Homo sapiens]	4	13	; regulation of biological process	0.864	1.034	1.048
5147689	hypothetical protein, partial [Homo sapiens]	6	12	ent movement; defense response	0.893	1.033	0.972
3645059	Chain A, Crystal Structure Of The Apo-Form Of Human Ck2 Alpha	6	26	metabolic process	1.020	1.033	0.951
1195924	electron-transfer-flavoprotein, beta polypeptide [Homo sapiens]	12	95	metabolic process	1.031	1.033	1.048
7680113	PREDICTED: symplekin isoform X2 [Homo sapiens]	5	16	; regulation of biological process	0.850	1.033	0.985
1591647	Chain X, Nmr Structure Of Card D2 Domain	3	14		0.850	1.033	1.106
1195919	ribosomal protein L22, isoform CRA_b [Homo sapiens]	3	35	sis; transport; cell differentiation	0.964	1.033	0.998
4503475	elongation factor 1-alpha 2 [Homo sapiens]	11	124	; regulation of biological process	0.992	1.033	0.993
703093	serine hydroxymethyltransferase, partial [Homo sapiens]	15	97	cell organization and biogenesis	0.960	1.033	1.036
1943792	unnamed protein product [Homo sapiens]	7	35	; regulation of biological process	0.931	1.032	1.098
1271129	dsRNA adenosine deaminase [Homo sapiens]	17	89	nd biogenesis; defense response	1.003	1.032	1.042
1160239	methylthioadenosine phosphorylase [Homo sapiens]	5	10	metabolic process	1.025	1.032	0.915
1196080	heat shock 70kDa protein 5 (glucose-regulated protein, 78kDa), iso	29	535	coagulation; cell communication	1.007	1.032	1.077
7680256	PREDICTED: ubiquitin fusion degradation protein 1 homolog isofo	4	12	; response to stimulus; transport	1.078	1.032	1.071
5249332	Chain A, Structure Of Otud2 Otu Domain	2	2		0.967	1.032	1.003
7679384	PREDICTED: 60S ribosomal protein L26-like 1 isoform X1 [Homo s	7	44	ization and biogenesis; transport	1.137	1.031	1.120
1943755	unnamed protein product [Homo sapiens]	17	104	; regulation of biological process	1.007	1.031	0.996
1425010	Dolichyl-phosphate mannosyltransferase polypeptide 1, catalytic s	5	23	metabolic process	1.227	1.031	1.161
5303695	PREDICTED: U3 small nucleolar ribonucleoprotein protein IMP4 is	2	6	metabolic process	1.035	1.031	1.036
1195848	STT3, subunit of the oligosaccharyltransferase complex, homolog	15	108	ic process; response to stimulus	1.163	1.031	1.029
2052168	KIAA0899 protein [Homo sapiens]	23	100	n; cellular component movement	1.101	1.031	0.970
1491718	hTAFII100 [Homo sapiens]	4	7	cell organization and biogenesis	1.172	1.030	0.994
3478501	FBL protein, partial [Homo sapiens]	12	87	cell organization and biogenesis	1.006	1.030	1.002
1196117	importin 9, isoform CRA_b [Homo sapiens]	9	27	transport	1.112	1.030	1.022
1195794	leukotriene B4 12-hydroxydehydrogenase, isoform CRA_b [Homo s	7	23	ic process; response to stimulus	1.135	1.030	1.032
5303966	PREDICTED: tetratricopeptide repeat protein 9C isoform X1 [Homo	2	7	metabolic process	0.988	1.030	1.025
1401781	KIAA1797 protein, partial [Homo sapiens]	9	19		0.935	1.030	0.940
5788312	PREDICTED: SWI/SNF-related matrix-associated actin-dependent r	4	12	; regulation of biological process	0.956	1.030	1.011
1890531	unnamed protein product [Homo sapiens]	6	29	metabolic process; transport	1.034	1.030	1.028
2210408	unnamed protein product [Homo sapiens]	9	18	transport; coagulation	0.966	1.029	1.014
1943740	unnamed protein product [Homo sapiens]	8	24	metabolic process	0.960	1.029	1.077
1582604	unnamed protein product [Homo sapiens]	12	67	al process; cellular homeostasis	1.055	1.029	1.030
3266340	Chain A, X-ray Structure Of Human Butyrylcholinesterase Inhibited	4	7	metabolic process	1.100	1.029	1.036
1196050	ribosomal protein L18a, isoform CRA_b [Homo sapiens]	6	51	ization and biogenesis; transport	1.005	1.029	0.910

5304005	PREDICTED: nuclear pore complex protein Nup107 isoform X1 [Ho	6	16	; transport; response to stimulus	1.011	1.029	1.062
1195810	keratin 20 [Homo sapiens]	3	29	; regulation of biological process	1.022	1.028	0.927
1578345	Chain A, Aldehyde Reductase	7	52	metabolic process	0.915	1.028	1.200
1147944	Chain A, Crystal Structure Of Human Carbonyl Reductase 3, Comp	8	35	metabolic process	0.976	1.028	1.127
7679799	PREDICTED: RAC-alpha serine/threonine-protein kinase isoform X	9	15	cell organization and biogenesis	1.022	1.028	1.129
1173905	spliceosome associated protein [Homo sapiens]	9	52	metabolic process	0.967	1.027	1.020
7679037	PREDICTED: protein argonaute-1 isoform X4 [Homo sapiens]	3	8	nd biogenesis; defense response	0.924	1.027	0.975
1585128	isopeptidase T	12	87	metabolic process	1.032	1.027	1.048
1582618	unnamed protein product [Homo sapiens]	17	119	; response to stimulus; transport	1.012	1.027	1.003
4767875	YWHAH [Homo sapiens]	10	116	cell organization and biogenesis	0.988	1.027	1.008
3230715	UDP-N-acetylglucosamine--peptide N-acetylglucosaminyltransfera	4	5	cell death; response to stimulus	1.013	1.026	1.125
1195984	optic atrophy 1 (autosomal dominant), isoform CRA_b [Homo sapi	16	56	al process; response to stimulus	0.935	1.026	0.962
1196239	superkiller viralicidic activity 2-like (S. cerevisiae), isoform CRA_b	4	7	metabolic process	0.966	1.026	1.002
2210444	unnamed protein product [Homo sapiens]	10	32	transport; metabolic process	1.122	1.026	1.031
1043608	RNA binding motif protein 8B [Homo sapiens]	3	24	n of biological process; transport	1.132	1.025	1.156
1195955	chromosome 20 open reading frame 14, isoform CRA_d [Homo sa	16	76	; regulation of biological process	1.009	1.025	1.051
2210417	unnamed protein product [Homo sapiens]	5	37	ization and biogenesis; transport	1.064	1.025	1.038
6168054	Chain A, Structural Basis For Recruitment Of Ubc12 By An E2-Bind	3	40		1.013	1.025	1.051
1890675	unnamed protein product [Homo sapiens]	2	16	transport	0.998	1.025	1.115
7561426	Chain b, Human Constitutive 20s Proteasome In Complex With Ca	5	14		0.941	1.025	0.979
1332519	C11orf68 protein, partial [Homo sapiens]	3	8		1.136	1.024	1.091
3228584	calcium modulating cyclophilin ligand CAMLG, partial [Homo sapi	2	9	; regulation of biological process	0.973	1.024	1.059
4097975	unnamed protein product, partial [Homo sapiens]	2	4		1.169	1.024	0.981
2210431	unnamed protein product [Homo sapiens]	6	16	e response; response to stimulus	0.971	1.024	0.989
1890540	unnamed protein product [Homo sapiens]	10	73	; regulation of biological process	1.170	1.023	1.067
1582602	unnamed protein product [Homo sapiens]	9	30	transport; metabolic process	1.305	1.023	0.985
5303723	PREDICTED: acylamino-acid-releasing enzyme isoform X6 [Homo	12	51	cell organization and biogenesis	1.006	1.023	0.993
1195786	polymerase (RNA) I polypeptide E, 53kDa, isoform CRA_a [Homo s	4	13	; regulation of biological process	0.988	1.023	0.970
7023733	unnamed protein product [Homo sapiens]	3	8	lifferentiation; metabolic process	1.067	1.023	0.918
3389689	cold shock domain-containing protein E1 isoform 2 [Homo sapien	7	22	gical process; metabolic process	0.979	1.023	0.885
7679528	PREDICTED: protein argonaute-2 isoform X4 [Homo sapiens]	10	25	nd biogenesis; defense response	0.935	1.023	1.018
7682779	STRN protein [Homo sapiens]	4	6	cell organization and biogenesis	0.830	1.022	0.908
1417970	IQ motif containing GTPase activating protein 1 [Homo sapiens]	34	200	esponse to stimulus; cell growth	1.089	1.022	1.052
1196113	KIAA0859, isoform CRA_d [Homo sapiens]	4	6	metabolic process	0.874	1.022	1.041
2197530	unnamed protein product, partial [Homo sapiens]	3	5		0.738	1.021	0.888
6289765	elongation protein 3 homolog, partial [Homo sapiens]	8	26	n of biological process; transport	1.025	1.020	1.007
1043282	unnamed protein product [Homo sapiens]	5	21	cell organization and biogenesis	0.876	1.020	1.006
6841462	HSPC120 [Homo sapiens]	7	64	c process; transport; cell growth	0.994	1.019	0.952
3335397	TNRC11, partial [Homo sapiens]	2	4	al process; response to stimulus	1.054	1.019	1.070
1195990	G elongation factor, mitochondrial 1, isoform CRA_c [Homo sapie	8	30	cell organization and biogenesis	1.171	1.019	1.091

1943759	unnamed protein product [Homo sapiens]	9	249	tabolic process; cell proliferation	1.018	1.019	1.086
3929969	UV excision repair protein RAD23 homolog A isoform 3 [Homo sap	7	34	; regulation of biological process	1.057	1.019	0.927
1943809	unnamed protein product [Homo sapiens]	13	62	metabolic process	1.009	1.019	0.965
1937878	unnamed protein product [Homo sapiens]	2	5	e to stimulus; metabolic process	1.007	1.019	1.237
1196289	neurobeachin, isoform CRA_c [Homo sapiens]	12	22	transport	1.103	1.018	1.116
7679025	PREDICTED: zinc transporter 7 isoform X1 [Homo sapiens]	4	13	; regulation of biological process	0.839	1.018	0.876
4097537	unnamed protein product, partial [Homo sapiens]	2	3		0.852	1.018	0.954
4680717	CGI-39 protein [Homo sapiens]	4	27		1.143	1.018	0.970
1195931	ARD1 homolog A, N-acetyltransferase (S. cerevisiae), isoform CRA	2	13	d biogenesis; metabolic process	1.040	1.017	1.010
2037902	Rho GDP dissociation inhibitor alpha [Homo sapiens]	5	45	al process; response to stimulus	1.227	1.016	0.894
2032825	Chain B, Crystal Structure Of Human Protein Kinase Ck2 Regulato	4	18		1.058	1.016	1.025
1890691	unnamed protein product [Homo sapiens]	12	84	biogenesis; response to stimulus	1.078	1.016	1.016
5788193	PREDICTED: heterogeneous nuclear ribonucleoprotein H3 isoform	7	133	bolic process; cell differentiation	1.007	1.016	1.052
1043901	unnamed protein product [Homo sapiens]	2	5	f biological process; cell division	0.722	1.016	0.918
1943781	unnamed protein product [Homo sapiens]	13	55	cell organization and biogenesis	1.097	1.016	0.998
7679302	PREDICTED: sister chromatid cohesion protein PDS5 homolog A i	10	22	f biological process; cell division	1.035	1.016	0.971
5304109	PREDICTED: flotillin-2 isoform X3 [Homo sapiens]	4	14	; regulation of biological process	1.002	1.016	1.000
1669555	RBP-MS/type 5, partial [Homo sapiens]	2	5	; regulation of biological process	0.843	1.015	1.004
3478298	CAND1 protein, partial [Homo sapiens]	21	77	; regulation of biological process	1.098	1.015	1.010
1196224	adaptor-related protein complex 3, sigma 2 subunit, isoform CRA_	3	11	rt; cellular component movement	0.981	1.014	0.867
1196207	metastasis associated 1 family, member 3, isoform CRA_d [Homo	6	22	; regulation of biological process	0.963	1.014	1.008
1943857	unnamed protein product [Homo sapiens]	13	78	metabolic process	1.060	1.014	1.023
7511305	6-phosphogluconate dehydrogenase, decarboxylating isoform 2 [H	13	78	metabolic process	1.053	1.014	1.023
3199188	CORO7-PAM16 protein [Homo sapiens]	3	7		0.899	1.014	0.982
1196068	GDP dissociation inhibitor 2, isoform CRA_a [Homo sapiens]	18	159	us; transport; metabolic process	1.198	1.013	1.033
1195808	NHP2 non-histone chromosome protein 2-like 1 (S. cerevisiae), iso	2	9	metabolic process	0.940	1.013	1.097
1527750	ACTB protein, partial [Homo sapiens]	14	895	; coagulation; defense response	1.138	1.013	0.990
1890536	unnamed protein product [Homo sapiens]	10	85	; transport; response to stimulus	1.144	1.013	0.992
4079438	Chain C, Crystal Structure Of Mif L46g Mutant	2	65		1.140	1.013	0.716
1195875	radixin, isoform CRA_c [Homo sapiens]	13	63	biogenesis; response to stimulus	0.964	1.013	1.043
7679361	PREDICTED: lysine-specific demethylase 3B isoform X2 [Homo sa	5	10	al process; response to stimulus	1.331	1.013	1.105
1937856	unnamed protein product [Homo sapiens]	2	4	nd biogenesis; defense response	0.957	1.013	0.956
1196147	WD repeat domain 68, isoform CRA_a [Homo sapiens]	2	8	development; metabolic process	1.055	1.013	1.012
5303707	PREDICTED: mitochondrial chaperone BCS1 isoform X3 [Homo sa	2	3	cell organization and biogenesis	0.990	1.012	1.090
5839661	isocitrate dehydrogenase [NADP], mitochondrial isoform 2 [Homo	18	163	metabolic process	1.081	1.012	1.048
1196041	nuclear respiratory factor 1, isoform CRA_c [Homo sapiens]	2	8	cell organization and biogenesis	0.900	1.012	0.969
7679085	PREDICTED: torsin-1A-interacting protein 2 isoform X2 [Homo sap	3	5	; regulation of biological process	0.911	1.012	0.949
337580	ribosomal protein L3, partial [Homo sapiens]	16	88	; transport; response to stimulus	1.143	1.012	0.995
2646815	WD repeat-containing protein 26 isoform b [Homo sapiens]	4	17		1.124	1.012	1.116
1196052	ATPase type 13A1, isoform CRA_f [Homo sapiens]	10	39	; transport; cellular homeostasis	0.981	1.012	1.013

7679482	PREDICTED: solute carrier family 25 member 40 isoform X3 [Homo	3	7	transport	0.897	1.011	1.075
5469689	tyrosine 3-monooxygenase/tryptophan 5-monooxygenase activatio	11	139	cell organization and biogenesis	1.018	1.011	1.005
1195861	ribosomal protein L36a-like [Homo sapiens]	3	21	metabolic process	1.126	1.011	0.895
3964479	EEF1G protein, partial [Homo sapiens]	17	165	ic process; response to stimulus	1.038	1.010	1.013
1404281	unnamed protein product [Homo sapiens]	2	4	metabolic process	1.132	1.010	0.986
2015119	Chain F, Structure Of Human Glutamate Dehydrogenase-Apo Form	14	96	metabolic process	1.097	1.010	1.051
1196249	family with sequence similarity 62 (C2 domain containing) member	8	27	transport	1.031	1.010	0.959
5303884	PREDICTED: DNA replication licensing factor MCM4 isoform X1 [H	11	36	metabolic process	0.986	1.010	0.987
3333956	crn-related protein kim1 [Homo sapiens]	10	35	ic process; response to stimulus	0.917	1.009	0.982
1196303	chaperonin containing TCP1, subunit 8 (theta), isoform CRA_a [Ho	23	233	ization and biogenesis; transport	1.131	1.009	1.035
3315079	ATP-dependent RNA helicase [Homo sapiens]	2	3	metabolic process	0.818	1.009	0.944
3486797	dipeptidylpeptidase 9 short form [Homo sapiens]	4	11	metabolic process	0.809	1.009	0.893
1312181	unnamed protein product [Homo sapiens]	4	27		0.826	1.009	0.824
7679938	PREDICTED: AP-2 complex subunit beta isoform X5 [Homo sapien	18	75	cell organization and biogenesis	1.002	1.009	0.954
1309730	RAP2C, member of RAS oncogene family [Homo sapiens]	2	6	cell organization and biogenesis	1.017	1.009	1.079
1098637	fragile X mental retardation syndrome related protein [Homo sapie	8	24	regulation of biological process	0.991	1.008	1.042
6599262	hypothetical protein, partial [Homo sapiens]	7	15	; regulation of biological process	1.142	1.008	0.993
7680459	PREDICTED: phosphatidylinositol 5-phosphate 4-kinase type-2 bet	2	9	al process; response to stimulus	0.884	1.008	1.013
1195684	protein tyrosine phosphatase, receptor type, K, isoform CRA_e [Ho	2	4	cell organization and biogenesis	0.880	1.008	1.113
1196037	peptidase (mitochondrial processing) beta, isoform CRA_d [Homo	5	23	metabolic process; transport	0.972	1.008	0.918
1860629	VPRBP protein [Homo sapiens]	3	7	cell organization and biogenesis	0.731	1.008	1.008
1943862	unnamed protein product [Homo sapiens]	2	2	cell organization and biogenesis	0.975	1.007	0.984
1166673	Chain E, Crystal Structure Of P5cr Complexed With Nadh	7	108	metabolic process	1.117	1.007	0.973
1646910	unnamed protein product, partial [Homo sapiens]	7	23	ic process; response to stimulus	0.974	1.007	0.943
8340516	RELA protein [Homo sapiens]	10	35	bolic process; defense response	0.928	1.007	1.064
1196132	DEAH (Asp-Glu-Ala-His) box polypeptide 15, isoform CRA_h [Hom	22	114	metabolic process	1.051	1.006	0.993
1195837	proline synthetase co-transcribed homolog (bacterial), isoform CR	2	10	metabolic process	0.981	1.006	0.902
5254589	hypothetical protein, partial [Homo sapiens]	6	12	; regulation of biological process	0.862	1.006	1.010
1196025	glycosylphosphatidylinositol anchor attachment protein 1 homolog	6	18	d biogenesis; metabolic process	0.933	1.006	0.916
1943838	unnamed protein product [Homo sapiens]	7	178	; regulation of biological process	1.075	1.005	1.047
1195719	coiled-coil domain containing 43, isoform CRA_a [Homo sapiens]	2	6		1.171	1.005	1.051
1195835	farnesyltransferase, CAAX box, alpha, isoform CRA_d [Homo sapi	3	9	e to stimulus; metabolic process	1.144	1.005	1.084
1890531	unnamed protein product [Homo sapiens]	8	84	ization and biogenesis; transport	1.152	1.004	0.994
2821658	transmembrane emp24 domain-containing protein 5 isoform 1 pre	2	17	cell organization and biogenesis	0.863	1.004	1.033
5472347	RRP12-like protein isoform 3 [Homo sapiens]	12	32		0.921	1.004	1.022
1582555	unnamed protein product [Homo sapiens]	13	33	metabolic process	1.034	1.004	0.980
1890542	unnamed protein product [Homo sapiens]	2	5	f biological process; cell division	1.018	1.004	0.944
7679716	PREDICTED: importin-8 isoform X4 [Homo sapiens]	3	7	e to stimulus; metabolic process	0.954	1.004	1.019
1582554	unnamed protein product [Homo sapiens]	3	9	gical process; metabolic process	1.089	1.004	0.935
6759699	Chain A, Crystal Structure Of Human Stromal Antigen 2 (sa2) In Co	6	13		0.952	1.003	1.052

6634296	protein transport protein Sec31A isoform 6 [Homo sapiens]	14	46	cell organization and biogenesis	0.936	1.003	0.994
1582570	unnamed protein product [Homo sapiens]	9	24	cell organization and biogenesis	0.897	1.003	1.006
1195729	adaptor-related protein complex 2, alpha 1 subunit, isoform CRA_a	25	109	n; cellular component movement	1.053	1.003	0.973
5788390	PREDICTED: ATP-dependent RNA helicase DDX3Y isoform X1 [Ho	13	85	; regulation of biological process	1.050	1.003	0.904
5639665	EH-domain containing protein testilin [Homo sapiens]	7	20	biogenesis; response to stimulus	0.964	1.003	0.949
2098705	Chain C, Crystal Structure Of The Human Copii-Coat Protein Sec24	17	103	transport	0.974	1.003	0.989
1943872	unnamed protein product [Homo sapiens]	4	21	ization and biogenesis; transport	1.074	1.002	1.009
5304112	PREDICTED: Golgi SNAP receptor complex member 1 isoform X1 [	2	4	; regulation of biological process	0.968	1.002	1.049
5787992	PREDICTED: mRNA turnover protein 4 homolog isoform X1 [Homo	4	16		0.943	1.002	1.028
1890690	unnamed protein product [Homo sapiens]	3	16		1.238	1.002	0.974
1022961	HuR RNA binding protein [Homo sapiens]	11	119	; regulation of biological process	0.998	1.001	0.968
1195947	zinc finger protein-like 1, isoform CRA_b, partial [Homo sapiens]	3	5	n of biological process; transport	1.178	1.001	0.842
1943836	unnamed protein product [Homo sapiens]	15	106	cell organization and biogenesis	1.032	1.001	1.033
1943860	unnamed protein product [Homo sapiens]	3	10	metabolic process	0.953	1.001	1.049
1972104	uridine monophosphate synthetase isoform G [Homo sapiens]	9	32	; transport; response to stimulus	1.070	1.001	1.097
1187644	VWA2 protein [Homo sapiens]	2	5	cell organization and biogenesis	0.887	1.001	1.024
7679815	PREDICTED: L-2-hydroxyglutarate dehydrogenase, mitochondrial i	9	42	metabolic process	0.905	1.001	0.964
1937858	unnamed protein product [Homo sapiens]	7	24	d biogenesis; metabolic process	1.022	1.000	0.994
2210420	unnamed protein product [Homo sapiens]	27	228	; regulation of biological process	1.058	1.000	0.982
4097620	unnamed protein product, partial [Homo sapiens]	3	10		1.475	1.000	0.955
7679105	PREDICTED: threonine--tRNA ligase, mitochondrial isoform X1 [Ho	7	32	metabolic process	0.904	1.000	1.014
2210430	unnamed protein product [Homo sapiens]	3	24	metabolic process	0.895	0.999	0.916
1196235	histone 1, H2bl [Homo sapiens]	9	249	cell organization and biogenesis	1.225	0.999	0.972
4004173	unnamed protein product, partial [Homo sapiens]	12	96		1.172	0.999	0.976
1890537	unnamed protein product [Homo sapiens]	2	6	metabolic process	1.058	0.999	0.952
1195767	nucleolar complex associated 2 homolog (S. cerevisiae), isoform C	5	16	; transport; response to stimulus	0.923	0.998	0.936
5303709	PREDICTED: tubulin alpha-4A chain isoform X1 [Homo sapiens]	15	416	tion and biogenesis; coagulation	1.134	0.998	1.026
3387452	SYNCRIP protein, partial [Homo sapiens]	11	58	al process; response to stimulus	0.946	0.998	0.960
4078803	ubiquitin-specific proteinase 34 [Homo sapiens]	2	3	al process; response to stimulus	0.801	0.998	0.902
5303748	PREDICTED: presenilins-associated rhomboid-like protein, mitoch	2	5	; regulation of biological process	1.229	0.998	0.918
190117	PML-1 [Homo sapiens]	3	6	homeostasis; defense response	0.985	0.998	1.047
5304159	PREDICTED: cell adhesion molecule 4 isoform X1 [Homo sapiens]	4	13		1.168	0.998	1.056
7679851	PREDICTED: kunitz-type protease inhibitor 1 isoform X3 [Homo sa	2	5	cell organization and biogenesis	0.895	0.997	1.115
1196232	heterogeneous nuclear ribonucleoprotein H2 (H') [Homo sapiens]	9	114	metabolic process	1.005	0.997	1.019
7679899	PREDICTED: diphosphomevalonate decarboxylase isoform X3 [Ho	6	21	; regulation of biological process	1.240	0.997	0.958
1890665	unnamed protein product [Homo sapiens]	3	60	regulation of biological process	1.077	0.997	0.959
2320059	Mina53 form-3 [Homo sapiens]	2	3	gical process; metabolic process	0.851	0.997	0.982
1943794	unnamed protein product [Homo sapiens]	2	6	; regulation of biological process	0.974	0.996	0.948
1195822	transcription elongation regulator 1, isoform CRA_c [Homo sapien	8	19	gical process; metabolic process	0.925	0.996	0.992
1265579	prune protein [Homo sapiens]	3	9		0.750	0.996	0.960

7679947	PREDICTED: lethal(2) giant larvae protein homolog 2 isoform X6 [H	10	34	tion and biogenesis; cell division	1.130	0.996	0.988
1196160	hCG1979897, isoform CRA_b [Homo sapiens]	2	11		0.955	0.996	1.042
2210425	unnamed protein product [Homo sapiens]	14	69	cell organization and biogenesis	1.142	0.995	1.012
1195897	translocase of inner mitochondrial membrane 13 homolog (yeast),	2	19	cell organization and biogenesis	0.914	0.995	0.938
1783407	haymaker protein, partial [Homo sapiens]	3	20	; regulation of biological process	1.151	0.995	0.978
1196097	exocyst complex component 7, isoform CRA_d [Homo sapiens]	2	4	; regulation of biological process	1.149	0.995	1.019
1507941	RNA methyltransferase like 1 [Homo sapiens]	2	9	metabolic process	1.162	0.995	1.154
3187464	hypothetical protein [Homo sapiens]	3	5	response; response to stimulus	1.128	0.995	1.140
1195871	adenine phosphoribosyltransferase, isoform CRA_b [Homo sapien	7	65	; transport; response to stimulus	1.055	0.994	1.020
7679705	PREDICTED: sortilin-related receptor isoform X1 [Homo sapiens]	6	11	e to stimulus; metabolic process	0.964	0.994	0.962
1196187	hCG1782202, isoform CRA_c [Homo sapiens]	6	44	al process; response to stimulus	1.163	0.994	0.976
7679795	PREDICTED: FACT complex subunit SPT16 isoform X1 [Homo sap	30	214	; regulation of biological process	1.086	0.994	1.029
4814558	PTK9L [Homo sapiens]	4	9	biogenesis; response to stimulus	1.112	0.994	1.019
4758844	nuclear pore complex protein Nup155 isoform 2 [Homo sapiens]	16	37	al process; response to stimulus	1.026	0.994	0.939
6094124	Chain 5, Partial Rotational Order Disorder Structure Of Human Ste	2	7		0.944	0.994	1.056
4187240	FAD synthase isoform 2 [Homo sapiens]	7	21	metabolic process	0.967	0.994	0.988
3212998	spliceosome associated protein 114 (SF3a) [Homo sapiens]	7	24	d biogenesis; metabolic process	0.994	0.994	1.062
2820787	unnamed protein product, partial [Homo sapiens]	2	6	transport	1.153	0.994	1.016
1196094	protein kinase, cAMP-dependent, regulatory, type I, alpha (tissue s	3	4	fense response; cell proliferation	1.093	0.993	0.853
1196228	CD151 antigen (Raph blood group), isoform CRA_c, partial [Homo	4	12	and biogenesis; cell proliferation	1.143	0.993	1.044
5788368	PREDICTED: nuclear pore complex protein Nup50 isoform X2 [Hor	2	3	al process; response to stimulus	0.945	0.993	0.727
1890540	unnamed protein product [Homo sapiens]	2	5	regulation of biological process	1.002	0.993	0.944
7679042	PREDICTED: agrin isoform X4 [Homo sapiens]	14	33	n; cellular component movement	0.932	0.993	0.932
2026998	HNF1-alpha dimerization cofactor [Homo sapiens]	2	3	cell organization and biogenesis	0.747	0.993	0.909
1043359	unnamed protein product [Homo sapiens]	12	39	metabolic process	0.953	0.993	0.970
1943846	unnamed protein product [Homo sapiens]	6	13	l proliferation; defense response	0.969	0.993	0.910
8270124	platelet-activating factor acetylhydrolase IB subunit beta isoform e	3	28	; regulation of biological process	1.140	0.992	1.220
1196097	tripartite motif-containing 65, isoform CRA_d [Homo sapiens]	3	5	regulation of biological process	0.786	0.992	1.046
1280422	Unknown (protein for IMAGE:3543711), partial [Homo sapiens]	17	120	; response to stimulus; transport	0.990	0.992	0.992
1196143	3-hydroxyisobutyrate dehydrogenase, isoform CRA_b [Homo sapie	2	5	metabolic process	0.896	0.992	0.993
1582595	unnamed protein product [Homo sapiens]	10	60	s; coagulation; defense response	0.995	0.992	0.942
6347433	bromodomain-containing protein 2 isoform 4 [Homo sapiens]	3	9	; regulation of biological process	0.993	0.992	0.964
7235870	Chain B, Crystal Structure Of N6-methyladenosine Rna Reader Yth	3	11		1.129	0.992	1.094
1043878	unnamed protein product [Homo sapiens]	2	2	nunication; response to stimulus	0.897	0.991	0.982
2210442	unnamed protein product [Homo sapiens]	12	50	; regulation of biological process	1.156	0.991	0.993
2037905	small GTP binding protein RAB6C [Homo sapiens]	9	101	s; cellular component movement	1.130	0.991	0.982
1196059	solute carrier family 9 (sodium/hydrogen exchanger), member 3 re	3	16	cell organization and biogenesis	0.777	0.990	0.905
609449	amyloid precursor protein, partial [Homo sapiens]	4	14	response; cell growth; cell death	1.078	0.990	0.967
1195765	ATPase family, AAA domain containing 3A, isoform CRA_b [Homo	15	52	; regulation of biological process	0.992	0.990	1.004
2259034	mannosyl-oligosaccharide glucosidase isoform 2 [Homo sapiens]	10	37	metabolic process	1.047	0.990	0.986

5787988	PREDICTED: microtubule-actin cross-linking factor 1 isoform X27	2	5	ic process; response to stimulus	1.216	0.990	1.069
3006395	unnamed protein product [Homo sapiens]	7	35		0.939	0.990	1.216
6399524	unknown [Homo sapiens]	2	4	metabolic process	0.841	0.989	0.875
1943749	unnamed protein product [Homo sapiens]	9	42	; regulation of biological process	1.056	0.989	0.934
5453565	brain-specific angiogenesis inhibitor 1-associated protein 2 isofor	6	24	se to stimulus; defense response	0.933	0.989	0.850
1582585	unnamed protein product [Homo sapiens]	15	57	ic process; response to stimulus	1.139	0.989	0.975
1195784	tubulin, alpha 3, isoform CRA_c [Homo sapiens]	17	450	ogenesis; transport; cell division	1.180	0.988	1.030
1196071	v-yes-1 Yamaguchi sarcoma viral related oncogene homolog, isofo	4	11	mmunication; defense response	1.178	0.988	1.104
1266349	unnamed protein product [Homo sapiens]	2	3		0.908	0.988	1.063
5788370	PREDICTED: histone acetyltransferase p300 isoform X1 [Homo sap	6	13	e response; cellular homeostasis	1.149	0.988	1.115
1196025	diacylglycerol O-acyltransferase homolog 1 (mouse), isoform CRA	2	7	cell organization and biogenesis	1.199	0.988	0.920
5566974	Chain A, The Crystal Structure And Mechanism Of Human Glutaryl	5	12	metabolic process	0.917	0.988	0.896
4575156	ERC1 protein, partial [Homo sapiens]	2	5	process; development; transport	0.825	0.987	1.084
1943813	unnamed protein product [Homo sapiens]	12	41	cell organization and biogenesis	1.067	0.987	0.989
1043394	unnamed protein product [Homo sapiens]	5	8	; metabolic process; cell division	0.953	0.987	0.836
8080432	Chain A, Crytsal Structure Of Human Pleiotropic Regulator 1 (prl1)	8	16		0.910	0.987	0.984
1890691	unnamed protein product [Homo sapiens]	3	13	o stimulus; cellular homeostasis	1.146	0.987	1.062
5697202	Unknown (protein for IMAGE:30389268), partial [Homo sapiens]	8	38	metabolic process	0.999	0.987	0.926
3432935	SFRS protein kinase 2 isoform c [Homo sapiens]	3	8	se to stimulus; defense response	1.027	0.986	1.046
1195866	defender against cell death 1, isoform CRA_a [Homo sapiens]	3	32	; regulation of biological process	1.310	0.986	1.030
1943751	unnamed protein product [Homo sapiens]	4	13	ic process; response to stimulus	0.904	0.986	1.122
7023581	unnamed protein product [Homo sapiens]	2	3		1.571	0.986	1.000
8626693	negative elongation factor B [Homo sapiens]	4	11	; regulation of biological process	1.200	0.985	1.070
1943835	unnamed protein product [Homo sapiens]	4	10	sponse to stimulus; cell division	1.167	0.985	0.945
1582576	unnamed protein product [Homo sapiens]	2	3	cess; cell proliferation; transport	0.951	0.985	0.865
4826960	glutamine--tRNA ligase isoform a [Homo sapiens]	27	141	metabolic process	1.024	0.985	0.969
8160451	Chain D, Crystal Structure Of Human Trap1 With Amppnp	16	368		1.178	0.985	1.061
5304001	PREDICTED: heterogeneous nuclear ribonucleoprotein A1 isoform	12	137	lic process; transport; cell death	1.107	0.985	1.184
1582566	unnamed protein product [Homo sapiens]	19	74	n; defense response; cell growth	0.973	0.985	0.939
5304049	PREDICTED: serine/threonine-protein kinase MRCK beta isoform X	8	12	s; cellular component movement	0.963	0.985	0.906
6318548	retinal short-chain dehydrogenase/reductase retSDR4 [Homo sapi	7	22	metabolic process	0.852	0.984	0.981
1706408	p25 [Homo sapiens]	5	35	; regulation of biological process	0.987	0.984	0.986
1195734	misato homolog 1 (Drosophila), isoform CRA_a [Homo sapiens]	2	6	d biogenesis; metabolic process	0.748	0.984	0.893
1196033	hCG1782167, isoform CRA_a [Homo sapiens]	18	51		0.984	0.984	0.987
6208788	heat shock 70kDa protein 4 isoform a variant, partial [Homo sapien	24	151	; response to stimulus; transport	1.186	0.984	1.024
4102711	EH-binding protein, partial [Homo sapiens]	2	5	al process; response to stimulus	0.961	0.984	0.876
1582559	unnamed protein product [Homo sapiens]	15	48	cell organization and biogenesis	1.072	0.984	0.952
5304182	PREDICTED: regulation of nuclear pre-mRNA domain-containing p	4	15	; regulation of biological process	1.337	0.984	1.021
1195775	platelet-activating factor acetylhydrolase, isoform Ib, gamma subu	6	28	metabolic process	0.913	0.984	0.988
9327859	Chain B, Irf3-Cbp Complex	5	10	regulation of biological process	0.904	0.984	0.952

1196259	ubiquitin-activating enzyme E1-like 2, isoform CRA_a [Homo sapie	7	17	ic process; response to stimulus	1.112	0.984	0.995
1890532	unnamed protein product [Homo sapiens]	4	9	cell organization and biogenesis	1.118	0.984	0.870
3405454	mysterin [Homo sapiens]	14	28	metabolic process	0.916	0.984	0.992
1195987	ATPase, Class VI, type 11B, isoform CRA_d [Homo sapiens]	2	2	cell organization and biogenesis	0.781	0.983	0.935
3306892	Chain A, Refinement Of Rna Binding Domain In Human Tra2 Beta P	3	34		1.023	0.983	0.914
1195773	spectrin, beta, non-erythrocytic 4, isoform CRA_c [Homo sapiens]	2	13	ent; transport; defense response	0.945	0.983	1.056
1577386	E3 ubiquitin-protein ligase HECTD3 [Homo sapiens]	3	6	metabolic process	0.840	0.983	0.934
1195944	hCG2020143, isoform CRA_a [Homo sapiens]	4	12	nd biogenesis; cell differentiation	0.978	0.983	0.816
1581615	oligosaccharyltransferase	9	60	metabolic process	1.076	0.982	1.021
1195753	similar to RIKEN cDNA 4732495G21 gene [Homo sapiens]	4	302		1.056	0.982	0.992
4147272	unknown [Homo sapiens]	3	18	metabolic process	0.964	0.982	0.977
8047856	STRBP protein, partial [Homo sapiens]	8	14	velopment; response to stimulus	0.813	0.982	1.004
2736256	aflatoxin aldehyde reductase AFAR [Homo sapiens]	8	44	metabolic process	1.079	0.982	0.922
3478280	NIBP protein [Homo sapiens]	5	6	cell organization and biogenesis	0.936	0.982	1.022
5304271	PREDICTED: WD repeat-containing protein 5 isoform X1 [Homo sa	8	16	; regulation of biological process	1.030	0.982	1.076
1943911	unnamed protein product [Homo sapiens]	4	8	al process; response to stimulus	0.877	0.981	1.052
4712527	CRYZ protein [Homo sapiens]	4	12	cell organization and biogenesis	0.892	0.981	1.014
1196161	G elongation factor, mitochondrial 2, isoform CRA_c [Homo sapier	2	3	cell organization and biogenesis	1.044	0.981	0.856
2988398	Unknown gene product [Homo sapiens]	5	30		1.044	0.981	0.965
5254573	hypothetical protein, partial [Homo sapiens]	2	4		1.044	0.981	1.066
6573280	beta tropomyosin, partial [Homo sapiens]	7	59	; regulation of biological process	1.293	0.981	1.078
1196122	WD repeats and SOF1 domain containing, isoform CRA_a [Homo s	4	9	metabolic process	1.128	0.980	1.020
1195748	hCG2024613, isoform CRA_c [Homo sapiens]	3	7	cell organization and biogenesis	1.000	0.980	0.882
1582614	unnamed protein product [Homo sapiens]	4	7	; transport; response to stimulus	0.968	0.980	0.885
2279681	Chain A, The Mad2 Spindle Checkpoint Protein Undergoes Similar	3	10		1.031	0.980	1.017
1195960	ADP-ribosylation factor guanine nucleotide-exchange factor 2 (bre	5	10	al process; response to stimulus	0.997	0.980	0.986
7679895	PREDICTED: L-fucose kinase isoform X6 [Homo sapiens]	2	5	metabolic process	0.972	0.979	0.752
1195698	hCG40944, isoform CRA_a [Homo sapiens]	3	6		0.868	0.979	0.958
1929871	Chain B, Crystal Structure Of The Protein-disulfide Isomerase Rela	3	6	transport	0.999	0.979	1.056
1943826	unnamed protein product [Homo sapiens]	7	14	ic process; response to stimulus	0.920	0.979	0.963
1968411	NCLN protein [Homo sapiens]	14	63	; regulation of biological process	1.011	0.979	0.948
1937861	unnamed protein product [Homo sapiens]	20	144	ent movement; defense response	1.042	0.978	0.973
1196098	ubiquitin-conjugating enzyme E2O, isoform CRA_d [Homo sapiens	5	9	n of biological process; transport	0.863	0.978	1.012
2037905	small GTP binding protein RAB6A [Homo sapiens]	9	100	s; cellular component movement	1.114	0.978	0.971
7807052	UBE2Q1 protein, partial [Homo sapiens]	3	5	us; metabolic process; transport	0.897	0.977	1.108
1162839	UBTF protein, partial [Homo sapiens]	3	5	d biogenesis; metabolic process	0.764	0.977	1.205
1196318	hCG1811743, isoform CRA_f [Homo sapiens]	5	18		0.889	0.977	0.932
7679665	PREDICTED: mitochondrial glutamate carrier 1 isoform X1 [Homo s	8	38	transport	1.067	0.977	1.014
1579726	unnamed protein product, partial [Homo sapiens]	4	6		0.858	0.977	0.994
1195802	activating signal cointegrator 1 complex subunit 2, isoform CRA_a	2	5	; regulation of biological process	1.119	0.977	0.988

1195914	centaurin, gamma 2, isoform CRA_b [Homo sapiens]	4	7	; response to stimulus; transport	0.913	0.977	1.027
6289769	mitochondrial ribosomal protein L24 variant, partial [Homo sapiens	3	9	cell organization and biogenesis	0.889	0.977	0.976
1196011	exocyst complex component 5, isoform CRA_a [Homo sapiens]	3	4	d biogenesis; metabolic process	0.990	0.976	1.095
7336061	tubulin alpha-1C chain isoform b [Homo sapiens]	17	419	tion and biogenesis; cell division	1.224	0.976	1.044
1890550	unnamed protein product [Homo sapiens]	8	33	cell organization and biogenesis	1.199	0.976	0.959
1943867	unnamed protein product [Homo sapiens]	5	29	ent movement; defense response	0.919	0.976	0.936
1943912	unnamed protein product [Homo sapiens]	3	10	s; cellular component movement	0.857	0.976	0.885
1196213	tyrosine 3-monooxygenase/tryptophan 5-monooxygenase activatio	11	145	cell organization and biogenesis	1.029	0.976	1.004
5303765	PREDICTED: 60S ribosomal protein L9 isoform X1 [Homo sapiens]	6	34	ization and biogenesis; transport	0.984	0.976	0.950
5303797	PREDICTED: eukaryotic peptide chain release factor subunit 1 isof	4	11	; regulation of biological process	0.978	0.975	0.858
5254574	hypothetical protein, partial [Homo sapiens]	7	27	; response to stimulus; transport	1.098	0.975	0.971
1937851	unnamed protein product [Homo sapiens]	24	182	ization and biogenesis; transport	1.019	0.975	0.928
1195716	RAD17 homolog (S. pombe), isoform CRA_g [Homo sapiens]	2	3	; regulation of biological process	0.813	0.975	1.042
1582598	unnamed protein product [Homo sapiens]	9	57	metabolic process	1.218	0.975	0.956
5595818	surfeit 1, partial [Homo sapiens]	2	2	ization and biogenesis; transport	0.906	0.975	0.943
8240748	Chain A, Crystal Structure Of Vps4b	2	9	metabolic process	0.946	0.975	0.995
1196266	SEC24 related gene family, member B (S. cerevisiae) [Homo sapier	8	32	; regulation of biological process	1.033	0.975	0.956
1943794	unnamed protein product [Homo sapiens]	4	7	l proliferation; cell differentiation	1.047	0.975	0.910
4309884	similar to Schizosaccharomyces pombe splicing factor; similar to	3	17	metabolic process	0.992	0.974	1.003
1195730	protoporphyrinogen oxidase, isoform CRA_b [Homo sapiens]	12	51	ic process; response to stimulus	0.959	0.974	0.953
1890690	unnamed protein product [Homo sapiens]	3	6	metabolic process	1.061	0.974	1.046
6282219	unknown [Homo sapiens]	8	33	metabolic process	1.103	0.974	0.958
5304135	PREDICTED: COP9 signalosome complex subunit 1 isoform X18 [H	11	44	ic process; response to stimulus	1.018	0.972	0.944
7679817	PREDICTED: alpha-actinin-1 isoform X7 [Homo sapiens]	19	178	; regulation of biological process	1.084	0.972	1.018
182395	focal adhesion kinase [Homo sapiens]	2	3	; regulation of biological process	1.236	0.972	1.112
5457463	E3 ubiquitin-protein ligase TRIP12 isoform d [Homo sapiens]	7	14	; regulation of biological process	0.957	0.972	0.887
1826276	mediator complex subunit MED23 variant MED23_i8 [Homo sapien	2	5	gical process; metabolic process	0.992	0.972	0.793
7468160	tubulin beta-6 chain isoform 6 [Homo sapiens]	8	220	cell organization and biogenesis	0.988	0.971	0.860
1106247	band 4.1-like protein 4B isoform 1 [Homo sapiens]	2	4	biogenesis; response to stimulus	0.795	0.971	0.934
1195880	hCG2032701, isoform CRA_a [Homo sapiens]	15	81	metabolic process	1.194	0.971	1.013
1196170	achalasia, adrenocortical insufficiency, alacrimia (Allgrove, triple-A	6	16	; regulation of biological process	0.918	0.971	0.920
7679089	PREDICTED: digestive organ expansion factor homolog isoform X	2	4	metabolic process; development	0.871	0.971	1.008
3288459	transcription elongation factor TFIIS.h, partial (plasmid) [Homo sa	2	6	; regulation of biological process	0.995	0.970	0.883
1196116	cell division cycle 73, Paf1/RNA polymerase II complex component	9	24	biogenesis; response to stimulus	0.853	0.970	0.936
1181428	RSL1D1 protein, partial [Homo sapiens]	3	3	; regulation of biological process	1.014	0.969	1.053
5788009	PREDICTED: metaxin-1 isoform X1 [Homo sapiens]	2	10	transport; metabolic process	0.909	0.969	0.956
2816041	serine/threonine-protein phosphatase PGAM5, mitochondrial isofo	7	44	response to stimulus; cell death	0.960	0.969	0.945
5303984	PREDICTED: DCN1-like protein 5 isoform X2 [Homo sapiens]	3	7	; regulation of biological process	0.726	0.969	0.975
1195987	eukaryotic translation initiation factor 2B, subunit 5 epsilon, 82kDa	3	8	gical process; cell differentiation	1.058	0.969	0.960
1196247	methylmalonyl Coenzyme A mutase, isoform CRA_b [Homo sapien	3	9	metabolic process	0.728	0.969	0.911

1196298	phosphofructokinase, liver, isoform CRA_b [Homo sapiens]	21	129	cell organization and biogenesis	1.054	0.968	0.943
2817520	General transcription factor IIIC, polypeptide 3, 102kDa [Homo sap	6	10	metabolic process	1.027	0.968	0.928
5788311	PREDICTED: transmembrane protein 104 isoform X1 [Homo sapien	2	3		0.954	0.968	1.058
1196185	hCG2016250, isoform CRA_d [Homo sapiens]	11	71	metabolic process	0.985	0.967	0.990
2621184	Chain H, Mutation Of Thr163 To Ser In Human Thymidine Kinase S	4	13		0.915	0.967	0.899
1196078	RAB14, member RAS oncogene family, isoform CRA_a [Homo sap	10	101	cell organization and biogenesis	1.012	0.967	0.977
1890530	unnamed protein product [Homo sapiens]	3	19	ization and biogenesis; transport	0.913	0.967	0.998
338700	tat binding protein-1 (tbp-1) [Homo sapiens]	5	24	ent movement; defense response	0.985	0.967	0.965
3957594	Chain A, Crystal Structure Of Kap Beta2-Py-Nls	7	34	transport	0.984	0.967	0.969
1943847	unnamed protein product [Homo sapiens]	6	9	cell organization and biogenesis	1.046	0.967	1.058
4604172	actin-related protein 3 isoform 2 [Homo sapiens]	5	16	; cell division; defense response	1.111	0.966	0.958
1381027	phosphoribosypyrophosphate synthetase-associated protein 39 [H	5	14	; regulation of biological process	1.020	0.966	0.979
31092	unnamed protein product [Homo sapiens]	15	209	al process; response to stimulus	0.979	0.966	0.954
1544488	exportin-7 [Homo sapiens]	25	95	transport	1.079	0.965	0.969
1196298	hCG401289, isoform CRA_a [Homo sapiens]	7	37	tabolic process; cell proliferation	0.981	0.965	0.948
1943772	unnamed protein product [Homo sapiens]	7	33	metabolic process	1.096	0.965	0.938
1582592	unnamed protein product [Homo sapiens]	10	38	transport; metabolic process	1.123	0.965	1.030
5303669	PREDICTED: cleavage and polyadenylation specificity factor subu	7	19	metabolic process; transport	0.985	0.965	0.956
9296546	KIAA1838 protein [Homo sapiens]	2	6		0.881	0.965	0.983
3441898	Chain A, Crystal Structure Analysis Of H74a Mutant Of Human Clic	7	59		1.000	0.965	0.998
7468174	60S ribosomal protein L10 isoform c [Homo sapiens]	5	48	sis; metabolic process; transport	0.949	0.965	0.910
7680394	PREDICTED: cullin-4B isoform X5 [Homo sapiens]	16	53	; regulation of biological process	0.969	0.965	0.988
4825705	TALDO1 protein, partial [Homo sapiens]	16	105	metabolic process	1.190	0.965	0.929
1943827	unnamed protein product [Homo sapiens]	7	17	ic process; response to stimulus	1.024	0.964	0.913
1196224	calcium and integrin binding 1 (calmyrin), isoform CRA_a, partial [	2	3	tion and biogenesis; cell division	0.762	0.964	0.855
1943885	unnamed protein product [Homo sapiens]	3	5	mponent movement; cell division	1.321	0.964	1.078
1943787	unnamed protein product [Homo sapiens]	9	47	metabolic process	1.118	0.964	1.114
4594634	IARS2 protein, partial [Homo sapiens]	11	49	cell organization and biogenesis	1.038	0.963	0.979
1196033	glutamic-oxaloacetic transaminase 2, mitochondrial (aspartate am	12	74	; transport; response to stimulus	0.993	0.963	0.904
2234601	SYNJ1 protein [Homo sapiens]	2	2	; regulation of biological process	0.959	0.963	1.013
1196216	desmoglein 1 [Homo sapiens]	2	2	; regulation of biological process	0.951	0.963	1.300
7679851	PREDICTED: kunitz-type protease inhibitor 1 isoform X2 [Homo sa	2	6	cell organization and biogenesis	0.882	0.963	1.041
7679801	PREDICTED: endoribonuclease Dicer isoform X3 [Homo sapiens]	2	3	fense response; cell proliferation	0.953	0.963	0.889
1592985	QTRT1 protein [Homo sapiens]	3	5	metabolic process	1.057	0.963	0.855
5430440	unnamed protein product, partial [Homo sapiens]	2	2		1.093	0.963	0.846
4454524	similar to insulin receptor substrate BAP2; similar to PID:g4126477	5	13	al process; response to stimulus	0.852	0.963	0.907
1937884	unnamed protein product [Homo sapiens]	18	138	ic process; response to stimulus	0.964	0.962	0.954
1195748	hCG2024613, isoform CRA_a [Homo sapiens]	3	14	al process; response to stimulus	0.949	0.962	0.942
1584303	Chain A, Crystal Structure Of Human Rac3 In Complex With Crib D	4	29	al process; response to stimulus	0.936	0.962	0.999
5303935	PREDICTED: hexokinase-1 isoform X6 [Homo sapiens]	22	120	sis; metabolic process; transport	1.052	0.962	1.022

6674847	Chain O, Crystal Structure Of The Cop9 Signalosome	3	12		1.297	0.962	1.051
4035301	CSGlcA-T protein [Homo sapiens]	3	4	metabolic process	0.950	0.961	0.903
4003524	unnamed protein product [Homo sapiens]	5	13		0.967	0.961	0.919
1196283	hCG1984214, isoform CRA_a [Homo sapiens]	2	8	cell organization and biogenesis	0.996	0.961	0.917
2591553	mitochondrial 2-oxoglutarate/malate carrier protein isoform 2 [Hon	13	63	metabolic process; transport	1.026	0.961	0.939
5590984	stomatin-like protein 2, mitochondrial isoform b [Homo sapiens]	5	40	al process; response to stimulus	1.066	0.961	0.974
4506221	26S proteasome non-ATPase regulatory subunit 12 isoform 1 [Hon	9	30	ent movement; defense response	0.963	0.961	0.914
6055247	SSRP1 protein, partial [Homo sapiens]	8	58	; regulation of biological process	1.176	0.960	1.014
1195781	cat eye syndrome chromosome region, candidate 5, isoform CRA_	3	11	metabolic process	1.174	0.960	0.977
1890539	unnamed protein product [Homo sapiens]	9	58	ent movement; defense response	1.116	0.960	0.997
1943865	unnamed protein product [Homo sapiens]	17	88	biogenesis; cellular homeostasis	1.031	0.960	1.013
1196130	WD repeat domain 1, isoform CRA_a [Homo sapiens]	15	75	; regulation of biological process	1.052	0.960	0.929
4767848	dJ508I15.5 [Homo sapiens]	2	16	cell organization and biogenesis	1.372	0.960	0.914
3441790	neuroblastoma-amplified sequence, partial [Homo sapiens]	2	4	; regulation of biological process	0.998	0.959	0.827
1937873	unnamed protein product [Homo sapiens]	13	71	; transport; response to stimulus	1.218	0.959	0.983
1196194	eukaryotic translation initiation factor 2, subunit 3 gamma, 52kDa,	11	45	sis; metabolic process; transport	1.013	0.959	0.902
2624695	Chain B, Human Mitochondrial Single-Stranded Dna Binding Prote	5	38		1.218	0.959	0.987
1196029	tetracycline transporter-like protein, isoform CRA_a [Homo sapien	6	25	transport; cell death	0.890	0.959	1.016
7679259	PREDICTED: disks large homolog 1 isoform X10 [Homo sapiens]	4	10	n; cellular component movement	0.977	0.959	0.978
7679239	PREDICTED: protein SEC13 homolog isoform X5 [Homo sapiens]	4	40	cell organization and biogenesis	1.081	0.959	0.960
1195950	slingshot homolog 3 (Drosophila), isoform CRA_f [Homo sapiens]	8	22	; regulation of biological process	1.104	0.959	0.881
913174	TRPP, partial [Homo sapiens]	7	69	regulation of biological process	0.863	0.958	0.966
5147649	hypothetical protein [Homo sapiens]	6	15	ic process; response to stimulus	0.891	0.958	1.003
1195770	WD repeat domain 3, isoform CRA_a [Homo sapiens]	8	21	metabolic process	1.069	0.958	0.945
5303858	PREDICTED: beta-glucuronidase isoform X1 [Homo sapiens]	7	21	metabolic process	1.094	0.957	1.033
7679976	PREDICTED: structural maintenance of chromosomes flexible hing	11	31	; regulation of biological process	0.910	0.957	0.952
1195945	OTU domain, ubiquitin aldehyde binding 1, isoform CRA_c [Homo	6	39	se to stimulus; defense response	1.209	0.956	0.972
3342734	NUKM_HUMAN, partial CDS, partial [Homo sapiens]	4	12	cell organization and biogenesis	1.254	0.956	0.984
1582598	unnamed protein product [Homo sapiens]	4	25	transport	0.962	0.956	0.976
1196152	KIAA0090, isoform CRA_b [Homo sapiens]	14	47	metabolic process	1.073	0.956	0.942
1195916	ATG4 autophagy related 4 homolog B (S. cerevisiae), isoform CRA	2	4	; regulation of biological process	0.927	0.956	0.939
1205282	hypothetical protein [Homo sapiens]	9	102	nd biogenesis; cell differentiation	1.203	0.955	1.046
1196038	component of oligomeric golgi complex 5, isoform CRA_a [Homo s	11	32	transport	0.912	0.955	0.904
2343185	tubulin folding cofactor B [Homo sapiens]	7	51	lifferentiation; metabolic process	1.145	0.955	0.920
4825711	RIC8A protein, partial [Homo sapiens]	6	20	cell organization and biogenesis	1.065	0.955	0.961
1195966	acyl-CoA synthetase short-chain family member 2, isoform CRA_c	8	13	metabolic process	0.913	0.955	0.854
1195695	antigen identified by monoclonal antibody Ki-67, isoform CRA_c [H	2	3	oliferation; response to stimulus	0.864	0.955	0.915
1195845	transmembrane protein 43, isoform CRA_b, partial [Homo sapiens]	5	19	cell organization and biogenesis	1.074	0.954	0.944
7020317	unnamed protein product [Homo sapiens]	3	7	ess; transport; cell differentiation	0.944	0.954	0.923
1575347	HU-K4 [Homo sapiens]	3	4	metabolic process	1.032	0.954	1.094

1002369	coatomer protein [Homo sapiens]	40	217	cell organization and biogenesis	1.069	0.954	0.915
5107857	integrin-linked protein kinase isoform 2 [Homo sapiens]	4	4	cell organization and biogenesis	0.863	0.953	0.930
9381488	DNA-directed RNA polymerases I, II, and III subunit RPABC1 isofor	4	13		0.877	0.952	0.939
1890652	unnamed protein product [Homo sapiens]	3	10	ic process; response to stimulus	0.900	0.952	0.913
1136398	KIAA0169 protein, partial [Homo sapiens]	8	13	al process; response to stimulus	0.957	0.952	0.955
5207286	Chain B, Crystal Structure Of Recombinant Human Hexokinase Ty	22	118		1.092	0.952	1.017
8874929	Chain a, Structure Of The Hcv Ires Bound To The Human Ribosom	2	28		1.259	0.951	0.969
1943905	unnamed protein product [Homo sapiens]	4	7	tion and biogenesis; cell division	1.051	0.951	1.122
2210424	unnamed protein product [Homo sapiens]	2	28	; regulation of biological process	1.613	0.951	0.984
1337772	RPS4Y1 protein, partial [Homo sapiens]	11	77	genesis; transport; development	1.015	0.951	0.942
1196176	RAB21, member RAS oncogene family, isoform CRA_a [Homo sap	4	14	s; cellular component movement	1.058	0.951	0.910
1937878	unnamed protein product [Homo sapiens]	2	3	metabolic process	0.693	0.950	1.013
1943902	unnamed protein product [Homo sapiens]	15	59	cell organization and biogenesis	1.080	0.950	0.886
7679777	PREDICTED: mitochondrial intermediate peptidase isoform X1 [Ho	7	14	metabolic process	0.938	0.950	0.927
3387836	Isochorismatase domain containing 1 [Homo sapiens]	3	9	metabolic process	1.201	0.950	0.975
1196279	tyrosyl-tRNA synthetase, isoform CRA_a [Homo sapiens]	10	29	al process; response to stimulus	1.094	0.950	1.098
2175018	unnamed protein product [Homo sapiens]	16	184	al process; response to stimulus	1.004	0.950	0.984
1943809	unnamed protein product [Homo sapiens]	18	70	cell organization and biogenesis	1.113	0.950	1.028
4004297	unnamed protein product, partial [Homo sapiens]	2	8		1.513	0.950	1.058
1890654	unnamed protein product [Homo sapiens]	5	59	; regulation of biological process	1.214	0.949	0.945
1195846	mitochondrial ribosomal protein S25, isoform CRA_a [Homo sapie	2	3	d biogenesis; metabolic process	1.011	0.949	1.004
1196051	hCG2040050, isoform CRA_a [Homo sapiens]	2	4	al process; response to stimulus	0.815	0.949	0.948
1195920	exosome component 10, isoform CRA_c [Homo sapiens]	4	5	; regulation of biological process	0.768	0.949	0.981
1195740	hCG2002711, isoform CRA_b [Homo sapiens]	6	21	metabolic process	0.958	0.948	0.996
5453838	Sjoegren syndrome/scleroderma autoantigen 1 isoform 1 [Homo s	2	5	tion and biogenesis; cell division	1.136	0.948	0.931
1195714	phosphatidylinositol glycan, class S, isoform CRA_a [Homo sapien	7	30	metabolic process	0.826	0.948	0.932
1318308	steroid dehydrogenase-like protein [Homo sapiens]	4	13	metabolic process	1.065	0.948	0.934
3001932	Chain B, The Structure Of Human Prolylcarboxypeptidase At 2.80 A	3	6	metabolic process	0.998	0.947	0.791
1937850	unnamed protein product [Homo sapiens]	18	62	lifferentiation; metabolic process	0.917	0.947	0.940
5630086	lysyl hydroxylase 3 [Homo sapiens]	10	28	ic process; response to stimulus	0.943	0.946	0.910
1196111	uridine-cytidine kinase 2, isoform CRA_b [Homo sapiens]	2	2	metabolic process	0.992	0.946	0.896
1893108	truncated E-cadherin [Homo sapiens]	6	13	e to stimulus; metabolic process	1.035	0.946	1.115
1196313	DnaJ (Hsp40) homolog, subfamily C, member 10, isoform CRA_d [	2	3	o stimulus; cellular homeostasis	0.680	0.946	0.796
7679931	PREDICTED: proteasome activator complex subunit 3 isoform X1 [	4	25	ent movement; defense response	1.196	0.946	0.980
1891683	KIAA1970 protein, partial [Homo sapiens]	7	20	metabolic process	0.941	0.946	0.928
3154316	THO complex subunit 6 homolog isoform 1 [Homo sapiens]	6	28	; regulation of biological process	0.988	0.946	0.939
1083477	PNAS-107 [Homo sapiens]	6	34	erentiation; response to stimulus	1.102	0.946	0.969
1196254	ATP-binding cassette, sub-family E (OABP), member 1 [Homo sapi	6	24	n of biological process; transport	1.105	0.946	1.154
1195769	hCG2043376, isoform CRA_b [Homo sapiens]	3	25	cell organization and biogenesis	1.053	0.946	0.924
5273173	tRNA (adenine(58)-N(1))-methyltransferase non-catalytic subunit T	3	11	metabolic process	0.919	0.946	0.890

1943837	unnamed protein product [Homo sapiens]	4	108	cell organization and biogenesis	1.103	0.945	0.973
2146596	Chain E, Structure Of The Human Replication Protein A (Rpa) Trim	2	7		1.081	0.945	0.956
1195948	SCY1-like 1 (S. cerevisiae), isoform CRA_f [Homo sapiens]	2	4	n of biological process; transport	1.006	0.945	0.829
1260308	Chain B, Human Mitochondrial Beta-ketoacyl Acp Synthase	2	5	metabolic process	0.961	0.945	0.834
9903162	Chain B, Crystal Structure Of Homo Sapien Glycerol-3-Phosphate I	2	6	metabolic process	1.140	0.945	1.018
1890533	unnamed protein product [Homo sapiens]	5	26	metabolic process	0.940	0.944	0.938
7679949	PREDICTED: peroxisomal acyl-coenzyme A oxidase 1 isoform X2 [	6	20	; regulation of biological process	1.010	0.944	0.945
7679034	PREDICTED: E3 ubiquitin-protein ligase UBR4 isoform X14 [Homo	26	61	metabolic process	0.961	0.944	0.975
6270233	unknown [Homo sapiens]	5	22	lifferentiation; metabolic process	1.004	0.944	0.972
1195916	septin 2, isoform CRA_a [Homo sapiens]	7	39	sponse to stimulus; cell division	1.207	0.944	1.020
1196014	hCG2028724 [Homo sapiens]	11	68		0.991	0.944	1.001
1196261	vesicle docking protein p115, isoform CRA_b [Homo sapiens]	14	71	cell organization and biogenesis	1.143	0.944	0.927
9296547	MYO1C variant protein [Homo sapiens]	29	130		1.086	0.943	0.898
9311398	Chain C, Crystal Structure Of Pro1 Deletion Mutant Of Human Maci	2	12		1.226	0.943	0.753
5411211	splicing factor 3B subunit 1 isoform 1 [Homo sapiens]	18	67	al process; response to stimulus	0.817	0.943	0.969
1244942	unconventional myosin-Ic isoform b [Homo sapiens]	29	135	se to stimulus; defense response	1.074	0.943	0.901
1195715	suppressor of Ty 6 homolog (S. cerevisiae), isoform CRA_a [Homo	9	29	n of biological process; transport	0.962	0.943	1.032
1937864	unnamed protein product [Homo sapiens]	3	9	d biogenesis; metabolic process	0.829	0.943	0.918
1043464	unnamed protein product [Homo sapiens]	8	26	bolic process; cell differentiation	1.055	0.943	0.909
4003751	unnamed protein product, partial [Homo sapiens]	3	5		0.983	0.943	0.997
7679089	PREDICTED: TIP41-like protein isoform X1 [Homo sapiens]	3	6	; regulation of biological process	1.039	0.942	0.984
1195888	coatomer protein complex, subunit beta, isoform CRA_a [Homo sa	15	76	cell organization and biogenesis	1.049	0.942	0.960
4004044	unnamed protein product, partial [Homo sapiens]	11	91		0.990	0.942	1.011
3002382	protein FAM134C [Homo sapiens]	3	15	regulation of biological process	1.171	0.942	1.018
1890531	unnamed protein product [Homo sapiens]	10	45	ization and biogenesis; transport	1.085	0.941	0.967
4004194	unnamed protein product, partial [Homo sapiens]	2	10		1.309	0.941	1.007
1196243	peptidylprolyl isomerase (cyclophilin)-like 1, isoform CRA_a [Homo	4	16	metabolic process	0.937	0.941	0.927
1277991	Aminopeptidase puromycin sensitive [Homo sapiens]	24	214	ic process; response to stimulus	0.954	0.941	0.924
1943746	unnamed protein product [Homo sapiens]	4	25	metabolic process	1.077	0.940	0.833
6208818	NACHT, leucine rich repeat and PYD containing 2 variant, partial [H	3	7	; regulation of biological process	0.858	0.940	0.905
3003605	actin-related protein 2/3 complex subunit 1A isoform 2 [Homo sapi	9	27	nd biogenesis; defense response	1.049	0.939	0.866
1196237	tubulin, beta, isoform CRA_b [Homo sapiens]	13	394	sponse to stimulus; cell division	0.987	0.939	0.918
1196273	peroxiredoxin 1, isoform CRA_b, partial [Homo sapiens]	9	226	gical process; defense response	0.962	0.939	1.002
9040302	AP1B1 [Homo sapiens]	17	63	cell organization and biogenesis	1.051	0.939	0.910
1195887	tumor susceptibility gene 101, isoform CRA_e [Homo sapiens]	3	25	; cell differentiation; cell division	1.097	0.939	0.965
5788212	PREDICTED: atlastin-3 isoform X3 [Homo sapiens]	5	13	d biogenesis; metabolic process	1.132	0.939	1.064
5304103	PREDICTED: Golgi apparatus membrane protein TVP23 homolog E	2	4	transport	0.994	0.938	0.850
7561430	Chain A, Crystal Structure Of Autophagic Snare Complex	3	12		0.996	0.938	0.933
1937877	unnamed protein product [Homo sapiens]	5	15	gical process; metabolic process	1.019	0.938	0.861
7679204	PREDICTED: cell differentiation protein RCD1 homolog isoform X1	2	3	al process; response to stimulus	1.057	0.938	0.952

1195728	glycogen synthase 1 (muscle), isoform CRA_a [Homo sapiens]	10	40	metabolic process	0.997	0.938	0.880
7679898	PREDICTED: FH1/FH2 domain-containing protein 1 isoform X4 [Ho	10	19	regulation of biological process	0.950	0.937	0.875
1195821	dynactin 4 (p62), isoform CRA_b [Homo sapiens]	2	5		0.875	0.937	0.933
1195986	transmembrane protein 41A, isoform CRA_d [Homo sapiens]	2	8		1.226	0.937	0.911
1195734	death associated protein 3, isoform CRA_e [Homo sapiens]	4	14	al process; response to stimulus	1.117	0.937	0.994
5051326	Chain A, Crystal Structure Of The Aminoacylation Catalytic Fragme	4	19	metabolic process	0.888	0.937	0.906
1943846	unnamed protein product [Homo sapiens]	3	10	l differentiation; cell proliferation	1.313	0.937	1.005
4564448	ADAM15 isoform 1 [Homo sapiens]	7	19	nd biogenesis; defense response	1.109	0.936	0.899
7679096	PREDICTED: protein odr-4 homolog isoform X3 [Homo sapiens]	3	7		1.111	0.936	1.014
1732421	C2f [Homo sapiens]	3	9	metabolic process	0.957	0.936	0.835
5435837	ubiquitin-conjugating enzyme E2 variant 1 isoform g [Homo sapien	5	14	differentiation; defense response	1.000	0.936	0.913
1455796	Chain D, Crystallographic And Kinetic Studies Of Human Mitochon	9	38	metabolic process	0.870	0.936	1.051
1195793	ring finger protein 20, isoform CRA_a [Homo sapiens]	3	4	cell organization and biogenesis	1.047	0.936	1.020
3742537	ethanolamine-phosphate cytidylyltransferase isoform 5 [Homo sap	6	16	metabolic process	0.963	0.935	0.849
7679254	PREDICTED: multidrug resistance-associated protein 5 isoform X2	2	2	metabolic process; transport	0.990	0.935	0.838
1196200	dynactin 1 (p150, glued homolog, Drosophila), isoform CRA_a [Ho	12	43	e to stimulus; metabolic process	1.083	0.935	0.925
1943879	unnamed protein product [Homo sapiens]	21	94	; regulation of biological process	1.076	0.935	0.958
3544593	ras-related protein Rap-1b isoform 4 [Homo sapiens]	3	22	cell organization and biogenesis	1.380	0.935	1.106
1890654	unnamed protein product [Homo sapiens]	10	123	cell organization and biogenesis	1.030	0.935	0.976
1582560	unnamed protein product [Homo sapiens]	20	450	; regulation of biological process	1.172	0.935	0.945
1196124	family with sequence similarity 91, member A1, isoform CRA_b [Ho	4	7		1.026	0.935	0.865
2792375	RecName: Full=Cingulin	7	15	e to stimulus; metabolic process	0.918	0.935	0.860
1195808	hypothetical protein LOC63929, isoform CRA_b [Homo sapiens]	3	29	metabolic process	0.729	0.934	0.924
5788144	PREDICTED: replication factor C subunit 2 isoform X1 [Homo sapi	2	4	o stimulus; cellular homeostasis	1.091	0.934	0.949
1195826	SAR1 gene homolog B (S. cerevisiae), isoform CRA_a [Homo sapie	3	7	cell organization and biogenesis	0.952	0.934	0.952
1196123	TAF2 RNA polymerase II, TATA box binding protein (TBP)-associat	3	5	cell organization and biogenesis	0.855	0.934	0.943
1196036	DEAD (Asp-Glu-Ala-Asp) box polypeptide 28 [Homo sapiens]	4	6	cell organization and biogenesis	0.861	0.934	0.903
1976924	autoantigen La, partial [Homo sapiens]	5	30	metabolic process	0.971	0.934	0.938
3817445	MED1 protein [Homo sapiens]	2	6	l differentiation; cell proliferation	0.864	0.934	0.947
1895027	mitochondrial heat shock 60kD protein 1 variant 1 [Homo sapiens]	17	187	cell organization and biogenesis	0.964	0.934	0.921
5443463	transformer-2 protein homolog alpha isoform 3 [Homo sapiens]	3	42	metabolic process	1.003	0.934	0.910
1943880	unnamed protein product [Homo sapiens]	19	436	al process; response to stimulus	1.174	0.934	0.944
2037906	small GTP binding protein RAB7 [Homo sapiens]	11	65	biogenesis; cellular homeostasis	1.220	0.934	0.890
1196007	growth hormone inducible transmembrane protein, isoform CRA_	4	11	cell death	1.067	0.934	0.942
1943799	unnamed protein product [Homo sapiens]	3	6	f biological process; cell division	0.777	0.934	0.954
7679908	PREDICTED: Y+L amino acid transporter 2 isoform X2 [Homo sapi	2	3	n; cellular component movement	1.006	0.933	0.891
5788333	PREDICTED: dual specificity mitogen-activated protein kinase kina	3	3	fense response; cell proliferation	1.094	0.933	0.946
1943784	unnamed protein product [Homo sapiens]	2	15	ent movement; defense response	0.948	0.932	1.047
1196141	chromosome 7 open reading frame 30 [Homo sapiens]	2	3	regulation of biological process	1.374	0.932	0.989
7418660	CWF19-like protein 1 isoform 2 [Homo sapiens]	2	3	metabolic process	1.162	0.932	0.887

5768663	cytochrome c oxidase subunit II, partial (mitochondrion) [Homo sa	2	16	transport; metabolic process	0.724	0.932	0.967
1196295	hypothetical protein FLJ11305, isoform CRA_b [Homo sapiens]	2	5	regulation of biological process	0.800	0.932	0.978
1008915	proteasome activator hPA28 suunit beta [Homo sapiens]	6	34	ent movement; defense response	1.019	0.932	1.074
1195991	phospholipase C, eta 1, isoform CRA_c [Homo sapiens]	2	4	al process; response to stimulus	0.897	0.932	0.987
7679429	PREDICTED: pre-mRNA-processing factor 17 isoform X1 [Homo sa	3	8	sis; metabolic process; transport	1.215	0.932	1.034
4004431	unnamed protein product, partial [Homo sapiens]	3	3		1.100	0.932	0.961
1195740	guanine nucleotide binding protein (G protein), beta polypeptide 2	16	185	velopment; cellular homeostasis	1.077	0.932	0.932
1195796	adaptor-related protein complex 1, gamma 1 subunit, isoform CRA	13	46	; regulation of biological process	1.112	0.932	0.945
7680400	PREDICTED: sterol-4-alpha-carboxylate 3-dehydrogenase, decarbo	8	39	al process; response to stimulus	1.110	0.931	1.075
1937841	unnamed protein product [Homo sapiens]	6	16	development; cell differentiation	0.940	0.931	0.988
7679270	PREDICTED: ATP-binding cassette sub-family F member 3 isoform	8	25	e response; response to stimulus	1.073	0.931	0.976
1260313	Chain A, Crystal Structure Of Engineered Thimet Oligopeptidase W	4	12	metabolic process	0.997	0.931	0.892
7680333	PREDICTED: ubiquitin-like modifier-activating enzyme 1 isoform X	26	225	response to stimulus; cell death	1.083	0.931	0.950
3532518	Chain G, The Crystal Structure Of The Catalytic Domain Of Amsh	2	12		0.882	0.931	0.933
2210439	unnamed protein product [Homo sapiens]	2	2	gical process; cell differentiation	0.700	0.931	1.111
7679902	PREDICTED: vacuolar protein sorting-associated protein 35 isofor	12	64	; regulation of biological process	1.012	0.931	0.976
4098252	unnamed protein product, partial [Homo sapiens]	5	28		0.975	0.931	0.953
7679742	PREDICTED: leukotriene A-4 hydrolase isoform X2 [Homo sapiens	10	35	se to stimulus; cell differentiation	1.027	0.930	0.946
1404269	unnamed protein product [Homo sapiens]	6	17	se to stimulus; defense response	0.929	0.930	0.864
1415014	protein syndesmos isoform 1 [Homo sapiens]	4	9	metabolic process	1.530	0.930	1.026
2605936	xaa-Pro dipeptidase isoform 2 [Homo sapiens]	5	14	metabolic process	0.821	0.930	0.936
1393980	unnamed protein product [Homo sapiens]	4	7		0.862	0.930	0.908
1195996	coatomer protein complex, subunit gamma, isoform CRA_a [Homo	17	80	s; cellular component movement	1.050	0.929	0.926
1943835	unnamed protein product [Homo sapiens]	4	12	d biogenesis; metabolic process	0.957	0.929	0.860
1196241	ribosomal protein S18, isoform CRA_c [Homo sapiens]	6	113	ization and biogenesis; transport	1.172	0.929	0.956
1879601	ubiquitin-protein ligase E3C [Homo sapiens]	10	29	metabolic process	1.015	0.929	0.841
1043607	RNA binding motif protein 8A [Homo sapiens]	3	11	n of biological process; transport	0.868	0.929	0.873
1280463	Golgi membrane protein 1 [Homo sapiens]	7	33	; regulation of biological process	0.936	0.928	0.931
1562087	KIAA1907 protein, partial [Homo sapiens]	3	6	l proliferation; cell differentiation	0.982	0.927	0.843
5304102	PREDICTED: dehydrogenase/reductase SDR family member 7B iso	8	22	metabolic process	0.988	0.927	0.883
1196087	tubulin, beta 2C, isoform CRA_b [Homo sapiens]	20	515	e response; response to stimulus	1.023	0.927	0.911
1196036	cirrhosis, autosomal recessive 1A (cirhin), isoform CRA_g [Homo	5	11	; regulation of biological process	1.115	0.927	0.876
4035280	PDCD11 protein, partial [Homo sapiens]	8	16	metabolic process	1.011	0.927	0.910
1639311	Chain H, Crystal Structure Of Human Short-Chain Acyl Coa Dehydr	5	8	metabolic process	1.030	0.927	0.874
5788081	PREDICTED: LETM1 and EF-hand domain-containing protein 1, mi	7	27	cell organization and biogenesis	1.070	0.926	1.021
5304029	PREDICTED: poly(A) polymerase alpha isoform X2 [Homo sapiens]	3	5	; regulation of biological process	0.981	0.926	0.946
1890652	unnamed protein product [Homo sapiens]	5	34	; regulation of biological process	0.862	0.926	0.911
1196320	aspartyl-tRNA synthetase, isoform CRA_b [Homo sapiens]	21	135	cell organization and biogenesis	1.022	0.926	0.910
1193723	beta-galactosidase isoform b [Homo sapiens]	10	37	metabolic process	1.100	0.926	0.927
1196200	ancient ubiquitous protein 1, isoform CRA_a [Homo sapiens]	6	9	; regulation of biological process	1.084	0.926	0.850

8923892	peroxisomal membrane protein 2 [Homo sapiens]	4	17		0.832	0.925	0.932
1890653	unnamed protein product [Homo sapiens]	10	60	cell organization and biogenesis	1.176	0.925	0.957
4524056	succinate dehydrogenase [ubiquinone] cytochrome b small subun	2	5	metabolic process	0.854	0.925	0.932
3478285	CCDC47 protein, partial [Homo sapiens]	6	21	d biogenesis; metabolic process	1.021	0.925	0.885
1195883	zinc finger protein 289, ID1 regulated, isoform CRA_e [Homo sapie	6	14	; regulation of biological process	1.081	0.925	0.998
4842547	Chain C, Structure Of The Signal Recognition Particle Interacting V	5	22	; regulation of biological process	1.155	0.925	0.900
1943819	unnamed protein product [Homo sapiens]	6	11	al process; response to stimulus	0.961	0.925	0.828
1195786	hypothetical protein LOC144501, isoform CRA_c [Homo sapiens]	3	16	ization and biogenesis; transport	1.065	0.924	1.325
7200628	Chain A, Crystal Structure Of Ythdf1 Yth Domain In Complex With	2	5		1.179	0.924	1.006
1582614	unnamed protein product [Homo sapiens]	16	60	ent movement; defense response	1.160	0.924	0.958
7680025	PREDICTED: unconventional myosin-IXb isoform X8 [Homo sapien	2	2	cell organization and biogenesis	0.826	0.924	0.944
7679221	PREDICTED: E3 ubiquitin-protein ligase ARIH2 isoform X6 [Homo s	2	4	ess; cell growth; cell proliferation	1.090	0.924	0.943
1043666	unnamed protein product [Homo sapiens]	3	10	; regulation of biological process	0.836	0.923	0.926
4636255	Splicing factor 3b, subunit 3, 130kDa [Homo sapiens]	32	161	; regulation of biological process	1.084	0.923	0.921
1195721	DnaJ (Hsp40) homolog, subfamily C, member 16, isoform CRA_a [I	2	6	; regulation of biological process	0.908	0.923	0.904
1582558	unnamed protein product [Homo sapiens]	9	24	; defense response; cell division	1.035	0.922	0.919
5303875	PREDICTED: erlin-2 isoform X1 [Homo sapiens]	5	28	al process; response to stimulus	1.171	0.922	0.889
6094126	Chain A, Crystal Structure Of Human Mlh1	4	11	ic process; response to stimulus	0.943	0.922	0.928
1195995	signal recognition particle receptor, B subunit, isoform CRA_b [Ho	5	37	al process; response to stimulus	1.080	0.922	0.928
913159	neuropolypeptide h3 [human, brain, Peptide, 186 aa]	7	42		1.109	0.922	0.961
2210447	unnamed protein product [Homo sapiens]	5	12	metabolic process; transport	1.053	0.922	0.949
6808223	hypothetical protein, partial [Homo sapiens]	2	3	; regulation of biological process	1.147	0.921	0.985
1808871	Tubulin, beta [Homo sapiens]	20	542	sponse to stimulus; cell division	1.032	0.921	0.921
6208765	myosin head domain containing 1 variant, partial [Homo sapiens]	2	3	metabolic process	1.040	0.921	0.805
3611325	Chain A, Structure Of Ddb1-Ddb2-Cul4a-Rbx1 Bound To A 12 Bp A	7	21	metabolic process	0.956	0.921	1.004
1196108	MYB binding protein (P160) 1a, isoform CRA_b [Homo sapiens]	15	55	nunication; response to stimulus	1.014	0.921	0.968
2210406	unnamed protein product [Homo sapiens]	4	6	cell organization and biogenesis	0.886	0.921	0.927
5633175	FGFR2-AHCYL1 fusion kinase protein [Homo sapiens]	11	57	onse; transport; cell proliferation	0.970	0.921	0.974
7679093	PREDICTED: keratinocyte proline-rich protein isoform X1 [Homo sa	3	6		0.893	0.921	1.610
1582582	unnamed protein product [Homo sapiens]	18	70	metabolic process	0.986	0.920	0.843
7679697	PREDICTED: probable ATP-dependent RNA helicase DDX10 isofor	3	4	metabolic process	1.002	0.920	0.962
1278027	Protein kinase C, iota [Homo sapiens]	2	13	movement; response to stimulus	1.126	0.920	0.959
1195735	dolichyl-phosphate mannosyltransferase polypeptide 3, isoform Cl	2	17	; regulation of biological process	1.121	0.920	0.881
7612327	Chain A, Complex Of Human Eif4e With The 4e Binding Protein 4e-	3	6		0.954	0.920	0.884
2817509	Similar to ring finger protein 40, partial [Homo sapiens]	5	7	biogenesis; response to stimulus	0.915	0.919	0.960
1196106	hCG1987397, isoform CRA_c [Homo sapiens]	2	6	; regulation of biological process	0.938	0.919	0.943
7422612	Chain q, Minimal U1 Snrnp	3	27		1.137	0.919	0.945
7680216	PREDICTED: splicing factor U2AF 35 kDa subunit isoform X1 [Hom	4	56	; regulation of biological process	1.093	0.919	0.942
1143254	YRDC protein, partial [Homo sapiens]	3	6	regulation of biological process	1.005	0.919	0.897
7679227	PREDICTED: neurobeachin-like protein 2 isoform X5 [Homo sapien	4	7	cell organization and biogenesis	0.889	0.919	0.929

1943858	unnamed protein product [Homo sapiens]	3	7	al process; response to stimulus	0.817	0.919	0.852
1195932	B-cell receptor-associated protein 31, isoform CRA_a [Homo sapie	8	43	; regulation of biological process	0.985	0.919	0.922
1943762	unnamed protein product [Homo sapiens]	8	65	metabolic process	0.947	0.919	0.943
7561425	Chain s, Human Constitutive 20s Proteasome In Complex With Car	5	18		0.898	0.919	0.901
3306895	Chain A, Crystal Structure Of Human Flap Endonuclease Fen1 (Wt)	3	12	ic process; response to stimulus	0.880	0.919	1.042
1582566	unnamed protein product [Homo sapiens]	27	883	ent movement; defense response	1.227	0.919	0.995
1196001	CD47 antigen (Rh-related antigen, integrin-associated signal trans	3	11	s; cellular component movement	1.051	0.919	1.023
3848920	membrane glycoprotein gp140, partial [Homo sapiens]	7	18		0.981	0.919	0.884
1195798	ubiquitin specific peptidase 9, X-linked, isoform CRA_d [Homo sap	12	36	imulus; cell growth; cell division	1.026	0.918	0.968
2210395	unnamed protein product [Homo sapiens]	6	7	cell organization and biogenesis	1.138	0.918	0.970
1195931	GDP dissociation inhibitor 1, isoform CRA_b [Homo sapiens]	15	160	us; transport; metabolic process	1.157	0.918	0.888
1890653	unnamed protein product [Homo sapiens]	6	16	ic process; response to stimulus	1.146	0.918	0.958
5187398	LRP10 protein, partial [Homo sapiens]	2	2	transport	0.902	0.918	0.909
7561425	Chain q, Human Constitutive 20s Proteasome In Complex With Car	8	50		0.929	0.917	1.039
7679377	PREDICTED: leucine--tRNA ligase, cytoplasmic isoform X3 [Homo	12	50	; regulation of biological process	1.115	0.917	0.958
3232766	COMMD3-BMI1 read-through protein [Homo sapiens]	2	6		1.313	0.917	1.051
1195960	protein tyrosine phosphatase, non-receptor type 1, isoform CRA_b	6	16	cell organization and biogenesis	0.887	0.917	0.944
1195821	hCG1783090 [Homo sapiens]	12	116	metabolic process	0.993	0.917	0.879
5304185	PREDICTED: transmembrane 9 superfamily member 4 isoform X1 [	8	41		1.056	0.916	0.976
1196161	mitochondrial ribosomal protein S27, isoform CRA_b [Homo sapie	4	7	d biogenesis; metabolic process	0.946	0.916	0.896
1196070	ubiquitin-conjugating enzyme E2 variant 2, isoform CRA_c [Homo	4	13	logical process; cell proliferation	0.958	0.916	0.870
340188	H+ -ATPase C subunit, partial [Homo sapiens]	4	11	cell organization and biogenesis	1.002	0.916	0.906
7679580	PREDICTED: serine/threonine-protein kinase WNK2 isoform X17 [H	2	3	al process; response to stimulus	0.728	0.916	0.954
3819720	RPN2 protein, partial [Homo sapiens]	13	107	; transport; response to stimulus	1.138	0.916	0.992
7680103	PREDICTED: nectin-2 isoform X1 [Homo sapiens]	4	6	al process; response to stimulus	0.859	0.916	0.953
5303781	PREDICTED: N-alpha-acetyltransferase 15, NatA auxiliary subunit i	7	30	; regulation of biological process	1.026	0.916	0.830
1195986	proteasome (prosome, macropain) 26S subunit, non-ATPase, 2, isc	24	121	ent movement; defense response	1.061	0.914	0.904
5595689	keratin, type I cytoskeletal 9 [Homo sapiens]	25	144	cell organization and biogenesis	1.007	0.914	2.032
7679601	PREDICTED: phosphatidylinositol 5-phosphate 4-kinase type-2 alp	2	6	; regulation of biological process	1.376	0.914	1.012
1196190	implantation-associated protein, isoform CRA_b [Homo sapiens]	5	26	; regulation of biological process	1.015	0.914	0.897
7679351	PREDICTED: cyclin-dependent kinase 7 isoform X1 [Homo sapiens	2	6	us; cell proliferation; cell division	0.809	0.913	0.932
6208903	chaperonin containing TCP1, subunit 6A isoform a variant, partial	15	130	metabolic process	1.004	0.913	0.903
3106899	Chain A, Crystal Structure Of Cugbp1 Rrm1/2-rna Complex	4	17		0.897	0.913	0.841
1804460	RPL14 protein [Homo sapiens]	4	46	ization and biogenesis; transport	0.939	0.913	0.881
1229205	Chain F, Crystal Structure Of A Protein Phosphatase 2a (Pp2a) Hol	8	63		1.199	0.913	0.885
1890536	unnamed protein product [Homo sapiens]	12	62	cell organization and biogenesis	1.050	0.913	0.933
1311180	Unknown (protein for IMAGE:2901253), partial [Homo sapiens]	5	16	metabolic process	0.975	0.912	1.094
2817559	FARSA protein, partial [Homo sapiens]	6	15	metabolic process	0.947	0.912	1.030
1943743	unnamed protein product [Homo sapiens]	2	5	cell organization and biogenesis	1.024	0.912	0.987
1196139	mitogen-activated protein kinase-activated protein kinase 2, isofor	3	7	lus; defense response; transport	0.789	0.912	0.981

5788042	PREDICTED: integrin alpha-6 isoform X2 [Homo sapiens]	6	14	n; cellular component movement	0.929	0.912	0.895
1196074	transmembrane protein 70, isoform CRA_a [Homo sapiens]	3	5	cell organization and biogenesis	1.052	0.912	0.919
1195864	importin 4, isoform CRA_d [Homo sapiens]	12	57	ization and biogenesis; transport	1.050	0.912	0.928
1861867	unnamed protein product [Homo sapiens]	7	20		0.994	0.912	0.874
1196047	nuclear factor I/X (CCAAT-binding transcription factor), isoform CF	4	30	bolic process; cell differentiation	0.980	0.912	1.108
7679652	PREDICTED: protein arginine N-methyltransferase 3 isoform X1 [H	2	8	cell organization and biogenesis	0.952	0.911	0.866
1943777	unnamed protein product [Homo sapiens]	5	13		0.839	0.911	0.867
1196174	Ts translation elongation factor, mitochondrial, isoform CRA_d [Ho	5	13	; regulation of biological process	1.155	0.911	0.920
7020356	unnamed protein product [Homo sapiens]	7	24		1.043	0.911	0.899
1195818	nucleophosmin (nucleolar phosphoprotein B23, numatrin), isoform	8	76	; regulation of biological process	1.046	0.911	0.940
5304014	PREDICTED: N-alpha-acetyltransferase 25, NatB auxiliary subunit i	4	8	metabolic process	0.933	0.911	0.885
2210424	unnamed protein product [Homo sapiens]	4	9	al process; response to stimulus	1.081	0.911	0.932
2195179	GEMIN5 protein [Homo sapiens]	6	12	d biogenesis; metabolic process	0.900	0.910	0.949
1710248	protein disulfide isomerase-related protein 5, partial [Homo sapien	7	129	o stimulus; cellular homeostasis	1.167	0.910	0.941
3469863	bis(5'-nucleosyl)-tetraphosphatase [asymmetrical] [Homo sapiens]	2	4	us; metabolic process; cell death	0.946	0.910	0.923
1196276	poly(A) binding protein, cytoplasmic 4 (inducible form), isoform CF	16	94	metabolic process; coagulation	1.065	0.910	0.913
28876	ash protein, partial [Homo sapiens]	2	8	sis; transport; defense response	0.921	0.910	0.874
1890654	unnamed protein product [Homo sapiens]	27	322	ization and biogenesis; transport	1.226	0.910	0.901
4826769	Chain D, Nucleotide-free Dynamin 1-like Protein (dnm1l, Drp1, Dlp1	13	30		0.974	0.910	0.943
7679641	PREDICTED: sphingosine-1-phosphate lyase 1 isoform X2 [Homo s	10	30	al process; response to stimulus	1.065	0.910	0.962
1196147	coiled-coil domain containing 44, isoform CRA_b [Homo sapiens]	2	3	regulation of biological process	0.882	0.910	0.966
4768831	unknown [Homo sapiens]	2	8	transport; metabolic process	0.923	0.909	0.938
1195769	DEAD (Asp-Glu-Ala-Asp) box polypeptide 20, isoform CRA_b [Hom	3	8	d biogenesis; metabolic process	0.901	0.909	0.894
1196171	heterogeneous nuclear ribonucleoprotein A1, isoform CRA_e [Hom	11	111	metabolic process; transport	1.131	0.909	1.102
3262052	ATP-dependent RNA helicase DDX1 [Homo sapiens]	10	47	biological process; development	1.069	0.909	0.962
5254572	hypothetical protein, partial [Homo sapiens]	3	5	metabolic process	0.806	0.909	0.769
7680401	PREDICTED: translocon-associated protein subunit delta isoform 1	5	48	ization and biogenesis; transport	1.302	0.909	0.958
7679273	PREDICTED: D-beta-hydroxybutyrate dehydrogenase, mitochondri	6	28	metabolic process	1.014	0.909	0.881
1890532	unnamed protein product [Homo sapiens]	3	21	; regulation of biological process	0.934	0.909	0.903
1196075	hypothetical protein MGC11257, isoform CRA_a, partial [Homo sap	2	13		0.965	0.909	0.927
2210449	unnamed protein product, partial [Homo sapiens]	4	7	metabolic process	1.053	0.909	0.814
1232077	huMCM2 [Homo sapiens]	17	65	biogenesis; response to stimulus	1.049	0.908	0.944
1195693	hCG1640785, isoform CRA_a [Homo sapiens]	3	25		0.861	0.908	0.889
9368544	c3orf1 hypothetical protein, M5-14 alternatively spliced product, si	2	8	metabolic process	0.991	0.907	0.884
1195886	dendritic cell protein, isoform CRA_b [Homo sapiens]	3	5	; regulation of biological process	0.972	0.907	0.972
1401035	tropomyosin 4-anaplastic lymphoma kinase fusion protein minor is	5	111		1.117	0.907	0.947
5788215	PREDICTED: serine/threonine-protein phosphatase 6 regulatory su	4	8	cell organization and biogenesis	0.936	0.907	0.817
1195870	hCG1983504, isoform CRA_a [Homo sapiens]	14	260	s; cellular component movement	1.036	0.906	0.897
1195903	KIAA0133, isoform CRA_a [Homo sapiens]	4	9		0.970	0.906	0.845
7679111	PREDICTED: glycosaminoglycan xylosylkinase isoform X2 [Homo	2	4	metabolic process	0.987	0.906	0.860

1195957	oxysterol binding protein-like 2, isoform CRA_b, partial [Homo sap	2	6	transport	0.846	0.906	0.869
7491974	Chain A, Crystal Structure Of Human Camp-dependent Protein Kin	5	14		1.113	0.906	0.955
1890532	unnamed protein product [Homo sapiens]	2	18	cell organization and biogenesis	0.999	0.905	0.871
1280488	N-ethylmaleimide-sensitive factor attachment protein, gamma [Hor	2	5	; regulation of biological process	0.746	0.905	0.928
1196203	malate dehydrogenase 1, NAD (soluble), isoform CRA_d [Homo sa	10	49	gical process; metabolic process	1.095	0.905	0.923
7413655	LDLR chaperone MESD precursor [Homo sapiens]	3	7	al process; response to stimulus	1.190	0.905	1.095
1442454	RPL14 protein [Homo sapiens]	4	46	ization and biogenesis; transport	0.932	0.904	0.881
5788160	PREDICTED: regulator of microtubule dynamics protein 1 isoform	2	2		1.203	0.904	0.857
1943755	unnamed protein product [Homo sapiens]	8	43	; regulation of biological process	0.982	0.904	0.904
1168126	epimerase family protein SDR39U1 isoform 1 [Homo sapiens]	3	8	metabolic process	0.789	0.904	0.880
1943886	unnamed protein product [Homo sapiens]	13	38	cell organization and biogenesis	0.971	0.904	0.943
1943829	unnamed protein product, partial [Homo sapiens]	6	24	metabolic process	0.951	0.904	0.899
1195740	hCG1734196, isoform CRA_a [Homo sapiens]	2	8	cell organization and biogenesis	0.839	0.904	0.921
1943888	unnamed protein product [Homo sapiens]	6	16	; regulation of biological process	0.903	0.903	0.877
8957417	succinate dehydrogenase complex subunit A, partial [Homo sapier	14	135	metabolic process; transport	1.040	0.903	0.926
1582546	unnamed protein product [Homo sapiens]	7	36	nse response; cell differentiation	1.154	0.903	0.924
5788117	PREDICTED: p21-activated protein kinase-interacting protein 1 iso	2	4	; regulation of biological process	1.060	0.903	0.856
3718727	transmembrane and coiled-coil domain-containing protein 1 isofor	2	10		0.797	0.903	0.905
3801396	TKT protein, partial [Homo sapiens]	19	228	; regulation of biological process	1.089	0.903	0.870
7680215	PREDICTED: trafficking protein particle complex subunit 10 isofor	2	4	cell organization and biogenesis	1.121	0.903	0.917
550343	BAP31 [Homo sapiens]	8	37	transport	0.989	0.903	0.922
4147211	unknown, partial [Homo sapiens]	9	73	ent movement; defense response	1.250	0.903	0.898
1195682	utrophin (homologous to dystrophin), isoform CRA_a [Homo sapie	18	41	regulation of biological process	0.979	0.903	0.888
1871199	pM5 (3' partial), partial [Homo sapiens]	20	63		1.054	0.902	0.868
1943790	unnamed protein product [Homo sapiens]	2	4	; regulation of biological process	0.752	0.902	0.915
3315053	GTP:ATP phosphotransferase [Homo sapiens]	3	7	metabolic process; coagulation	0.949	0.902	0.867
2376405	Chain B, Crystal Structure Of The Hscarg R37a Mutant	6	9	transport	1.019	0.901	0.843
1195767	seryl-tRNA synthetase, isoform CRA_c [Homo sapiens]	4	7	metabolic process	0.779	0.901	0.718
3883281	Chain C, Crystal Structure Of Human Otub1UBCH5B~UBUB	7	41		1.175	0.901	0.964
1195988	actin-like 6A, isoform CRA_c [Homo sapiens]	4	21	; regulation of biological process	1.048	0.901	0.825
1043801	unnamed protein product [Homo sapiens]	3	8	development	0.927	0.901	0.963
1880362	Chain A, Set7/9-er-sinefungin Complex	2	9		0.871	0.901	0.766
5788285	PREDICTED: serine/threonine-protein phosphatase 4 catalytic sub	3	15	; regulation of biological process	1.298	0.901	0.976
1196012	heat shock 70kDa protein 2, isoform CRA_a [Homo sapiens]	13	400	e to stimulus; metabolic process	1.270	0.901	0.934
5059298	cytochrome b-c1 complex subunit 2, mitochondrial precursor [Hor	11	71	metabolic process; transport	1.007	0.901	0.962
1584294	Chain A, Crystal Structure Of Human Cbr1 In Complex With Bigf2.	8	48	metabolic process	1.049	0.900	0.852
1195878	hydroxymethylbilane synthase, isoform CRA_c [Homo sapiens]	3	7	bolic process; cell differentiation	0.870	0.900	0.921
7243115	KIAA1367 protein, partial [Homo sapiens]	2	6	metabolic process; transport	1.173	0.900	1.169
1943200	Chain A, Crystal Structure Of Nore1a In Complex With Ras	3	21	; response to stimulus; transport	1.372	0.900	1.039
1195703	aldehyde dehydrogenase 18 family, member A1, isoform CRA_b [H	25	186	metabolic process	1.076	0.900	0.885

7679141	PREDICTED: dystrobrevin beta isoform X12 [Homo sapiens]	4	7		0.885	0.900	1.008
4318224	heat shock protein HSP 90-beta isoform c [Homo sapiens]	30	968	cell organization and biogenesis	1.241	0.900	0.963
1943873	unnamed protein product [Homo sapiens]	2	7	nd biogenesis; defense response	1.108	0.899	0.959
1195810	keratin 25D [Homo sapiens]	4	58		0.895	0.899	1.462
1312901	gamma-glutamylcyclotransferase isoform 1 [Homo sapiens]	8	68	d biogenesis; metabolic process	1.056	0.899	0.907
1065071	ras-related protein Rab-12 [Homo sapiens]	4	48	cell organization and biogenesis	1.110	0.899	0.839
5254570	hypothetical protein [Homo sapiens]	3	9		1.143	0.899	1.006
5505455	Chain B, Crystal Structure Of Nucleotide-free Rab8:rabin8	9	70		1.112	0.898	0.945
4004137	unnamed protein product, partial [Homo sapiens]	3	7		0.992	0.898	0.833
1196183	ATPase, Ca++ transporting, cardiac muscle, slow twitch 2, isoform	22	120	; coagulation; metabolic process	1.136	0.898	0.926
1943892	unnamed protein product [Homo sapiens]	14	44	; regulation of biological process	1.013	0.898	0.920
2968033	ESRP1/RAF1 fusion protein [Homo sapiens]	9	39	metabolic process	0.959	0.898	0.956
2700634	Chain A, Cdc42(T35a)	5	20	al process; response to stimulus	1.070	0.898	1.064
7679797	PREDICTED: mitochondrial basic amino acids transporter isoform	6	13	transport	0.834	0.898	0.951
8819197	Chain D, Crystal Structure Of Human Deoxycytidine Kinase In Com	2	4	metabolic process	0.812	0.897	0.959
1582598	unnamed protein product [Homo sapiens]	17	465	cell organization and biogenesis	1.028	0.897	0.894
1890691	unnamed protein product [Homo sapiens]	6	14	; regulation of biological process	0.969	0.897	0.910
1196030	cyclin G associated kinase, isoform CRA_b [Homo sapiens]	4	8	; regulation of biological process	1.166	0.897	0.863
1834481	Chain C, Crystal Structure Of Human Peroxisomal Delta3,5, Delta2,	10	116	metabolic process	1.082	0.897	0.844
2099441	arrestin beta 1 transcript variant 2 [Homo sapiens]	7	20	cell organization and biogenesis	1.177	0.897	0.912
7679486	PREDICTED: sorcin isoform X1 [Homo sapiens]	5	25	al process; response to stimulus	1.019	0.897	0.912
3997863	unnamed protein product, partial [Homo sapiens]	14	82		0.927	0.897	0.929
1943770	unnamed protein product [Homo sapiens]	9	45	; regulation of biological process	1.145	0.896	0.976
6208736	hepatoma-derived growth factor-related protein 2 isoform 1 variant	3	20	; regulation of biological process	0.909	0.896	0.955
1943872	unnamed protein product [Homo sapiens]	6	61	cell organization and biogenesis	1.012	0.896	0.872
1937878	unnamed protein product [Homo sapiens]	17	118	; regulation of biological process	1.067	0.896	0.901
1195881	amyloid beta (A4) precursor-like protein 2, isoform CRA_d [Homo s	3	12	cell organization and biogenesis	0.922	0.896	0.850
1591635	Chain A, Solution Structures Of The Hth Domain Of Human Edf-1 P	2	4		0.716	0.895	0.894
1992412	DNA repair protein RAD50 [Homo sapiens]	8	28	ic process; response to stimulus	1.074	0.895	0.934
1403592	unnamed protein product [Homo sapiens]	2	2		0.990	0.895	0.861
1195746	vacuolar protein sorting 26 homolog A (yeast), isoform CRA_b [Ho	8	46	transport	1.052	0.895	0.910
7022627	unnamed protein product [Homo sapiens]	6	16	cell organization and biogenesis	0.925	0.895	0.946
5303972	PREDICTED: neuroblast differentiation-associated protein AHNAK	11	51	cell organization and biogenesis	1.089	0.895	0.911
7679518	PREDICTED: chromodomain-helicase-DNA-binding protein 7 isofo	3	6	nd biogenesis; cell differentiation	0.899	0.895	0.899
3740818	ras-related protein Rab-18 isoform 3 [Homo sapiens]	4	12	cell organization and biogenesis	1.053	0.895	0.996
1196215	acid phosphatase 1, soluble, isoform CRA_d [Homo sapiens]	5	29	metabolic process	1.024	0.895	1.014
1196071	transcription elongation factor A (SII), 1, isoform CRA_a [Homo sa	7	40	gical process; cell differentiation	0.918	0.894	0.800
2827444	p50 dynamitin [Homo sapiens]	7	44	tabolic process; cell proliferation	0.973	0.894	0.882
2494308	3'-5' RNA exonuclease [Homo sapiens]	5	15	; response to stimulus; transport	1.062	0.894	1.000
1196030	hCG1815489 [Homo sapiens]	5	53	al process; response to stimulus	1.144	0.894	0.911

1196152	capping protein (actin filament) muscle Z-line, beta, isoform CRA_	11	79	; regulation of biological process	1.069	0.894	0.809
1195809	hCG31406, isoform CRA_a [Homo sapiens]	2	3		1.068	0.894	0.907
300302	adenylosuccinate lyase [Homo sapiens]	5	10	cell organization and biogenesis	1.017	0.894	0.918
1937858	unnamed protein product [Homo sapiens]	6	10	; metabolic process; cell division	1.101	0.893	0.828
1195985	eukaryotic translation initiation factor 4A, isoform 2, isoform CRA_	13	96	al process; response to stimulus	1.064	0.893	0.922
1196122	poly(A) binding protein, cytoplasmic 1, isoform CRA_c [Homo sapi	18	119	; regulation of biological process	1.058	0.893	0.905
4004081	unnamed protein product, partial [Homo sapiens]	3	27		0.839	0.893	0.841
1141551	tropomyosin alpha-3 chain isoform Tpm3.2cy [Homo sapiens]	12	156	cellular component movement	1.112	0.892	0.930
1228045	nuclear pore protein, partial [Homo sapiens]	9	36	al process; response to stimulus	0.997	0.892	0.923
1195884	peroxisomal biogenesis factor 16, isoform CRA_b [Homo sapiens]	4	12	ization and biogenesis; transport	0.791	0.892	0.849
2781202	Chain A, Three-Dimensional Structure Of Human Electron Transfer	8	19		1.132	0.892	0.983
9096180	clathrin light chain A isoform g [Homo sapiens]	4	35	cell organization and biogenesis	1.165	0.891	0.951
1582600	unnamed protein product [Homo sapiens]	3	4	ic process; response to stimulus	0.875	0.891	0.926
1943749	unnamed protein product [Homo sapiens]	21	118	; regulation of biological process	1.058	0.891	0.939
5094942	hypothetical protein, partial [Homo sapiens]	2	3	metabolic process	0.867	0.891	0.853
1309715	Similar to NADH dehydrogenase (ubiquinone) 1 alpha subcomplex	8	38	metabolic process; transport	1.019	0.891	0.906
1424995	DUS3L protein, partial [Homo sapiens]	2	5	metabolic process	0.840	0.891	0.950
1195904	tubulin-specific chaperone e, isoform CRA_c [Homo sapiens]	3	7	ic process; response to stimulus	0.760	0.891	0.983
2790021	unnamed protein product [Homo sapiens]	2	7		0.922	0.890	0.871
1582552	unnamed protein product [Homo sapiens]	2	3	al process; response to stimulus	1.015	0.890	0.813
3232765	NADH dehydrogenase [ubiquinone] 1 subunit C2, isoform 2 isofor	3	9	metabolic process; transport	1.059	0.890	0.937
1195680	t-complex 1, isoform CRA_a [Homo sapiens]	25	238	; regulation of biological process	1.081	0.890	0.899
1937837	unnamed protein product [Homo sapiens]	5	10		0.864	0.890	0.899
6879981	AP-2 complex subunit mu isoform b [Homo sapiens]	11	41	cell organization and biogenesis	1.077	0.890	0.886
1195782	PTK9 protein tyrosine kinase 9, isoform CRA_a [Homo sapiens]	6	14	; regulation of biological process	1.212	0.889	0.983
2210455	unnamed protein product [Homo sapiens]	2	3	gical process; metabolic process	0.907	0.889	0.819
4884138	hypothetical protein, partial [Homo sapiens]	7	26	al process; response to stimulus	1.023	0.889	0.845
2962784	Chain A, Crystal Structure Of Ccm3, A Cerebral Cavernous Malforn	3	11		0.927	0.889	0.900
7238007	unnamed protein product [Homo sapiens]	4	52	cell organization and biogenesis	1.038	0.889	0.852
1890533	unnamed protein product [Homo sapiens]	4	33	cell organization and biogenesis	1.219	0.889	0.929
1195964	KIAA1219 protein, isoform CRA_d [Homo sapiens]	4	12	cell organization and biogenesis	0.858	0.889	0.831
6399281	unknown [Homo sapiens]	2	6	; regulation of biological process	1.115	0.888	0.874
1943756	unnamed protein product [Homo sapiens]	3	76	n of biological process; transport	1.117	0.888	0.838
1196037	hCG18199, isoform CRA_a [Homo sapiens]	3	10	; regulation of biological process	0.814	0.888	0.889
7679964	PREDICTED: cytohesin-1 isoform X3 [Homo sapiens]	3	10	; regulation of biological process	0.830	0.888	0.873
7679367	PREDICTED: secretory carrier-associated membrane protein 1 isof	4	11	transport	1.170	0.888	0.785
1175585	Phosphoinositide-3-kinase, regulatory subunit 4 [Homo sapiens]	2	6	nd biogenesis; defense response	1.167	0.888	0.853
1195975	transcription factor B2, mitochondrial, isoform CRA_b [Homo sapi	2	3	; regulation of biological process	0.769	0.888	0.870
1195725	branched chain ketoacid dehydrogenase kinase, isoform CRA_a [H	4	8	metabolic process	1.095	0.888	0.959
7679361	PREDICTED: geranylgeranyl transferase type-1 subunit beta isofor	2	5	ic process; response to stimulus	0.949	0.888	0.925

4109919	pyrroline-5-carboxylate reductase 2 isoform 2 [Homo sapiens]	4	43	ic process; response to stimulus	1.024	0.887	0.831
1105915	Chain B, Structure Of The Binary Complex Of The E67l Mutant Of H	6	17	metabolic process	1.042	0.887	0.824
6624131	similar to T13C5.6 gene product [Caenorhabditis elegans]; similar	3	11	zation and biogenesis; cell death	1.041	0.887	0.913
3925136	interleukin enhancer-binding factor 2 isoform 2 [Homo sapiens]	8	44	; regulation of biological process	0.951	0.887	0.921
1196034	thymidine kinase 2, mitochondrial, isoform CRA_a [Homo sapiens]	3	6	metabolic process	1.205	0.886	0.906
7679175	PREDICTED: 3-hydroxyisobutyryl-CoA hydrolase, mitochondrial is	2	4	metabolic process	1.099	0.886	0.900
1943876	unnamed protein product [Homo sapiens]	2	4	entiation; cell death; cell division	0.997	0.886	0.938
7680239	PREDICTED: tubulin--tyrosine ligase-like protein 12 isoform X1 [Ho	8	26	metabolic process	1.088	0.886	1.020
1471780	glutathione peroxidase [Homo sapiens]	2	4	ar homeostasis; cell proliferation	0.828	0.886	0.774
5441948	serine/threonine protein kinase; similar to B-raf proto-oncogene; n	5	12	cell organization and biogenesis	1.105	0.886	0.902
1340152	unnamed protein product [Homo sapiens]	5	12	ent movement; defense response	1.084	0.886	0.902
34201	unnamed protein product [Homo sapiens]	4	25	ization and biogenesis; transport	0.958	0.886	0.816
3315062	DCHT2 [Homo sapiens]	3	8	; regulation of biological process	0.791	0.885	0.890
1196059	transducin (beta)-like 3, isoform CRA_e [Homo sapiens]	8	27	al process; response to stimulus	1.068	0.885	0.917
2401042	Chain A, Solution Structure Of 1-112 Fragment Of Human Program	4	35		0.936	0.885	0.850
5898115	ribosyldihydronicotinamide dehydrogenase [quinone] isoform 2 [H	2	6	e to stimulus; metabolic process	0.801	0.885	0.883
3478321	SLC25A3 protein, partial [Homo sapiens]	9	77	metabolic process; transport	0.953	0.885	0.781
3964460	DVL1 protein, partial [Homo sapiens]	2	4	esponse to stimulus; cell growth	0.769	0.884	0.877
5303678	PREDICTED: tetratricopeptide repeat protein 27 isoform X2 [Homo	4	7		0.876	0.884	0.857
5305376	Chain B, Crystal Structure Of The Grp1 Ph Domain In Complex Wit	2	7		0.800	0.884	0.904
247307	cytochrome P450 reductase, partial [Homo sapiens]	13	62	e to stimulus; metabolic process	1.166	0.884	0.954
1683411	nicotinamide adenine dinucleotide phosphate (NADP[H])-binding p	3	13		1.061	0.884	0.815
2014769	guanine nucleotide binding protein alpha 13 [Homo sapiens]	5	12	; coagulation; cell differentiation	1.009	0.883	0.888
1265608	fibroblast growth factor 2-interacting factor, partial (plasmid) [Hom	5	16	; regulation of biological process	0.898	0.883	0.890
1196241	WD repeat domain 46, isoform CRA_a [Homo sapiens]	2	5	metabolic process	0.796	0.882	0.954
6087856	C-terminal binding protein 1 isoform D [Homo sapiens]	8	32	bolic process; cell differentiation	1.037	0.882	0.894
2098705	Chain A, Crystal Structure Of The Mammalian Copii-coat Protein S	19	147	transport	0.980	0.882	0.858
7679125	PREDICTED: histone-lysine N-methyltransferase SMYD3 isoform X	2	6	ic process; response to stimulus	0.889	0.882	0.909
7679201	PREDICTED: cullin-3 isoform X2 [Homo sapiens]	8	16	on; cell death; defense response	1.059	0.882	0.949
1196058	potassium channel tetramerisation domain containing 5, isoform C	2	4	cell organization and biogenesis	0.843	0.882	0.841
1229203	Chain D, C6aC111A CUZN SUPEROXIDE DISMUTASE	3	15	metabolic process	0.767	0.882	0.875
7679660	PREDICTED: methylthioribulose-1-phosphate dehydratase isoform	2	3	; regulation of biological process	0.904	0.882	0.738
7680161	PREDICTED: exportin-2 isoform X1 [Homo sapiens]	23	143	port; cell death; cell proliferation	1.067	0.881	0.921
4004274	unnamed protein product, partial [Homo sapiens]	3	19		1.076	0.881	0.962
7679149	PREDICTED: ras-related protein Rab-1A isoform X1 [Homo sapiens	12	106	se to stimulus; defense response	1.225	0.881	0.896
1196274	eukaryotic translation initiation factor 2B, subunit 3 gamma, 58kDa	3	8	; regulation of biological process	1.038	0.881	0.845
1162838	PAPSS1 protein, partial [Homo sapiens]	5	29	metabolic process	1.012	0.881	0.858
493066	glycyl-tRNA synthetase [Homo sapiens]	16	58	metabolic process	1.029	0.881	0.874
1043525	unnamed protein product [Homo sapiens]	3	12	metabolic process	0.856	0.880	0.915
854189	tropomyosin isoform [Homo sapiens]	13	83	biogenesis; response to stimulus	1.231	0.880	0.928

2443015	26S protease regulatory subunit 6B isoform 2 [Homo sapiens]	6	30	ent movement; defense response	0.999	0.880	0.929
2210455	unnamed protein product [Homo sapiens]	7	47	; regulation of biological process	1.137	0.880	0.857
7679737	PREDICTED: coronin-1C isoform X1 [Homo sapiens]	5	19	cell organization and biogenesis	0.932	0.880	0.913
1943812	unnamed protein product, partial [Homo sapiens]	3	6	gical process; cell differentiation	0.952	0.880	0.871
1195747	SAR1 gene homolog A (S. cerevisiae), isoform CRA_a [Homo sapie	5	25	al process; response to stimulus	1.028	0.880	0.902
7679908	PREDICTED: V-type proton ATPase subunit d 1 isoform X1 [Homo	5	19	d biogenesis; metabolic process	1.008	0.879	0.807
5573577	adenosine 3'-phospho 5'-phosphosulfate transporter 1 isoform c [I	7	29	; response to stimulus; transport	0.859	0.879	0.762
1195714	polymerase (DNA-directed), delta interacting protein 2 [Homo sapi	8	26	cell organization and biogenesis	1.104	0.879	0.906
1195812	ATPase, H+ transporting, lysosomal V0 subunit a1, isoform CRA_b	11	46	cell organization and biogenesis	1.043	0.879	0.832
4097850	unnamed protein product, partial [Homo sapiens]	2	12		1.060	0.879	0.977
1706404	insulinoma-glucagonoma protein 20 splice variant 4 [Homo sapien	2	3	al process; response to stimulus	0.816	0.879	0.800
1195712	hCG37070, partial [Homo sapiens]	3	25	metabolic process	1.013	0.879	1.046
7023874	unnamed protein product, partial [Homo sapiens]	4	6	metabolic process; transport	0.843	0.878	0.869
7679226	PREDICTED: 2',5'-phosphodiesterase 12 isoform X1 [Homo sapien	5	27	metabolic process	0.909	0.878	0.857
5304307	PREDICTED: general transcription factor IIH subunit 2-like protein	2	4	; regulation of biological process	1.065	0.878	0.946
5456874	protein RTF2 homolog isoform d [Homo sapiens]	2	4		0.897	0.878	0.839
6649223	selenoprotein Zf1 [Homo sapiens]	2	4	gical process; metabolic process	0.824	0.878	0.836
7680033	PREDICTED: ras-related protein Rab-3A isoform X1 [Homo sapiens	4	52	to stimulus; cell communication	1.200	0.878	0.852
1582563	unnamed protein product [Homo sapiens]	2	17	; regulation of biological process	0.977	0.878	1.012
7679560	PREDICTED: structural maintenance of chromosomes protein 2 iso	8	24	f biological process; cell division	1.017	0.877	0.977
1582570	unnamed protein product, partial [Homo sapiens]	12	58	logical process; cell proliferation	0.978	0.877	0.910
7679136	PREDICTED: leucine-rich PPR motif-containing protein, mitochond	31	119	component movement; transport	1.059	0.876	0.895
1196108	hCG1986053, isoform CRA_b [Homo sapiens]	4	8		0.920	0.876	0.878
7679779	PREDICTED: replication factor C subunit 3 isoform X5 [Homo sapi	3	12	o stimulus; cellular homeostasis	0.900	0.876	0.843
1943876	unnamed protein product [Homo sapiens]	11	98	transport	0.983	0.876	0.911
1943735	unnamed protein product [Homo sapiens]	5	9	gical process; cell differentiation	1.141	0.876	1.012
1890535	unnamed protein product [Homo sapiens]	4	11	metabolic process	1.116	0.876	0.933
1147946	Chain D, Crystal Structure Of Human Apo-Eif4aiii	15	105		1.047	0.875	0.927
6208768	SWI/SNF-related matrix-associated actin-dependent regulator of ch	7	19	cell organization and biogenesis	0.904	0.875	0.903
1196108	arrestin, beta 2, isoform CRA_a [Homo sapiens]	3	7	n; cellular component movement	1.029	0.875	1.004
5441859	exosome complex component RRP4 isoform 2 [Homo sapiens]	4	14	; regulation of biological process	0.965	0.875	0.834
1662353	Chain C, Human Neuronal Rab6b In Three Intermediate Forms	5	51	; response to stimulus; transport	1.066	0.875	0.942
1195772	seryl-tRNA synthetase 2, isoform CRA_d [Homo sapiens]	6	12	d biogenesis; metabolic process	0.864	0.875	1.028
1196284	family with sequence similarity 105, member A, isoform CRA_b [Ho	3	7		0.996	0.874	0.808
1943829	unnamed protein product [Homo sapiens]	16	77	metabolic process	1.020	0.874	0.827
1195811	ATP citrate lyase, isoform CRA_a [Homo sapiens]	38	372	; regulation of biological process	1.083	0.874	0.868
1582556	unnamed protein product [Homo sapiens]	4	12	o stimulus; cellular homeostasis	0.861	0.874	0.832
1808831	Nitrilase family, member 2 [Homo sapiens]	8	46	metabolic process	0.961	0.874	0.802
1327928	CHMP7 protein, partial [Homo sapiens]	2	6	ization and biogenesis; transport	1.098	0.874	0.833
5605750	tumor protein D52 isoform 7 [Homo sapiens]	3	6	ess; cell differentiation; transport	0.877	0.874	0.902

1196183	actin related protein 2/3 complex, subunit 3, 21kDa, isoform CRA_I	3	7	nd biogenesis; defense response	0.857	0.874	0.853
7679688	PREDICTED: proteasomal ATPase-associated factor 1 isoform X2	6	12		0.919	0.873	0.848
1890534	unnamed protein product [Homo sapiens]	9	46	ic process; response to stimulus	1.052	0.873	0.950
1196132	phosphoglucomutase 2 [Homo sapiens]	14	48	metabolic process	1.044	0.873	0.879
7353575	Chain D, Gdp-Bound Rab2a Gtpase	11	56	al process; response to stimulus	1.074	0.872	0.918
1617612	Chain A, Crystal Structure Of The Human Laminin Receptor Precu	8	116	metabolic process	0.952	0.872	0.774
1196118	cytochrome b5 reductase 1, isoform CRA_a [Homo sapiens]	4	19	metabolic process	1.207	0.872	0.971
4991375	tropomyosin alpha-3 chain isoform 8 [Homo sapiens]	13	168	cellular component movement	1.115	0.872	0.918
1196152	karyopherin (importin) beta 1, isoform CRA_b [Homo sapiens]	17	132	al process; response to stimulus	1.012	0.872	0.837
1196140	NADH dehydrogenase (ubiquinone) 1 alpha subcomplex, 4, 9kDa, i	4	16	metabolic process; transport	1.279	0.872	0.992
1937869	unnamed protein product [Homo sapiens]	3	5	al process; response to stimulus	1.132	0.872	0.853
1173565	golgin-245 [Homo sapiens]	5	12	; regulation of biological process	0.979	0.872	0.813
1196069	phosphofructokinase, platelet, isoform CRA_b [Homo sapiens]	12	54	metabolic process	0.994	0.871	0.858
2895771	eukaryotic translation initiation factor 4 gamma 2 isoform 2 [Homo	6	11	cell death; response to stimulus	1.157	0.871	0.985
7679020	PREDICTED: target of EGR1 protein 1 isoform X3 [Homo sapiens]	3	5	metabolic process	1.015	0.871	0.871
1265279	Chromosome 22 open reading frame 28 [Homo sapiens]	9	34	metabolic process	0.949	0.871	0.911
9305889	dnaJ homolog subfamily A member 1 isoform 2 [Homo sapiens]	2	3		0.961	0.871	0.813
1584308	Chain A, Solution Structure Of Cu(i) Human Sco2	4	9	metabolic process	0.980	0.870	0.841
7679084	PREDICTED: ras GTPase-activating-like protein IQGAP3 isoform X	2	6	d biogenesis; metabolic process	0.760	0.870	0.905
1195953	phosphoglucomutase 2-like 1 [Homo sapiens]	4	9	metabolic process	1.117	0.870	0.969
2910451	N-acetylserotonin O-methyltransferase-like protein isoform 3 [Hom	10	38	metabolic process	0.957	0.870	0.917
1890544	unnamed protein product [Homo sapiens]	2	5	cell organization and biogenesis	0.974	0.870	0.885
2081037	C12orf10 protein [Homo sapiens]	5	25	response to stimulus	1.006	0.870	0.849
1195997	Sec61 alpha 1 subunit (S. cerevisiae), isoform CRA_b [Homo sapie	7	99	e to stimulus; metabolic process	0.921	0.870	0.880
1196310	NSFL1 (p97) cofactor (p47), isoform CRA_d [Homo sapiens]	8	47	; regulation of biological process	0.967	0.869	0.847
8579490	ATP synthase subunit f, mitochondrial isoform 2d [Homo sapiens]	2	24	transport; metabolic process	1.119	0.869	0.881
1195814	transforming growth factor beta regulator 4, isoform CRA_b [Hom	7	17	; regulation of biological process	1.020	0.869	0.965
5787991	PREDICTED: sodium/potassium-transporting ATPase subunit alph	30	294	to stimulus; cell communication	1.034	0.868	0.831
5304119	PREDICTED: protein HID1 isoform X1 [Homo sapiens]	8	39	transport; response to stimulus	0.949	0.868	1.006
2276119	unnamed protein product [Homo sapiens]	4	9	n of biological process; transport	0.799	0.868	0.863
5707008	H/ACA ribonucleoprotein complex subunit 4 isoform 3 [Homo sapi	7	24	; regulation of biological process	1.099	0.868	0.851
7679228	PREDICTED: serine/threonine-protein phosphatase 6 regulatory ar	4	6	cell organization and biogenesis	0.997	0.867	0.879
3753323	Chain D, Crystal Structure Of Human Pacsin 2 F-Bar	5	22		0.990	0.867	0.911
1582545	unnamed protein product [Homo sapiens]	20	208	metabolic process; transport	0.973	0.867	0.850
1196015	aldehyde dehydrogenase 6 family, member A1, isoform CRA_a [Ho	10	54	bolic process; cell differentiation	0.999	0.867	0.936
860963	DNA ligase III [Homo sapiens]	6	24	f biological process; cell division	0.960	0.867	0.902
1206118	ASC-1 complex subunit P200, partial [Homo sapiens]	14	31	logical process; cell proliferation	1.029	0.867	0.876
7679473	PREDICTED: transducin beta-like protein 2 isoform X2 [Homo sapi	10	47	to stimulus; cell communication	0.983	0.867	0.853
5788240	PREDICTED: disco-interacting protein 2 homolog B isoform X7 [Ho	9	22	metabolic process	0.939	0.867	0.811
1195951	carnitine palmitoyltransferase 1A (liver), isoform CRA_a [Homo sa	25	146	cell organization and biogenesis	1.048	0.866	0.795

2276030	unnamed protein product [Homo sapiens]	9	23	cell organization and biogenesis	0.950	0.866	0.915
1271163	CGI-201 protein, short form [Homo sapiens]	8	21	; regulation of biological process	1.067	0.866	0.848
7679838	PREDICTED: vacuolar protein sorting-associated protein 33B isofo	4	9	; regulation of biological process	1.155	0.866	0.844
5517016	Chain D, Structure Of Recombinant Human Cytochrome C	5	24		0.964	0.866	0.825
7679186	PREDICTED: protein phosphatase 1 regulatory subunit 7 isoform X	4	11	regulation of biological process	1.025	0.865	0.813
1106696	EH domain-containing protein FKSG7 [Homo sapiens]	8	21	biogenesis; response to stimulus	1.031	0.865	0.849
4088960	Chain H, Appbp1-Uba3-Nedd8, An E1-Ubiquitin-Like Protein Comp	5	22	metabolic process	1.025	0.865	0.905
7679236	PREDICTED: solute carrier family 25 member 38 isoform X4 [Homo	2	3	ess; transport; cell differentiation	0.802	0.865	0.928
2837240	kinase/transmembrane domain fusion protein, partial [Homo sapie	2	2	metabolic process	0.824	0.865	0.881
5788042	PREDICTED: integrin alpha-V isoform X1 [Homo sapiens]	6	12	on; transport; metabolic process	0.877	0.864	0.829
4004133	unnamed protein product, partial [Homo sapiens]	4	17		1.082	0.864	0.915
1196170	keratin 2A (epidermal ichthyosis bullosa of Siemens) [Homo sapie	32	249	n; cellular component movement	0.918	0.864	1.399
1196170	keratin 6E, isoform CRA_b [Homo sapiens]	25	205	cell organization and biogenesis	1.010	0.864	1.082
1890533	unnamed protein product [Homo sapiens]	8	49	roliferation; cellular homeostasis	1.021	0.864	0.885
1943798	unnamed protein product [Homo sapiens]	2	4	ic process; response to stimulus	0.987	0.864	0.880
1196196	cytochrome c oxidase subunit Va, isoform CRA_b [Homo sapiens]	3	47	metabolic process; transport	0.928	0.864	0.819
1196102	progesterone receptor membrane component 1, isoform CRA_b [H	7	61	cellular component movement	1.006	0.864	0.867
1943879	unnamed protein product [Homo sapiens]	25	203	cell organization and biogenesis	1.010	0.863	1.081
4030720	Chain E, Espg-Rab1-Arf6 Complex	11	101		1.233	0.863	0.881
1943839	unnamed protein product [Homo sapiens]	5	23	; regulation of biological process	0.880	0.863	0.864
5303751	PREDICTED: selenocysteine-specific elongation factor isoform X4	7	21	; regulation of biological process	0.913	0.862	0.923
1943824	unnamed protein product [Homo sapiens]	11	62	metabolic process	0.960	0.862	0.897
1043409	unnamed protein product, partial [Homo sapiens]	4	7	d biogenesis; metabolic process	0.962	0.862	0.825
1195973	hCG2023776 [Homo sapiens]	6	47	metabolic process	1.134	0.862	1.120
1195773	small nuclear ribonucleoprotein polypeptide A, isoform CRA_a [Ho	6	27	metabolic process	0.844	0.862	0.923
1890551	unnamed protein product [Homo sapiens]	4	12	oliferation; coagulation; transport	0.950	0.862	0.784
1195810	keratin 10 (epidermolytic hyperkeratosis; keratosis palmaris et pla	28	294	erentiation; response to stimulus	0.895	0.861	1.707
1195804	F-box protein 7, isoform CRA_b, partial [Homo sapiens]	2	4	; regulation of biological process	1.039	0.861	0.908
3875280	alanyl-tRNA editing protein Aarsd1 [Homo sapiens]	5	19	; regulation of biological process	1.011	0.861	0.908
1461343	NAD kinase 2, mitochondrial isoform 1 [Homo sapiens]	6	23	al process; response to stimulus	1.150	0.861	0.919
1196057	DnaJ (Hsp40) homolog, subfamily A, member 3, isoform CRA_a, pa	4	14	nd biogenesis; cell differentiation	0.980	0.861	0.891
1195706	membrane interacting protein of RGS16, isoform CRA_c [Homo sa	2	4	metabolic process	0.900	0.861	0.892
4842540	Chain D, Crystal Structure Of Isobutyryl-Coa Dehydrogenase Comp	6	11	metabolic process	1.155	0.861	0.899
1203465	ribeye [Homo sapiens]	9	35	bolic process; cell differentiation	1.097	0.860	0.886
1260316	Chain H, Crystal Structure Of Human Ctbp2 Dehydrogenase Comp	9	35	metabolic process	1.076	0.860	0.882
1449561	WDR77 protein [Homo sapiens]	2	11	bolic process; cell differentiation	0.961	0.859	0.799
1943808	unnamed protein product [Homo sapiens]	5	13	; response to stimulus; transport	0.809	0.859	0.913
1890550	unnamed protein product [Homo sapiens]	2	10	d biogenesis; metabolic process	0.827	0.859	0.889
4503351	DNA (cytosine-5)-methyltransferase 1 isoform b [Homo sapiens]	10	33	biogenesis; response to stimulus	0.954	0.859	0.867
181402	epidermal cytokeratin 2 [Homo sapiens]	32	245	n; cellular component movement	0.911	0.859	1.384

1860550	PMPCA protein, partial [Homo sapiens]	8	14	metabolic process; transport	1.041	0.859	0.884
4028684	wasp-interacting SH3 protein isoform B [Homo sapiens]	3	5	se to stimulus; defense response	0.848	0.859	0.789
1195903	ATP-binding cassette, sub-family B (MDR/TAP), member 10 [Homo	10	24	metabolic process; transport	1.007	0.859	0.832
7679376	PREDICTED: probable histidine--tRNA ligase, mitochondrial isofor	5	27	cell organization and biogenesis	0.936	0.859	0.879
1196272	cytidylate kinase, isoform CRA_b [Homo sapiens]	2	5	metabolic process	0.879	0.859	0.906
1009337	RanBP2 (Ran-binding protein 2) [Homo sapiens]	16	33	; regulation of biological process	0.968	0.858	0.869
3778244	LP6054 [Homo sapiens]	2	3		1.139	0.858	0.827
1196288	heat shock 105kDa/110kDa protein 1, isoform CRA_a [Homo sapie	11	35	; regulation of biological process	0.998	0.858	0.848
1195860	chromosome 14 open reading frame 166, isoform CRA_b [Homo sa	4	19	n of biological process; transport	1.057	0.858	0.741
5788257	PREDICTED: kinectin isoform X6 [Homo sapiens]	9	33	al process; response to stimulus	0.891	0.858	0.909
1196027	mannosidase, alpha, class 2B, member 2, isoform CRA_b [Homo s	3	4	metabolic process	0.744	0.858	0.859
7253347	Apolipoprotein A-I binding protein [Homo sapiens]	4	16	cell organization and biogenesis	1.091	0.858	0.955
1195955	hCG27673, isoform CRA_b, partial [Homo sapiens]	2	4	metabolic process	1.111	0.857	0.719
5095962	TARSL2 protein, partial [Homo sapiens]	2	6	metabolic process	1.065	0.857	0.960
5765835	dihydrolipoyl dehydrogenase, mitochondrial isoform 2 [Homo sapi	9	43	al process; cellular homeostasis	1.117	0.857	0.846
1196214	tumor suppressing subtransferable candidate 1, isoform CRA_a [H	6	15		0.963	0.857	0.891
2210410	unnamed protein product [Homo sapiens]	3	7	cell organization and biogenesis	0.913	0.857	0.871
1025739	serine protease TADG15 [Homo sapiens]	3	5	bolic process; cell differentiation	0.745	0.856	0.865
1195876	SID1 transmembrane family, member 2, isoform CRA_c [Homo sap	2	3	; regulation of biological process	0.823	0.856	0.852
2240362	Chain F, Crystal Structure Of The Eif4a-Pdcd4 Complex	22	213	metabolic process	1.104	0.856	0.874
2702887	NADH dehydrogenase [ubiquinone] 1 beta subcomplex subunit 4 i	3	14	ic process; response to stimulus	1.038	0.855	0.879
1943796	unnamed protein product [Homo sapiens]	7	23	l proliferation; defense response	0.944	0.855	0.910
5303974	PREDICTED: phosphatidylinositol-binding clathrin assembly prote	5	17	gical process; metabolic process	1.085	0.855	0.875
5304026	PREDICTED: exportin-4 isoform X2 [Homo sapiens]	4	5	; regulation of biological process	0.795	0.855	0.829
1196170	keratin 1 (epidermolytic hyperkeratosis) [Homo sapiens]	32	383	; response to stimulus; transport	1.003	0.855	1.649
1943909	unnamed protein product [Homo sapiens]	11	30		0.944	0.855	0.827
2274888	carnosine N-methyltransferase [Homo sapiens]	2	3	metabolic process	0.935	0.855	0.865
1582600	unnamed protein product [Homo sapiens]	8	51	; regulation of biological process	0.958	0.854	0.779
1264687	unnamed protein product [Homo sapiens]	26	142		1.160	0.854	0.890
1890530	unnamed protein product [Homo sapiens]	3	5	metabolic process; transport	0.898	0.854	0.823
1195531	type I keratin 16 [Homo sapiens]	16	134	s; cellular component movement	1.079	0.854	0.913
1890537	unnamed protein product [Homo sapiens]	5	30	ization and biogenesis; transport	1.030	0.854	0.924
1195757	kinesin family member 13A, isoform CRA_c [Homo sapiens]	2	3	cell organization and biogenesis	1.253	0.853	0.756
1582597	unnamed protein product [Homo sapiens]	5	25	to stimulus; cell communication	1.137	0.853	0.879
4803121	Chain A, Structure Of The Npp2ac-alpha4 Complex	2	2		1.002	0.853	0.852
2080963	PIGO protein, partial [Homo sapiens]	2	4	metabolic process	0.931	0.853	0.879
2780883	Chain N, Proteasome Activator Reg(Alpha)	6	19		1.065	0.853	0.848
1196016	eukaryotic translation initiation factor 2B, subunit 2 beta, 39kDa [H	5	8	al process; response to stimulus	0.915	0.852	0.753
1195985	replication factor C (activator 1) 4, 37kDa, isoform CRA_b [Homo s	4	12	o stimulus; cellular homeostasis	1.117	0.852	0.956
1196123	eukaryotic translation initiation factor 3, subunit 6 48kDa, isoform	9	43	; regulation of biological process	1.102	0.852	0.891

1943742	unnamed protein product [Homo sapiens]	3	4	us; cell proliferation; cell growth	0.885	0.852	0.838
1196176	chaperonin containing TCP1, subunit 2 (beta), isoform CRA_b [Hor	14	188	ization and biogenesis; transport	1.070	0.852	0.854
1943743	unnamed protein product [Homo sapiens]	3	16	cell organization and biogenesis	0.851	0.851	0.773
2173926	hypothetical protein, partial [Homo sapiens]	3	5	rt; cellular component movement	1.004	0.851	0.923
3452770	unnamed protein product [Homo sapiens]	2	4		0.940	0.851	0.933
1265347	Proteasome (prosome, macropain) subunit, beta type, 1 [Homo sa	8	65	ent movement; defense response	0.976	0.850	0.893
7679613	PREDICTED: erlin-1 isoform X3 [Homo sapiens]	3	22	al process; response to stimulus	1.155	0.850	0.854
3452774	unnamed protein product [Homo sapiens]	2	3	; regulation of biological process	0.889	0.850	0.848
5321646	vacuolar protein-sorting-associated protein 36 isoform 3 [Homo sa	2	3	cell organization and biogenesis	0.976	0.850	0.894
2618825	CEV14, partial [Homo sapiens]	2	4	; regulation of biological process	0.904	0.849	0.853
5304247	PREDICTED: myosin regulatory light chain 12A isoform X1 [Homo	5	46	ization and biogenesis; transport	1.046	0.849	0.976
1196046	hCG2002486, isoform CRA_b [Homo sapiens]	2	6	nse response; metabolic process	0.909	0.849	0.912
3187391	hypothetical protein, partial [Homo sapiens]	6	15	t; coagulation; defense response	0.982	0.849	0.859
1196106	hCG42028, isoform CRA_a [Homo sapiens]	3	5		0.889	0.849	0.904
4158146	formin-binding protein 1-like isoform 2 [Homo sapiens]	3	7	; regulation of biological process	1.120	0.849	0.874
1196199	hypothetical protein LOC51255, isoform CRA_b [Homo sapiens]	3	6	metabolic process	0.759	0.849	0.844
1196258	steroid 5 alpha-reductase 2-like [Homo sapiens]	2	3	metabolic process	1.019	0.848	0.789
182790	alpha-L fucosidase, partial [Homo sapiens]	6	17	metabolic process	1.045	0.848	0.982
2173993	hypothetical protein, partial [Homo sapiens]	2	3	; regulation of biological process	0.725	0.848	0.786
3495878	Chain D, Crystal Structure Of Full-Length Human Peroxiredoxin 4 I	7	70	metabolic process	0.994	0.848	0.936
3825715	very-long-chain (3R)-3-hydroxyacyl-CoA dehydratase 2 [Homo sapi	2	7	metabolic process	0.828	0.848	0.769
1195704	membrane-associated ring finger (C3HC4) 5, isoform CRA_c [Hom	2	4	; regulation of biological process	1.169	0.847	0.988
7561426	Chain 3, Human Constitutive 20s Proteasome In Complex With Car	13	84		1.040	0.847	0.835
7679320	PREDICTED: N-acetylgalactosaminyltransferase 7 isoform X2 [Hom	6	10	metabolic process	0.821	0.846	0.838
1196320	RAB3 GTPase activating protein subunit 1 (catalytic), isoform CRA	2	5	biogenesis; response to stimulus	1.052	0.846	0.761
1196047	deoxyhypusine synthase, isoform CRA_a [Homo sapiens]	3	11	cell organization and biogenesis	1.141	0.846	0.893
7679924	PREDICTED: phosphoribosyl pyrophosphate synthase-associated	7	27	; regulation of biological process	1.040	0.846	0.895
7679178	PREDICTED: vigilin isoform X2 [Homo sapiens]	12	39	transport; metabolic process	0.977	0.846	0.847
28880	acid sphingomyelinase (502 AA), partial [Homo sapiens]	3	8	al process; response to stimulus	1.007	0.845	0.785
7679615	PREDICTED: sideroflexin-2 isoform X1 [Homo sapiens]	3	11	transport	0.937	0.845	0.905
7679816	PREDICTED: histone-lysine N-methyltransferase setd3 isoform X5	5	12	; regulation of biological process	1.119	0.845	0.828
8295817	Chain B, Crystal Structure Of Cladosporin In Complex With Human	8	23		1.077	0.845	0.927
1196136	melanoma antigen family D, 2, isoform CRA_e [Homo sapiens]	13	31		1.140	0.845	0.925
1196258	phosphoribosylaminoimidazole carboxylase, phosphoribosylamino	12	45	metabolic process	1.057	0.845	0.850
1196040	IMP (inosine monophosphate) dehydrogenase 1, isoform CRA_c [H	3	9	nse to stimulus; cell proliferation	0.904	0.844	0.797
4884455	hypothetical protein, partial [Homo sapiens]	2	3	transport	0.680	0.844	0.769
8288064	ubiquitin carboxyl-terminal hydrolase 14 isoform b [Homo sapiens	9	78	; regulation of biological process	0.931	0.844	0.876
7679152	PREDICTED: exportin-1 isoform X3 [Homo sapiens]	15	53	e to stimulus; metabolic process	0.940	0.843	0.863
6674849	Chain A, Structure Of The Sortilin:neurotensin Complex At Excess	4	8		1.034	0.843	0.967
9296546	KIAA1792 protein [Homo sapiens]	2	2		0.960	0.842	0.815

7807038	CDC2 protein [Homo sapiens]	4	19	; cell differentiation; cell division	0.935	0.842	0.846
4425708	Chain J, Crystal Structure Of Human Rab1b Bound To Gdp And Be	12	107	al process; response to stimulus	1.229	0.841	0.897
1971163	5'-nucleotidase domain-containing protein 2 isoform 1 [Homo sapi	12	70	metabolic process	1.037	0.841	0.810
1195796	hCG1748768, isoform CRA_b, partial [Homo sapiens]	9	102		1.217	0.841	0.870
1195778	hypothetical protein FLJ20512 [Homo sapiens]	2	13		1.007	0.841	0.918
7679641	PREDICTED: golgi-specific brefeldin A-resistance guanine nucleot	9	26	e to stimulus; metabolic process	0.953	0.841	0.851
3187333	hypothetical protein [Homo sapiens]	11	28	; regulation of biological process	0.913	0.841	0.770
3204615	adenosine kinase isoform c [Homo sapiens]	4	12	metabolic process	0.855	0.840	0.923
5489239	U3 small nucleolar RNA-associated protein 15 homolog isoform 2	2	4	; regulation of biological process	0.897	0.840	0.886
1943835	unnamed protein product [Homo sapiens]	3	7	cell organization and biogenesis	1.145	0.840	0.832
179832	calnexin [Homo sapiens]	7	26	metabolic process; transport	1.554	0.840	0.969
2133613	unnamed protein product [Homo sapiens]	3	6		0.815	0.840	0.904
3852520	Chain A, Rsk2 C-Terminal Kinase Domain With Inhibitor (E)-Methyl	2	5	metabolic process	0.854	0.840	0.757
1586247	unnamed protein product [Homo sapiens]	4	9		1.230	0.839	0.821
1677450	Chain A, Structural Basis For Substrate Fatty-Acyl Chain Specificit	18	60	metabolic process	1.070	0.839	0.891
5094949	hypothetical protein, partial [Homo sapiens]	4	15	metabolic process	0.928	0.839	0.833
1453379	LONP1 protein, partial [Homo sapiens]	5	28	e to stimulus; metabolic process	0.934	0.839	0.871
5303654	PREDICTED: probable ATP-dependent RNA helicase DDX59 isofor	3	5	metabolic process	0.939	0.839	0.795
1937850	unnamed protein product [Homo sapiens]	2	12	gical process; metabolic process	1.068	0.839	0.900
7679307	PREDICTED: signal recognition particle subunit SRP72 isoform X1	8	30	; transport; response to stimulus	1.065	0.839	0.914
5303649	PREDICTED: dual specificity protein phosphatase 23 isoform X1 [H	5	21	metabolic process	1.099	0.838	0.796
3003881	26S proteasome non-ATPase regulatory subunit 1 isoform 2 [Homo	15	67	ent movement; defense response	1.085	0.838	0.911
1943872	unnamed protein product [Homo sapiens]	9	57	; regulation of biological process	0.976	0.838	0.844
7679700	PREDICTED: heat shock cognate 71 kDa protein isoform X1 [Homo	25	661	n; cellular component movement	1.189	0.838	0.866
1943783	unnamed protein product [Homo sapiens]	9	30	metabolic process	1.102	0.838	0.892
5788014	PREDICTED: tropomyosin alpha-3 chain isoform X3 [Homo sapiens	13	76	cellular component movement	1.237	0.838	0.918
4004158	unnamed protein product, partial [Homo sapiens]	4	78		0.910	0.838	0.936
7287972	epithelial splicing regulatory protein 2 [Homo sapiens]	7	23	; regulation of biological process	0.883	0.837	0.848
1195871	copine VII, isoform CRA_b [Homo sapiens]	2	15	; transport; response to stimulus	0.942	0.837	0.796
3120324	aminoacylase-1 isoform d [Homo sapiens]	4	7	metabolic process	0.825	0.837	0.773
5304107	PREDICTED: COP9 signalosome complex subunit 3 isoform X1 [Ho	5	8	; regulation of biological process	1.277	0.837	0.870
2537220	Chain B, Human Platelet Profilin Complexed With The L-Pro10 Pep	8	290	cell organization and biogenesis	1.236	0.836	0.835
1943794	unnamed protein product [Homo sapiens]	16	87	al process; response to stimulus	1.125	0.836	0.846
1337780	LOC728395 protein [Homo sapiens]	3	11	l proliferation; cell differentiation	0.866	0.836	0.854
1890535	unnamed protein product [Homo sapiens]	10	55	se to stimulus; defense response	0.991	0.836	0.865
1244817	PCM1 protein [Homo sapiens]	4	10	; regulation of biological process	1.021	0.835	0.925
1582568	unnamed protein product [Homo sapiens]	8	31	metabolic process	0.993	0.835	0.786
5456880	GMP reductase 2 isoform 5 [Homo sapiens]	4	11	metabolic process	0.914	0.835	0.774
4680679	CGI-20 protein [Homo sapiens]	5	13	; regulation of biological process	1.040	0.835	0.855
3478639	delta-adaptin, partial CDS, partial [Homo sapiens]	7	16	; regulation of biological process	0.961	0.835	0.808

1591635	Chain A, The Solution Structure Of The Thioredoxin-Like Domain C	2	14	; regulation of biological process	1.086	0.835	0.911
3573807	Chain D, Gef Domain Of Dennd 1b In Complex With Rab Gtpase Ra	5	61	al process; response to stimulus	1.153	0.834	0.839
1015321	alanyl-tRNA synthetase [Homo sapiens]	19	114	al process; response to stimulus	1.162	0.834	0.908
1890549	unnamed protein product [Homo sapiens]	18	78	ic process; response to stimulus	1.017	0.833	0.859
1195996	DnaJ (Hsp40) homolog, subfamily C, member 13, isoform CRA_a [I	2	3	; regulation of biological process	1.282	0.833	0.858
7680391	PREDICTED: inositol polyphosphate 5-phosphatase OCRL-1 isofor	4	9	cell organization and biogenesis	0.859	0.833	0.880
8009092	low density lipoprotein receptor, partial [Homo sapiens]	2	5	al process; response to stimulus	0.894	0.833	0.775
1582607	unnamed protein product [Homo sapiens]	4	23	gical process; metabolic process	0.985	0.833	1.004
5514059	protein arginine methyltransferase 1 isoform 4 [Homo sapiens]	8	37	ic process; response to stimulus	1.181	0.833	0.933
1196185	GCN1 general control of amino-acid synthesis 1-like 1 (yeast), isof	20	37	nunication; response to stimulus	0.965	0.833	0.831
1195812	signal transducer and activator of transcription 3 (acute-phase res	6	14	cell organization and biogenesis	1.060	0.832	0.841
1196187	proteasome (prosome, macropain) 26S subunit, non-ATPase, 9, isc	2	11	cell organization and biogenesis	1.222	0.832	0.852
1937875	unnamed protein product [Homo sapiens]	6	10	transport	1.135	0.831	0.848
7679503	PREDICTED: myotubularin-related protein 9 isoform X2 [Homo sap	2	5	regulation of biological process	0.905	0.831	0.766
2968020	AGTRAP-BRAF fusion protein [Homo sapiens]	6	21	metabolic process	1.107	0.831	0.821
227448	phosphofructokinase	12	54	metabolic process	1.022	0.830	0.811
1378684	Chain B, Human Heart L-lactate Dehydrogenase H Chain, Ternary C	8	107	metabolic process	0.955	0.830	0.718
5788235	PREDICTED: V-type proton ATPase 116 kDa subunit a isoform 2 is	3	5	cell organization and biogenesis	0.940	0.830	0.844
5303721	PREDICTED: filamin-B isoform X2 [Homo sapiens]	74	348	al process; response to stimulus	0.980	0.830	0.845
1155271	PEX11A protein [Homo sapiens]	3	5	bolic process; cell differentiation	0.971	0.829	0.766
5721533	Chain N, Crystal Structure Of The Vif-cbfbeta-cul5-elob-eloc Penta	3	7		1.282	0.829	0.860
4825713	GLRX3 protein, partial [Homo sapiens]	3	9	homeostasis; metabolic process	0.901	0.829	0.847
1195789	ELAV (embryonic lethal, abnormal vision, Drosophila)-like 2 (Hu ar	2	23	regulation of biological process	0.850	0.828	0.856
1113058	Valosin-containing protein [Homo sapiens]	23	236	cell organization and biogenesis	1.057	0.828	0.877
8331837	CLCN7 protein, partial [Homo sapiens]	2	4	e to stimulus; metabolic process	0.936	0.828	0.826
6188551	Chain A, Structural Basis Of Soss1 Complex Assembly	5	7		1.073	0.827	0.974
2210406	unnamed protein product [Homo sapiens]	3	79	metabolic process	1.154	0.827	0.944
469039	nuclear factor 1 B-type, partial [Homo sapiens]	2	20	s; cellular component movement	1.087	0.827	0.965
7680376	PREDICTED: protein YIPF6 isoform X2 [Homo sapiens]	3	12		1.174	0.827	0.918
6168042	Chain B, Crystal Structure Of The Hspbp1 Core Domain Complexe	6	17		1.298	0.826	0.805
1429058	TIMM50 protein, partial [Homo sapiens]	5	14	sis; metabolic process; transport	1.257	0.826	0.958
179969	CCAAT-box-binding factor [Homo sapiens]	3	5	; regulation of biological process	1.125	0.826	0.924
8988645	TBC1 domain family member 24 isoform 2 [Homo sapiens]	4	10	; regulation of biological process	1.242	0.826	0.886
1890539	unnamed protein product [Homo sapiens]	8	47	metabolic process	1.094	0.826	0.874
1943818	unnamed protein product [Homo sapiens]	3	7	cell organization and biogenesis	1.083	0.825	0.833
3758895	FARSB protein, partial [Homo sapiens]	7	22	metabolic process	1.001	0.825	0.867
3058272	dihydropyrimidinase-like 2 [Homo sapiens]	3	10	; regulation of biological process	0.829	0.825	0.744
1196270	nuclear factor I/A, isoform CRA_a [Homo sapiens]	2	19	cell organization and biogenesis	1.034	0.825	0.987
2257356	disco-interacting protein 2 homolog A isoform f [Homo sapiens]	2	6	; regulation of biological process	1.009	0.825	0.828
1937850	unnamed protein product [Homo sapiens]	2	3		1.048	0.824	0.850

1195777	small nuclear ribonucleoprotein D2 polypeptide 16.5kDa, isoform C	2	8	d biogenesis; metabolic process	0.997	0.824	0.995
1195694	WD repeat domain 36, isoform CRA_b [Homo sapiens]	11	32	al process; response to stimulus	0.940	0.824	0.899
1195921	KIAA2013, isoform CRA_a [Homo sapiens]	6	14		0.803	0.824	0.796
2773489	deleted in autism protein 1 isoform a precursor [Homo sapiens]	3	7	logical process; cell proliferation	0.910	0.824	0.724
8894612	KIAA0851 protein, partial [Homo sapiens]	10	37	metabolic process	0.919	0.823	0.830
6282230	unknown, partial [Homo sapiens]	3	5	al process; response to stimulus	0.832	0.823	0.866
1195902	CDC42 binding protein kinase alpha (DMPK-like), isoform CRA_d [	3	3	al process; response to stimulus	0.919	0.823	0.872
2161856	EEF2K protein [Homo sapiens]	2	6	al process; response to stimulus	0.722	0.823	0.767
1865571	Chain F, Crystal Structure Of The Adenylyl Cyclase Domain Of Antl	5	130		1.098	0.823	0.870
1562090	KIAA1922 protein, partial [Homo sapiens]	2	3	; regulation of biological process	1.382	0.823	0.907
1196322	malignant T cell amplified sequence 1, isoform CRA_b [Homo sapi	2	8	al process; response to stimulus	1.220	0.823	0.862
1265456	Adenylate kinase 1 [Homo sapiens]	3	20	; regulation of biological process	0.838	0.823	0.910
1043602	unnamed protein product [Homo sapiens]	6	20		0.939	0.822	0.785
1195774	Rab acceptor 1 (prenylated), isoform CRA_a [Homo sapiens]	2	9		0.860	0.822	0.763
1195702	hCG1642531 [Homo sapiens]	2	30	metabolic process	0.844	0.822	0.819
7561426	Chain 0, Human Constitutive 20s Proteasome In Complex With Car	8	48		0.913	0.822	0.819
4098006	unnamed protein product, partial [Homo sapiens]	2	17		1.099	0.822	0.878
3891520	Chain D, Human Glyoxalase I Q33e, E172q Double Mutant	5	10		1.137	0.821	0.947
1943813	unnamed protein product [Homo sapiens]	2	4	cell organization and biogenesis	0.890	0.821	0.716
7679314	PREDICTED: heat shock 70 kDa protein 4L isoform X2 [Homo sapi	7	17	ic process; response to stimulus	0.998	0.821	0.866
1890653	unnamed protein product [Homo sapiens]	7	49	ization and biogenesis; transport	1.020	0.821	0.781
1196187	hCG2045883 [Homo sapiens]	2	3	transport	0.921	0.821	0.856
2210450	unnamed protein product [Homo sapiens]	4	13		0.910	0.821	0.981
1582614	unnamed protein product [Homo sapiens]	3	3	al process; response to stimulus	1.053	0.821	0.764
7679952	PREDICTED: pyridoxine-5'-phosphate oxidase isoform X2 [Homo s	3	10	gical process; metabolic process	1.114	0.821	0.795
1501055	heat shock protein gp96 precursor, partial [Homo sapiens]	26	452	nd biogenesis; defense response	1.173	0.821	0.927
7679819	PREDICTED: nuclear export mediator factor NEMF isoform X3 [Hon	5	13	transport	1.014	0.821	0.873
4825596	UTP--glucose-1-phosphate uridylyltransferase isoform a [Homo sa	8	26	metabolic process	0.945	0.820	0.818
1195785	solute carrier family 11 (proton-coupled divalent metal ion transpo	2	4	cell organization and biogenesis	0.794	0.820	0.746
551547	prosome beta-subunit [Homo sapiens]	5	14	ent movement; defense response	0.944	0.820	0.834
4884627	cytokine receptor-like molecule 9 [Homo sapiens]	4	20	regulation of biological process	1.067	0.820	0.842
5304141	PREDICTED: 3-ketodihydrosphingosine reductase isoform X2 [Hon	2	4	metabolic process	1.359	0.820	0.911
1196086	chromosome 9 open reading frame 86, isoform CRA_a [Homo sapi	3	6	al process; response to stimulus	0.887	0.820	0.863
5304044	PREDICTED: protein sel-1 homolog 1 isoform X1 [Homo sapiens]	9	29	; response to stimulus; transport	1.053	0.820	0.800
2726263	nuclear autoantigenic sperm protein isoform 3 [Homo sapiens]	5	22	; transport; response to stimulus	0.958	0.819	0.798
7679228	PREDICTED: calpain-7 isoform X3 [Homo sapiens]	2	3	gical process; metabolic process	1.159	0.819	0.797
3882327	KIAA0803 protein, partial [Homo sapiens]	2	3	ent movement; defense response	0.948	0.819	0.816
1179387	protein GNAS isoform g [Homo sapiens]	14	72	esponse to stimulus; coagulation	1.362	0.819	0.847
1195944	echinoderm microtubule associated protein like 3, isoform CRA_c	6	11		1.086	0.819	0.855
7679935	PREDICTED: 2',3'-cyclic-nucleotide 3'-phosphodiesterase isoform	12	68	se to stimulus; cell differentiation	1.124	0.819	0.859

1943776	unnamed protein product [Homo sapiens]	5	11	transport; metabolic process	0.850	0.819	0.750
1195968	solute carrier family 12 (potassium/chloride transporters), member	4	10	transport; cellular homeostasis	0.940	0.818	0.842
1195932	hCG2004980, isoform CRA_i [Homo sapiens]	2	4	; regulation of biological process	1.074	0.818	0.849
1195950	NADH dehydrogenase (ubiquinone) flavoprotein 1, 51kDa, isoform	6	13	metabolic process	1.021	0.818	0.872
4593587	Chain B, Crystal Structure Of Mst4-mo25 Complex With Dki	2	3		1.081	0.818	0.830
1943845	unnamed protein product [Homo sapiens]	4	9	lic process; cell death; transport	0.950	0.818	0.817
2850022	cytochrome c-type heme lyase [Homo sapiens]	2	4	metabolic process	0.988	0.818	0.827
5577960	Po66 carbohydrate binding protein [Homo sapiens]	5	9	; regulation of biological process	0.958	0.817	0.751
1890538	unnamed protein product [Homo sapiens]	7	36	erentiation; cellular homeostasis	1.134	0.816	0.908
1752960	protein kinase C inhibitor-2 [Homo sapiens]	2	12	metabolic process; cell death	1.099	0.816	0.931
1890541	unnamed protein product [Homo sapiens]	3	5	cell death; response to stimulus	0.848	0.816	0.752
1937859	unnamed protein product [Homo sapiens]	2	7	; regulation of biological process	1.193	0.815	0.848
1195834	thioredoxin-like 1, isoform CRA_b [Homo sapiens]	2	6	; regulation of biological process	1.631	0.815	0.939
1890674	unnamed protein product [Homo sapiens]	9	42	al process; response to stimulus	1.040	0.815	0.870
5031981	26S proteasome non-ATPase regulatory subunit 14 [Homo sapiens	3	6	ent movement; defense response	0.845	0.815	0.868
1195903	translin-associated factor X, isoform CRA_b [Homo sapiens]	3	3	; regulation of biological process	1.172	0.815	0.849
1582618	unnamed protein product [Homo sapiens]	15	116	se to stimulus; defense response	1.025	0.815	0.793
4128142	basic leucine zipper and W2 domain-containing protein 1 isoform 4	5	11	; regulation of biological process	0.965	0.814	0.868
1937860	unnamed protein product [Homo sapiens]	6	25	e to stimulus; metabolic process	0.896	0.813	0.854
1195909	5-aminoimidazole-4-carboxamide ribonucleotide formyltransferase	18	77	ic process; response to stimulus	1.059	0.813	0.859
7679629	PREDICTED: nuclear factor NF-kappa-B p100 subunit isoform X2 [	6	13	cell organization and biogenesis	0.932	0.813	0.852
2997583	dynamin-2 isoform 5 [Homo sapiens]	15	50	cell organization and biogenesis	1.053	0.813	0.907
1196258	phosphoribosyl pyrophosphate amidotransferase, isoform CRA_a	7	39	cell organization and biogenesis	1.010	0.813	0.870
5890581	vacuolar protein sorting-associated protein 11 homolog isoform 2	2	3	cell organization and biogenesis	0.908	0.812	0.850
1534199	Unknown (protein for IMAGE:4347401), partial [Homo sapiens]	14	223	f biological process; cell division	1.163	0.812	0.842
7129729	DNAJB6 protein [Homo sapiens]	2	13	se to stimulus; cell differentiation	0.950	0.812	0.882
1196194	hCG1983058 [Homo sapiens]	2	9	metabolic process	0.833	0.812	0.763
1943780	unnamed protein product [Homo sapiens]	3	9	se to stimulus; defense response	0.856	0.811	0.873
8874929	Chain b, Structure Of The Hcv Ires Bound To The Human Ribosom	2	13		0.935	0.811	0.678
1327920	ALG3 protein, partial [Homo sapiens]	2	5	metabolic process	1.261	0.811	0.808
2086100	atlastin-2 isoform 2 [Homo sapiens]	4	6	cell organization and biogenesis	0.819	0.811	0.737
4098140	unnamed protein product, partial [Homo sapiens]	2	10		0.891	0.810	0.831
2234616	Leucine rich repeat containing 26 [Homo sapiens]	2	4	transport	0.854	0.810	0.812
1195980	talin 2, isoform CRA_c [Homo sapiens]	7	26	cell organization and biogenesis	1.021	0.810	0.873
1195920	FK506 binding protein 12-rapamycin associated protein 1, isoform	4	9	nd biogenesis; defense response	0.956	0.809	0.835
4004167	unnamed protein product, partial [Homo sapiens]	4	13		0.970	0.808	0.871
7679317	PREDICTED: G-rich sequence factor 1 isoform X1 [Homo sapiens]	3	19	metabolic process	1.010	0.808	0.873
1943746	unnamed protein product [Homo sapiens]	2	4	al process; response to stimulus	0.923	0.808	0.853
4004226	unnamed protein product, partial [Homo sapiens]	3	9		0.814	0.808	0.810
3169831	cytochrome b-c1 complex subunit 7 isoform 2 [Homo sapiens]	3	18	metabolic process	1.022	0.807	0.946

1943743	unnamed protein product [Homo sapiens]	4	17	metabolic process	1.086	0.807	0.790
5303769	PREDICTED: ankyrin repeat domain-containing protein 17 isoform	4	7	e response; response to stimulus	0.812	0.806	0.770
7679423	PREDICTED: cyclin-dependent kinase 19 isoform X3 [Homo sapien	2	8	al process; response to stimulus	0.880	0.806	0.870
1582547	unnamed protein product [Homo sapiens]	9	47	metabolic process	0.979	0.806	0.843
1196083	G protein-coupled receptor 107, isoform CRA_e [Homo sapiens]	2	4	transport	0.914	0.806	0.852
1195816	proteasome (prosome, macropain) 26S subunit, non-ATPase, 13, is	11	33	ent movement; defense response	1.102	0.806	0.895
1890553	unnamed protein product [Homo sapiens]	6	76	f biological process; cell division	1.186	0.806	0.855
2397820	Chain D, Human Sahh-Like Domain Of Human Adenosylhomocyste	8	44	metabolic process; transport	0.977	0.806	0.935
2569851	TRAM adaptor with GOLD domain isoform 2 precursor [Homo sapi	6	25	transport	1.040	0.806	0.840
6253114	LRRC41 protein, partial [Homo sapiens]	2	3	metabolic process	0.973	0.806	0.853
5469635	protein phosphatase 1, catalytic subunit, beta isoform [Homo sapi	6	42	sponse to stimulus; cell division	1.043	0.805	0.784
1195844	hCG1995701, isoform CRA_d [Homo sapiens]	6	38	nd biogenesis; defense response	1.100	0.805	0.844
1196152	succinate dehydrogenase complex, subunit B, iron sulfur (Ip), isof	6	31	metabolic process	1.030	0.805	0.784
1196075	hypothetical protein FLJ20397, isoform CRA_e [Homo sapiens]	4	7	cell organization and biogenesis	1.128	0.805	0.831
7679729	PREDICTED: citron Rho-interacting kinase isoform X7 [Homo sapi	4	7	se to stimulus; cell differentiation	0.683	0.805	0.781
1277982	Proteasome (prosome, macropain) 26S subunit, non-ATPase, 8 [Ho	5	18	nd biogenesis; defense response	1.199	0.804	0.896
1196270	hook homolog 1 (Drosophila), isoform CRA_b [Homo sapiens]	3	6	genesis; development; transport	1.256	0.804	0.893
1196170	keratin 5 (epidermolysis bullosa simplex, Dowling-Meara/Kobner/W	24	146	cell organization and biogenesis	0.987	0.804	1.180
7200629	Chain D, Lactate Dehydrogenase In Complex With Inhibitor Compo	16	300	metabolic process	1.075	0.804	0.750
5788069	PREDICTED: structural maintenance of chromosomes protein 4 is	4	6	; metabolic process; cell division	0.999	0.804	0.869
2443869	epsilon-COP, partial [Homo sapiens]	3	34	cell organization and biogenesis	1.249	0.804	0.802
1196263	COP9 constitutive photomorphogenic homolog subunit 4 (Arabido	8	33	biogenesis; response to stimulus	1.001	0.804	0.811
3025661	Chain B, Crystal Structure Of An Rb C-Terminal Peptide Bound To	7	51		0.961	0.804	0.784
1195823	diaphanous homolog 1 (Drosophila), isoform CRA_a [Homo sapier	7	12	al process; response to stimulus	0.991	0.803	0.819
6634421	putative protein TH1, partial [Homo sapiens]	3	5	; regulation of biological process	0.898	0.803	0.922
5303606	PREDICTED: protein LZIC isoform X1 [Homo sapiens]	2	6	response to stimulus	0.847	0.803	0.802
4088963	Chain A, Structure Of Arf1-Gdp Bound To Sec7 Domain Complexe	8	112	al process; response to stimulus	1.222	0.803	0.841
7561426	Chain y, Human Constitutive 20s Proteasome In Complex With Car	4	13		0.915	0.802	0.796
2414516	surface 4 integral membrane protein, partial [Homo sapiens]	5	27	n of biological process; transport	1.145	0.802	0.795
1943803	unnamed protein product [Homo sapiens]	4	12	; regulation of biological process	1.027	0.802	0.801
1195819	NudC domain containing 2 [Homo sapiens]	2	5		0.943	0.802	0.696
1196177	oxysterol binding protein-like 8, isoform CRA_a, partial [Homo sap	3	5	cell organization and biogenesis	1.025	0.802	0.836
1196192	phosphoribosyl pyrophosphate synthetase 2, isoform CRA_b [Hon	6	19	metabolic process	1.190	0.801	0.783
6941068	c371H6.2 (similar to NDP kinase), partial [Homo sapiens]	7	23	; regulation of biological process	0.942	0.801	0.793
2174866	FLJ00410 protein, partial [Homo sapiens]	3	11		0.801	0.800	0.674
5788026	PREDICTED: bifunctional methylenetetrahydrofolate dehydrogenas	3	3	metabolic process	0.936	0.800	0.860
1196308	hCG39342, isoform CRA_d [Homo sapiens]	3	5	cell organization and biogenesis	0.884	0.800	0.719
1960499	Chain D, Crystal Structure Of Human Fumarate Hydratase	8	44	metabolic process	1.103	0.799	0.818
2143474	beige-like protein [Homo sapiens]	32	105	transport	1.084	0.799	0.786
7561425	Chain u, Human Constitutive 20s Proteasome In Complex With Car	5	37		1.052	0.799	0.788

3767441	unknown [Homo sapiens]	24	256	; regulation of biological process	1.003	0.798	0.805
3478599	PPP2R2D protein, partial [Homo sapiens]	4	7	sponse to stimulus; cell division	1.124	0.798	0.835
6298895	unknown [Homo sapiens]	7	37	metabolic process	1.034	0.798	0.757
4004133	unnamed protein product, partial [Homo sapiens]	2	9		1.029	0.797	0.782
5304127	PREDICTED: signal transducer and activator of transcription 5B is	3	5	al process; response to stimulus	1.072	0.797	0.784
181238	cytochrome c1, partial [Homo sapiens]	5	70	ic process; response to stimulus	0.789	0.797	0.808
2661071	similar to 26S proteasome subunit p45 [Homo sapiens]	7	58	ent movement; defense response	1.188	0.797	0.894
7020132	unnamed protein product [Homo sapiens]	2	7	metabolic process	1.013	0.797	0.971
1195869	chromosome 18 open reading frame 55, isoform CRA_b [Homo sa	4	10	d biogenesis; metabolic process	1.150	0.796	0.862
1943860	unnamed protein product [Homo sapiens]	4	19	regulation of biological process	1.349	0.796	0.846
1890654	unnamed protein product [Homo sapiens]	12	74	cell organization and biogenesis	0.860	0.796	0.790
2965313	histone lysine demethylase PHF8 isoform 4 [Homo sapiens]	2	4	; regulation of biological process	1.024	0.796	0.769
1195994	coatomer protein complex, subunit beta 2 (beta prime), isoform CF	30	129	cell organization and biogenesis	1.125	0.795	0.785
2210418	unnamed protein product [Homo sapiens]	4	18	bolic process; cell differentiation	0.782	0.795	0.753
1582592	unnamed protein product [Homo sapiens]	10	42	al process; response to stimulus	1.042	0.795	0.803
2559583	perilipin-3 isoform 3 [Homo sapiens]	4	17	transport	0.919	0.795	0.727
854103	chloride channel 3 [Homo sapiens]	3	7	al process; cellular homeostasis	1.143	0.794	0.863
4128156	WD repeat-containing protein 37 [Homo sapiens]	3	4		0.998	0.794	0.853
1943898	unnamed protein product [Homo sapiens]	2	3		0.708	0.794	0.872
1943886	unnamed protein product [Homo sapiens]	3	8	; regulation of biological process	0.779	0.794	0.773
7535342	Chain A, Importin Alpha 3 Delta Ibb In Complex With Influenza Pb2	3	7		1.013	0.793	0.886
2210437	unnamed protein product [Homo sapiens]	6	23	ic process; response to stimulus	1.005	0.793	0.785
5788050	PREDICTED: protein TANC1 isoform X17 [Homo sapiens]	9	18	cell organization and biogenesis	1.024	0.793	0.773
4945655	RABGGTA, partial [Homo sapiens]	10	21	metabolic process	0.940	0.793	0.815
1645195	Chain Y, Crystal Structure Of Human Peroxiredoxin I In Complex W	2	5		1.017	0.793	0.701
1943759	unnamed protein product [Homo sapiens]	4	7	metabolic process; transport	1.150	0.792	0.909
1162834	PSMA7 protein, partial [Homo sapiens]	7	50	ent movement; defense response	1.321	0.792	0.896
6739602	talin [Homo sapiens]	36	132	e to stimulus; metabolic process	0.987	0.792	0.795
1195839	protein phosphatase 2 (formerly 2A), regulatory subunit B (PR 52),	8	22	al process; response to stimulus	1.181	0.792	0.841
2891913	golgin subfamily A member 3 isoform 2 [Homo sapiens]	6	16	; regulation of biological process	1.016	0.792	0.849
1195682	chromosome 6 open reading frame 55, isoform CRA_b [Homo sapi	3	6	cell organization and biogenesis	0.991	0.791	0.877
7680016	PREDICTED: bromodomain-containing protein 4 isoform X2 [Homo	2	11	ic process; response to stimulus	1.191	0.791	0.865
7680218	PREDICTED: cystathionine beta-synthase isoform X5 [Homo sapie	9	55	nunication; response to stimulus	1.143	0.791	0.857
1943825	unnamed protein product [Homo sapiens]	6	14	; regulation of biological process	1.042	0.791	0.805
1751225	C8orf55 protein [Homo sapiens]	7	20		1.069	0.790	0.625
1937869	unnamed protein product [Homo sapiens]	2	4	; regulation of biological process	1.170	0.790	0.781
5303856	PREDICTED: leucine-rich repeat and WD repeat-containing protein	2	6	cell organization and biogenesis	0.984	0.790	0.728
1890548	unnamed protein product [Homo sapiens]	4	56	ion; transport; defense response	0.993	0.790	0.825
3801485	DDX54 protein, partial [Homo sapiens]	3	8	al process; response to stimulus	0.883	0.790	0.827
1196102	tubulin-specific chaperone d, isoform CRA_c [Homo sapiens]	19	81	; regulation of biological process	1.026	0.789	0.796

1578299	Chain A, N-Terminal Actin-Crosslinking Domain From Human Fimb	2	7		0.974	0.788	0.912
7739468	diphosphoinositol polyphosphate phosphohydrolase type 2 alpha,	3	7	; response to stimulus; transport	1.242	0.788	0.904
1196264	methionyl aminopeptidase 1, isoform CRA_a [Homo sapiens]	3	10	ic process; response to stimulus	1.107	0.788	0.784
1890532	unnamed protein product [Homo sapiens]	7	91	al process; response to stimulus	1.348	0.788	0.833
9677391	unnamed protein product [Homo sapiens]	7	83		1.184	0.788	0.756
1082503	unnamed protein product [Homo sapiens]	4	18		0.917	0.788	0.886
5083424	unnamed protein product [Homo sapiens]	3	15		1.195	0.788	0.721
1943910	unnamed protein product [Homo sapiens]	2	5	ss; metabolic process; transport	0.879	0.788	0.831
5788028	PREDICTED: DNA-directed RNA polymerase I subunit RPA1 isofor	2	4	; regulation of biological process	0.940	0.788	0.797
2837391	Chain C, Crystal Structure Of Human Saposin B	2	9		1.107	0.787	0.759
1890551	unnamed protein product [Homo sapiens]	2	10	; regulation of biological process	0.881	0.787	0.727
3945821	serine/threonine-protein phosphatase 2A 56 kDa regulatory subun	4	14	bolic process; defense response	0.868	0.787	0.730
1195904	geranylgeranyl diphosphate synthase 1, isoform CRA_a [Homo sa	2	3	metabolic process	0.878	0.787	0.758
1195946	stress-induced-phosphoprotein 1 (Hsp70/Hsp90-organizing protei	15	93	response to stimulus	1.054	0.787	0.784
5269597	Chain A, Crystal Structure Of A Protein Phosphatase-1: Calcineuri	6	41		0.960	0.787	0.784
1943761	unnamed protein product [Homo sapiens]	3	10		1.037	0.786	0.806
1992366	BRI3-binding protein precursor [Homo sapiens]	3	19		1.134	0.785	0.816
4498027	Chain D, Crystal Structure Of Coactivator-associated Arginine Met	7	26		1.042	0.785	0.821
1738950	CHORDC1 protein [Homo sapiens]	2	4	gical process; metabolic process	1.071	0.784	0.711
1890531	unnamed protein product [Homo sapiens]	3	23	ization and biogenesis; transport	1.041	0.784	0.917
2172728	choline dehydrogenase, mitochondrial [Homo sapiens]	11	38	metabolic process	0.961	0.784	0.821
6399167	unknown [Homo sapiens]	2	43	ic process; response to stimulus	1.030	0.784	0.802
1943749	unnamed protein product [Homo sapiens]	2	4		0.983	0.784	0.809
3296658	Chain A, Crystal Structure Of Human S-adenosylmethionine Synth	4	7	metabolic process	1.045	0.784	0.650
1521480	TRNT1 protein [Homo sapiens]	4	10	metabolic process; transport	0.818	0.783	0.842
1195862	hCG2033479, isoform CRA_d, partial [Homo sapiens]	7	15	ization and biogenesis; transport	0.862	0.783	0.834
1943869	unnamed protein product [Homo sapiens]	4	10	; regulation of biological process	1.118	0.783	0.821
1582602	unnamed protein product [Homo sapiens]	4	9	; regulation of biological process	1.140	0.783	0.847
1196315	GTP-binding protein PTD004, isoform CRA_c [Homo sapiens]	2	16	metabolic process; transport	1.382	0.783	0.882
1943898	unnamed protein product [Homo sapiens]	3	60	; regulation of biological process	0.806	0.783	0.767
1195960	CDC91 cell division cycle 91-like 1 (S. cerevisiae), isoform CRA_c [	2	8	; regulation of biological process	0.925	0.783	0.812
1310882	Chain A, Cyclophilin B Complexed With [d-(Cholinylester)ser8]-Cyc	9	78	metabolic process	1.143	0.782	0.816
1196112	IQ motif and WD repeats 1, isoform CRA_d [Homo sapiens]	2	4	; regulation of biological process	0.855	0.782	0.772
5788136	PREDICTED: aminoacyl tRNA synthase complex-interacting multifu	2	11	gical process; cell differentiation	0.915	0.781	0.839
1703319	RecName: Full=Annexin A4; AltName: Full=35-beta calcimedin; Alt	14	92	e to stimulus; cell differentiation	1.240	0.781	0.847
2133561	unnamed protein product [Homo sapiens]	5	15		0.903	0.781	0.732
1195945	reticulon 3, isoform CRA_a [Homo sapiens]	3	30	cell organization and biogenesis	0.959	0.781	0.710
1429057	RBM17 protein, partial [Homo sapiens]	2	9	metabolic process	0.754	0.781	0.791
2207025	Chain C, TapasinERP57 HETERODIMER	14	236	; regulation of biological process	1.245	0.781	0.842
1196087	ectonucleoside triphosphate diphosphohydrolase 2, isoform CRA_	7	39	cell organization and biogenesis	0.966	0.780	0.707

1196231	phosphoribosyl pyrophosphate synthetase 1, isoform CRA_a [Hom	8	25	metabolic process; cell death	1.225	0.780	0.759
3478505	ACBD3 protein, partial [Homo sapiens]	3	5	metabolic process; transport	1.188	0.780	0.804
7679770	PREDICTED: DNA polymerase epsilon catalytic subunit A isoform	2	3	lic process; cellular homeostasis	1.042	0.780	0.829
1195904	HEAT repeat containing 1, isoform CRA_b [Homo sapiens]	7	18	; regulation of biological process	1.106	0.779	0.906
7679883	PREDICTED: exportin-6 isoform X5 [Homo sapiens]	4	7	transport	1.057	0.779	0.873
1195697	peroxiredoxin 3, isoform CRA_a, partial [Homo sapiens]	6	74	bolic process; cell differentiation	0.991	0.779	0.922
3886040	Chain p, Crystalstructure Of The Ubiquitin Protease	5	61		1.112	0.778	0.779
3857191	caseinolytic peptidase B protein homolog isoform 2 [Homo sapien	3	13	ic process; response to stimulus	0.940	0.778	0.729
7679803	PREDICTED: ERO1-like protein alpha isoform X1 [Homo sapiens]	8	23	sis; cell differentiation; transport	1.249	0.778	0.847
1195680	villin 2 (ezrin), isoform CRA_a [Homo sapiens]	23	207	ifferentiation; metabolic process	1.049	0.777	0.809
7679964	PREDICTED: glucose-6-phosphatase 3 isoform X1 [Homo sapiens]	2	4	metabolic process; transport	1.073	0.777	0.862
7679264	PREDICTED: golgin subfamily B member 1 isoform X9 [Homo sapi	7	16	cell organization and biogenesis	0.879	0.776	0.807
5565033	nucleolar protein 9 isoform 2 [Homo sapiens]	4	22		0.818	0.776	0.805
1196269	asparagine-linked glycosylation 6 homolog (yeast, alpha-1,3-gluco	3	5	metabolic process	1.118	0.776	0.793
3856482	isocitrate dehydrogenase [NAD] subunit beta, mitochondrial isofor	5	10	metabolic process	1.040	0.775	0.694
5303909	PREDICTED: golgin subfamily A member 2 isoform X3 [Homo sapi	12	44	; regulation of biological process	1.047	0.774	0.719
3844755	histidine--tRNA ligase, cytoplasmic isoform 4 [Homo sapiens]	7	31	cell organization and biogenesis	1.026	0.773	0.824
1890654	unnamed protein product [Homo sapiens]	9	44	al process; response to stimulus	1.061	0.773	0.771
1196217	signal transducer and activator of transcription 3 interacting prote	7	16	d biogenesis; metabolic process	0.956	0.773	0.781
1893396	Chain A, Crystal Structure Of Human Catechol O-Methyltransferase	4	11	metabolic process	1.545	0.772	0.782
1943895	unnamed protein product [Homo sapiens]	2	3	; regulation of biological process	1.327	0.771	0.799
1890655	unnamed protein product [Homo sapiens]	29	129	; regulation of biological process	1.180	0.771	0.768
5788111	PREDICTED: endoplasmic reticulum-Golgi intermediate compartm	8	31	transport	0.950	0.770	0.764
4004233	unnamed protein product, partial [Homo sapiens]	2	25		0.878	0.770	0.792
1196090	O-acyltransferase (membrane bound) domain containing 5, isoforn	3	5	; regulation of biological process	1.241	0.770	0.805
1196059	hCG41030 [Homo sapiens]	3	21	metabolic process	0.976	0.770	0.707
9311398	Chain A, Crystal Structure Of 2,2'-(naphthalene-1,4-diylbis(((4-meth	2	3		0.953	0.769	0.767
1043562	unnamed protein product [Homo sapiens]	2	9		1.065	0.768	0.725
2118262	PRKCSH protein, partial [Homo sapiens]	7	47	se to stimulus; defense response	1.092	0.768	0.743
2161879	Unknown (protein for IMAGE:5116230), partial [Homo sapiens]	5	9	transport	0.886	0.768	0.790
3134828	biogenesis of lysosome-related organelles complex 1 subunit 5 isc	2	2	; regulation of biological process	1.146	0.768	0.884
1195819	arginyl-tRNA synthetase [Homo sapiens]	15	83	metabolic process	0.913	0.767	0.798
1196300	tryptophan rich basic protein, isoform CRA_b [Homo sapiens]	3	6		0.880	0.767	0.732
1224840	cisplatin resistance related protein CRR9p [Homo sapiens]	5	11	cell death	0.955	0.765	0.753
1404389	EIF2S2 protein [Homo sapiens]	4	21	on; metabolic process; transport	1.263	0.765	0.861
3920559	EGLN1, partial [Homo sapiens]	2	4	; regulation of biological process	0.981	0.764	0.780
2175387	unnamed protein product [Homo sapiens]	2	6	cell organization and biogenesis	0.971	0.764	0.787
5288810	electron transfer flavoprotein-ubiquinone oxidoreductase, mitocho	4	17	e to stimulus; metabolic process	0.945	0.764	0.820
1196036	vacuolar protein sorting 4A (yeast), isoform CRA_a [Homo sapiens	3	12	f biological process; cell division	1.204	0.763	0.859
5254587	hypothetical protein, partial [Homo sapiens]	4	6	metabolic process	1.152	0.763	0.831

6808243	hypothetical protein, partial [Homo sapiens]	9	43	regulation of biological process	0.899	0.762	0.775
6208943	ralA binding protein 1 variant, partial [Homo sapiens]	2	6	; regulation of biological process	0.938	0.762	0.798
6598354	Chain A, Vesiculoviral Matrix (m) Protein Occupies Nucleic Acid Bi	2	14		1.250	0.762	0.809
4088121	unnamed protein product [Homo sapiens]	3	5		1.097	0.762	0.754
1592910	MARS protein, partial [Homo sapiens]	13	56	metabolic process	1.064	0.761	0.762
7680254	PREDICTED: ran GTPase-activating protein 1 isoform X1 [Homo sa	5	40	al process; response to stimulus	1.055	0.761	0.775
1195718	tight junction protein 1 (zona occludens 1), isoform CRA_c [Homo	8	28	to stimulus; cell communication	0.905	0.761	0.794
1937835	unnamed protein product [Homo sapiens]	2	4	; regulation of biological process	1.227	0.761	0.828
4491129	Chain D, Crystal Structures Of Native And Inhibited Forms Of Hum	6	24	metabolic process	0.926	0.761	0.765
1265337	Phosphoglycerate mutase 1 (brain) [Homo sapiens]	5	60	; regulation of biological process	1.004	0.760	0.723
1943890	unnamed protein product [Homo sapiens]	3	17	cell organization and biogenesis	0.974	0.760	0.784
1195830	phosphoserine aminotransferase 1, isoform CRA_d [Homo sapiens	6	27	metabolic process	1.047	0.760	0.781
2234602	KIAA1244 [Homo sapiens]	14	35	n of biological process; transport	1.006	0.759	0.747
5304257	PREDICTED: 1-phosphatidylinositol 4,5-bisphosphate phosphodie	2	5	al process; response to stimulus	0.817	0.758	0.756
1584292	Chain A, Crystal Structure Of The Selenocysteine To Cysteine Muta	3	12	e to stimulus; metabolic process	1.055	0.758	0.810
5303901	PREDICTED: endoplasmic reticulum metallopeptidase 1 isoform X	5	13	metabolic process	1.001	0.757	0.793
1082501	unnamed protein product [Homo sapiens]	2	5		0.809	0.757	0.668
6841170	HSPC260, partial [Homo sapiens]	2	7	cell organization and biogenesis	0.842	0.756	0.822
1793951	N-acetylneuraminic acid synthase [Homo sapiens]	3	10	metabolic process	1.167	0.756	0.781
7680117	PREDICTED: alpha-soluble NSF attachment protein isoform X2 [Ho	2	12	cell organization and biogenesis	1.109	0.755	0.778
1943888	unnamed protein product [Homo sapiens]	2	4	d biogenesis; metabolic process	1.025	0.755	0.712
1943811	unnamed protein product [Homo sapiens]	4	12	lifferentiation; metabolic process	0.908	0.755	0.853
1682030	gastric cancer hepatocellular carcinoma suppressor 1 variant [Hor	2	4	transport	0.757	0.754	0.775
1196209	sorting nexin 17, isoform CRA_c [Homo sapiens]	2	4	al process; response to stimulus	0.856	0.754	0.715
1265445	RecQ protein-like (DNA helicase Q1-like) [Homo sapiens]	9	27	ic process; response to stimulus	1.016	0.754	0.671
1151445	Chain H, Glutathione Transferase P1-1	6	54		1.062	0.754	0.692
4711517	STX12, partial [Homo sapiens]	3	7	; regulation of biological process	1.112	0.754	0.868
7679019	PREDICTED: nuclear migration protein nudC isoform X2 [Homo sa	4	17	nt; cell proliferation; cell division	1.124	0.753	0.755
1195899	ATP synthase, H+ transporting, mitochondrial F1 complex, delta su	2	26	; transport; response to stimulus	1.042	0.753	0.703
4712341	RPLP0 protein [Homo sapiens]	8	40	ization and biogenesis; transport	0.942	0.752	0.793
5788075	PREDICTED: DNA-directed RNA polymerases I, II, and III subunit R	3	42	gical process; defense response	1.245	0.751	0.809
3849254	Chain D, Human TfiiaTBPDNA COMPLEX	2	3	metabolic process	0.870	0.751	0.810
1195757	guanosine monophosphate reductase, isoform CRA_a [Homo sapi	2	5	ic process; response to stimulus	0.897	0.751	0.730
5147614	hypothetical protein [Homo sapiens]	11	41	; regulation of biological process	0.873	0.749	0.703
7546546	Chain B, Crystal Structure Of Human Cathepsin X	3	24	metabolic process	0.984	0.748	0.789
5577861	probable palmitoyltransferase ZDHHC20 isoform 2 [Homo sapiens]	2	3	sis; transport; metabolic process	0.850	0.748	0.648
7756755	TMX3 protein, partial [Homo sapiens]	3	8	; regulation of biological process	0.875	0.748	0.786
1425058	Protein-L-isoaspartate (D-aspartate) O-methyltransferase [Homo sa	4	34	metabolic process	0.906	0.748	0.731
1195856	glycosyltransferase 8 domain containing 1, isoform CRA_a [Homo	3	12	metabolic process	1.109	0.748	0.789
5304064	PREDICTED: tropomyosin alpha-1 chain isoform X13 [Homo sapier	13	123	biogenesis; response to stimulus	1.222	0.747	0.843

1196121	phosphatidylserine synthase 1, isoform CRA_b [Homo sapiens]	3	6	metabolic process	0.855	0.747	0.733
1943913	unnamed protein product [Homo sapiens]	11	48	metabolic process	0.934	0.747	0.733
1196105	trafficking protein particle complex 1, isoform CRA_a [Homo sapie	2	2	cell organization and biogenesis	0.874	0.747	0.889
7680078	PREDICTED: echinoderm microtubule-associated protein-like 2 iso	4	11	regulation of biological process	1.053	0.747	0.761
1655039	unnamed protein product [Homo sapiens]	2	3		0.872	0.746	0.691
1195762	synaptobrevin-like 1, isoform CRA_c [Homo sapiens]	3	8	cell organization and biogenesis	1.073	0.746	0.807
1196068	calmodulin-like 5 [Homo sapiens]	6	274	al process; response to stimulus	1.295	0.746	0.808
3109005	unnamed protein product [Homo sapiens]	24	234		0.980	0.746	0.782
2747713	nuclear pore membrane glycoprotein 210 precursor [Homo sapiens	25	112	al process; response to stimulus	1.028	0.745	0.797
1195845	nucleoporin 210kDa, isoform CRA_b [Homo sapiens]	25	108	al process; response to stimulus	1.023	0.745	0.784
7679740	PREDICTED: apoptotic protease-activating factor 1 isoform X4 [Ho	2	5	cell organization and biogenesis	0.957	0.745	0.735
1663700	KIAA0241, partial [Homo sapiens]	4	6	cellular component movement	0.974	0.745	0.747
7679947	PREDICTED: keratin, type I cytoskeletal 15 isoform X3 [Homo sapi	13	129		0.978	0.745	0.710
6102941	hypothetical protein, partial [Homo sapiens]	2	3	; regulation of biological process	0.793	0.745	0.695
6746393	Chain A, Crystal Structure Of Isg15, The Interferon-Induced Ubiqui	2	5		0.792	0.744	0.734
7043608	Chain N, Crystal Structure Of The Ring1b/bmi1/ubch5c Prc1 Ubiqu	4	27		1.013	0.744	0.748
1196098	phosphatidylserine receptor, isoform CRA_a [Homo sapiens]	2	4	se to stimulus; cell differentiation	0.854	0.744	0.666
1265455	Translocator protein (18kDa) [Homo sapiens]	3	14	cell organization and biogenesis	0.881	0.744	0.785
7679736	PREDICTED: palmitoyltransferase ZDHHC17 isoform X4 [Homo sa	2	2	us; metabolic process; transport	0.884	0.744	0.772
1196145	ubiquitin-conjugating enzyme E2D 4 (putative), isoform CRA_d [Ho	2	21	metabolic process	1.013	0.743	0.735
1500431	sodium-dependent neutral amino acid transporter type 2 truncated	8	49	transport	1.091	0.743	0.725
1196115	actin related protein 2/3 complex, subunit 5, 16kDa, isoform CRA_	2	6	nd biogenesis; defense response	0.832	0.743	0.715
6650104	bicarbonate transporter [Homo sapiens]	6	13	homeostasis; cell differentiation	0.968	0.742	0.737
1195957	ribosomal protein S21, isoform CRA_b [Homo sapiens]	3	22	ization and biogenesis; transport	1.696	0.741	0.742
6144582	phosphoenolpyruvate carboxykinase [GTP], mitochondrial isoform	25	164	metabolic process	0.968	0.741	0.716
1890655	unnamed protein product [Homo sapiens]	15	98	ic process; response to stimulus	1.015	0.741	0.762
2590135	thymidylate kinase isoform 2 [Homo sapiens]	6	36	se to stimulus; cell differentiation	1.204	0.740	0.787
1834483	Chain B, Crystal Structure Of Human Eukaryotic Translation Initiat	3	10	; regulation of biological process	0.716	0.740	0.727
7680034	PREDICTED: small glutamine-rich tetratricopeptide repeat-contain	2	3	regulation of biological process	0.795	0.739	0.805
4793958	RTN4 protein [Homo sapiens]	7	49	cell organization and biogenesis	0.938	0.739	0.838
5158693	hsc70-interacting protein isoform 2 [Homo sapiens]	5	24	; regulation of biological process	1.023	0.737	0.818
7679550	PREDICTED: DENN domain-containing protein 4C isoform X4 [Hom	2	3	cell organization and biogenesis	0.876	0.737	0.772
1195920	spermidine synthase, isoform CRA_a [Homo sapiens]	4	17	metabolic process	0.957	0.736	0.792
1195868	nucleoside phosphorylase, isoform CRA_a [Homo sapiens]	10	45	n of biological process; transport	1.059	0.736	0.762
1196279	retinoblastoma binding protein 4, isoform CRA_b [Homo sapiens]	8	36	; regulation of biological process	0.979	0.736	0.811
1582608	unnamed protein product [Homo sapiens]	2	12	; regulation of biological process	0.964	0.736	0.784
1582573	unnamed protein product [Homo sapiens]	4	10	e to stimulus; metabolic process	0.819	0.735	0.737
1196052	hCG38577, partial [Homo sapiens]	2	6		1.562	0.735	0.899
6750824	Chain A, Crystal Structure Of Extracellular Part Of Human Epcam	3	5		1.047	0.735	0.885
7679410	PREDICTED: BAG family molecular chaperone regulator 2 isoform	2	6	; regulation of biological process	1.138	0.735	0.700

1772974	endonuclease III homologue 1, partial [Homo sapiens]	4	6	ic process; response to stimulus	0.982	0.734	0.785
7679658	PREDICTED: rho GTPase-activating protein 1 isoform X2 [Homo sa	8	40	al process; response to stimulus	1.029	0.732	0.683
7679643	PREDICTED: STE20-like serine/threonine-protein kinase isoform X	3	7	ic process; response to stimulus	0.831	0.731	0.835
1943855	unnamed protein product [Homo sapiens]	12	40	cell organization and biogenesis	1.026	0.731	0.760
1196009	MYC binding protein 2, isoform CRA_c [Homo sapiens]	15	25	cell organization and biogenesis	0.906	0.729	0.732
7280557	Chain C, Crystal Structure Of The T229k Mutant Of Human Gapdh /	16	606		1.033	0.729	0.769
1937858	unnamed protein product [Homo sapiens]	5	7	transport	1.038	0.729	0.731
7679812	PREDICTED: RNA-binding protein 25 isoform X2 [Homo sapiens]	3	18	gical process; metabolic process	1.107	0.729	0.829
7018510	hypothetical protein [Homo sapiens]	3	8	transport; development	1.086	0.728	0.755
8890050	protein arginine N-methyltransferase 5 isoform b [Homo sapiens]	9	35	logical process; cell proliferation	0.925	0.728	0.756
2032823	Chain D, Crystal Structure Of Human Enolase 1	15	300	metabolic process	1.062	0.728	0.800
1591627	Chain A, Solution Structure Of The Ppiase Domain Of Human Pin1	2	5		0.658	0.727	0.697
1543131	keratin, type I cytoskeletal 14 [Homo sapiens]	22	227	cell organization and biogenesis	0.953	0.727	0.696
1195994	debranching enzyme homolog 1 (S. cerevisiae) [Homo sapiens]	2	10	metabolic process	0.873	0.727	0.787
6655059	sorting nexin-3 isoform c [Homo sapiens]	2	8	ss; transport; metabolic process	1.265	0.727	0.860
7679401	PREDICTED: serpin B6 isoform X3 [Homo sapiens]	5	12	al process; response to stimulus	0.803	0.727	0.755
1105912	Chain A, Extended Substrate Recognition In Caspase-3 Revealed E	3	14	metabolic process	1.128	0.727	0.743
2210396	unnamed protein product [Homo sapiens]	14	123	cell organization and biogenesis	0.947	0.727	0.725
340062	pro-ubiquitin, partial [Homo sapiens]	5	446	ent movement; defense response	1.006	0.726	0.736
4782472	Chain B, Lys6-linked Tri-ubiquitin	5	446		1.005	0.726	0.736
5304129	PREDICTED: A-kinase anchor protein 1, mitochondrial isoform X1	3	4	al process; response to stimulus	0.893	0.726	0.772
6619184	Chain A, Crystal Structure Of Human Farnesyl Diphosphoate Synth	9	42		1.110	0.726	0.793
4298368	signal peptidase complex catalytic subunit SEC11A isoform 5 [Hor	3	17	; regulation of biological process	1.082	0.725	0.782
3355455	R33729_1, partial [Homo sapiens]	5	42	e to stimulus; metabolic process	1.147	0.725	0.727
1195751	N-terminal asparagine amidase [Homo sapiens]	3	6	e to stimulus; metabolic process	0.811	0.725	0.722
6094125	Chain B, Crystal Structure Of Transportin-sr2, A Karyopherin Invol	6	21		0.894	0.724	0.793
7679576	PREDICTED: phosducin-like protein isoform X1 [Homo sapiens]	3	8	cell organization and biogenesis	0.988	0.724	0.758
4004101	unnamed protein product, partial [Homo sapiens]	2	4		1.105	0.723	0.738
1630706	SUB1 homolog (S. cerevisiae) [Homo sapiens]	3	23	ic process; response to stimulus	1.287	0.723	0.783
1205386	TBC1 domain family, member 9B (with GRAM domain) [Homo sapi	4	7	regulation of biological process	0.812	0.723	0.696
3128368	adenylate kinase 2, mitochondrial isoform c [Homo sapiens]	6	31	metabolic process	1.004	0.723	0.742
1195801	phosphatidylinositol transfer protein, beta, isoform CRA_c [Homo	2	10	metabolic process; transport	1.172	0.723	0.684
1043513	unnamed protein product [Homo sapiens]	3	5	; regulation of biological process	1.122	0.722	0.633
7679627	PREDICTED: NHL repeat-containing protein 2 isoform X1 [Homo sa	3	13		0.885	0.722	0.856
5304057	PREDICTED: isocitrate dehydrogenase [NAD] subunit alpha, mitoc	5	26	metabolic process	1.311	0.722	0.764
1890532	unnamed protein product [Homo sapiens]	2	13	cell organization and biogenesis	1.001	0.721	0.767
433938	ERGIC53 [Homo sapiens]	7	46	; regulation of biological process	1.059	0.721	0.743
2065179	unnamed protein product, partial [Homo sapiens]	3	18	; regulation of biological process	1.574	0.721	0.868
5788010	PREDICTED: gamma-secretase subunit APH-1A isoform X1 [Homo	2	3	s; cellular component movement	1.254	0.721	0.767
230868	Chain G, Twinning In Crystals Of Human Skeletal Muscle D-Glycera	6	171	metabolic process	1.033	0.721	0.667

1943769	unnamed protein product [Homo sapiens]	5	16	sis; transport; metabolic process	1.173	0.720	0.717
4503483	elongation factor 2 [Homo sapiens]	27	258	; regulation of biological process	1.095	0.720	0.791
1582598	unnamed protein product, partial [Homo sapiens]	7	23	metabolic process	1.071	0.720	0.760
5303921	PREDICTED: 6-phosphofructo-2-kinase/fructose-2,6-bisphosphata	5	13	metabolic process	1.212	0.720	0.740
1195812	Coenzyme A synthase, isoform CRA_b [Homo sapiens]	6	60	metabolic process	1.038	0.719	0.727
1943832	unnamed protein product [Homo sapiens]	4	25	cell organization and biogenesis	1.105	0.719	0.750
1403584	unnamed protein product [Homo sapiens]	2	2		0.887	0.719	0.728
1196180	ADP-ribosylation factor-like 1, isoform CRA_c [Homo sapiens]	5	10	us; metabolic process; transport	1.009	0.718	0.731
3398838	PTGES2 protein, partial [Homo sapiens]	4	26	n of biological process; transport	1.088	0.718	0.815
4004134	unnamed protein product, partial [Homo sapiens]	8	36		0.912	0.717	0.770
3860240	PHP [Homo sapiens]	7	53	metabolic process	0.971	0.717	0.815
2210440	unnamed protein product [Homo sapiens]	7	15	metabolic process	1.145	0.717	0.779
1196262	E-1 enzyme, isoform CRA_c, partial [Homo sapiens]	2	8	metabolic process	1.143	0.716	0.792
1890532	unnamed protein product [Homo sapiens]	3	6	metabolic process; transport	0.910	0.716	0.662
1195702	COX15 homolog, cytochrome c oxidase assembly protein (yeast), i	4	8	ization and biogenesis; transport	0.875	0.715	0.700
1195715	hCG1998851, isoform CRA_b [Homo sapiens]	2	3	metabolic process	1.157	0.714	0.730
6782054	unnamed protein product [Homo sapiens]	3	18		1.116	0.714	0.692
1103585	laminin beta 2 chain [Homo sapiens]	4	7	t; cell differentiation; cell growth	0.771	0.714	0.651
1196170	cytokeratin type II [Homo sapiens]	14	171	cell differentiation	0.955	0.713	0.857
8350203	Chain A, Pi3k Alpha Lipid Kinase With Active Site Inhibitor	3	11		1.130	0.712	0.735
1196132	recombining binding protein suppressor of hairless (Drosophila), i	2	3	ess; cell differentiation; transport	0.947	0.712	0.761
7680450	PREDICTED: pyridoxal-dependent decarboxylase domain-containi	8	43	metabolic process	1.063	0.712	0.703
5655419	Chain A, The Structure Of Human Erythrocyte Nadh-Cytochrome B	7	51	metabolic process	0.960	0.711	0.740
2385379	Chain B, Human Ubiquitin-activating Enzyme 5 In Complex With At	3	7		0.854	0.711	0.772
3399611	Chain A, Solution Structure Of The N-Terminal Domain Of Human A	3	12	metabolic process	1.069	0.710	0.770
1943855	unnamed protein product [Homo sapiens]	3	14	al process; cellular homeostasis	1.102	0.710	0.750
1195931	family with sequence similarity 50, member A, isoform CRA_b [Hor	2	3		0.827	0.709	0.675
4557976	Chain A, Human Muscle Fructose 1,6-Bisphosphate Aldolase Com	21	318	metabolic process	1.175	0.708	0.757
3282243	alpha enolase like 1, partial [Homo sapiens]	9	162	metabolic process	1.025	0.707	0.765
5435837	prostaglandin E synthase 3 isoform f [Homo sapiens]	4	18	al process; response to stimulus	1.020	0.707	0.829
5770194	glucose-6-phosphate isomerase isoform 4 [Homo sapiens]	21	239	; regulation of biological process	0.991	0.707	0.685
4782472	Chain B, Crystal Structure Of Oxidized Hpdi (abb'xa')	21	352		1.161	0.706	0.772
5788356	PREDICTED: phosphatidate cytidylyltransferase 2 isoform X1 [Hon	2	4	metabolic process	0.966	0.706	0.705
1890678	unnamed protein product [Homo sapiens]	9	44	al process; response to stimulus	1.082	0.706	0.708
1195864	transmembrane 9 superfamily member 1, isoform CRA_d [Homo sa	8	32	metabolic process	1.009	0.706	0.707
181573	cytokeratin 8 [Homo sapiens]	35	824	sis; cell differentiation; cell death	1.108	0.705	0.727
5138916	PTD012 [Homo sapiens]	2	3	metabolic process	0.943	0.705	0.523
1943778	unnamed protein product [Homo sapiens]	4	11	cell organization and biogenesis	1.387	0.705	0.657
1196281	DnaJ (Hsp40) homolog, subfamily C, member 8, isoform CRA_a [H	2	3	metabolic process	0.703	0.705	0.782
1943842	unnamed protein product [Homo sapiens]	7	24	; regulation of biological process	1.348	0.704	0.661

6110491	heat shock protein 90Bf [Homo sapiens]	2	9	ic process; response to stimulus	1.466	0.703	0.805
7679941	PREDICTED: WW domain-binding protein 2 isoform X1 [Homo sap	2	11	al process; response to stimulus	1.062	0.703	0.586
8957412	mitochondrial malate dehydrogenase 2, NAD, partial [Homo sapier	14	183	metabolic process	0.918	0.702	0.734
7767140	Chain H, Crystal Structure Of Human Ubiquitous Mitochondrial Cre	8	109		0.907	0.702	0.688
1592860	Solute carrier family 25 (mitochondrial carrier; adenine nucleotide	13	220	al process; response to stimulus	0.916	0.702	0.714
1196102	solute carrier family 25 (mitochondrial carrier; adenine nucleotide	15	284	; regulation of biological process	0.923	0.701	0.706
1012102	Chain B, Fk506 Binding Protein Mutant, Homodimeric Complex	2	63	metabolic process	0.919	0.700	0.757
1515679	Chain L, Structure Of Human Udp-glucose Dehydrogenase Comple	12	140	metabolic process	1.229	0.699	0.737
307132	membrane glycoprotein [Homo sapiens]	9	47	to stimulus; cell death; transport	1.045	0.698	0.698
2279631	3-oxoacyl-CoA thiolase, partial [Homo sapiens]	4	13	metabolic process	1.001	0.698	0.762
7680096	PREDICTED: glutaminyl-peptide cyclotransferase-like protein isofo	4	20	metabolic process	0.837	0.698	0.693
1196277	trafficking protein particle complex 3, isoform CRA_a [Homo sapie	3	12	cell organization and biogenesis	1.019	0.698	0.745
1196317	hCG1783452, isoform CRA_a [Homo sapiens]	3	23		1.522	0.697	0.769
1582572	unnamed protein product [Homo sapiens]	14	168	; regulation of biological process	0.906	0.697	0.713
6208908	itchy homolog E3 ubiquitin protein ligase variant, partial [Homo sa	5	11	response; response to stimulus	0.909	0.697	0.707
5303732	PREDICTED: protein RFT1 homolog isoform X4 [Homo sapiens]	6	10	cell organization and biogenesis	0.994	0.696	0.745
2623678	Chain A, Catalytic Domain Of Uch37	4	17	metabolic process	1.212	0.696	0.754
2703465	Chain B, Crystal Structure Of Human Insulin-Degrading Enzyme In	10	17	metabolic process	1.045	0.696	0.813
1943863	unnamed protein product [Homo sapiens]	4	11	metabolic process; development	1.207	0.695	0.674
1195930	von Hippel-Lindau binding protein 1, isoform CRA_a [Homo sapier	2	17	cell organization and biogenesis	0.876	0.695	0.669
1196095	solute carrier family 9 (sodium/hydrogen exchanger), member 3 re	6	61	homeostasis; cell differentiation	1.018	0.694	0.744
1043908	unnamed protein product [Homo sapiens]	3	4	; regulation of biological process	0.903	0.694	0.759
2809488	cytochrome c oxidase subunit IV, partial [Homo sapiens]	3	11		1.139	0.694	0.669
1196015	zinc binding alcohol dehydrogenase, domain containing 1, isoform	4	12	metabolic process	1.037	0.693	0.681
2175458	unnamed protein product [Homo sapiens]	13	124	cell organization and biogenesis	1.068	0.692	0.681
1890549	unnamed protein product [Homo sapiens]	3	19	biogenesis; response to stimulus	1.063	0.691	0.714
1591626	Chain A, Human Protein Disulfide Isomerase, Nmr, 40 Structures	6	76	; regulation of biological process	1.456	0.691	0.775
6208891	Insulin-like growth factor 2 receptor variant, partial [Homo sapiens	8	17	al process; response to stimulus	0.884	0.691	0.696
1455805	Chain B, Crystal Structure Of Human 3-Hydroxy-3-Methylglutaryl C	8	29	metabolic process	1.221	0.691	0.695
1943815	unnamed protein product [Homo sapiens]	2	4	transport	1.107	0.690	0.714
4814572	RER1 [Homo sapiens]	3	5	; regulation of biological process	0.803	0.688	0.688
1943841	unnamed protein product [Homo sapiens]	4	12	; regulation of biological process	0.816	0.688	0.734
1641838	vacuolar protein sorting-associated protein 26B [Homo sapiens]	4	10	transport	0.977	0.688	0.642
7679359	PREDICTED: alpha-mannosidase 2 isoform X1 [Homo sapiens]	7	18	; regulation of biological process	1.231	0.687	0.691
3169010	putative GTP-binding protein, partial [Homo sapiens]	2	3	; regulation of biological process	1.322	0.687	0.755
4282289	migration and invasion enhancer 1 [Homo sapiens]	3	13	al process; cellular homeostasis	1.118	0.686	0.872
2190371	carboxypeptidase D [Homo sapiens]	13	40	metabolic process	0.945	0.686	0.756
1195911	ATP-binding cassette, sub-family B (MDR/TAP), member 6, isoform	10	31	; transport; cellular homeostasis	0.958	0.685	0.678
3387563	ANP32A protein, partial [Homo sapiens]	3	43	; transport; response to stimulus	1.128	0.685	0.832
3718238	COG7 [Homo sapiens]	2	11	; regulation of biological process	0.976	0.683	0.710

1943871	unnamed protein product [Homo sapiens]	3	4	cell organization and biogenesis	0.877	0.683	0.687
1195988	mitofusin 1, isoform CRA_d [Homo sapiens]	2	3	cell organization and biogenesis	1.065	0.683	0.721
1890665	unnamed protein product [Homo sapiens]	8	87	s; cellular component movement	1.208	0.682	0.722
5430402	glyceraldehyde-3-phosphate dehydrogenase, partial [Homo sapien	5	169	cell death; response to stimulus	0.869	0.682	0.700
2148297	ADP-ribosylation factor GTPase-activating protein 3 isoform 2 [Hor	4	11	; regulation of biological process	1.006	0.681	0.662
1196281	hCG18930 [Homo sapiens]	4	14	metabolic process	1.152	0.681	0.708
2807107	unnamed protein product [Homo sapiens]	4	44	gical process; defense response	0.762	0.681	0.647
1004725	KIAA1592 protein, partial [Homo sapiens]	2	4	transport; response to stimulus	1.035	0.681	0.742
7679666	PREDICTED: phosphatidylserine synthase 2 isoform X2 [Homo sap	3	4	metabolic process	0.880	0.679	0.699
182979	glyceraldehyde 3-phosphate dehydrogenase, partial [Homo sapien	2	57	cell death; response to stimulus	1.161	0.679	0.485
3044456	Chain A, Complex Of Human Dihydroorotate Dehydrogenase With	4	8	metabolic process	1.051	0.678	0.634
6289768	calreticulin precursor variant, partial [Homo sapiens]	10	116	e to stimulus; cell differentiation	1.183	0.678	0.762
7679684	PREDICTED: RING finger protein 121 isoform X2 [Homo sapiens]	2	3	al process; response to stimulus	0.852	0.677	0.733
1943872	unnamed protein product [Homo sapiens]	2	6	; response to stimulus; transport	1.095	0.677	0.724
7679570	PREDICTED: osteoclast-stimulating factor 1 isoform X3 [Homo sap	2	5	al process; response to stimulus	0.849	0.677	0.758
3255339	Chain A, Crystal Structure Of The Globular Domain Of Human Calr	10	109		1.145	0.677	0.754
1138121	MYL6 protein, partial [Homo sapiens]	5	30	s; cellular component movement	1.275	0.677	0.664
1196059	dodecenoyl-Coenzyme A delta isomerase (3,2 trans-enoyl-Coenzyr	5	36	metabolic process	0.991	0.677	0.756
1895823	zinc finger protein [Homo sapiens]	2	2	ss; metabolic process; cell death	0.933	0.676	0.659
5788325	PREDICTED: dymeclin isoform X11 [Homo sapiens]	2	7	cell organization and biogenesis	0.780	0.676	0.670
7679700	PREDICTED: m7GpppX diphosphatase isoform X2 [Homo sapiens]	4	16	; regulation of biological process	1.151	0.675	0.688
1582549	unnamed protein product [Homo sapiens]	2	4	metabolic process	0.731	0.675	0.766
1827593	Chain B, Recombinant Cyclophilin A From Human T Cell	7	99	metabolic process	1.096	0.672	0.657
1196150	leucine rich repeat containing 59, isoform CRA_a [Homo sapiens]	2	10		0.690	0.671	0.711
2159554	Lectin, mannose-binding, 1 [Homo sapiens]	7	49	; regulation of biological process	1.045	0.670	0.715
1890536	unnamed protein product [Homo sapiens]	5	15	transport; cell communication	1.222	0.669	0.672
1195727	KDEL (Lys-Asp-Glu-Leu) endoplasmic reticulum protein retention	2	18	transport	0.914	0.668	0.742
5304002	PREDICTED: tRNA (guanine-N(7)-)-methyltransferase isoform X1 [H	2	6	metabolic process	0.959	0.668	0.657
5739521	TAP2 protein, partial [Homo sapiens]	2	3	sis; transport; defense response	1.084	0.667	0.634
3886038	Chain A, Oxidoreductase Fragment Of Human Qsox1	3	5		0.964	0.666	0.612
1890655	unnamed protein product [Homo sapiens]	2	6	; regulation of biological process	1.824	0.665	0.801
7138809	hepatopoietin PCn127 [Homo sapiens]	2	20		1.074	0.665	0.699
5303766	PREDICTED: transmembrane protein 33 isoform X1 [Homo sapiens	5	28	cell organization and biogenesis	0.947	0.663	0.664
5744514	Chain D, The Structure Of Cc1-ih In Human Stim1.	2	5		1.104	0.663	0.669
8465929	unnamed protein product, partial [Homo sapiens]	10	35		1.315	0.662	0.688
1890532	unnamed protein product [Homo sapiens]	2	5	metabolic process; transport	1.074	0.662	0.719
3152378	DnaJ protein, partial [Homo sapiens]	3	6	al process; response to stimulus	0.918	0.662	0.629
6282208	unknown [Homo sapiens]	4	13	cell death	0.889	0.662	0.725
7491978	Chain F, Crystal Structure Of Bag6-ubl4a Dimerization Domain	2	6		1.090	0.661	0.728
1196168	golgi transport 1 homolog B (S. cerevisiae), isoform CRA_a [Homo	2	16	; response to stimulus; transport	0.800	0.661	0.786

5788226	PREDICTED: tyrosine-protein phosphatase non-receptor type 6 iso	3	7	n; cellular component movement	1.051	0.661	0.721
1195885	GPI-anchored membrane protein 1, isoform CRA_c [Homo sapiens	5	10	regulation of biological process	1.201	0.661	0.758
5254581	hypothetical protein, partial [Homo sapiens]	3	8	al process; response to stimulus	1.042	0.660	0.748
7679018	PREDICTED: CAAX prenyl protease 1 homolog isoform X1 [Homo s	8	24	cell organization and biogenesis	1.062	0.660	0.677
2210459	unnamed protein product [Homo sapiens]	2	4	d biogenesis; metabolic process	0.780	0.660	0.656
1937875	unnamed protein product [Homo sapiens]	5	8	metabolic process; transport	1.001	0.659	0.639
3116413	unnamed protein product [Homo sapiens]	6	27		1.035	0.658	0.768
2977875	Chain A, Solution Structure Of The Leucine Rich Repeat Of Human	2	22		1.212	0.657	0.809
3753724	Acetoacetyl-CoA synthetase [Homo sapiens]	5	21	erentiation; cellular homeostasis	0.898	0.656	0.637
6624942	hypothetical protein, partial [Homo sapiens]	5	29	; regulation of biological process	1.139	0.656	0.735
31746	glutathione-insulin transhydrogenase (216 AA), partial [Homo sapi	8	52	; regulation of biological process	1.562	0.656	0.705
1937854	unnamed protein product [Homo sapiens]	2	6	metabolic process	1.012	0.656	0.696
5094925	hHa8 protein, partial [Homo sapiens]	2	18		0.858	0.656	0.655
5754033	Chain A, Crystal Structure Of G Protein-coupled Receptor Kinase 2	5	15		1.018	0.655	0.645
7679347	PREDICTED: pachytene checkpoint protein 2 homolog isoform X1	5	25	; regulation of biological process	1.054	0.655	0.718
4506975	solute carrier family 12 member 2 isoform 1 [Homo sapiens]	5	15	; regulation of biological process	0.986	0.654	0.591
2098705	Chain B, Crystal Structure Of The Mammalian Copii-coat Protein S	10	43	transport	1.067	0.653	0.672
1196186	KIAA0152, isoform CRA_c [Homo sapiens]	6	43	metabolic process	1.148	0.653	0.675
1091578	Chain H, X-Ray Crystal Structure Of Ngt-Bound Hexa	5	15	metabolic process	0.946	0.653	0.628
1196270	tumor-associated calcium signal transducer 2 [Homo sapiens]	2	11	nse to stimulus; cell proliferation	1.366	0.651	0.676
5304048	PREDICTED: AP-1 complex subunit gamma-like 2 isoform X6 [Hom	7	18	cell organization and biogenesis	0.919	0.651	0.693
1196265	mannosidase, beta A, lysosomal [Homo sapiens]	5	13	metabolic process	0.861	0.651	0.670
1943787	unnamed protein product [Homo sapiens]	11	32	ical process; cell communication	1.138	0.650	0.666
3382214	p180/ribosome receptor [Homo sapiens]	15	92	ss; metabolic process; transport	0.947	0.650	0.639
7679361	PREDICTED: phosphatidylinositol 3-kinase regulatory subunit alph	4	15	component movement; transport	1.130	0.649	0.693
7679293	PREDICTED: peroxisomal acyl-coenzyme A oxidase 3 isoform X4 [	4	9	metabolic process	1.093	0.648	0.674
1195947	synovial apoptosis inhibitor 1, synoviolin, isoform CRA_b [Homo s	2	11	; response to stimulus; transport	0.849	0.646	0.626
5147644	hypothetical protein [Homo sapiens]	2	3	; cell division; defense response	1.058	0.645	0.784
1195834	neural precursor cell expressed, developmentally down-regulated	5	24	meostasis; response to stimulus	0.938	0.644	0.644
1091578	Chain B, Crystal Structure Of Human Sco1 Complexed With Nickel	3	17	metabolic process	1.076	0.642	0.815
1196060	hydroxyacylglutathione hydrolase, isoform CRA_a [Homo sapiens]	2	5	metabolic process	1.015	0.642	0.718
2210395	unnamed protein product [Homo sapiens]	8	34	al process; response to stimulus	0.976	0.641	0.599
7679638	PREDICTED: mini-chromosome maintenance complex-binding pro	6	16	tion and biogenesis; cell division	0.951	0.638	0.585
4022560	RAB13 protein, partial [Homo sapiens]	5	38	s; cellular component movement	1.063	0.635	0.589
7353623	Chain A, The 1.58a Crystal Structure Of Human Coproporphyrinog	4	14	metabolic process	1.369	0.635	0.734
1578362	Chain A, Crystal Structure Of Human Sdr Family Member 1	3	7	metabolic process	0.886	0.635	0.640
1890535	unnamed protein product [Homo sapiens]	9	85	transport	1.106	0.633	0.696
1196172	dermcidin [Homo sapiens]	2	4	e response; response to stimulus	0.728	0.633	1.150
30311	cytokeratin 18 (424 AA), partial [Homo sapiens]	24	680	biogenesis; transport; cell death	0.939	0.632	0.612
1195838	kinesin family member 13B, isoform CRA_a [Homo sapiens]	13	29	e to stimulus; metabolic process	1.137	0.632	0.694

2813069	Chain B, Crystal Structure Of The Human Mgc45594 Gene Product	3	7	metabolic process	1.049	0.629	0.673
7679315	PREDICTED: cystine/glutamate transporter isoform X6 [Homo sapi	2	3	ent movement; cell differentiation	0.972	0.628	0.671
7679776	PREDICTED: high mobility group protein B1 isoform X2 [Homo sap	3	4	; cell death; cellular homeostasis	1.323	0.627	0.802
4004194	unnamed protein product, partial [Homo sapiens]	3	15		0.936	0.627	0.702
5979601	Chain B, Crystal Structure Of Se-met Hn33/tusc3	3	12		1.001	0.627	0.683
2014766	ADP-ribosylation factor-like protein 2 [Homo sapiens]	2	15	al process; response to stimulus	1.461	0.626	0.685
1195680	acetyl-Coenzyme A acetyltransferase 2 (acetoacetyl Coenzyme A th	5	22	metabolic process	1.031	0.625	0.670
1890531	unnamed protein product [Homo sapiens]	2	3	cell organization and biogenesis	0.750	0.625	0.687
7576577	Chain B, Crystal Structure Of The Cyclophiln_ring Domain Of Hum	2	4	metabolic process	0.846	0.624	0.600
7280553	Chain B, Crystal Structure Of Human Insulin Regulated Aminopept	2	4		0.866	0.624	0.621
5253426	acyl-protein thioesterase 1 isoform 6 [Homo sapiens]	5	32	; regulation of biological process	0.851	0.621	0.673
1195691	solute carrier family 17 (anion/sugar transporter), member 5, isofor	2	5	metabolic process; transport	0.912	0.620	0.687
7679583	PREDICTED: actin-related protein 2/3 complex subunit 5-like prote	3	7	; regulation of biological process	0.824	0.619	0.615
1890553	unnamed protein product [Homo sapiens]	33	644	nd biogenesis; cell differentiation	1.028	0.619	0.609
5304105	PREDICTED: nucleoredoxin isoform X3 [Homo sapiens]	4	12	lifferentiation; metabolic process	0.735	0.616	0.651
913349	squalene synthase [Homo sapiens]	8	47	metabolic process	1.130	0.616	0.617
5559439	heme oxygenase 2 isoform c [Homo sapiens]	4	9	; cellular homeostasis; transport	0.770	0.615	0.706
1943861	unnamed protein product [Homo sapiens]	7	33	nunication; response to stimulus	1.109	0.615	0.629
307066	inosine-5'-monophosphate dehydrogenase (EC 1.1.1.205) [Homo s	8	47	biogenesis; response to stimulus	0.948	0.613	0.619
1196299	WD repeat domain 4, isoform CRA_a [Homo sapiens]	2	5	metabolic process	1.044	0.613	0.608
1195682	fucosidase, alpha-L- 2, plasma [Homo sapiens]	3	7	; regulation of biological process	0.861	0.612	0.653
3757226	RNA (guanine-9-) methyltransferase domain containing 1 [Homo sa	2	3	d biogenesis; metabolic process	1.405	0.612	0.738
9187600	hypothetical protein, similar to (AAF46469.1) CG12121 predicted pr	4	8		0.970	0.612	0.684
2959876	synaptogyrin 2, partial [Homo sapiens]	4	16		1.000	0.612	0.633
7679923	PREDICTED: phosphatidylinositol transfer protein alpha isoform is	2	7	rt; cellular component movement	1.244	0.612	0.719
5121254	Chain B, Protease-induced Heterodimer Of Human Triosephospha	14	120		1.140	0.612	0.625
7535342	Chain B, Crystal Structure Of A Single Mutant (n71d) Of Triosepho	14	120		1.140	0.612	0.628
5303848	PREDICTED: MAGUK p55 subfamily member 6 isoform X2 [Homo s	3	7	cell organization and biogenesis	0.873	0.612	0.685
1195860	farnesyl-diphosphate farnesyltransferase 1, isoform CRA_a [Homo	8	49	metabolic process	1.130	0.609	0.619
1582556	unnamed protein product [Homo sapiens]	9	84	erentiation; cellular homeostasis	1.014	0.609	0.603
1195686	5'-nucleotidase domain containing 1, isoform CRA_a [Homo sapie	2	4	metabolic process	1.076	0.608	0.599
7679088	PREDICTED: nicastrin isoform X2 [Homo sapiens]	11	57	and biogenesis; cell proliferation	0.978	0.608	0.624
1943840	unnamed protein product [Homo sapiens]	6	13	cell organization and biogenesis	1.031	0.604	0.607
1739149	HAT1 protein [Homo sapiens]	3	5	al process; response to stimulus	1.244	0.603	0.648
1578304	Chain A, Structure Of M2bp Scavenger Receptor Cysteine-rich Dor	2	4		1.041	0.603	0.588
1508041	PNPLA2 protein, partial [Homo sapiens]	2	5	cell organization and biogenesis	1.107	0.602	0.550
7679941	PREDICTED: fructose-bisphosphate aldolase C isoform X1 [Homo	16	249	cell organization and biogenesis	1.079	0.602	0.611
7679906	PREDICTED: large neutral amino acids transporter small subunit 1	2	6	n; cellular component movement	0.799	0.600	0.567
1196094	nucleolar protein 11, isoform CRA_b [Homo sapiens]	3	6	; regulation of biological process	1.112	0.600	0.673
1937836	unnamed protein product [Homo sapiens]	3	6	metabolic process	1.001	0.600	0.616

1943760	unnamed protein product [Homo sapiens]	8	17	cell organization and biogenesis	0.964	0.599	0.630
2996578	glucosyltransferase, partial [Homo sapiens]	2	4	metabolic process	1.056	0.599	0.629
2819320	unnamed protein product, partial [Homo sapiens]	3	15	metabolic process	1.147	0.597	0.751
5303606	PREDICTED: solute carrier family 25 member 33 isoform X2 [Homo	2	3	; transport; response to stimulus	0.829	0.596	0.608
6639220	NME1-NME2 protein [Homo sapiens]	12	98	metabolic process	1.021	0.595	0.594
2210459	unnamed protein product [Homo sapiens]	2	6	n; cellular component movement	0.871	0.595	0.582
1582594	unnamed protein product [Homo sapiens]	4	25	s; cellular component movement	1.237	0.594	0.877
1492424	Chain A, Structure Of The Calcineurin-Nfat Complex	6	40		1.243	0.594	0.632
1578342	Chain A, Human Glutathione Reductase In Complex With A Xanthe	13	164	gical process; metabolic process	1.154	0.594	0.577
1890530	unnamed protein product [Homo sapiens]	2	10	metabolic process; transport	1.225	0.593	0.723
1943793	unnamed protein product [Homo sapiens]	7	29	; regulation of biological process	1.049	0.592	0.605
1196199	all-trans-13,14-dihydroretinol saturase, isoform CRA_c [Homo sapi	7	20	metabolic process	1.029	0.589	0.604
1943910	unnamed protein product [Homo sapiens]	4	16	metabolic process; reproduction	1.091	0.589	0.661
7679631	PREDICTED: serine/threonine-protein phosphatase 2B catalytic su	4	22	n; defense response; cell growth	1.252	0.585	0.620
1196306	hCG22457, isoform CRA_b [Homo sapiens]	2	5	regulation of biological process	1.009	0.584	0.600
1943857	unnamed protein product [Homo sapiens]	5	15	; regulation of biological process	1.189	0.584	0.615
1943906	unnamed protein product [Homo sapiens]	6	24	metabolic process	0.938	0.584	0.542
2173027	Chain B, D92n,D94n Double Point Mutant Of Human Nuclear Trans	2	5	transport	1.461	0.584	0.824
2210447	unnamed protein product [Homo sapiens]	3	6	; regulation of biological process	1.036	0.583	0.533
1196099	calcium activated nucleotidase 1, isoform CRA_a [Homo sapiens]	3	5	e to stimulus; metabolic process	1.034	0.582	0.631
9403766	Chain G, The Crystal Structure Of The Gst-like Domains Complex C	3	10		1.211	0.581	0.653
4147446	unknown [Homo sapiens]	3	26	; regulation of biological process	1.247	0.580	0.652
1195700	ADP-ribosylation factor-like 3, partial [Homo sapiens]	4	12	s; cellular component movement	0.850	0.578	0.658
7679327	PREDICTED: calcium/calmodulin-dependent protein kinase type II	2	3	ation and biogenesis; cell growth	0.885	0.577	0.571
1890533	unnamed protein product [Homo sapiens]	4	11	al process; response to stimulus	1.118	0.575	0.590
1195815	basigin (Ok blood group), isoform CRA_e [Homo sapiens]	5	29	component movement; transport	1.021	0.575	0.621
1195793	thioredoxin domain containing 4 (endoplasmic reticulum), isoform	5	35	; regulation of biological process	1.097	0.572	0.639
1196283	argininosuccinate lyase, isoform CRA_b [Homo sapiens]	6	29	cell organization and biogenesis	1.007	0.572	0.530
1890674	unnamed protein product [Homo sapiens]	2	2	; regulation of biological process	0.979	0.572	0.576
3964464	DHRS2 protein, partial [Homo sapiens]	14	277	se to stimulus; cell differentiation	0.974	0.567	0.534
2210408	unnamed protein product [Homo sapiens]	3	9	metabolic process	0.843	0.564	0.541
6270222	unknown [Homo sapiens]	7	41	al process; response to stimulus	0.912	0.561	0.513
1330302	Mn-superoxiddismutase [Homo sapiens]	3	5		1.160	0.558	0.718
7353595	Chain A, C Domain Of Human Cyclophilin-33(Hcyp33)	2	2	metabolic process	1.032	0.556	0.491
1196101	dicarbonyl/L-xylulose reductase, isoform CRA_a [Homo sapiens]	9	120	cell organization and biogenesis	0.869	0.555	0.561
3006507	unnamed protein product [Homo sapiens]	2	6		1.043	0.540	0.652
1592891	Unknown (protein for IMAGE:3906970), partial [Homo sapiens]	2	7	e to stimulus; metabolic process	1.107	0.538	0.516
4071588	placental protein 23 [Homo sapiens]	6	26	cell organization and biogenesis	1.141	0.538	0.552
1195816	phosphatidic acid phosphatase type 2C, isoform CRA_c [Homo sa	4	24	al process; response to stimulus	1.027	0.537	0.550
5254565	hypothetical protein, partial [Homo sapiens]	2	3	regulation of biological process	0.929	0.536	0.498

1195737	S100 calcium binding protein A11 (calgizzarin) [Homo sapiens]	3	38	al process; response to stimulus	0.955	0.535	0.560
5303661	PREDICTED: lamin-B receptor isoform X3 [Homo sapiens]	5	35	metabolic process	1.069	0.532	0.496
5303726	PREDICTED: 6-phosphofructo-2-kinase/fructose-2,6-bisphosphata	3	10	metabolic process	1.219	0.531	0.588
7680136	PREDICTED: monoacylglycerol lipase ABHD12 isoform X2 [Homo s	6	25	bagulation; response to stimulus	1.029	0.531	0.568
2173970	hypothetical protein, partial [Homo sapiens]	6	15	; regulation of biological process	1.121	0.530	0.482
7679431	PREDICTED: probable lysosomal cobalamin transporter isoform X	2	6	; regulation of biological process	0.811	0.528	0.616
1196154	UDP-galactose-4-epimerase, isoform CRA_a [Homo sapiens]	7	27	metabolic process	1.124	0.526	0.596
3868693	P2X purinoceptor 4 isoform 4 [Homo sapiens]	2	4	cell organization and biogenesis	0.886	0.524	0.536
1195930	hCG2002525, isoform CRA_c [Homo sapiens]	3	7		1.104	0.522	0.467
4004097	unnamed protein product, partial [Homo sapiens]	2	8		1.015	0.520	0.600
1943827	unnamed protein product [Homo sapiens]	16	84	n of biological process; transport	1.021	0.518	0.525
1943875	unnamed protein product [Homo sapiens]	7	20		1.054	0.516	0.588
1582565	unnamed protein product [Homo sapiens]	3	15	transport; metabolic process	1.441	0.515	0.513
1195891	tripartite motif-containing 3, isoform CRA_f, partial [Homo sapiens	2	12	transport; metabolic process	1.193	0.514	0.561
1195945	solute carrier family 3 (activators of dibasic and neutral amino acid	15	65	s; cellular component movement	1.114	0.512	0.545
1937857	unnamed protein product [Homo sapiens]	9	42	n of biological process; transport	1.015	0.507	0.499
1879369	UDP-N-acetylhexosamine pyrophosphorylase-like protein 1 [Homo	3	7	metabolic process	1.211	0.505	0.517
3611297	Chain B, Solution Structure Of The Dimerization Domain Of Human	3	21	metabolic process	1.039	0.500	0.539
3044457	Chain A, Crystal Structure Of Human H Ferritin E140a Mutant	6	37	cellular homeostasis	1.013	0.497	0.582
5578786	RNA 3'-terminal phosphate cyclase-like protein isoform b [Homo s	2	3	metabolic process	0.964	0.497	0.548
1582612	unnamed protein product [Homo sapiens]	13	277	lic process; cellular homeostasis	1.279	0.496	0.536
2210432	unnamed protein product [Homo sapiens]	4	13	response to stimulus; cell death	0.931	0.495	0.488
3436524	hypothetical protein, partial [Homo sapiens]	3	8	n; cellular component movement	0.796	0.484	0.464
4003946	unnamed protein product, partial [Homo sapiens]	2	4		0.851	0.480	0.558
1196001	activated leukocyte cell adhesion molecule, isoform CRA_c [Homo	7	51	mponent movement; cell growth	0.856	0.478	0.459
7680457	PREDICTED: acetyl-CoA carboxylase 1 isoform X5 [Homo sapiens]	35	125	biogenesis; response to stimulus	1.052	0.477	0.487
544759	biliverdin-IX beta reductase isozyme I {EC 1.3.1.24} [human, liver, F	6	120	metabolic process	1.109	0.470	0.455
7679131	PREDICTED: lysophospholipid acyltransferase 2 isoform X4 [Homo	4	17	metabolic process	1.053	0.463	0.467
1427864	Chain C, Human Gm2 Activator Structure	3	6		1.321	0.461	0.562
6208774	brain glycogen phosphorylase variant, partial [Homo sapiens]	29	204	metabolic process	1.202	0.457	0.483
3838725	CD63 antigen isoform D precursor [Homo sapiens]	3	35	movement; response to stimulus	1.275	0.455	0.443
4502281	sodium/potassium-transporting ATPase subunit beta-3 [Homo sap	4	10	cell organization and biogenesis	1.136	0.455	0.542
1196082	carnitine acetyltransferase, isoform CRA_c [Homo sapiens]	14	49	transport; metabolic process	1.077	0.451	0.438
1195882	protein tyrosine phosphatase, receptor type, J, isoform CRA_a [Ho	2	4	al process; response to stimulus	1.126	0.449	0.434
520587	protein kinase C delta-type [Homo sapiens]	2	6	e; cellular component movement	0.971	0.446	0.400
4132773	epidermal growth factor receptor isoform c precursor [Homo sapie	2	7	l proliferation; defense response	0.786	0.445	0.473
182516	ferritin light subunit, partial [Homo sapiens]	3	7	cell organization and biogenesis	0.957	0.445	0.463
4601523	Chain D, Human SdhNAD+ COMPLEX	15	181	metabolic process	1.171	0.440	0.458
1583520	sorbitol dehydrogenase	15	181	metabolic process	1.171	0.440	0.458
3718327	C20orf3 [Homo sapiens]	14	102	metabolic process	1.198	0.439	0.459

1890961	Chain B, Human Liver Glycogen Phosphorylase (Tense State) In Co	6	46	metabolic process	1.290	0.433	0.460
9367826	LLPL, LCAT-like lysophospholipase, partial [Homo sapiens]	2	4	metabolic process	0.979	0.433	0.445
7680208	PREDICTED: neural cell adhesion molecule 2 isoform X8 [Homo sa	2	3		0.877	0.431	0.473
1196030	spondin 2, extracellular matrix protein, isoform CRA_a [Homo sapi	2	3	e response; response to stimulus	0.856	0.430	0.477
6208752	ABCC4 protein variant, partial [Homo sapiens]	3	7	; metabolic process; coagulation	1.055	0.430	0.431
5865004	Chain D, Structure Of Human Aldh4a1 Crystallized In Space Group	14	73		0.981	0.418	0.420
4758334	fatty acid desaturase 2 isoform 1 [Homo sapiens]	12	54	metabolic process; transport	1.041	0.414	0.442
1404394	FASN protein [Homo sapiens]	20	468	al process; response to stimulus	1.139	0.413	0.394
3306893	Chain D, The Structure Of The Fully Closed Conformation Of Huma	20	168	metabolic process	1.061	0.404	0.461
1195921	angiotensin II receptor-associated protein, isoform CRA_d [Homo	2	6	; regulation of biological process	0.872	0.388	0.414
1196140	transmembrane protein 106B, isoform CRA_c [Homo sapiens]	4	12	cell organization and biogenesis	1.094	0.386	0.395
1166428	prolactin-induced protein, partial [Homo sapiens]	2	4	n of biological process; transport	1.203	0.371	0.474
1937877	unnamed protein product [Homo sapiens]	3	7	roliferation; response to stimulus	1.062	0.370	0.418
7679973	PREDICTED: fatty acid synthase isoform X1 [Homo sapiens]	103	3124	al process; response to stimulus	1.123	0.367	0.358
1195679	ribonuclease T2, isoform CRA_c [Homo sapiens]	5	18	metabolic process	1.244	0.365	0.443
7679734	PREDICTED: putative phospholipase B-like 2 isoform X1 [Homo sa	3	9	metabolic process	0.837	0.362	0.342
1890533	unnamed protein product [Homo sapiens]	15	52	metabolic process	0.947	0.361	0.338
1195702	stearoyl-CoA desaturase (delta-9-desaturase), isoform CRA_c [Hor	5	19	metabolic process	1.230	0.359	0.450
3453132	unnamed protein product [Homo sapiens]	3	6		0.966	0.355	0.356
2560323	Chain B, Crystal Structure Of Phosphorylated Mimic Form Of Huma	7	24	metabolic process	1.045	0.348	0.382
1049053	encodes region of fatty acid synthase activity; FAS; multifunctiona	70	2198	al process; response to stimulus	1.105	0.343	0.335
6459859	Chain C, Human D-dt Complexed With Tartrate	3	7		1.215	0.342	0.373
1196193	spermine synthase, isoform CRA_c [Homo sapiens]	4	17	metabolic process	1.077	0.340	0.356
1404212	unnamed protein product [Homo sapiens]	2	3	; regulation of biological process	1.688	0.338	0.384
1937853	unnamed protein product [Homo sapiens]	5	21	metabolic process	1.068	0.336	0.320
1196273	hCG2031650, isoform CRA_e [Homo sapiens]	7	23		1.111	0.334	0.366
3343592	Chain B, Crystal Structure Of A Fragment Of Fkbp51 Comprising T	2	7	metabolic process	1.331	0.324	0.317
1043396	unnamed protein product [Homo sapiens]	2	3	al process; response to stimulus	1.266	0.322	0.315
9296541	KIAA0830 protein [Homo sapiens]	6	16		1.252	0.322	0.289
5801855	CLN3 protein [Homo sapiens]	2	3	s; cellular component movement	0.959	0.320	0.513
1195743	ATP-binding cassette, sub-family C (CFTR/MRP), member 1, isofor	23	82	; transport; response to stimulus	1.029	0.319	0.314
1582615	unnamed protein product [Homo sapiens]	7	20	metabolic process; transport	1.194	0.316	0.343
7679037	PREDICTED: F-box only protein 6 isoform X1 [Homo sapiens]	3	6	e to stimulus; metabolic process	0.972	0.316	0.339
4004135	unnamed protein product, partial [Homo sapiens]	4	29		1.235	0.306	0.317
1196161	microtubule-associated protein 1B, isoform CRA_b [Homo sapiens	6	17	novement; transport; cell growth	0.788	0.298	0.318
1196162	serine incorporator 5, isoform CRA_a [Homo sapiens]	2	6	lus; transport; defense response	0.816	0.294	0.291
7679330	PREDICTED: type II inositol 3,4-bisphosphate 4-phosphatase isofo	3	5	al process; response to stimulus	0.841	0.294	0.283
9146850	Chain A, Human Cyclophilin D Complexed With An Inhibitor At Roo	3	5	metabolic process	1.323	0.292	0.361
7679229	PREDICTED: leucine-rich repeats and immunoglobulin-like domair	2	3		0.801	0.291	0.325
1196196	tetraspanin 3, isoform CRA_a [Homo sapiens]	3	10	al process; response to stimulus	0.854	0.280	0.291

179034	androgen-receptor [Homo sapiens]	2	3	cell organization and biogenesis	1.012	0.274	0.296
6973516	Chain C, Enoyl-acyl Carrier Protein-reductase Domain From Humai	15	408		1.255	0.274	0.278
1582613	unnamed protein product [Homo sapiens]	2	2	; regulation of biological process	0.948	0.258	0.290
1196212	apolipoprotein B (including Ag(x) antigen), isoform CRA_b [Homo	4	16	s; cellular component movement	1.087	0.244	0.237
2210462	unnamed protein product [Homo sapiens]	4	24	cell organization and biogenesis	1.210	0.239	0.265
7679008	PREDICTED: inactive gamma-glutamyltranspeptidase 2 isoform X2	6	17		0.906	0.232	0.232
1196248	ELOVL family member 5, elongation of long chain fatty acids (FEN	2	10	metabolic process	0.917	0.193	0.190
2210441	unnamed protein product, partial [Homo sapiens]	7	17	cell organization and biogenesis	1.034	0.185	0.224
1341044	Chain B, Crystal Structure Of Human Mu_crystallin At 2.6 Angstror	3	7	metabolic process	1.419	0.184	0.206
7680216	PREDICTED: transmembrane protease serine 2 isoform X4 [Homo	4	12	; regulation of biological process	1.073	0.182	0.177
3213415	unnamed protein product [Homo sapiens]	2	10		1.040	0.171	0.202
5566959	Chain A, Structural Analysis Of Neprilysin With Various Specific Ar	12	44	metabolic process	1.300	0.118	0.125
